# Supplementary material for: Ynimines as versatile precursors to 2-imido- and 2-amido-1,3-dienes for stereodivergent diels–alder reactions
Source: Nat Commun. 2026 Mar 16;17:4031. doi: 10.1038/s41467-026-70363-w (PMC13139502; doi:10.1038/s41467-026-70363-w)
Supplement: Supplementary file 1 — Supplementary Information [file 41467_2026_70363_MOESM1_ESM.pdf]

Supplementary Information

Ynimines as Versatile Precursors to 2-Imido- and 2-Amido-1,3-Dienes for Stereodivergent Diels–Alder Reactions

Ruijia Wang,[a,b] Xin-Qi Zhu,[b,c] Maël Djaïd,[b] Rémi Lavernhe,[b] Qian Wang,[b] Jieping Zhu[b*]

[a] Dr. R. Wang

School of Pharmacy, Hunan University of Chinese Medicine, Changsha 410208, PR China

[b] Maël Djaïd, Dr. R. Lavernhe, Dr. Q. Wang, Prof. Dr. J. Zhu

Laboratory of Synthesis and Natural Products (LSPN), Institute of Chemical Sciences and Engineering

Ecole Polytechnique Fédérale de Lausanne, EPFL-SB-ISIC-LSPN, BCH 5304, 1015 Lausanne (Switzerland)

E-mail: jieping.zhu@epfl.ch

Homepage: http://lspn.epfl.ch

[c] Prof. Dr. X.-Q. Zhu

Yunnan Key Laboratory of Modern Separation Analysis and Substance Transformation, College of Chemistry and Chemical Engineering, Yunnan Normal University, Kunming, 650500, P. R. China

*Correspondence to: jieping.zhu@epfl.ch

Table of Contents

1 General Information

2 Synthesis and characterization data of the starting materials **S4-S17**

Figure S1 Structures of ynimines **4**
Figure S2 Synthesis of ynimines **4y**
Figure S3 Synthesis of ynimines **15a-15d**
Figure S4 Synthesis of dienophiles **3d-3l**
characterization data of the ynimines and dienophiles

3 Condition optimization of the Diels-Alder reaction **S17-S20**

Condition optimization for the reaction of the β-aryl substituted α,β-unsaturated ynimines
Table S1: Solvent screening
Table S2: Temperature screening
Condition optimization for the reaction of the β-alkyl substituted α,β-unsaturated ynimines
Table S3: Temperature screening
Table S4: Base screening
Condition optimization of the catalytic enantioselective Diels-Alder reaction
Table S5: Catalyst screening
Table S6: Temperature screening
Table S7: Screening of catalyst loading.

4 Synthesis and characterization data of the Diels-Alder reaction products **S20-S41**

Figure S5 Synthesis of **7a-7q**
Figure S6 Synthesis of **7r-7y**
Figure S7 Synthesis of **9a-9h**
Figure S8 Synthesis of **9i**
Figure S9 Synthesis of **16a-16d**
characterization data of the Diels-Alder reaction products

5 Copies of the SFC chromatograms (**9a-9i**) **S42-S50**

6 Crystallographic data **S51-S85,** Complementary Figures 10-12, Tables S8-S37

Crystallographic data for **7k** (CCDC 2260903)
Crystallographic data for **8a** (CCDC 2324899)
Crystallographic data for **9h** (CCDC 2324850)
Crystallographic data for **16b** (CCDC 2324851)

7 Copies of the NMR spectra **S86-S167**

8 References **S168**

1. General information. NMR spectra were recorded on AV2 400, AV2 500, AV2 600 or AV2 800 MHz Bruker spectrometers. Chemical shifts are given in ppm. The spectra are calibrated to the residual ^{1}H and ^{13}C signals of the solvents. Chemical shifts (δ) were reported relative to residual solvent peaks (*$CDCl_3$ [^{1}H: 7.26, ^{13}C: 77.20;]; CD_3CN [^{1}H: 1.94, ^{13}C: 1.32 (CD_3)]*]).Multiplicities are abbreviated as follows: singlet (s), doublet (d), triplet (t), quartet (q), doublet-doublet (dd), quintet (quint), sextet (sext), septet (sept), multiplet (m), and broad (b). Infrared spectra were recorded on a JASCO FT/IR-4100 spectrometer. Mass spectra were determined with a Waters ACQUITY H-class UPLC/MS ACQ-SQD by electron ionization (EI positive and negative) or a Finnigan TSQ7000 by electrospray ionization (ESI+). The accurate masses were measured by the mass spectrometry service of the EPFL by ESI-TOF using a QTOF Ultima from Waters or APPI-FT-ICR using a linear ion trap Fourier transform ion cyclotron resonance mass spectrometer from Thermo Scientific. Optical rotations α_D were obtained with a Jasco P-2000 polarimeter (589 nm). Enantiomeric excesses were determined with a Thar SFC Investigator system using chiral stationary phase columns by comparing the samples with the appropriate racemic samples, column and elution details specified in each entry. Melting points were measured using a Stuart SMP30.

Materials and Methods: Unless otherwise stated, starting materials were purchased from commercial sources (Aldrich, Acros, Merck, Fluka and VWR international). More sensitive compounds were stored in a desiccator or in a glove-box if required. Solvents were purchased in HPLC quality, degassed by purging thoroughly with nitrogen and dried over activated molecular sieves of appropriate size. Alternatively, they were purged with argon and passed through alumina columns in a solvent purification system (Innovative Technology). Molecular sieves were activated in a vacuum drying oven at 200 ℃. Reactions were monitored by thin layer chromatography (TLC) using Merck TLC silica gel 60 F254. Compounds were visualized by UV-light at 254 nm and by dipping the plates in an ethanolic vanillin/sulfuric acid solution or an aqueous potassium permanganate solution followed by heating. Flash column chromatography was performed over silica gel (230-400 mesh). The $CDCl_3$ used in the NMR experiments was stored over anhydrous K_2CO_3 before use.

2. Synthesis and characterization data of the starting materialsF

Figure S1: Structures of ynimines **4** and Synthesis of **4a-4m, 4r, 4x**

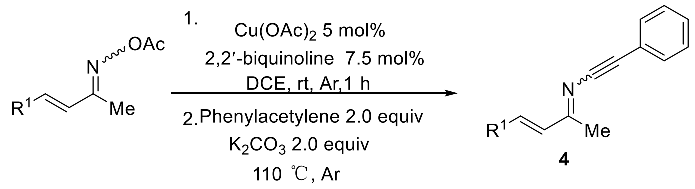

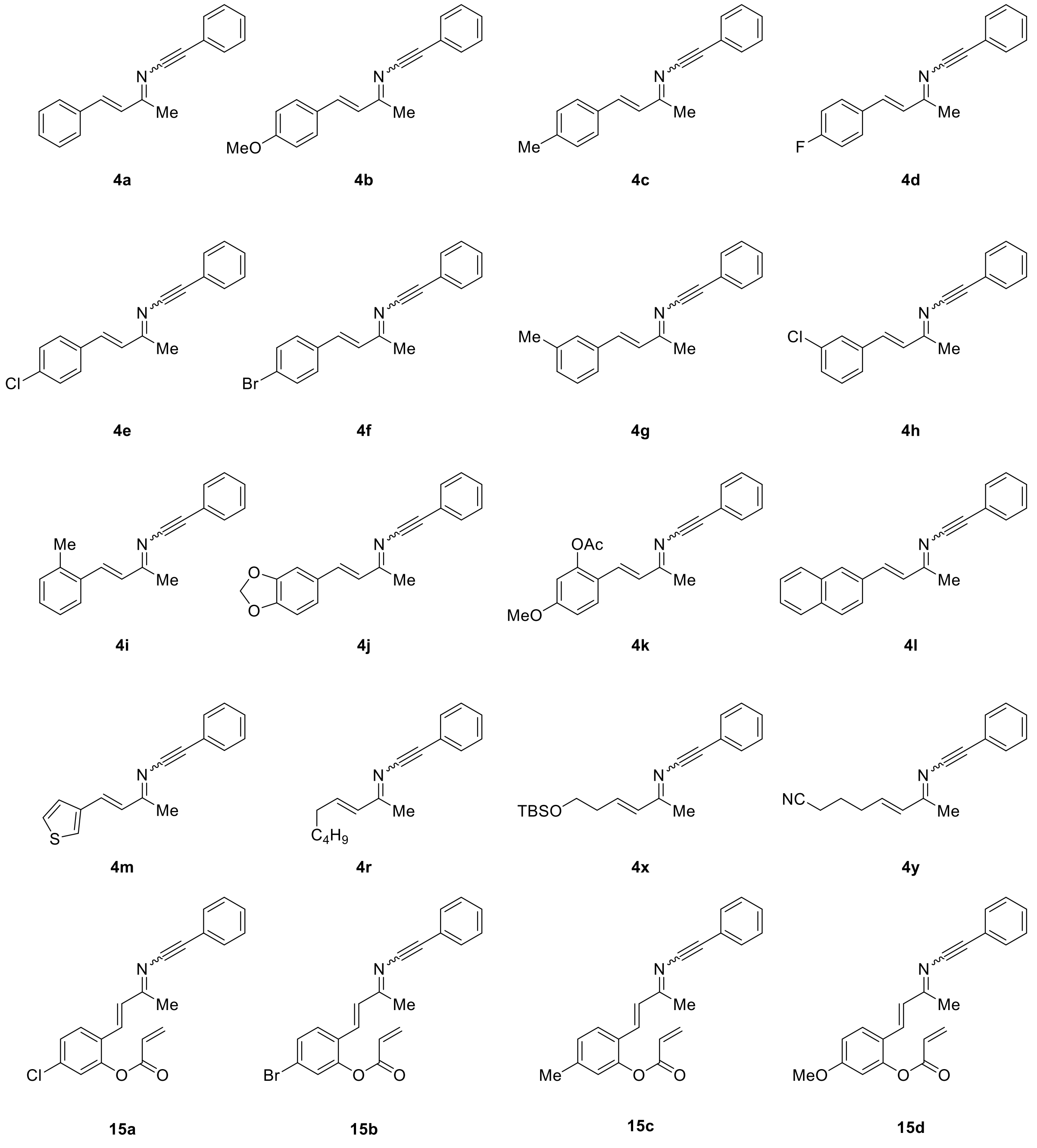

A solution of $Cu(OAc)_2$ (5% mmol) and 2,2'-biquinoline (7.5% mmol) in degassed DCE was stirred vigorously at 25 °C for 40 min under argon. The resulting solution was then added to a screw-capped vessel containing the acetyl oxime, K_2CO_3 (2 equiv) and the alkyne (2 equiv) under argon. The vial was sealed with a Teflon-lined cap (under a flow of argon) and stirred at 110 °C for 10 - 48 h. After completion, the crude reaction mixture was filtered through a plug of Celite, concentrated under reduced pressure, and purified by flash chromatography on deactivated silica gel to afford the desired alkynylated product **4.**[1]

Figure S2: Synthesis of **4y**

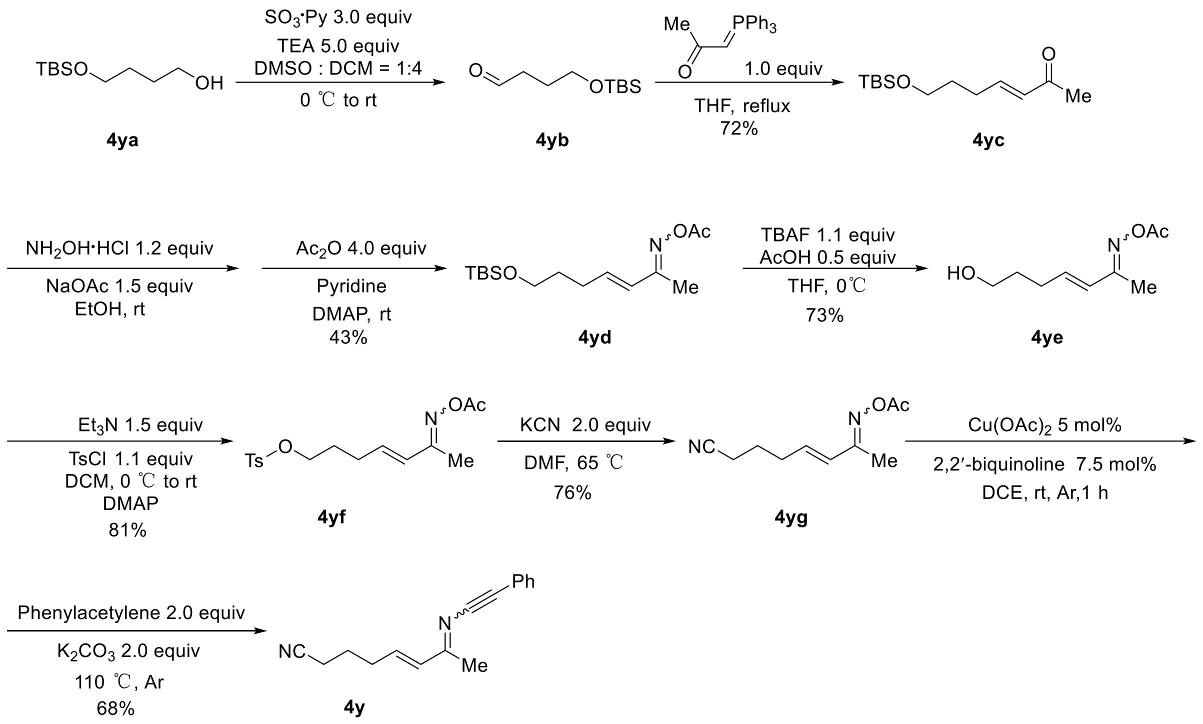

To a solution of 4-((*tert*-butyldimethylsilyl)oxy)butan-1-ol (**4ya**) (8.16 g, 40.0 mmol, 1.0 equiv) and Et_3N (28.0 mL, 200.0 mmol, 5.0 equiv) in DCM (120 mL) and DMSO (30.0 mL) was added SO_3Py (19.1 g, 120.0 mmol, 3.0 equiv) at room temperature under N_2 atmosphere and the mixture was stirred until the starting material was completely consumed. The reaction mixture was poured into 5% HCl aqueous solution and extracted with DCM. The combined organic phases were washed with water and brine, dried over $MgSO_4$, filtered, and concentrated under vacuum. The obtained aldehyde **4yb** was directly used for the next step without further purification.[2]

To a solution of aldehyde **4yb** in THF (30 mL) was added 1-(triphenylphosphoranylidene)-2-propanone (7.0 g, 22 mmol, 1.1 equiv) at room temperature. The reaction mixture was refluxed for 8 h. Water was added and the reaction mixture was extracted with EtOAc. The combined organic layers were washed with water and brine, dried over $MgSO_4$ and concentrated under vacuum. The residue was purified by flash column chromatography to afford ketone **4yc**.

To a solution of hydroxylamine hydrochloride (2.09 g, 24.0 mmol, 1.2 equiv) and NaOAc (2.46 g, 30.0 mmol, 1.5 equiv) in EtOH (30 mL) was added ketone **4yc** (4.84 g, 20 mmol, 1.0 equiv). The mixture was stirred at room temperature for 2 h. EtOH was removed under reduced pressure and water was added. The mixture was extracted with EtOAc. The combined organic layers were washed with water and brine, dried over $MgSO_4$ and concentrated under vacuum. The obtained oxime was directly used for the next step without further purification.

To a solution of the oxime and DMAP in pyridine (20 mL, 1.0 M) was added acetic anhydride (7.6 mL, 80.0 mmol, 4.0 equiv). The mixture was stirred at room temperature until completion of the reaction. Water was added. The reaction mixture was extracted with EtOAc. The resulting solution was washed sequentially with 1N HCl (aq), saturated aqueous solution of $NaHCO_3$ and brine, dried over anhydrous $MgSO_4$, filtered, and concentrated under vacuum. The residue was purified by flash column chromatography to afford the oxime acetate **4yd**.

nBu_4NF (1.0 M in THF, 11.0 mL, 11.0 mmol, 1.1 equiv) was slowly added to a solution of the oxime acetate **4yd** (2.99 g, 10.0 mmol, 1.0 equiv) and acetic acid (0.3 g, 5.0 mmol, 0.5 equiv) in THF (20 mL) at 0 °C. The reaction mixture was stirred at 0 °C until the starting material was completely consumed. Water was added.

The reaction mixture was extracted with EtOAc and the combined organic phases were washed with water and brine, dried over $MgSO_4$, filtered, and concentrated under vacuum. The residue was purified by flash column chromatography to afford alcohol **4ye.**[3]

To a solution of (4*E*)-7-hydroxyhept-3-en-2-one *O*-acetyl oxime (**4ye**) (0.925 g, 5.0 mmol, 1.0 equiv), Et_3N (1.04 mL, 7.5 mmol, 1.5 equiv) and DMAP in DCM (10.0 mL (0.5 M)) at 0 °C was slowly added *p*-toluenesulfonyl chloride (1.05 g, 5.5 mmol, 1.1 equiv) over a period of 10 minutes. The resulting mixture was stirred at room temperature until the starting material was completely consumed. The reaction mixture was extracted with EtOAc. The combined organic phases were washed with water and brine, dried over $MgSO_4$, filtered, and concentrated under vacuum. The residue was purified by flash column chromatography to afford tosylate **4yf.**

A mixture of (4*E*)-6-(acetoxyimino)hept-4-en-1-yl 4-methylbenzenesulfonate (**4yf**) (1.7 g, 5.0 mol, 1.0 equiv) and KCN (0.65 g, 10.0 mol, 2.0 equiv) in DMF (10.0 mL 0.5 M) was stirred at 65°C until the starting material was completely consumed. The reaction mixture was extracted with EtOAc and the combined organic phases were washed with water and brine, dried over $MgSO_4$, filtered, and concentrated under vacuum. The residue was purified by flash column chromatography to afford nitrile **4yg.**[4] Finally, the corresponding ynimine **4y** was synthesized according to the reported procedure in 68% yield. [1]

Figure S3: Synthesis of **15a-15d**

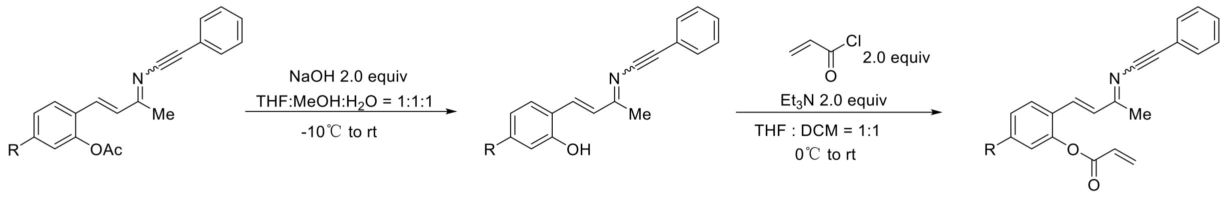

NaOH was added to a solution of 2-((1*E*)-3-((phenylethynyl)imino)but-1-en-1-yl)phenyl acetate in a mixture of THF/MeOH/H_2O (v:v:v 1:1:1) at -10 °C. The mixture was stirred until the starting material was completely consumed.[5] The reaction mixture was extracted with EtOAc then the combined organic phases were washed with water and brine, dried over $MgSO_4$, filtered, and concentrated under vacuum to give the crude phenol which was used directly for the next step without further purification. Acryloyl chloride was added dropwise to a solution of the above crude phenol and Et_3N in a mixture of THF/DCM (v:v 1:1) at 0 °C. The resulting solution was stirred at room temperature until the starting material was completely consumed. The reaction mixture was quenched with water and extracted with EtOAc. The combined organic phases were washed with water and brine, dried over $MgSO_4$, filtered, and concentrated under vacuum. The residue was purified by flash column chromatography to give ynimines **15a, 15b, 15c, 15d**.

Figure S4 Synthesis of dienophiles **3.**

Enones **3a**–**3c** are commercially available.

Synthesis of Enones **3d** and **3e.**[6]

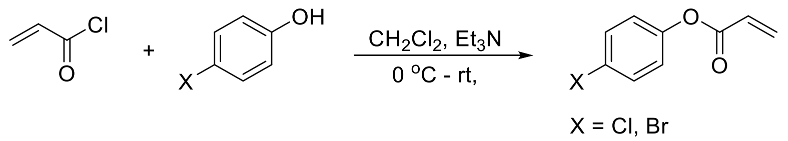

The phenol derivative (3.0 mmol) was dissolved in dry CH_2Cl_2 (10.0 mL) and Et_3N (4.5 mmol) was added. The reaction mixture was cooled to 0 °C in an ice-water bath. Then acryloyl chloride (3.6 mmol) was added dropwise. The mixture was warmed to room temperature and stirred until the starting material was

completely consumed. The solvent was removed under reduced pressure and the residue was purified by silica gel column chromatography to afford the desired product.

Synthesis of Enones **3f–3j**.[7]

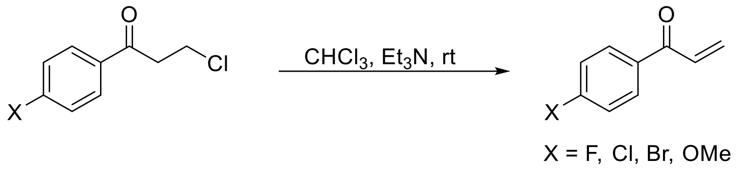

To a stirred solution of 3-chloropropiophenone (5.0 mmol) in $CHCl_3$ (10.0 mL) at room temperature was added Et_3N (10.0 mmol). The reaction mixture was stirred for 10 min, during which a white precipitate formed. This mixture was then diluted with saturated aqueous NH_4Cl, extracted with CH_2Cl_2. The combined organic phases were washed with water and brine, dried over $MgSO_4$, filtered, and concentrated under reduced pressure. The resulting residue was purified by flash column chromatography to afford the desired product.

Synthesis of Enones **3k–3l**.[8]

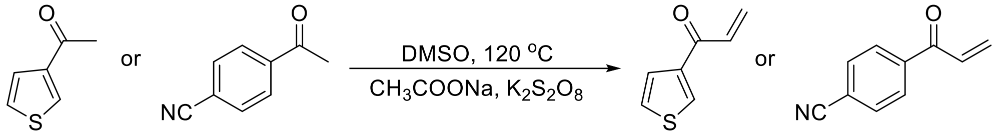

In a 25 mL Schlenk tube, CH_3COONa (1.0 mmol), acetophenone (0.5 mmol), and $K_2S_2O_8$ (1.0 mmol) were dissolved in DMSO (2.0 mL). The reaction mixture was stirred at 120 °C for 5 or 9 h. After completion, the mixture was cooled to room temperature and extracted with CH_2Cl_2. The combined organic phases were washed with water and brine, dried over $MgSO_4$, filtered, and concentrated under reduced pressure. The residue was purified by flash column chromatography to afford the desired product.

4-chlorophenyl acrylate (3d)

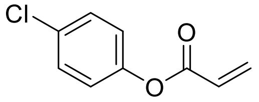

Compound **3d**: **Yield**: 95% (0.52 g), purified by column chromatography on silica gel (petroleum ether/ethyl acetate = 20/1).

^{1}H NMR (600 MHz, $CDCl_3$) δ 7.37 – 7.34 (m, 2H), 7.10 – 7.07 (m, 2H), 6.61 (dd, *J* = 17.3, 1.2 Hz, 1H), 6.31 (dd, *J* = 17.3, 10.5 Hz, 1H), 6.02 (dd, *J* = 10.5, 1.2 Hz, 1H).

^{13}C NMR (151 MHz, $CDCl_3$) δ 164.4, 149.2, 133.1, 131.3, 129.6, 127.7, 123.0.

IR ν (cm^{-1}) 1262 (s), 755 (s), 707 (s).

HRMS (ESI) m/z: $[M + H]^+$ Calcd for $C_9H_8ClO_2^+$ 183.0208; Found 183.0210.

4-bromophenyl acrylate (3e)

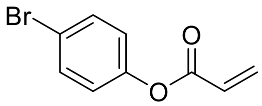

Compound **3e**: **Yield**: 96% (0.65 g), purified by column chromatography on silica gel (petroleum ether/ethyl acetate = 20/1).

^{1}H NMR (600 MHz, $CDCl_3$) δ 7.51 – 7.46 (m, 2H), 7.05 – 7.02 (m, 2H), 6.60 (dd, *J* = 17.3, 1.2 Hz, 1H), 6.30 (dd, *J* = 17.3, 10.5 Hz, 1H), 6.01 (dd, *J* = 10.5, 1.2 Hz, 1H).

^{13}C NMR (151 MHz, $CDCl_3$) δ 164.1, 149.7, 133.1, 132.5, 127.6, 123.4, 119.0.

IR ν (cm-1) 1264 (s), 745 (s), 704 (s).

HRMS (ESI) m/z: [M + H]+ Calcd for $C_9H_8BrO_2^+$ 226.9702; Found 226.9706.

1-phenylprop-2-en-1-one (3f)

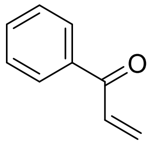

Compound **3f**: **Yield**: 93% (0.61 g), purified by column chromatography on silica gel (petroleum ether/ ethyl acetate = 20/1).

^{1}H NMR (600 MHz, $CDCl_3$) δ 7.95 (dd, *J* = 8.2, 1.4 Hz, 2H), 7.58 (tt, *J* = 7.5, 1.3 Hz, 1H), 7.48 (t, *J* = 7.7 Hz, 2H), 7.16 (dd, *J* = 17.1, 10.6 Hz, 1H), 6.44 (dd, *J* = 17.1, 1.6 Hz, 1H), 5.94 (dd, *J* = 10.5, 1.6 Hz, 1H).

^{13}C NMR (151 MHz, $CDCl_3$) δ 191.3, 137.5, 133.2, 132.6, 130.4, 128.9, 128.8.

IR ν (cm-1) 1264 (s), 735 (s), 704 (s).

HRMS (ESI) m/z: [M + H]+ Calcd for $C_9H_9O^+$ 133.0648; Found 133.0643.

1-(4-fluorophenyl)prop-2-en-1-one (3g)

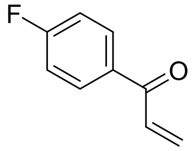

Compound **3g**: **Yield**: 90% (0.68 g), purified by column chromatography on silica gel (petroleum ether/ ethyl acetate = 20/1).

^{1}H NMR (600 MHz, $CDCl_3$) δ 8.00 – 7.96 (m, 2H), 7.17 – 7.11 (m, 3H), 6.43 (dd, *J* = 17.1, 1.6 Hz, 1H), 5.93 (dd, *J* = 10.6, 1.6 Hz, 1H).

^{13}C NMR (151 MHz, $CDCl_3$) δ 189.6, 165.9 (d, *J* = 254.8 Hz), 133.8 (d, *J* = 3.1 Hz), 132.2, 131.5 (d, *J* = 9.2 Hz), 130.5, 116.0 (d, *J* = 21.7 Hz).

^{19}F NMR (564 MHz, $CDCl_3$) δ -105.2.

IR ν (cm-1) 1264 (s), 735 (s), 705 (s).

HRMS (ESI) m/z: [M + H]+ Calcd for $C_9H_8FO^+$ 151.0554; Found 151.0551.

1-(4-chlorophenyl)prop-2-en-1-one (3h)

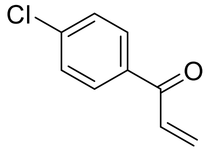

Compound **3h**: **Yield**: 90% (0.75 g), purified by column chromatography on silica gel (petroleum ether/ethyl acetate = 20/1).

^{1}H NMR (600 MHz, $CDCl_3$) δ 7.91 – 7.87 (m, 2H), 7.47 – 7.44 (m, 2H), 7.11 (dd, *J* = 17.1, 10.6 Hz, 1H), 6.44 (dd, *J* = 17.1, 1.6 Hz, 1H), 5.95 (dd, *J* = 10.6, 1.6 Hz, 1H).

^{13}C NMR (151 MHz, $CDCl_3$) δ 189.9, 139.7, 135.7, 132.1, 130.8, 130.3, 129.1.

IR ν (cm-1) 906 (s), 726 (s).

HRMS (ESI) m/z: [M + H]+ Calcd for $C_9H_8ClO^+$ 167.0258; Found 167.0255.

1-(4-bromophenyl)prop-2-en-1-one (3i)

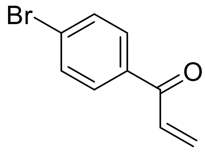

Compound **3i**: **Yield**: 92% (0.97 g), purified by column chromatography on silica gel (petroleum ether/ ethyl acetate = 20/1).

^{1}H NMR (600 MHz, $CDCl_3$) δ 7.80 (d, *J* = 7.7 Hz, 2H), 7.62 (d, *J* = 7.7 Hz, 2H), 7.10 (dd, *J* = 17.1, 10.6 Hz, 1H), 6.44 (brd, *J* = 17.1 Hz, 1H), 5.95 (brd, *J* = 10.6 Hz, 1H).

^{13}C NMR (151 MHz, $CDCl_3$) δ 190.11, 136.1, 132.12, 132.07, 130.9, 130.4, 128.3.

IR ν (cm^{-1}) 905 (s), 726 (s).

HRMS (ESI) m/z: $[M + H]^+$ Calcd for $C_9H_8BrO^+$ 210.9753; Found 210.9755.

1-(4-methoxyphenyl)prop-2-en-1-one (3j)

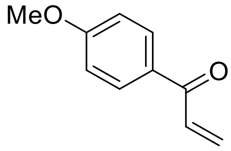

Compound **3j**: **Yield**: 90%(0.73 g), purified by column chromatography on silica gel (petroleum ether/ ethyl acetate = 20/1).

^{1}H NMR (600 MHz, $CDCl_3$) δ 7.96 – 7.93 (m, 2H), 7.15 (dd, *J* = 17.0, 10.5 Hz, 1H), 6.95 – 6.92 (m, 2H), 6.40 (dd, *J* = 17.1, 1.8 Hz, 1H), 5.85 (dd, *J* = 10.5, 1.8 Hz, 1H), 3.85 (s, 3H).

^{13}C NMR (151 MHz, $CDCl_3$) δ 189.4, 163.7, 132.3, 131.2, 130.3, 129.4, 114.0, 55.6.

IR ν (cm^{-1}) 1240 (s), 785 (s).

HRMS (ESI) m/z: $[M + H]^+$ Calcd for $C_{10}H_{11}O_2^+$ 163.0754; Found 163.0753.

4-acryloylbenzonitrile (3k)

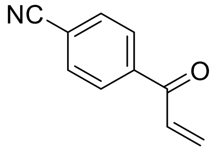

Compound **3k**: **Yield**: 65% (51 mg), purified by column chromatography on silica gel (petroleum ether/ ethyl acetate = 20/1).

^{1}H NMR (600 MHz, $CDCl_3$) δ 8.03 – 7.99 (m, 2H), 7.80 – 7.77 (m, 2H), 7.10 (dd, *J* = 17.1, 10.6 Hz, 1H), 6.47 (dd, *J* = 17.2, 1.3 Hz, 1H), 6.04 (dd, *J* = 10.6, 1.3 Hz, 1H).

^{13}C NMR (151 MHz, $CDCl_3$) δ 190.0, 140.6, 132.7, 132.1, 131.9, 129.2, 118.1, 116.4.

IR ν (cm^{-1}) 1263 (s), 747 (s), 707 (s).

HRMS (ESI) m/z: $[M + H]^+$ Calcd for $C_{10}H_8NO^+$ 158.0601; Found 158.0603.

1-(thiophen-3-yl)prop-2-en-1-one (3l)

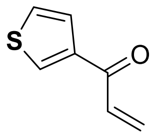

Compound **3l**: **Yield**: 55% (38 mg), purified by column chromatography on silica gel (petroleum ether/ethyl acetate = 20/1).

^{1}H NMR (600 MHz, $CDCl_3$) δ 8.09 (dd, *J* = 2.9, 1.3 Hz, 1H), 7.61 (dd, *J* = 5.1, 1.3 Hz, 1H), 7.35 (dd, *J* = 5.1, 2.9 Hz, 1H), 7.05 (dd, *J* = 17.1, 10.5 Hz, 1H), 6.46 (dd, *J* = 17.1, 1.6 Hz, 1H), 5.88 (dd, *J* = 10.5, 1.6 Hz, 1H).

^{13}C NMR (151 MHz, $CDCl_3$) δ 184.5, 142.4, 133.1, 132.8, 129.6, 127.6, 126.8.

IR ν (cm^{-1}) 1264 (s), 735 (s), 704 (s).

HRMS (ESI) m/z: $[M + H]^+$ Calcd for $C_7H_7OS^+$ 139.0212; Found 139.0212.

(2*E*,3*E*)-4-phenyl-N-(phenylethynyl)but-3-en-2-imine (4a)

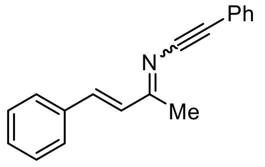

Compound **4a**: **Yield**: 59% (72.3 mg), yellow-orange solid as a 4:1 mixture of isomers, purified by column chromatography on silica gel (petroleum ether/ethyl acetate = 90/1).

^{1}H NMR (400 MHz, $CDCl_3$) Major isomer: δ 7.56 – 7.53 (m, 2H), 7.47 – 7.29 (m, 9H), 6.97 (d, J = 16.4 Hz, 1H), 2.50 (s, 3H).

^{13}C NMR (101 MHz, $CDCl_3$) Major isomer: δ 178.4, 140.0, 135.7, 131.4, 130.3, 129.8, 129.1, 128.5, 127.9, 127.9, 124.9, 97.3, 91.5, 19.2.

IR ν (cm^{-1}) 1543 (w), 1487 (w), 1442 (w), 1369 (w), 1072 (w), 958 (m), 912 (w), 746 (s), 688 (s)

HRMS (ESI/QTOF) m/z: $[M + H]^+$ Calcd for $C_{18}H_{16}N^+$ 246.1277; Found 246.1280.

(3*E*)-4-(4-methoxyphenyl)-N-(phenylethynyl)but-3-en-2-imine (4b)

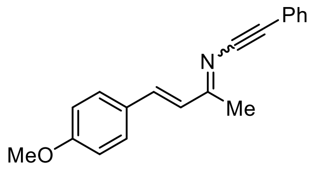

Compound **4b**: **Yield**: 72% (99.0 mg), yellow-orange solid as a 5:1 mixture of isomers, purified by column chromatography on silica gel (petroleum ether/ethyl acetate = 90/1).

^{1}H NMR (400 MHz, $CDCl_3$) major isomer: δ7.49 (d, *J* = 8.7 Hz, 2H), 7.46 – 7.43 (m, 2H), 7.36 – 7.28 (m, 3H), 6.92 (d, *J* = 8.8 Hz, 2H), 7.24 (d, *J* = 16.3 Hz, 1H), 6.85 (d, *J* = 16.3 Hz, 1H), 3.85 (s, 3H), 2.48 (s, 3H).

^{13}C NMR (101 MHz, $CDCl_3$) major isomer: δ 178.5, 161.1, 139.8, 131.4, 129.9, 129.5, 128.5, 128.1, 127.8, 125.1, 114.6, 96.0, 91.7, 55.6, 19.2.

IR (ν_{max}, cm^{-1}) 2154 (m), 2021 (w), 1603 (s), 1537 (m), 1512 (s), 1442 (m), 1306 (m), 1282 (m), 1248 (s), 1174 (m), 1028 (m), 964 (m), 816 (m), 752 (m), 690 (m).

HRMS (ESI/QTOF) m/z: $[M+H]^+$ Calcd for $C_{19}H_{18}NO^+$ 276.1383; Found 276.1383.

(3*E*)-N-(phenylethynyl)-4-(p-tolyl)but-3-en-2-imine (4c)

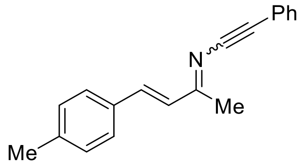

Compound **4c**: **Yield**: 80% (104 mg), yellow oil as a 5:1 mixture of isomers, purified by column chromatography on silica gel (petroleum ether/ethyl acetate = 90/1).

^{1}H NMR (500 MHz, $CDCl_3$) major isomer: δ 7.47-7.43 (m, 4H), 7.35-7.32 (m, 2H), 7.31-7.28 (m, 1H), 7.25 (d, *J* = 16.4 Hz, 1H), 7.22-7.19 (m, 2H), 6.93 (d, *J* = 16.4 Hz, 1H), 2.48 (s, 3H), 2.38 (d, *J* = 3.0 Hz, 3H).

^{13}C NMR (126 MHz, $CDCl_3$) major isomer: δ 178.5, 140.3, 140.2, 133.0, 131.4, 129.9, 129.4, 128.5, 127.89, 127.87, 125.0, 96.7, 91.6, 21.6, 19.2.

IR (ν_{max}, cm^{-1}) 2348 (w), 1603 (m), 1529 (m), 1486 (s), 1439 (s), 1374 (s), 1283(s), 1068 (w), 968 (s), 804 (s), 752 (s), 687 (s).

HRMS (ESI/QTOF) m/z: $[M + H]^+$ Calcd for $C_{19}H_{18}N^+$ 260.1434; Found 260.1437.

(3*E*)-4-(4-fluorophenyl)-N-(phenylethynyl)but-3-en-2-imine (4d)

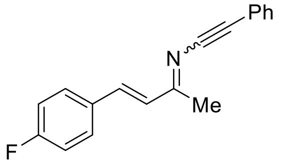

Compound **4d**: **Yield**: 49% (64.5 mg), yellow oil as a 5:1 mixture of isomers, purified by column chromatography on silica gel (petroleum ether/ethyl acetate = 90/1).

^{1}H NMR (400 MHz, $CDCl_3$) major isomer: δ 7.54-7.50 (m, 2H), 7.47-7.44 (m, 2H), 7.36-7.29 (m, 3H), 7.23 (d, *J* = 16.4 Hz, 1H), 7.12-7.05 (m, 2H), 6.88 (d, *J* = 16.4 Hz, 1H), 2.48 (s, 3H).

^{13}C NMR (101 MHz, $CDCl_3$) major isomer: δ 178.2, 163.6 (d, *J* = 250.8 Hz), 138.7, 131.9 (d, *J* = 3.5 Hz), 131.4, 130.05 (d, *J* = 8.0 Hz), 129.6 (d, *J* = 8.3 Hz), 128.5, 128.0, 124.9, 116.2 (d, *J* = 21.9 Hz), 97.4, 91.5, 19.3.

^{19}F NMR (376 MHz, $CDCl_3$) δ -110.4.

IR (ν_{max}, cm^{-1}) 2922(w), 1597 (s), 1534(m), 1508(s), 1232(s), 963(s), 819(s), 753 (s), 687(s).

HRMS (ESI/QTOF) m/z: $[M + H]^+$ Calcd for $C_{18}H_{15}FN^+$ 264.1183; Found 264.1187.

(3*E*)-4-(4-chlorophenyl)-N-(phenylethynyl)but-3-en-2-imine (4e)

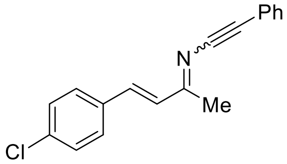

Compound **4e**: **Yield**: 37% (52 mg), yellow oil as a 6:1 mixture of isomers, purified by column chromatography on silica gel (petroleum ether/ethyl acetate = 90/1).

^{1}H NMR (400 MHz, $CDCl_3$) major isomer: δ 7.49-7.44 (m, 4H), 7.38-7.29 (m, 5H), 7.21 (d, *J* = 16.4 Hz, 1H), 6.92 (d, *J* = 16.4 Hz, 1H), 2.48 (s, 3H).

^{13}C NMR (101 MHz, $CDCl_3$) major isomer: δ 178.1, 138.5, 135.6, 134.2, 131.4, 130.8, 129.4, 129.0, 128.5, 128.0, 124.8, 98.0, 91.5, 19.3.

IR (ν_{max}, cm^{-1}) 3725(s), 2956(w), 1729 (m), 1537(s), 1488(s), 1439(s), 1372(m), 1280 (m), 1093(s), 964 (s), 811(s), 754 (s), 689(s).

HRMS (ESI/QTOF) m/z: $[M + H]^+$ Calcd for $C_{18}H_{15}ClN^+$ 280.0888; Found 280.0882.

(3*E*)-4-(4-bromophenyl)-N-(phenylethynyl)but-3-en-2-imine (4f)

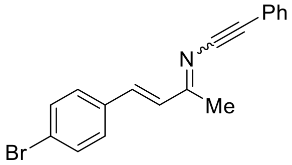

Compound 4f: Yield: 37% (70 mg), yellow oil as a 5:1 mixture of isomers, purified by column chromatography on silica gel (petroleum ether/ethyl acetate = 90/1).

^{1}**H NMR** (400 MHz, $CDCl_3$) major isomer: δ 7.54-7.51 (m, 2H), 7.47-7.43 (m, 2H), 7.41-7.39 (m, 2H), 7.36-7.31 (m, 3H), 7.20 (d, *J* = 16.4 Hz, 1H), 6.94 (d, *J* = 16.4 Hz, 1H), 2.48 (s, 3H).

^{13}**C NMR** (101 MHz, $CDCl_3$) major isomer: δ 178.1, 138.5, 134.6, 132.4, 131.4, 130.9, 129.2, 128.5, 128.1, 124.8, 124.0, 98.2, 91.5, 19.3.

IR (ν_{max}, cm^{-1}) 1733(m), 1537(s), 1485 (s), 1440 (S), 1401 (s), 1372(m), 1070 (s), 1007(s), 964 (s), 808(s), 754 (s), 689(s).

HRMS (ESI/QTOF) m/z: $[M + H]^+$ Calcd for $C_{18}H_{15}BrN^+$ 324.0382; Found 324.0388.

(3*E*)-N-(phenylethynyl)-4-(m-tolyl)but-3-en-2-imine (4g)

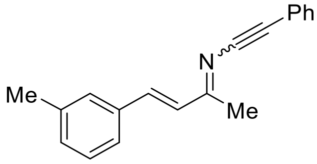

Compound **4g**: **Yield**: 46% (60 mg), yellow oil as a 5:1 mixture of isomers, purified by column chromatography on silica gel (petroleum ether/ethyl acetate = 90/1).

^{1}**H NMR** (400 MHz, $CDCl_3$) major isomer: δ 7.48-7.44 (m, 2H), 7.37-7.26 (m, 6H), 7.25 (d, *J* = 16.4 Hz, 1H), 7.18 (d, *J* = 7.4 Hz, 1H), 6.96 (d, *J* = 16.4 Hz, 1H), 2.49 (s, 3H), 2.39 (s, 3H).

^{13}**C NMR** (101 MHz, $CDCl_3$) major isomer: δ 178.5, 140.3, 138.7, 135.6, 131.4, 130.7, 130.1, 129.0, 128.6, 128.5, 127.9, 125.1, 124.9, 97.1, 91.6, 21.5, 19.2.

IR (ν_{max}, cm^{-1}) 3725(s), 3628(s), 2918(w), 2175(w), 1618(s), 1547(m), 1486(s), 1261(s), 966(s), 754(s), 689 (s).

HRMS (ESI/QTOF) m/z: $[M + H]^+$ Calcd for $C_{19}H_{18}N^+$ 260.1434; Found 260.1437.

(3*E*)-4-(3-chlorophenyl)-N-(phenylethynyl)but-3-en-2-imine (4h)

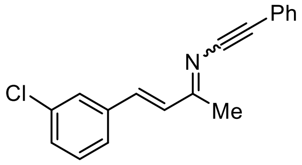

Compound **4h**: **Yield**: 37% (52 mg), yellow solid as a 5:1 mixture of isomers, purified by column chromatography on silica gel (petroleum ether/ethyl acetate = 90/1).

^{1}**H NMR** (400 MHz, $CDCl_3$) major isomer: δ 7.52 (brs, 1H), 7.47-7.41 (m, 3H), 7.37-7.29 (m, 5H), 7.20 (d, *J* = 16.4 Hz, 1H), 6.95 (d, *J* = 16.4 Hz, 1H), 2.48 (s, 3H).

^{13}**C NMR** (101 MHz, $CDCl_3$) major isomer: δ 178.0, 138.3, 137.6, 135.1, 131.6, 131.4, 130.4, 129.7, 128.6, 128.1, 127.8, 125.9, 124.8, 98.5, 91.4, 19.4.

IR (ν_{max}, cm^{-1}) 1620 (w), 1593 (m), 1562 (m), 1547 (m), 1485 (m), 1423 (m), 1371 (m), 1292 (m), 1201 (m), 1095 (w), 1076 (m), 962 (s), 906 (w), 885 (m), 779 (s), 754 (s), 687 (s).

HRMS (ESI/QTOF) m/z: $[M+H]^+$ Calcd for $C_{18}H_{15}ClN^+$ 280.0888; Found 280.0884.

(3*E*)-N-(phenylethynyl)-4-(o-tolyl)but-3-en-2-imine (4i)

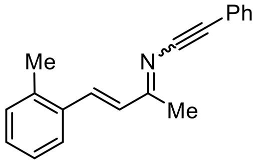

Compound **4i**: **Yield**: 52% (67.4 mg), yellow solid as a 5:1 mixture of isomers, purified by column chromatography on silica gel (petroleum ether/ethyl acetate = 90/1).

^{1}H NMR (400 MHz, $CDCl_3$) major isomer: δ 7.64-7.60 (m, 1H), 7.55 (d, *J* = 16.3 Hz, 1H), 7.47-7.44 (m, 2H), 7.36-7.29 (m, 3H), 7.26-7.19 (m, 3H), 6.89 (d, *J* = 16.3 Hz, 1H), 2.51 (s, 3H), 2.46 (s, 3H).

^{13}C NMR (101 MHz, $CDCl_3$) major isomer: δ 178.5, 137.5, 137.2, 134.6, 131.4, 131.3, 131.0, 129.7, 128.5, 128.0, 126.7, 126.3, 124.9, 97.2, 91.5, 20.0, 19.4.

IR (ν_{max}, cm^{-1}) 2181 (w), 2171 (w), 1616 (m), 1599 (w), 1545 (m), 1487 (m), 1371 (m), 1296 (m), 966 (m), 750 (s), 731 (m), 690 (m).

HRMS (ESI/QTOF) m/z: $[M+H]^+$ Calcd for $C_{19}H_{18}N^+$ 260.1434; Found 260.1436.

(3*E*)-4-(benzo[d][1,3]dioxol-5-yl)-N-(phenylethynyl)but-3-en-2-imine (4j)

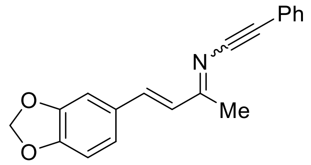

Compound **4j**: **Yield**: 40% (58 mg), yellow oil as a 5:1 mixture of isomers, purified by column chromatography on silica gel (petroleum ether/ethyl acetate = 40/1).

^{1}H NMR (400 MHz, $CDCl_3$) major isomer: δ 7.46-7.43 (m, 2H), 7.37-7.27 (m, 3H), 7.19 (d, *J* = 16.3 Hz, 1H), 7.07 (d, *J* = 1.8 Hz, 1H), 7.00 (dd, *J* = 8.2, 1.7 Hz, 1H), 6.84 (d, *J* = 7.4 Hz, 1H), 6.80 (d, *J* = 16.3 Hz, 1H), 6.01 (s, 2H), 2.46 (s, 3H).

^{13}C NMR (101 MHz, $CDCl_3$) major isomer: δ 178.3, 149.3, 148.6, 139.8, 131.4, 130.2, 128.5, 127.9, 125.0, 124.1, 108.8, 106.4, 101.7, 96.5, 91.7, 19.3.

IR (ν_{max}, cm^{-1}) 2908(w), 1737 (m), 1599 (S), 1500 (m), 1444 (s), 1253 (s), 1034(s), 963 (s), 925 (s), 799 (s), 754 (s), 688 (s).

HRMS (ESI/QTOF) m/z: $[M + H]^+$ Calcd for $C_{19}H_{16}NO_2^+$ 290.1176; Found 290.1174.

5-methoxy-2-((1*E*)-3-((phenylethynyl)imino)but-1-en-1-yl)phenyl acetate (4k)

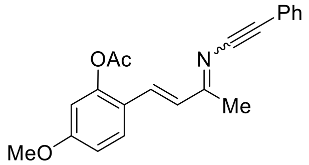

Compound **4k**: **Yield**: 50% (83 mg), yellow oil as a 5:1 mixture of isomers, purified by column chromatography on silica gel (petroleum ether/ethyl acetate = 40/1).

^{1}H NMR (400 MHz, $CDCl_3$) major isomer: δ 7.60 (d, *J* = 8.8 Hz, 1H), 7.47-7.44 (m, 2H), 7.35-7.28 (m, 3H), 7.24 (d, *J* = 16.5 Hz, 1H), 6.86 (d, *J* = 16.7 Hz, 1H), 6.85-6.81 (m, 1H), 6.66 (d, *J* = 2.6 Hz, 1H), 3.81 (s, 3H), 2.44 (s, 3H), 2.38 (s, 3H).

^{13}C NMR (101 MHz, $CDCl_3$) major isomer: δ 178.2, 169.1, 161.6, 150.2, 132.9, 131.3, 129.8, 128.44, 128.41, 127.8, 124.9, 120.8, 113.0, 108.6, 97.0, 91.6, 55.7, 21.1, 19.1.

IR (ν_{max}, cm^{-1}) 2960(w), 1767(s), 1610(s), 1543 (s), 1502(s), 1488 (S), 1285 (s), 1262 (s), 1200(m), 1155(s), 1102(s), 966 (s), 891 (s), 755(s), 690(s).

HRMS (ESI/QTOF) m/z: $[M + H]^+$ Calcd for $C_{21}H_{20}NO_3^+$ 334.1438; Found 334.1430.

(3*E*)-4-(naphthalen-2-yl)-N-(phenylethynyl)but-3-en-2-imine (4l)

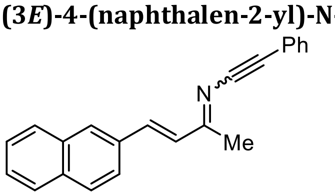

Compound **4l**: **Yield**: 41% (60 mg), yellow oil as a 5:1 mixture of isomers, purified by column chromatography on silica gel (petroleum ether/ethyl acetate = 60/1).

^{1}H NMR (400 MHz, $CDCl_3$) major isomer: δ 7.91 (s, 1H), 7.88-7.81 (m, 3H), 7.74-7.70 (m, 1H), 7.54-7.48 (m, 4H), 7.43 (d, *J* = 16.4 Hz, 1H), 7.39-7.30 (m, 3H), 7.09 (d, *J* = 16.4 Hz, 1H), 2.53 (s, 3H).

^{13}C NMR (101 MHz, $CDCl_3$) major isomer: δ 178.4, 140.1, 134.1, 133.6, 133.2, 131.4, 130.5, 129.4, 128.9, 128.6, 128.5, 127.94, 127.92, 127.2, 126.8, 124.9, 123.6, 97.5, 91.7, 19.3.

IR (ν_{max}, cm^{-1}) 2956(w), 2925(w), 1725 (m), 1614 (S), 1545 (s), 1486(m), 1370 (m), 1287(m), 1125 (s), 1071 (s), 963 (s), 813(s), 754 (m), 690(s).

HRMS (ESI/QTOF) m/z: $[M + H]^+$ Calcd for $C_{22}H_{18}N^+$ 296.1434; Found 296.1444.

(*3E*)-*N*-(phenylethynyl)-4-(thiophen-3-yl)but-3-en-2-imine (4m)

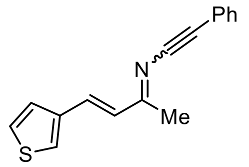

Compound **4m**: **Yield**: 41% (84.0 mg), yellow solid as a 5:1 mixture of isomers, purified by column chromatography on silica gel (petroleum ether/ethyl acetate = 90/1).

^{1}H NMR (400 MHz, $CDCl_3$) major isomer: δ 7.48-7.41 (m, 3H), 7.38-7.26 (m, 6H), 6.80 (d, *J* = 16.3 Hz, 1H), 2.46 (s, 3H).

^{13}C NMR (101 MHz, $CDCl_3$) major isomer: δ 178.5, 139.0, 133.7, 131.4, 130.3, 128.5, 127.9, 127.2, 126.8, 125.3, 125.0, 96.8, 91.6, 19.1.

IR (ν_{max}, cm^{-1}) 1614 (m), 1545 (m), 1487 (m), 1371 (m), 1308 (m), 1288 (m), 1246 (w), 962 (m), 775 (s), 756 (s), 688 (s).

HRMS (ESI/QTOF) m/z: $[M+H]^+$ Calcd for $C_{16}H_{14}NS^+$ 252.0841; Found 252.0840.

(3*E*)-N-(phenylethynyl)non-3-en-2-imine (4r)

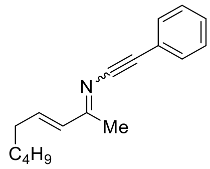

Compound **4r**: **Yield**: 58% (69.4 mg), yellow oil as a 3:1 mixture of isomers, purified by column chromatography on silica gel (petroleum ether/ethyl acetate = 90/1).

^{1}H NMR (400 MHz, $CDCl_3$) major isomer: δ 7.44-7.40 (m, 2H), 7.34-7.25 (m, 3H), 6.53 (dt, *J* = 15.9, 7.1 Hz, 1H), 6.27 (dt, *J* = 16.0, 1.5 Hz, 1H), 2.35 (s, 3H), 2.29-2.24 (m, 2H), 1.53-1.45 (m, 2H), 1.36-1.29 (m, 4H), 0.93 – 0.89 (m, 3H).

^{13}C NMR (101 MHz, $CDCl_3$) major isomer: δ 178.7, 144.8, 133.0, 131.3, 128.5, 127.7, 125.0, 94.6, 91.0, 33.3, 31.6, 28.5, 22.7, 19.1, 14.2.

IR (ν_{max}, cm^{-1}) 3059 (w), 3041 (w), 2954 (m), 2924 (s), 2866 (m), 2179 (m), 1631 (m), 1550 (s), 1487 (s), 1460 (m), 1437 (m), 1412 (m), 1371 (s), 1288 (m), 1076 (m), 1024 (m), 972 (s), 748 (s), 737 (s), 687 (s).

HRMS (ESI/QTOF) m/z: $[M+H]^+$ Calcd for $C_{17}H_{22}N^+$ 240.1747; Found 240.1743.

(3*E*)-6-((tert-butyldimethylsilyl)oxy)-N-(phenylethynyl)hex-3-en-2-imine (4x)

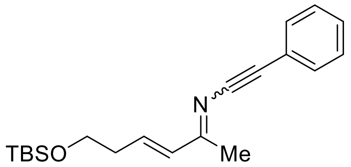

Compound **4x**: **Yield**: 43% (70 mg), yellow oil as a 3:1 mixture of isomers, purified by column chromatography on silica gel (petroleum ether/ethyl acetate = 90/1).

^{1}H NMR (400 MHz, $CDCl_3$) major isomer: δ 7.46-7.39 (m, 2H), 7.34-7.26 (m, 3H), 6.59– 6.51 (m, 1H), 6.33 (dt, *J* = 16.0, 1.4 Hz, 1H), 3.76 (td, *J* = 6.3, 4.2 Hz, 2H), 2.51-2.46 (m, 2H), 2.36 (s, 3H), 0.91 (s, 9H), 0.08 (s, 6H).

^{13}C NMR (101 MHz, $CDCl_3$) major isomer: δ 178.4, 141.2, 134.5, 131.3, 128.4, 127.8, 124.9, 95.2, 90.9, 62.2, 36.7, 26.0, 19.0, 18.5, -5.2.

IR ($ν_{max}$, cm^{-1}) 2927(w), 2854(w), 2180(s), 1557(m), 1488(s), 1371(m), 1253 (s), 1094 (m), 970(s), 832(s), 775(s), 753(s), 689(s).

HRMS (ESI/QTOF) m/z: $[M + H]^+$ Calcd for $C_{20}H_{30}NOSi^+$ 328.2091; Found 328.2091.

(5*E*)-7-((phenylethynyl)imino)oct-5-enenitrile (4y)

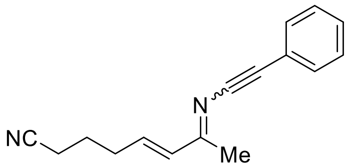

Compound **4y**: **Yield**: 68% (80.2 mg), yellow oil as a 3:1 mixture of isomers, purified by column chromatography on silica gel (petroleum ether/ethyl acetate = 90/1).

^{1}H NMR (400 MHz, $CDCl_3$) major isomer: δ 7.44-7.41 (m, 2H), 7.35-7.27 (m, 3H), 6.50-6.42 (m, 1H), 6.32 (dt, *J* = 16.0, 1.3 Hz, 1H), 2.47-2.38 (m, 4H), 2.36 (s, 3H), 1.91-1.84 (m, 2H).

^{13}C NMR (101 MHz, $CDCl_3$) major isomer: δ 177.9, 140.6, 134.6, 131.3, 128.5, 128.0, 124.7, 119.3, 96.1, 90.7, 31.8, 24.5, 19.3, 16.8.

IR ($ν_{max}$, cm^{-1}) 2930(w), 2246(s), 1717(w), 1675(w), 1636(s), 1559(s), 1488(s), 1449(s), 1371(s), 1287(w), 1252(w), 1177(s), 972(s), 832(s), 757(s), 692(s).

HRMS (APCI/QTOF) m/z: $[M + H]^+$ Calcd for $C_{16}H_{17}N_2^+$ 237.1386; Found 237.1390.

5-chloro-2-((1*E*)-3-((phenylethynyl)imino)but-1-en-1-yl)phenyl acrylate (15a)

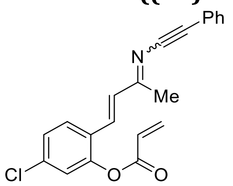

Compound **15a**: **Yield**: 15% (40 mg), yellow oil as a 5:1 mixture of isomers, purified by column chromatography on silica gel (petroleum ether/ethyl acetate = 90/1).

^{1}H NMR (400 MHz, $CDCl_3$) major isomer: δ 7.62 (d, *J* = 8.5 Hz, 1H), 7.46-7.43 (m, 2H), 7.36-7.20 (m, 6H), 6.93 (d, *J* = 16.5 Hz, 1H), 6.70 (d, *J* = 16.4 Hz, 1H), 6.40 (dd, *J* = 17.3, 10.5 Hz, 1H), 6.13 (d, *J* = 10.4 Hz, 1H), 2.42 (s, 3H).

^{13}C NMR (101 MHz, $CDCl_3$) major isomer: δ 177.8, 163.9, 149.1, 135.8, 134.2, 132.6, 131.7, 131.4, 131.2, 128.5, 128.1, 127.2, 127.12, 127.11, 124.7, 123.7, 99.0, 91.4, 19.2.

IR ($ν_{max}$, cm^{-1}) 3064(w), 2921(w), 1748 (s), 1697 (s), 1604(m), 1485(s), 1400(s), 1221(s), 1184(s), 1138(s), 1080(s), 983(s), 800(s), 756(s), 699(s).

HRMS (ESI/QTOF) m/z: $[M + H]^+$ Calcd for $C_{21}H_{17}ClNO_2^+$ 350.0942; Found 350.0947.

5-bromo-2-((1*E*)-3-((phenylethynyl)imino)but-1-en-1-yl)phenyl acrylate (15b)

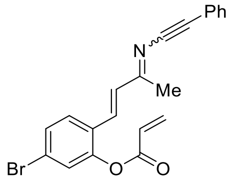

Compound **15b**: **Yield**: 13% (50 mg), yellow oil as a 6:1 mixture of isomers, purified by column chromatography on silica gel (petroleum ether/ethyl acetate = 90/1).

^{1}H NMR (600 MHz, $CDCl_3$) major isomer: δ 7.56 (d, *J* = 8.5 Hz, 1H), 7.45-7.42 (m, 3H), 7.39-7.28 (m, 4H), 7.21 (d, *J* = 16.5 Hz, 1H), 6.94 (d, *J* = 16.5 Hz, 1H), 6.70 (dd, *J* = 17.3, 1.0 Hz, 1H), 6.40 (dd, *J* = 17.3, 10.5 Hz, 1H), 6.13 (dd, *J* = 10.5, 1.1 Hz, 1H), 2.42 (s, 3H).

^{13}C NMR (151 MHz, $CDCl_3$) major isomer: δ 177.9, 164.0, 149.1, 134.3, 132.7, 131.8, 131.4, 130.0, 128.5, 128.3, 128.2, 127.6, 127.1, 126.5, 124.7, 123.6, 99.0, 91.4, 19.2.

IR (ν_{max}, cm^{-1}) 2924(w), 1541 (s), 1487 (s), 1398(s), 1213(s), 1137(s), 970(s), 912(s), 754(s), 690(s).

HRMS (ESI/QTOF) m/z: $[M + H]^+$ Calcd for $C_{21}H_{17}BrNO_2^+$ 394.0437; Found 394.0431.

5-methoxy-2-((1*E*)-3-((phenylethynyl)imino)but-1-en-1-yl)phenyl acrylate (15c)

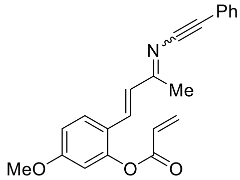

Compound **15c**: **Yield**: 24% (82.8 mg), yellow oil as a 5:1 mixture of isomers, purified by column chromatography on silica gel (petroleum ether/ethyl acetate = 90/1).

^{1}H NMR (600 MHz, $CDCl_3$) major isomer: δ 7.64 (d, *J* = 8.8 Hz, 1H), 7.45-7.43 (m, 2H), 7.33 (t, *J* = 7.5 Hz, 2H), 7.29-7.27 (m, 1H), 7.23 (d, *J* = 16.4 Hz, 1H), 6.86 (d, *J* = 16.6 Hz, 1H), 6.87-6.85 (m, 1H), 6.70 (d, *J* = 2.6 Hz, 1H), 6.69 (d, *J* = 17.3 Hz, 1H), 6.41 (dd, *J* = 17.3, 10.5 Hz, 1H), 6.12 (dd, *J* = 10.5, 1.1 Hz, 1H), 3.83 (s, 3H), 2.41 (s, 3H).

^{13}C NMR (151 MHz, $CDCl_3$) major isomer: δ 178.3, 164.3, 161.6, 150.1, 133.8, 132.9, 131.3, 129.9, 128.5, 128.3, 127.9, 127.5, 124.9, 121.0, 113.3, 108.4, 97.0, 91.6, 55.8, 19.0.

IR (ν_{max}, cm^{-1}) 2920(w), 2176(s), 1740(s), 1608(s), 1501 (s), 1402 (s), 1286(s), 1262(m), 1141 (m), 1101(s), 1024(m), 968(m), 756(s), 691(s).

HRMS (nanochip-ESI/LTQ-Orbitrap) m/z: $[M + H]^+$ Calcd for $C_{22}H_{20}NO_3^+$ 346.1438; Found 346.1436.

5-methyl-2-((1*E*)-3-((phenylethynyl)imino)but-1-en-1-yl)phenyl acrylate (15d)

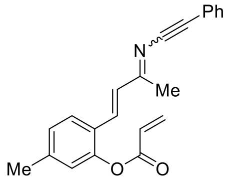

Compound **15d**: **Yield**: 20% (65.8 mg), yellow oil as a 4:1 mixture of isomers, purified by column chromatography on silica gel (petroleum ether/ethyl acetate = 90/1).

^{1}H NMR (400 MHz, $CDCl_3$) major isomer: δ 7.62-7.57 (m, 1H), 7.46-7.43(m, 2H), 7.36-7.28 (m, 4H), 7.12-7.09 (m, 1H), 7.00 (brs, 1H), 6.93 (d, *J* = 16.4 Hz, 1H), 6.69 (dd, *J* = 17.4, 1.2 Hz, 1H), 6.41 (dd, *J* = 17.3, 10.5 Hz, 1H), 6.10 (dd, *J* = 10.5, 1.2 Hz, 1H), 2.43 (s, 3H), 2.39 (s, 3H).

^{13}C NMR (101 MHz, $CDCl_3$) major isomer: δ 178.3, 164.5, 148.9, 141.6, 133.5, 133.0, 131.4, 131.3, 128.5, 127.9, 127.7, 127.6, 127.2, 125.5, 124.9, 123.6, 97.7, 91.5, 21.5, 19.1.

IR (ν_{max}, cm^{-1}) 2923(w), 1740(s), 1615(s), 1401 (s), 1231 (m), 1146 (m), 1101(s), 1024(s), 971(m), 756(s), 691(s).

HRMS (ESI/QTOF) m/z: $[M + H]^+$ Calcd for $C_{22}H_{20}NO_2^+$ 330.1489; Found 330.1483.

3. Condition optimization of the Diels-Alder reaction

Condition optimization for the reaction of the β-aryl substituted α,β-unsaturated ynimines

Table S1: Solvent screening

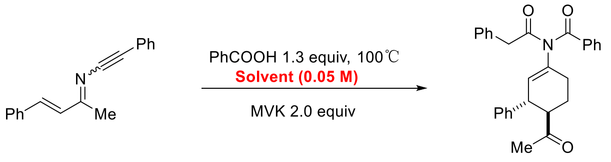

4a **7a**

Entry	Solvent	Yield (%)
1	PhCl	33
2	$PhCF_3$	17
3	$PhCH_3$	60
**4**	**DCE**	**66**
5	CH_3CN	21
6	Dioxane	9
7	DMF	0

[a]Reaction conditions: **4a** (0.05 mmol), PhCOOH (1.3 equiv), methyl vinyl ketone (MVK, 3.0 equiv), solvent (1.0 mL), RT, then 100 °C, 24 h. [b]Yield was determined by ^1H NMR spectroscopy with CH_2Br_2 as an internal standard.

Table S2: Temperature screening

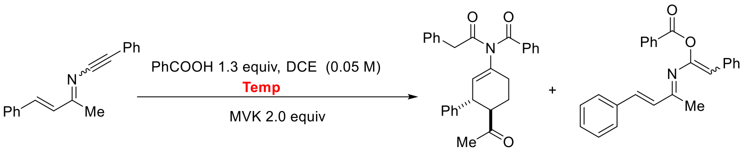

4a **7a** **11a**

Entry	Temp (°C)	Yield (%)
1	140	decomposed
2	120	58
3	80	44
4	60	12
5	RT	**11a** (95%)

[a]Reaction conditions: **4a** (0.05 mmol), PhCOOH (1.3 equiv), methyl vinyl ketone (MVK, 3.0 equiv), DCE (1.0 mL), RT, then 100 °C, 24 h. [b]Yield was determined by ^1H NMR spectroscopy with CH_2Br_2 as an internal standard.

Condition optimization for the reaction of the β-alkyl substituted α,β-unsaturated ynimines

Table S3: Temperature screening

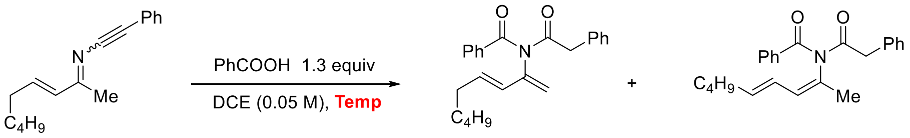

4r **6r** **14r**

Entry	Temp (℃)	Yield (%)
1	140	decomposed
2	60 (4 day)	**6r** and **14r** (41 : 46)
3	rt	No **6r** and **14r**
4	0	No reaction

[a]Reaction conditions: **4r** (0.05 mmol), PhCOOH (1.3 equiv), DCE (1.0 mL), 24 h. [b]Yield was determined by ^{1}H NMR spectroscopy with CH_2Br_2 as an internal standard.

Table S4: Base screening

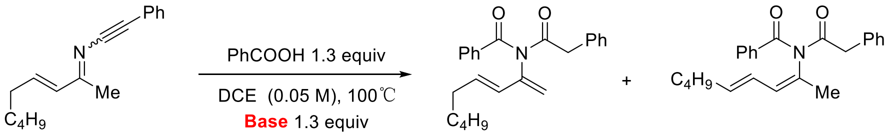

4r **6r** **14r**

Entry	Base	Yield (%)
1	Et_3N	**6r** and **14r** (40 : 25)
2	Pyridine	Decomposed
3	DIPEA	**6r** and **14r** (17 : 30)
4	DIPA	**6r** and **14r** (72 : 16)
5	Diethylamine	Decomposed
6	Piperazine	Decomposed
**7**	**2,2,6,6-Tetramethylpiperidine (TEMP)**	**6r and 14r (83 : 8)**
8	Imidazole	Decomposed
9	Pyrrole	**6r** and **14r** (21 : 31)
10	Piperidine	Decomposed
11	4-Phenylpiperidine	Decomposed
12	Morpholine	**6r** and **14r** (10 : 8)
13	2,4,6-Collidine	**6r** and **14r** (42 : 32)

[a]Reaction conditions: **4r** (0.05 mmol), PhCOOH (1.3 equiv), DCE (1.0 mL), 24 h, Base (1.3 equiv), RT, then 100 °C. [b]Yield was determined by ^{1}H NMR spectroscopy with CH_2Br_2 as an internal standard.

Condition optimization of the catalytic enantioselective Diels-Alder reaction

Table S5: Catalyst screening

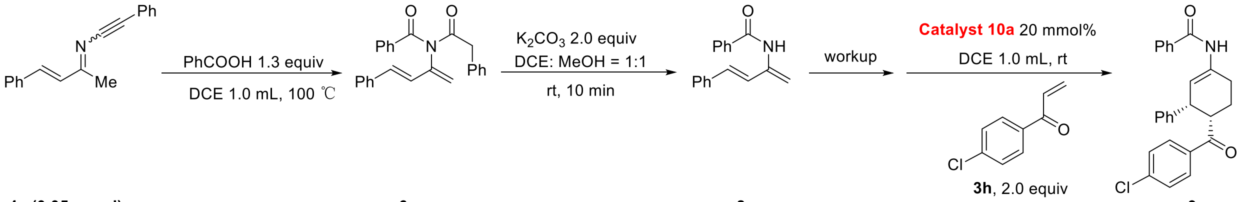

4a (0.05 mmol) **6a** **8a** **9a**

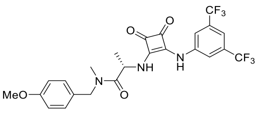
56%, 68% *ee*
10a

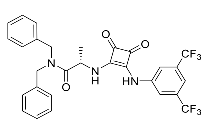
53%, 58% *ee*
10b

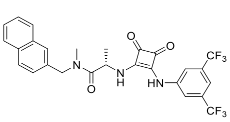
58%, 67% *ee*
10c

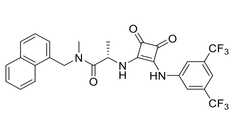
61%, 59% *ee*
10d

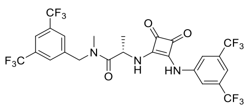
48%, 67% *ee*
10e

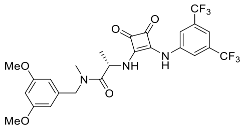
51%, 61% *ee*
10f

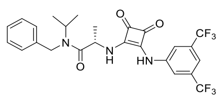
64%, 62% *ee*
10g

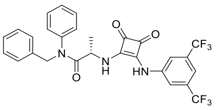
56%, 5% *ee*
10h

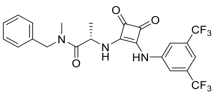
44%, 63% *ee*
10i

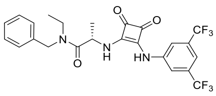
55%, 56% *ee*
10j

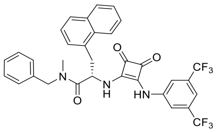
65%, -40% *ee*
10k

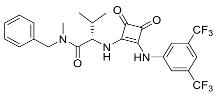
51%, 53% *ee*
10l

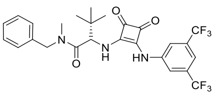
55%, 53% *ee*
10m

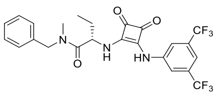
54%, 59% *ee*
10n

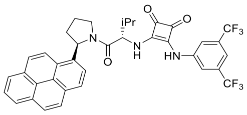
51%, 0% *ee*
10o

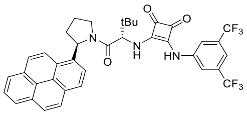
47%, 0% *ee*
10p

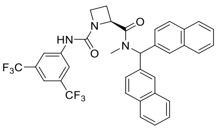
36%, 23% *ee*
10q

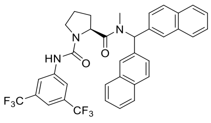
56%, 0% *ee*
10r

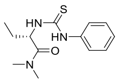
71%, 40% *ee*
10s

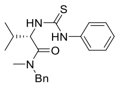
62%, 24% *ee*
10t

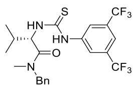
54%, 32% *ee*
10u

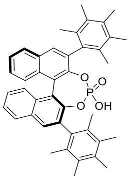
No product, decomposed
10v

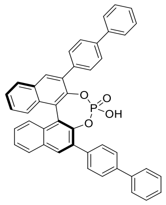
No product, decomposed
10w

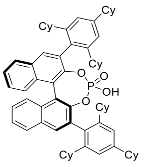
No product, decomposed
10x

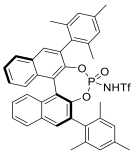
55%, 0% ee
10y

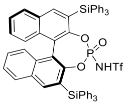
60%, 0% *ee*
1z

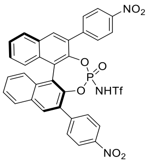
No product, decomposed
10aa

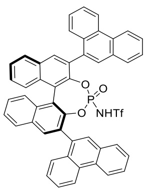
No product, decomposed
10ab

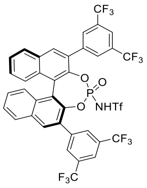
No product, decomposed
10ac

Table S6: Temperature screening

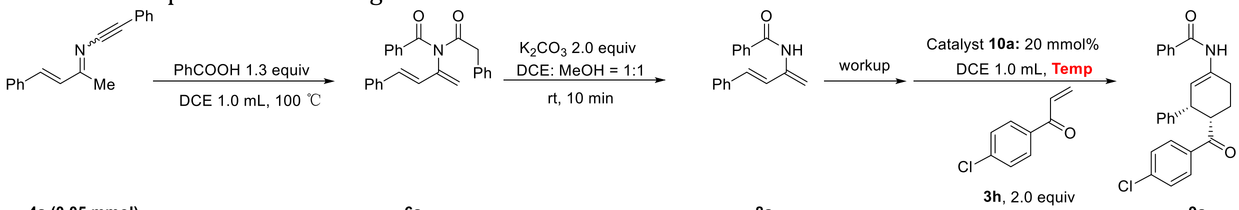

4a (0.05 mmol) **6a** **8a** **9a**

Entry	Temp (℃)	Ee (%)
1	60	51 (d:r = 10:1)
2	0	80 (d:r >20:1)

**3**	**-20**	**93 (d:r > 20:1)**
4	-40	90 (d:r > 20:1)

Table S7: Screening of catalyst loading.

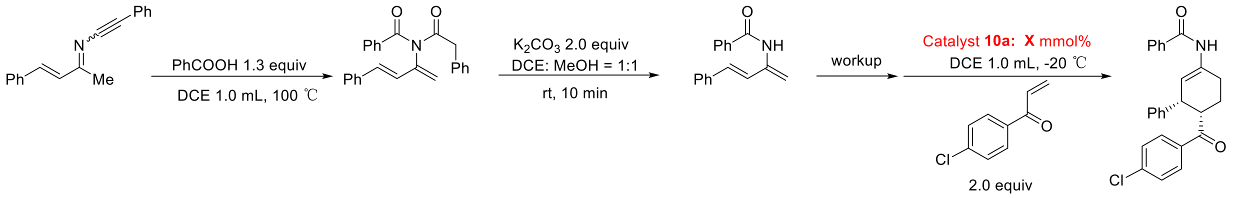

4a (0.05 mmol) **6a** **8a** **9a**

Entry	Loading of catalyst (%)	Ee (%)
1	5	79 (d:r > 20:1)
2	10	82 (d:r >20:1)
3	15	88 (d:r >20:1)
4	30	93 (d:r > 20:1)

4. Synthesis and characterization data of the Diels-Alder reaction products

Figure S5: Synthesis of compounds **7a-7q**

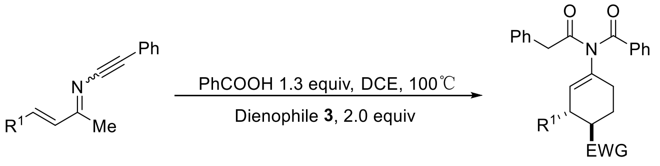

4
R^1 = aryl

7

A mixture of ynimine **4** (0.10 mmol, 1.0 equiv), benzoic acid (15.9 mg, 0.13 mmol, 1.3 equiv) and dienophile **3** (0.20 mmol, 2.0 equiv) in DCE (2.0 mL, 0.05 M) was stirred at room temperature until complete consumption of **4**, followed by heating the reaction mixture to reflux until the diene was completely consumed. The reaction was then quenched with water and extracted with DCM. The combined organic phases were washed with water and brine, dried over $MgSO_4$, filtered, and concentrated under vacuum. The residue was purified by flash chromatography or preparative TLC to give the desired product **7**.

Figure S6: Synthesis of compounds **7r-7y**

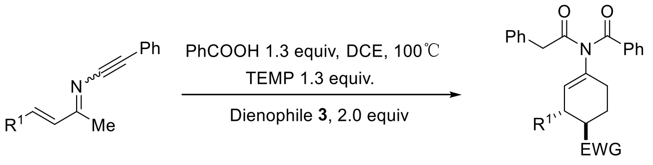

4
R^1 = alkyl

7

A mixture of ynimine **4** (0.10 mmol, 1.0 equiv), 2,2,6,6-tetramethylpiperidine (TEMP) (18.4 mg, 0.13 mmol, 1.3 equiv), dienophile **3** and benzoic acid (15.9 mg, 0.13 mmol, 1.3 equiv) in DCE (2.0 mL, 0.05 M) was stirred at room temperature until complete consumption of **4**, followed by heating the reaction mixture to reflux until

the diene was completely consumed. The reaction mixture was then quenched with water and extracted with DCM. Then the combined organic phases were washed with water and brine, dried over $MgSO_4$, filtered, and concentrated under vacuum. The residue was purified by flash chromatography or preparative TLC to give the desired product **7**.

Figure S7: Synthesis of compounds **9a-9h**

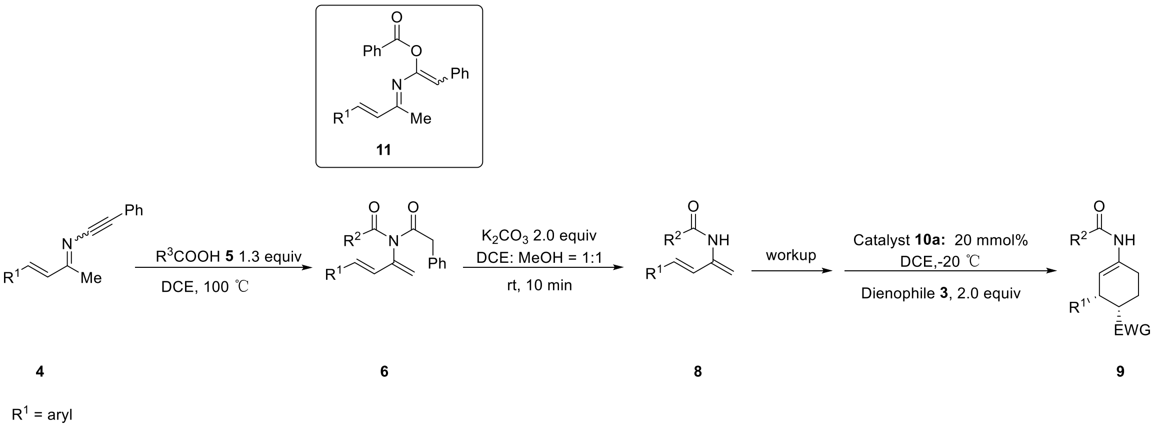

A mixture of ynimine **4** (0.10 mmol, 1.0 equiv) and carboxylic acid **5** (0.13 mmol, 1.3 equiv) in DCE (2.0 mL, 0.05 M) was stirred at 100 °C until no more α-alkylideneimino enol ester **11** detected by TLC. The reaction mixture was cooled to room temperature and K_2CO_3 (27.6 mg, 0.20 mmol, 2.0 equiv) and methanol (2.0 mL) were added. The reaction mixture was stirred at room temperature for 10 min, then quenched with water and extracted with DCM. The combined organic phases were washed with water and brine, dried over $MgSO_4$, filtered, and concentrated under vacuum. The residue was dissolved in DCE, followed by addition of catalyst **10a** (10.6 mg, 0.02 mmol, 20 mol%) and dienophile **3** (0.20 mmol, 2.0 equiv). The resulting solution was stirred at -20°C until the diene was completely consumed. The reaction mixture was quenched with water and extracted with DCM. The combined organic phases were washed with water and brine, dried over $MgSO_4$, filtered, and concentrated under vacuum. The residue was purified by flash chromatography or preparative TLC to give the desired product **9**.

Figure S8: Synthesis of compound **9i**

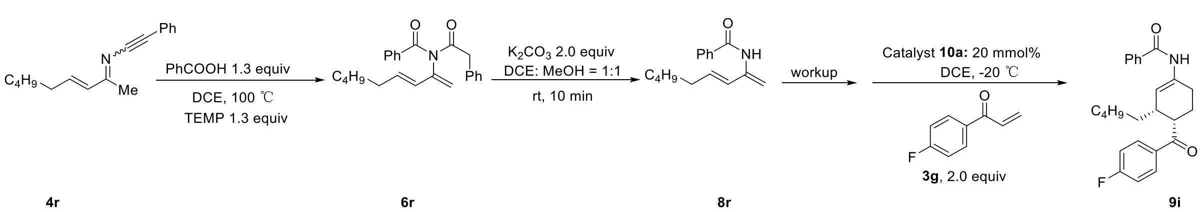

A mixture of ynimine **4r** (23.9mg, 0.10 mmol, 1.0 equiv), 2,2,6,6-tetramethylpiperidine (TEMP) (18.4 mg, 0.13 mmol, 1.3 equiv) and benzoic acid (15.9 mg, 0.13 mmol, 1.3 equiv) in DCE (2.0 mL, 0.05 M) was stirred at 100 °C until no more α-alkylideneimino enol ester **11** detected by TLC. After cooling to room temperature, K_2CO_3 (27.6 mg, 0.20 mmol, 2.0 equiv) and methanol (2.0 mL) were added and the reaction mixture was stirred at room temperature for 10 min. The reaction mixture was quenched with water and extracted with DCM then the combined organic phases were washed with water and brine, dried over $MgSO_4$, filtered, and concentrated under vacuum. The residue was dissolved in DCE, followed by addition of catalyst **10a** (10.6 mg, 0.02 mmol, 20 mol%) and dienophile **3g** (30.0mg, 0.20 mmol, 2.0 equiv). The resulting solution was stirred at -20 °C until the diene was completely consumed. The reaction mixture was quenched with water and extracted with DCM then

the combined organic phases were washed with water and brine, dried over $MgSO_4$, filtered and concentrated under vacuum. The residue was purified by preparative TLC to give the desired product **9i**.

Figure S9: Synthesis of compounds **16a-16d**

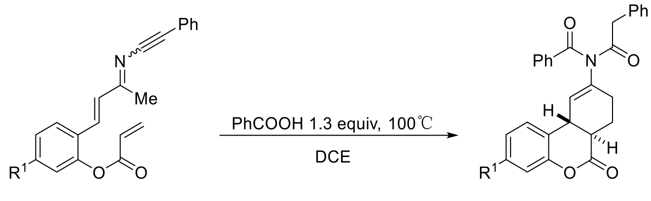

A mixture of ynimine **15** (0.10 mmol, 1.0 equiv) and benzoic acid (15.9 mg, 0.13 mmol, 1.3 equiv) in DCE (2.0 mL, 0.05 M) was stirred at 100 °C until the ynimine was completely consumed. The reaction mixture was quenched with water and extracted with DCM. The combined organic phases were washed with water and brine, dried over $MgSO_4$, filtered, and concentrated under vacuum. The residue was purified by flash chromatography or preparative TLC to give the desired product **16**.

(*E*)-N-(2-phenylacetyl)-N-(4-phenylbuta-1,3-dien-2-yl)benzamide (6a)

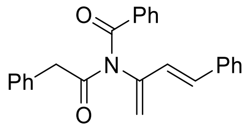

Yield: 90% (33.0 mg), pale oil as a single isomer, purified by column chromatography on silica gel (petroleum ether/ethyl acetate = 20/1).

^{1}H NMR (400 MHz, CD_3CN) δ 7.65 (dd, *J* = 8.0, 1.5 Hz, 2H), 7.52 (td, *J* = 7.1, 1.8 Hz, 1H), 7.44 – 7.26 (m, 12H), 6.90 (d, *J* = 16.1 Hz, 1H), 6.60 (d, *J* = 16.2 Hz, 1H), 5.57 (s, 1H), 5.23 (s, 1H), 4.12 (s, 2H).

^{13}C NMR (101 MHz, CD_3CN) δ 175.6, 173.8, 144.9, 137.0, 136.3, 136.0, 133.0, 131.7, 130.8, 129.8, 129.45, 129.43, 129.3, 129.1, 128.1, 127.8, 126.6, 121.0, 44.1.

IR ν (cm^{-1}) 1723 (m), 1718 (m), 1714 (m), 1690 (s), 1685 (s), 1680 (s), 1449 (m), 1273 (m), 1259 (m), 1247 (m), 1241 (m), 1128 (m), 1118 (w), 1073 (w), 1026 (m), 707 (s), 694 (s), 684 (m), 669 (m), 665 (w).

HRMS (ESI/QTOF) m/z: [M + Na]+ Calcd for $C_{25}H_{21}NNaO_2^+$ 390.1464; Found 390.1471.

(E)-N-(nona-1,3-dien-2-yl)-N-(2-phenylacetyl)benzamide (6r)

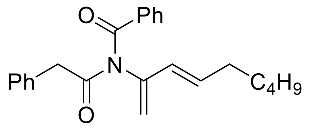

Yield: 41% (14.4 mg), pale oil as a single isomer, purified by column chromatography on silica gel (petroleum ether/ethyl acetate = 10/1).

^{1}H NMR (500 MHz, $CDCl_3$) δ 7.53-7.50 (m, 2H), 7.48-7.44 (m, 1H), 7.34-7.26 (m, 7H), 6.03 (dt, *J* = 15.6, 1.5 Hz, 1H), 5.65 (dt, *J* = 15.6, 7.0 Hz, 1H), 5.26 (s, 1H), 4.94 (s, 1H), 4.06 (s, 2H), 2.07 (qd, *J* = 7.2, 1.5 Hz, 2H), 1.38-1.19 (m, 6H), 0.89 (t, *J* = 7.2 Hz, 3H).

^{13}C NMR (126 MHz, $CDCl_3$) δ 174.7, 173.2, 143.7, 135.2, 134.7, 134.6, 132.2, 129.8, 128.8, 128.32, 128.31, 127.5, 126.8, 117.5, 43.8, 32.5, 31.5, 28.7, 22.7, 14.2.

IR ν (cm^{-1}) 2926 (w), 1707 (w), 1679 (w), 1280 (w), 913 (s), 743 (s).

HRMS (ESI/QTOF) m/z: $[M + Na]^+$ Calcd for $C_{24}H_{27}NNaO_2^+$ 384.1934; Found 384.1934.

N-6-acetyl-1,4,5,6-tetrahydro-[1,1'-biphenyl]-3-yl)-N-(2-phenylacetyl)benzamide (7a)

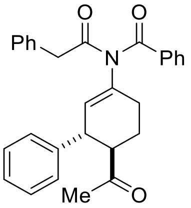

Yield: 66% (28.9 mg), pale oil as a single diastereoisomer, purified by column chromatography on silica gel (petroleum ether/ethyl acetate = 6/1).

^{1}H NMR (400 MHz, $CDCl_3$) δ 7.59-7.54 (m, 1H), 7.44-7.40 (m, 4H), 7.32-7.28 (m, 4H), 7.27-7.24 (m, 1H), 7.14-7.07 (m, 3H), 6.62-6.58 (m, 2H), 5.40 (dt, *J* = 2.9, 1.5 Hz, 1H), 4.16 (d, *J* = 15.1 Hz, 1H), 4.07 (d, *J* = 15.1 Hz, 1H), 3.70 (dq, *J* = 8.7, 2.9 Hz, 1H), 2.48 (ddd, *J* = 11.7, 8.8, 3.1 Hz, 1H), 2.34-2.28 (m, 2H), 1.96-1.89 (m, 1H), 1.86 (s, 3H), 1.82-1.73 (m, 1H).

^{13}C NMR (101 MHz, $CDCl_3$) δ 210.3, 174.9, 173.2, 142.8, 137.1, 135.9, 134.7, 132.2, 132.0, 129.7, 128.8, 128.76, 128.7, 128.1, 128.0, 127.4, 127.0, 54.9, 44.1, 43.8, 30.0, 27.3, 25.1.

IR ν (cm^{-1}) 2930(w), 1687(m), 1599(s), 1492(s), 1450(s), 1276(m), 1132(s), 911(s), 760(s), 722(m), 700(m), 670(s).

HRMS (ESI/QTOF) m/z: $[M + Na]^+$ Calcd for $C_{29}H_{27}NNaO_3^+$ 460.1883; Found 460.1881.

N-6-acetyl-4'-methoxy-1,4,5,6-tetrahydro-[1,1'-biphenyl]-3-yl)-N-(2-phenylacetyl)benzamide (7b)

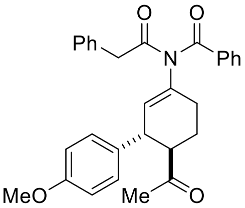

Yield: 59% (27.5 mg), pale oil as a single diastereoisomer, purified by column chromatography on silica gel (petroleum ether/ethyl acetate = 6/1).

^{1}H NMR (400 MHz, $CDCl_3$) 7.57-7.53 (m, 1H), 7.44-7.39 (m, 4H), 7.31-7.27 (m, 4H), 7.26-7.22 (m, 1H), 6.62 (d, *J* = 8.5 Hz, 2H), 6.50 (d, *J* = 8.5 Hz, 2H), 5.36 (dt, *J* = 2.9, 1.5 Hz, 1H), 4.15 (d, *J* = 15.0 Hz, 1H), 4.05 (d, *J* = 15.1 Hz, 1H), 3.72 (s, 3H), 3.64 (dq, *J* = 8.9, 2.9 Hz, 1H), 2.43 (ddd, *J* = 11.7, 8.9, 3.0 Hz, 1H), 2.32-2.26 (m, 2H), 1.93-1.87 (m, 1H), 1.85 (s, 3H), 1.79-1.71 (m, 1H).

^{13}C NMR (101 MHz, $CDCl_3$) δ 210.5, 174.9, 173.2, 158.5, 136.9, 135.8, 134.8, 134.7, 132.4, 132.2, 129.6, 129.0, 128.8, 128.7, 128.1, 127.3, 114.0, 55.3, 55.0, 44.0, 43.0, 30.0, 27.2, 25.1.

IR ν (cm^{-1}) 1687 (s), 1608 (w), 1510 (m), 1450 (m), 1356 (m), 1277 (s), 1244 (s), 1176 (m), 1134 (m), 1032 (m), 908 (m), 833 (m), 721 (s), 700 (s), 667 (m).

HRMS (ESI/QTOF) m/z: $[M + Na]^+$ Calcd for $C_{30}H_{29}NNaO_4^+$ 490.1989; Found 490.1985.

N-6-acetyl-4'-methyl-1,4,5,6-tetrahydro-[1,1'-biphenyl]-3-yl)-N-(2-phenylacetyl)benzamide (7c)

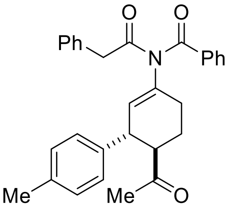

Yield: 60% (27 mg), pale oil as a single diastereoisomer, purified by column chromatography on silica gel (petroleum ether/ethyl acetate = 6/1).

^{1}H NMR (600 MHz, $CDCl_3$) δ 7.59-7.55 (m, 1H), 7.44-7.41 (m, 4H), 7.33-7.29 (m, 4H), 7.27-7.24 (m, 1H), 6.90 (d, *J* = 7.8 Hz, 2H), 6.46 (d, *J* = 8.1 Hz, 2H), 5.38 (dt, *J* = 2.8, 1.5 Hz, 1H), 4.16 (d, *J* = 15.1 Hz, 1H), 4.07 (d, *J* = 15.1 Hz, 1H), 3.66 (dq, *J* = 8.8, 2.9 Hz, 1H), 2.45 (ddd, *J* = 11.6, 8.9, 3.0 Hz, 1H), 2.32-2.29 (m, 2H), 2.25 (s, 3H), 1.94-1.90 (m, 1H), 1.86 (s, 3H), 1.81-1.75 (m, 1H).

^{13}C NMR (151 MHz, $CDCl_3$) δ 210.5, 174.9, 173.2, 139.7, 137.0, 136.5, 135.8, 134.7, 132.3, 132.2, 129.7, 129.4, 128.8, 128.7, 128.1, 127.9, 127.34, 55.0, 44.1, 43.4, 30.0, 27.3, 25.1, 21.1.

IR (ν_{max}, cm^{-1}) 2922(w), 1688(m), 1511(s), 1450(s), 1450(s), 1277(w), 1134(s), 911(s), 815(s), 721(m), 670(s), 613(s).

HRMS (ESI/QTOF) m/z: [M + Na]$^+$ Calcd for $C_{30}H_{29}NNaO_3^+$ 474.2040; Found 474.2044.

N-6-acetyl-4'-fluoro-1,4,5,6-tetrahydro-[1,1'-biphenyl]-3-yl)-N-(2-phenylacetyl)benzamide (7d)

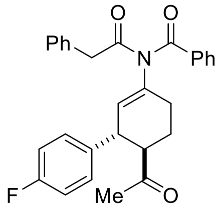

Yield: 66% (30 mg), pale oil as a single diastereoisomer, purified by column chromatography on silica gel (petroleum ether/ethyl acetate = 6/1).

^{1}H NMR (600 MHz, $CDCl_3$) δ 7.59-7.55 (m, 1H), 7.43-7.41 (m, 4H), 7.32-7.28 (m, 4H), 7.27-7.25 (m, 1H), 6.79-6.75 (m, 2H), 6.54-6.50 (m, 2H), 5.34 (dt, *J* = 2.9, 1.5 Hz, 1H), 4.16 (d, *J* = 15.1 Hz, 1H), 4.06 (d, *J* = 15.1 Hz, 1H), 3.71 (dq, *J* = 8.8, 2.9 Hz, 1H), 2.41 (ddd, *J* = 11.6, 8.9, 3.0 Hz, 1H), 2.35-2.25 (m, 2H), 1.96-1.91 (m, 1H), 1.88 (s, 3H), 1.78-1.71 (m, 1H).

^{13}C NMR (151 MHz, $CDCl_3$) δ 210.0, 175.0, 173.1, 161.75 (d, *J* = 245.5 Hz), 138.54 (d, *J* = 3.5 Hz), 137.3, 135.8, 134.6, 132.3, 131.8, 129.7, 129.55 (d, *J* = 7.9 Hz), 128.8, 128.8, 128.1, 127.4, 115.51 (d, *J* = 21.2 Hz), 55.1, 44.1, 42.8, 29.9, 27.3, 25.2.

^{19}F NMR (565 MHz, $CDCl_3$) δ -115.8.

IR (ν_{max}, cm^{-1}) 2934(w), 1685(m), 1601(s), 1507(s), 1449(s), 1275(w), 1220(w), 1158(s), 1132(s), 911(s), 836(s), 721(m), 696(m), 664(s).

HRMS (ESI/QTOF) m/z: [M + Na]$^+$ Calcd for $C_{29}H_{26}FNNaO_3^+$ 478.1789; Found 478.1799.

N-6-acetyl-4'-chloro-1,4,5,6-tetrahydro-[1,1'-biphenyl]-3-yl)-N-(2-phenylacetyl)benzamide (7e)

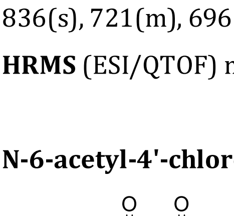

Yield: 64% (30.1 mg), pale oil as a single diastereoisomer, purified by column chromatography on silica gel (petroleum ether/ethyl acetate = 6/1).

^{1}H NMR (500 MHz, $CDCl_3$) δ 7.60-7.55 (m, 1H), 7.45-7.39 (m, 4H), 7.32-7.24 (m, 5H), 7.04 (d, *J* = 8.3 Hz, 2H), 6.48 (d, *J* = 8.3 Hz, 2H), 5.32 (t, *J* = 2.3 Hz, 1H), 4.17 (d, *J* = 15.1 Hz, 1H), 4.06 (d, *J* = 15.2 Hz, 1H), 3.71 (dq, *J* = 9.0, 3.0 Hz, 1H), 2.39 (ddd, *J* = 11.6, 8.8, 3.0 Hz, 1H), 2.35-2.26 (m, 2H), 1.97-1.91 (m, 1H), 1.89 (s, 3H), 1.79-1.71 (m, 1H).

^{13}C NMR (126 MHz, $CDCl_3$) δ 209.8, 175.0, 173.1, 141.4, 137.5, 135.8, 134.6, 132.7, 132.3, 131.5, 129.7, 129.4, 128.8, 128.8, 128.1, 127.4, 55.0, 44.1, 42.9, 29.9, 27.3, 25.2.

IR (ν_{max}, cm^{-1}) 2927(w), 1685(m), 1491(s), 1448(s), 1275(m), 1246(m), 1014(s), 911(s), 830(s), 721(m), 695(m), 668(s).

HRMS (nanochip-ESI/LTQ-Orbitrap) m/z: $[M + H]^+$ Calcd for $C_{29}H_{27}ClNO_3^+$ 472.1674; Found 472.1671.

N-6-acetyl-4'-bromo-1,4,5,6-tetrahydro-[1,1'-biphenyl]-3-yl)-N-(2-phenylacetyl)benzamide (7f)

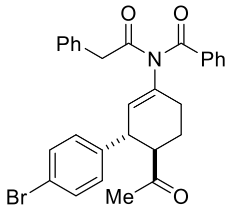

Yield: 50% (25.8 mg), pale oil as a single diastereoisomer, purified by column chromatography on silica gel (petroleum ether/ethyl acetate = 6/1).

^{1}H NMR (600 MHz, $CDCl_3$) δ 7.60-7.56 (m, 1H), 7.44-7.40 (m, 4H), 7.32-7.28 (m, 4H), 7.27-7.25 (m, 1H), 7.20-7.17 (m, 2H), 6.42-6.39 (m, 2H), 5.32 (qd, J = 1.8, 1.0 Hz, 1H), 4.17 (d, J = 15.1 Hz, 1H), 4.06 (d, J = 15.2 Hz, 1H), 3.70 (dq, J = 8.8, 2.8 Hz, 1H), 2.39 (ddd, J = 11.7, 8.9, 3.0 Hz, 1H), 2.35-2.26 (m, 2H), 1.96-1.91 (m, 1H), 1.89 (s, 3H), 1.78-1.71 (m, 1H).

^{13}C NMR (151 MHz, $CDCl_3$) δ 209.7, 175.0, 173.1, 141.9, 137.6, 135.8, 134.6, 132.3, 131.8, 131.4, 129.8, 129.7, 128.8, 128.8, 128.1, 127.4, 120.8, 54.9, 44.1, 42.9, 29.8, 27.3, 25.2.

IR ν (cm^{-1}) 2944(w), 1687(m), 1488(s), 1449(s), 1274(m), 1132(s), 1073(s), 1010(s), 725(m), 712(s).

HRMS (ESI/QTOF) m/z: $[M + Na]^+$ Calcd for $C_{29}H_{26}BrNNaO_3^+$ 538.0988; Found 538.0995.

N-6-acetyl-3'-methyl-1,4,5,6-tetrahydro-[1,1'-biphenyl]-3-yl)-N-(2-phenylacetyl)benzamide (7g)

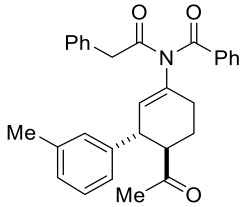

Yield: 55% (24.8 mg), pale oil as a single diastereoisomer, purified by column chromatography on silica gel (petroleum ether/ethyl acetate = 6/1).

^{1}H NMR (600 MHz, $CDCl_3$) δ 7.58-7.53 (m, 1H), 7.44-7.39 (m, 4H), 7.32-7.29 (m, 4H), 7.27-7.25 (m, 1H), 6.98 (t, J = 7.5 Hz, 1H), 6.94 (d, J = 7.5 Hz, 1H), 6.56 (s, 1H), 6.34 (d, J = 7.4 Hz, 1H), 5.40 (brs, 1H), 4.14 (d, J = 15.1 Hz, 1H), 4.06 (d, J = 15.1 Hz, 1H), 3.65 (dq, J = 8.8, 2.9 Hz, 1H), 2.49 (td, J = 8.9, 4.6 Hz, 1H), 2.33-2.30 (m, 2H), 2.21 (s, 3H), 1.95-1.91 (m, 1H), 1.86 (s, 3H), 1.82-1.75 (m, 1H).

^{13}C NMR (151 MHz, $CDCl_3$) δ 210.5, 174.9, 173.2, 142.6, 138.3, 136.9, 135.7, 134.7, 132.3, 132.1, 129.7, 128.8, 128.7, 128.65, 128.1, 127.8, 127.4, 125.0, 54.7, 44.0, 43.9, 30.0, 27.3, 25.2, 21.5.

IR (ν_{max}, cm^{-1}) 3026(w), 1686(m), 1602(s), 1492(s), 1449(s), 1276(m), 1132(s), 786(s), 721(s), 703(s).

HRMS (ESI/QTOF) m/z: $[M + Na]^+$ Calcd for $C_{30}H_{29}NNaO_3^+$ 474.2040; Found 474.2044.

N-6-acetyl-3'-chloro-1,4,5,6-tetrahydro-[1,1'-biphenyl]-3-yl)-N-(2-phenylacetyl)benzamide (7h)

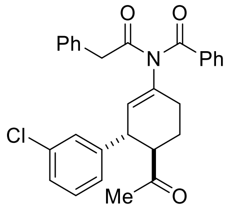

Yield: 49% (23.0 mg), pale oil as a single diastereoisomer, purified by column chromatography on silica gel (petroleum ether/ethyl acetate = 6/1).

^{1}H NMR (400 MHz, $CDCl_3$) δ 7.60-7.54 (m, 1H), 7.45-7.39 (m, 4H), 7.31-7.25 (m, 5H), 7.13-7.09 (m, 1H), 7.03 (t, *J* = 7.8 Hz, 1H), 6.63 (s, 1H), 6.52 (d, *J* = 7.6 Hz, 1H), 5.35 (brs, 1H), 4.16 (d, *J* = 15.1 Hz, 1H), 4.06 (d, *J* = 15.1 Hz, 1H), 3.72 (dq, *J* = 9.0, 2.9 Hz, 1H), 2.45 (ddd, *J* = 11.7, 8.9, 3.0 Hz, 1H), 2.35-2.26 (m, 2H), 1.98-1.87 (m, 1H), 1.89 (s, 3H), 1.79-1.72 (m, 1H).

^{13}C NMR (101 MHz, $CDCl_3$) δ 209.6, 174.9, 173.1, 144.8, 137.6, 135.6, 134.6, 134.5, 132.6, 131.1, 130., 129.7, 128.8, 128.0, 127.9, 127.4, 127.3, 126.5, 54.6, 44.1, 43.32, 29.8, 27.3, 25.2.

IR ν (cm^{-1}) 1689 (s), 1597 (w), 1574 (w), 1450 (w), 1427 (w), 1360 (m), 1275 (s), 1254 (m), 1159 (m), 1132 (m), 1080 (m), 910 (m), 787 (m), 729 (s), 696 (s), 667 (m).

HRMS (ESI/QTOF) m/z: $[M + Na]^+$ Calcd for $C_{29}H_{26}ClNNaO_3^+$ 494.1493; Found 494.1503.

N-6-acetyl-2'-methyl-1,4,5,6-tetrahydro-[1,1'-biphenyl]-3-yl)-N-(2-phenylacetyl)benzamide (7i)

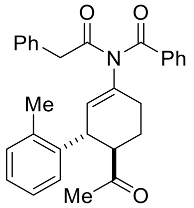

Yield: 70% (31.6 mg), pale oil as 11:1 mixture of diastereoisomers, purified by column chromatography on silica gel (petroleum ether/ethyl acetate = 6/1).

^{1}H NMR (400 MHz, $CDCl_3$) δ7.58-7.54 (m, 1H), 7.41-7.38 (m, 4H), 7.33-7.29 (m, 4H), 7.27-7.25 (m, 1H), 7.06-7.01 (m, 2H), 6.91-6.87 (m, 1H), 6.29 (d, *J* = 7.7 Hz, 1H), 5.34 (dt, *J* = 3.1, 1.5 Hz, 1H), 4.14 (d, *J* = 15.0 Hz, 1H), 4.05 (d, *J* = 15.0 Hz, 1H), 3.96 (dq, *J* = 8.1, 2.8 Hz, 1H), 2.55 (ddd, *J* = 9.1, 7.4, 3.9 Hz, 1H), 2.31-2.27 (m, 2H), 2.21 (s, 3H), 1.94-1.84 (m, 2H), 1.88 (s, 3H).

^{13}C NMR (101 MHz, $CDCl_3$) δ 210.3, 174.9, 173.3, 140.8, 136.8, 135.9, 135.7, 134.7, 132.2, 132.1, 130.7, 129.7, 128.8, 128.7, 128.05, 128.0, 127.4, 126.8, 126.5, 53.3, 44.0, 39.2, 29.7, 26.8, 24.2, 19.6.

IR (ν_{max}, cm^{-1}) 2930(w), 1689(m), 1602(s), 1490(s), 1450(s), 1278(m), 1133(s), 913(s), 758(m), 723(m), 696(m).

HRMS (ESI/QTOF) m/z: $[M + Na]^+$ Calcd for $C_{30}H_{29}NNaO_3^+$ 474.2040; Found 474.2043.

N-4-acetyl-3-(benzo[d][1,3]dioxol-5-yl)cyclohex-1-en-1-yl)-N-(2-phenylacetyl)benzamide (7j)

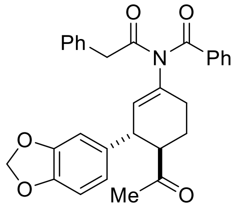

Yield: 54% (25.8 mg), pale oil as a single diastereoisomer, purified by column chromatography on silica gel (petroleum ether/ethyl acetate = 4/1).

^{1}H NMR (400 MHz, $CDCl_3$) δ 7.58-7.52 (m, 1H), 7.42-7.40 (m, 4H), 7.32-7.27 (m, 4H), 7.27-7.25 (m, 1H), 6.53 (d, *J* = 8.0 Hz, 1H), 6.09 (dd, *J* = 7.9, 1.8 Hz, 1H), 5.97 (d, *J* = 1.8 Hz, 1H), 5.88 (d, *J* = 1.5 Hz, 1H), 5.87 (d, *J* = 1.5 Hz, 1H), 5.35-5.32 (m, 1H), 4.16 (d, *J* = 15.1 Hz, 1H), 4.06 (d, *J* = 15.0 Hz, 1H), 3.62 (dq, *J* = 8.9, 2.9 Hz, 1H), 2.41 (ddd, *J* = 11.8, 9.0, 3.0 Hz, 1H), 2.31-2.21 (m, 2H), 1.95-1.86 (m, 1H), 1.88 (s, 3H), 1.80-1.70 (m, 1H).

^{13}C NMR (101 MHz, $CDCl_3$) δ 210.3, 174.9, 173.2, 147.8, 146.5, 137.1, 136.5, 135.8, 134.7, 132.4, 132.2, 129.7, 128.82, 128.79, 128.0, 127.4, 121.2, 108.3, 108.3, 101.1, 55.0, 44.1, 43.6, 30.0, 27.3, 25.2.

IR ν (cm^{-1}) 2896(w), 1687(m), 1485(s), 1440(s), 1243(m), 1130(s), 1037(s), 933(m), 724(m).

HRMS (ESI/QTOF) m/z: $[M + Na]^+$ Calcd for $C_{30}H_{27}NNaO_5^+$ 504.1781; Found 504.1793.

2'-acetyl-4-methoxy-5'-(N-(2-phenylacetyl)benzamido)-1',2',3',4'-tetrahydro-[1,1'-biphenyl]-2-yl acetate (7k)

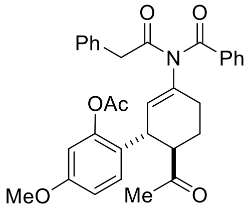

Yield: 49% (22.0 mg), pale oil as a single diastereoisomer, purified by column chromatography on silica gel (petroleum ether/ethyl acetate = 3/1).

The compound of **7k** was crystallized from Ethyl acetate/Pentane (v:v = 1:4) as a colorless crystal.

^{1}H NMR (500 MHz, $CDCl_3$) δ7.57-7.53 (m, 1H), 7.41-7.38 (m, 4H), 7.32-7.24 (m, 5H), 6.50-6.46 (m, 2H), 6.22 (d, *J* = 9.2 Hz, 1H), 5.29 (dt, *J* = 3.1, 1.5 Hz, 1H), 4.13 (d, *J* = 15.1 Hz, 1H), 4.06 (d, *J* = 15.1 Hz, 1H), 3.83 (dq, *J* = 7.3, 3.1 Hz, 1H), 3.73 (s, 3H), 2.48 (ddd, *J* = 9.2, 7.4, 3.7 Hz, 1H), 2.27 (s, 3H), 2.26-2.20 (m, 2H), 1.91 (s, 3H), 1.88-1.79 (m, 2H).

^{13}C NMR (126 MHz, $CDCl_3$) δ 210.0, 174.8, 173.1, 169.7, 159.1, 148.8, 137.3, 135.9, 134.7, 132.1, 131.4, 129.8, 129.7, 128.8, 128.7, 128.1, 127.4, 126.3, 112.9, 108.1, 55.6, 53.2, 44.0, 36.3, 29.4, 26.7, 24.3, 21.1.

IR ν (cm^{-1}) 2962 (w), 1763 (s), 1701 (m), 1616 (s), 1503 (s), 1447 (s), 1366(s), 1259(s), 1204 (s), 1153 (s), 1098 (s), 1031 (s), 795 (s), 721 (s), 696 (s).

HRMS (ESI/QTOF) m/z: $[M + Na]^+$ Calcd for $C_{32}H_{31}NNaO_6^+$ 548.2044; Found 548.2051.

N-4-acetyl-3-(naphthalen-2-yl)cyclohex-1-en-1-yl)-N-(2-phenylacetyl)benzamide (7l)

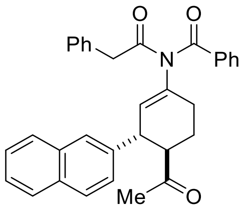

Yield: 60% (29.2 mg), pale oil as a single diastereoisomer, purified by column chromatography on silica gel (petroleum ether/ethyl acetate = 6/1).

^{1}H NMR (400 MHz, $CDCl_3$) δ 7.76-7.72 (m, 1H), 7.68-7.60 (m, 2H), 7.56 (d, *J* = 8.5 Hz, 1H), 7.46-7.41 (m, 6H), 7.33-7.29 (m, 4H), 7.27-7.24 (m, 2H), 6.62 (dd, *J* = 8.4, 1.8 Hz, 1H), 5.47 (dt, *J* = 2.9, 1.5 Hz, 1H), 4.17 (d, *J* = 15.0 Hz, 1H), 4.08 (d, *J* = 15.1 Hz, 1H), 3.89 (dq, *J* = 8.8, 2.8 Hz, 1H), 2.61 (ddd, *J* = 11.8, 9.0, 3.0 Hz, 1H), 2.41-2.33 (m, 2H), 2.01-1.94 (m, 1H), 1.86-1.79 (m, 1H), 1.83 (s, 3H).

^{13}C NMR (101 MHz, $CDCl_3$) δ 210.3, 175.0, 173.2, 140.1, 137.3, 135.8, 134.7, 133.5, 132.5, 132.4, 132.0, 129.7, 128.8, 128.8, 128.6, 128.2, 127.9, 127.7, 127.4, 126.9, 126.2, 126.0, 125.9, 54.6, 44.1, 44.0, 30.1, 27.4, 25.3.

IR ν (cm⁻¹) 1734 (w), 1712 (s), 1686 (s), 1449 (w), 1372 (w), 1360 (m), 1222 (m), 1201 (m), 1133 (m), 821 (w), 790 (w), 751 (m), 695 (m), 671 (w), 663 (w).

HRMS (ESI/QTOF) m/z: $[M + Na]^+$ Calcd for $C_{33}H_{29}NNaO_3^+$ 510.2040; Found 510.2045.

N-4-acetyl-3-(thiophen-3-yl)cyclohex-1-en-1-yl)-N-(2-phenylacetyl)benzamide (7m)

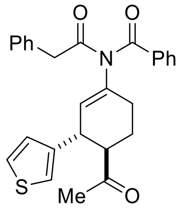

Yield: 57% (24.2 mg), pale oil as a single diastereoisomer, purified by column chromatography on silica gel (petroleum ether/ethyl acetate = 6/1).

^{1}H NMR (400 MHz, $CDCl_3$) δ 7.56-7.51 (m, 1H), 7.45-7.36 (m, 4H), 7.32-7.28 (m, 4H), 7.28-7.23 (m, 1H), 7.10 (dd, *J* = 5.0, 3.0 Hz, 1H), 6.55 (dd, *J* = 3.0, 1.3 Hz, 1H), 6.40 (dd, *J* = 5.0, 1.3 Hz, 1H), 5.45 (dt, *J* = 3.0, 1.5 Hz, 1H), 4.13 (d, *J* = 15.0 Hz, 1H), 4.05 (d, *J* = 15.0 Hz, 1H), 3.87 (dq, *J* = 8.4, 2.8 Hz, 1H), 2.50 (ddd, *J* = 11.0, 8.2, 3.2 Hz, 1H), 2.30-2.22 (m, 2H), 1.96-1.90 (m, 1H), 1.94 (s, 3H), 1.81-1.72 (m, 1H).

^{13}C NMR (101 MHz, $CDCl_3$) δ 210.1, 174.9, 173.1, 143.0, 136.8, 135.7, 134.6, 132.3, 131.5, 129.7, 128.8, 128.7, 128.1, 127.4, 127.1, 126.0, 121.8, 54.0, 44.0, 38.6, 29.6, 27.1, 24.7.

IR ν (cm⁻¹) 1691 (m), 1271 (m), 1250 (m), 1161 (w), 1134 (m), 910 (m), 785 (m), 719 (s), 696 (m), 663 (m)

HRMS (ESI/QTOF) m/z: $[M + Na]^+$ Calcd for $C_{27}H_{25}NNaO_3S^+$ 466.1447; Found 466.1445.

N-(2-phenylacetyl)-N-6-propionyl-1,4,5,6-tetrahydro-[1,1'-biphenyl]-3-yl)benzamide (7n)

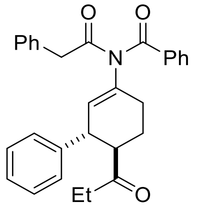

Yield: 63% (28.4 mg), pale oil as a single diastereoisomer, purified by column chromatography on silica gel (petroleum ether/ethyl acetate = 6/1).

^{1}H NMR (400 MHz, $CDCl_3$) δ 7.59-7.53 (m, 1H), 7.45-7.40 (m, 4H), 7.32-7.28 (m, 4H), 7.27-7.24 (m, 1H), 7.12-7.05 (m, 3H), 6.60-6.55 (m, 2H), 5.41 (dt, *J* = 2.8, 1.6 Hz, 1H), 4.16 (d, *J* = 15.0 Hz, 1H), 4.07 (d, *J* = 15.1 Hz, 1H), 3.70 (dq, *J* = 8.9, 2.9 Hz, 1H), 2.46 (ddd, *J* = 11.3, 9.2, 3.2 Hz, 1H), 2.34-2.22 (m, 3H), 1.89-1.75 (m, 3H), 0.78 (t, *J* = 7.3 Hz, 3H).

^{13}C NMR (101 MHz, $CDCl_3$) δ 213.1, 174.9, 173.2, 142.9, 137.2, 135.9, 134.7, 132.24, 132.23, 129.7, 128.8, 128.7, 128.1, 128.0, 127.4, 127.0, 54.2, 44.3, 44.1, 36.3, 27.4, 25.6, 7.5.

IR ν (cm⁻¹) 2938 (w), 1687 (m), 1599 (s), 1492 (s), 1451 (s), 1276 (m), 1131(s), 789 (s), 759 (s), 721 (m), 700 (m).

HRMS (ESI/QTOF) m/z: $[M + Na]^+$ Calcd for $C_{30}H_{29}NNaO_3^+$ 474.2040; Found 474.2045.

Ethyl-5-(N-(2-phenylacetyl)benzamido)-1,2,3,4-tetrahydro-[1,1'-biphenyl]-2-carboxylate (7o)

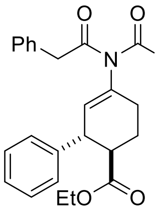

Yield: 40% (18.7 mg), pale oil as a single diastereoisomer, purified by column chromatography on silica gel (petroleum ether/ethyl acetate = 4/1).

^{1}H NMR (400 MHz, $CDCl_3$) δ 7.58-7.54 (m, 1H), 7.45-7.40 (m, 4H), 7.32-7.29 (m, 4H), 7.28-7.25 (m, 1H), 7.14-7.06 (m, 3H), 6.60-6.55 (m, 2H), 5.39 (t, *J* = 2.2 Hz, 1H), 4.18 (d, *J* = 15.0 Hz, 1H), 4.06 (d, *J* = 15.1 Hz, 1H), 4.02-3.93 (m, 2H), 3.74 (dq, *J* = 8.9, 2.9 Hz, 1H), 2.40-2.22 (m, 3H), 2.01-1.91 (m, 2H), 1.07 (t, *J* = 7.1 Hz, 3H).

^{13}C NMR (101 MHz, $CDCl_3$) δ 175.0, 174.3, 173.2, 142.6, 137.4, 135.8, 134.7, 132.2, 131.7, 129.7, 128.8, 128.7, 128.6, 128.1, 128.0, 127.4, 127.0, 60.6, 47.9, 44.4, 44.1, 27.0, 25.6, 14.2.

IR ν (cm^{-1}) 1726 (s), 1689 (s), 1601 (w), 1493 (w), 1450 (m), 1373 (m), 1257 (s), 1174 (s), 1134 (m), 1030 (m), 796 (w), 760 (m), 725 (m), 700 (s), 665 (m).

HRMS (ESI/QTOF) m/z: [M + Na]$^+$ Calcd for $C_{30}H_{29}NNaO_4^+$ 490.1989; Found 490.1978.

4-bromophenyl-5-(N-(2-phenylacetyl)benzamido)-1,2,3,4-tetrahydro-[1,1'-biphenyl]-2-carboxylate (7p)

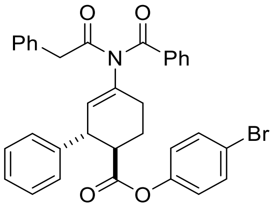

Yield: 44% (26.1 mg), pale oil as a single diastereoisomer, purified by column chromatography on silica gel (petroleum ether/ethyl acetate = 6/1).

^{1}H NMR (500 MHz, $CDCl_3$) δ 7.60-7.56 (m, 1H), 7.46-7.42 (m, 4H), 7.41-7.38 (m, 2H), 7.33-7.29 (m, 4H), 7.29-7.26 (m, 1H), 7.20-7.16 (m, 1H), 7.15-7.10 (m, 2H), 6.70-6.66 (m, 2H), 6.62-6.59 (m, 2H), 5.45-5.43 (m, 1H), 4.20 (d, *J* = 15.1 Hz, 1H), 4.08 (d, *J* = 15.1 Hz, 1H), 3.82 (dq, *J* = 8.8, 2.9 Hz, 1H), 2.53 (ddd, *J* = 11.0, 9.1, 3.1 Hz, 1H), 2.50-2.43 (m, 1H), 2.37-2.30 (m, 1H), 2.18-2.12 (m, 1H), 2.11-2.04 (m, 1H).

^{13}C NMR (126 MHz, $CDCl_3$) δ 175.0, 173.1, 172.7, 149.5, 142.1, 137.5, 135.8, 134.6, 132.5, 132.3, 131.3, 129.7, 128.9, 128.8, 128.12, 128.06, 127.4, 127.3, 123.3, 119.1, 48.0, 44.7, 44.2, 26.9, 25.5.

IR ν (cm^{-1}) 2934 (w), 1752 (s), 1688 (m), 1482 (s), 1451 (s), 1277 (m), 1197 (s), 1132 (s), 1067 (s), 1011 (s), 760 (s), 723 (m), 700 (m).

HRMS (ESI/QTOF) m/z: [M + Na]$^+$ Calcd for $C_{34}H_{28}BrNNaO_4^+$ 616.1094; Found 616.1092.

4-chlorophenyl-5-(N-(2-phenylacetyl)benzamido)-1,2,3,4-tetrahydro-[1,1'-biphenyl]-2-carboxylate (7q)

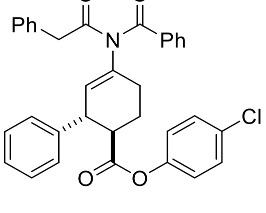

Yield: 42% (23.1 mg), pale oil as a single diastereoisomer, purified by column chromatography on silica gel (petroleum ether/ethyl acetate = 6/1).

^{1}H NMR (400 MHz, $CDCl_3$) δ 7.60-7.56 (m, 1H), 7.46-7.41 (m, 4H), 7.36-7.29 (m, 5H), 7.26-7.22 (m, 2H), 7.18-7.11 (m, 3H), 6.75-6.71 (m, 2H), 6.64-6.59 (m, 2H), 5.44 (dq, *J* = 2.0, 1.0 Hz, 1H), 4.20 (d, *J* = 15.1 Hz, 1H), 4.08

(d, J = 15.1 Hz, 1H), 3.82 (dq, J = 8.9, 2.9 Hz, 1H), 2.57-2.51 (m, 1H), 2.50-2.43 (m, 1H), 2.39-2.29 (m, 1H), 2.19-2.12 (m, 1H), 2.12-2.04 (m, 1H).

^{13}C NMR (101 MHz, $CDCl_3$) δ 175.0, 173.1, 172.8, 149.0, 142.1, 137.5, 135.8, 134.7, 132.3, 131.4, 131.3, 129.7, 129.6, 128.9, 128.82, 128.81, 128.14, 128.07, 127.4, 127.3, 122.9, 48.0, 44.7, 44.2, 26.9, 25.5.

IR ν (cm^{-1}) 2934 (w), 1752 (s), 1486 (s), 1450 (s), 1276 (m), 1198 (s), 1132 (s), 1088 (s), 1013 (s), 760 (s), 722 (m), 700 (s).

HRMS (ESI/QTOF) m/z: $[M + Na]^+$ Calcd for $C_{34}H_{28}ClNNaO_4^+$ 572.1599; Found 572.1614.

N-(6-acetyl-1,4,5,6-tetrahydro-[1,1'-biphenyl]-3-yl)-2-hydroxy-N-(2-phenylacetyl)acetamide(7r)

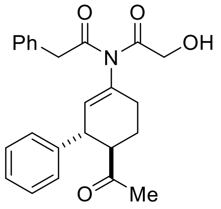

Yield: 55 % (20.0 mg), pale oil as a single diastereoisomer, purified by column chromatography on silica gel (petroleum ether/ethyl acetate = 3/1).

^{1}H NMR (600 MHz, $CDCl_3$) δ 7.37-7.32 (m, 4H), 7.29-7.23 (m, 3H), 7.23-7.20 (m, 1H), 7.11-7.09 (m, 2H), 6.62 (s, 1H), 6.01 (brd, J = 5.0 Hz, 1H), 4.59 (s, 2H), 3.99-3.96 (m, 1H), 3.98 (brt, J = 5.9 Hz, 1H), 3.75 (s, 2H), 2.88-2.84 (m, 1H), 2.86 (ddd, J = 12.4, 6.0, 2.9 Hz, 1H), 2.22-2.09 (m, 2H), 1.96-1.89 (m, 1H), 1.84 (s, 3H), 1.80-1.76 (m, 1H).

^{13}C NMR (151 MHz, $CDCl_3$) δ 210.4, 169.6, 165.0, 140.0, 133.5, 132.8, 129.4, 129.3, 129.3, 128.4, 127.9, 127.4, 115.0, 63.1, 51.9, 42.2, 41.6, 29.3, 27.1, 19.3.

IR ν (cm-1) 1748 (s), 1743 (s), 1739 (m), 1735 (m), 1713 (s), 1706 (s), 1700 (s), 1696 (s), 1690 (s), 1684 (s), 1675 (m), 1670 (m), 1558 (m), 1550 (m), 1545 (m), 1540 (m), 1533 (m), 1159 (m), 1139 (m), 706 (m).

HRMS (ESI/QTOF) m/z: $[M + Na]^+$Calcd for $C_{24}H_{25}NNaO_4$+ 414.1676; Found 414.1686.

N-4-benzoyl-3-pentylcyclohex-1-en-1-yl)-N-(2-phenylacetyl)benzamide (7s)

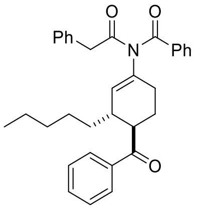

Yield: 73% (36.0 mg), pale oil as a single diastereoisomer, purified by column chromatography on silica gel (petroleum ether/ethyl acetate = 6/1).

^{1}H NMR (400 MHz, CDCl3) δ 7.90-7.86 (m, 2H), 7.59-7.54 (m, 1H), 7.50-7.40 (m, 5H), 7.38-7.33 (m, 2H), 7.33-7.29 (m, 4H), 7.28-7.25 (m, 1H), 5.46 (t, J = 2.6 Hz, 1H), 4.11 (d, J = 14.9 Hz, 1H), 4.08 (d, J = 14.9 Hz, 1H), 3.13 (ddd, J = 11.3, 8.6, 3.0 Hz, 1H), 2.76-2.67 (m, 1H), 2.37-2.28 (m, 1H), 2.18-2.10 (m, 1H), 1.93 (ddt, J = 12.2, 5.7, 3.2 Hz, 1H), 1.71-1.61 (m, 1H), 1.18-1.02 (m, 6H), 1.01-0.93 (m, 2H), 0.79 (t, J = 7.1 Hz, 3H).

^{13}C NMR (126 MHz, $CDCl_3$) δ 202.7, 174.7, 173.4, 136.7, 135.9, 135.9, 134.7, 133.4, 133.2, 132.1, 129.7, 128.9, 128.8, 128.5, 128.3, 127.9, 127.4, 46.0, 44.0, 37.2, 34.0, 31.9, 27.6, 27.0, 26.1, 22.6, 14.2.

IR ν (cm^{-1}) 2926 (w), 1680 (m), 1448(s), 1274 (w), 1130 (s), 787 (s), 700 (m), 671 (s).

HRMS (ESI/QTOF) m/z: $[M + Na]^+$ Calcd for $C_{33}H_{35}NNaO_3^+$ 516.2509; Found 516.2511.

N-4-(4-methoxybenzoyl)-3-pentylcyclohex-1-en-1-yl)-N-(2-phenylacetyl)benzamide (7t)

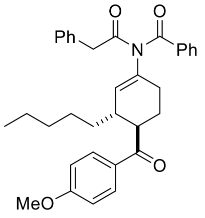

Yield: 54% (28.3 mg), pale oil as a single diastereoisomer, purified by column chromatography on silica gel (petroleum ether/ethyl acetate = 6/1).

^{1}H NMR (400 MHz, $CDCl_3$) δ 7.90-7.86 (m, 2H), 7.50-7.45 (m, 1H), 7.43-7.40 (m, 2H), 7.38-7.33 (m, 2H), 7.32-7.29 (m, 4H), 7.29-7.25 (m, 1H), 6.96-6.91 (m, 2H), 5.46 (t, *J* = 2.3 Hz, 1H), 4.10 (d, *J* = 14.9 Hz, 1H), 4.08 (d, *J* = 14.9 Hz, 1H), 3.87 (s, 3H), 3.08 (ddd, *J* = 11.5, 8.7, 2.9 Hz, 1H), 2.74-2.65 (m, 1H), 2.37-2.29 (m, 1H), 2.16-2.10 (m, 1H), 1.96-1.85 (m, 1H), 1.71-1.63 (m, 1H), 1.17-1.03 (m, 6H), 0.99-0.91 (m, 2H), 0.78 (t, *J* = 7.1 Hz, 3H).

^{13}C NMR (126 MHz, $CDCl_3$) δ 201.2, 174.7, 173.4, 163.8, 135.9, 135.8, 134.7, 133.3, 132.1, 130.7, 129.67, 129.65, 128.8, 128.5, 127.9, 127.3, 114.0, 55.7, 45.7, 44.0, 37.3, 33.9, 31.9, 27.7, 27.2, 26.2, 22.6, 14.2.

IR ν (cm^{-1}) 2927 (w), 1672 (m), 1598(s), 1509 (s), 1453 (s), 1257 (m), 1168 (s), 1027 (s), 842 (s), 696 (m), 608 (s).

HRMS (ESI/QTOF) m/z: $[M + Na]^+$ Calcd for $C_{33}H_{35}NNaO_3^+$ 546.2615; Found 546.2617.

N-4-(4-chlorobenzoyl)-3-pentylcyclohex-1-en-1-yl)-N-(2-phenylacetyl)benzamide (7u)

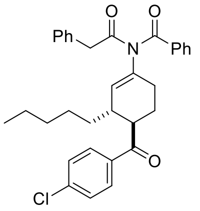

Yield: 73% (38.5 mg), pale oil as a single diastereoisomer, purified by column chromatography on silica gel (petroleum ether/ethyl acetate = 6/1).

^{1}H NMR (400 MHz, $CDCl_3$) δ 7.84-7.80 (m, 2H), 7.51-7.46 (m, 1H), 7.45-7.40 (m, 4H), 7.38-7.33 (m, 2H), 7.32-7.28 (m, 4H), 7.26-7.24 (m, 1H), 5.45 (t, *J* = 2.2 Hz, 1H), 4.11 (d, *J* = 15.0 Hz, 1H), 4.06 (d, *J* = 14.9 Hz, 1H), 3.07 (ddd, *J* = 11.3, 8.5, 3.0 Hz, 1H), 2.74-2.64 (m, 1H), 2.36-2.26 (m, 1H), 2.18-2.09 (m, 1H), 1.94-1.86 (m, 1H), 1.71-1.62 (m, 1H), 1.18-1.02 (m, 6H), 0.99-0.91 (m, 2H), 0.79 (t, *J* = 7.1 Hz, 3H).

^{13}C NMR (126 MHz, $CDCl_3$) δ 201.4, 174.7, 173.3, 139.9, 135.9, 134.9, 134.7, 133.0, 132.1, 129.8, 129.7, 129.3, 128.8, 128.5, 127.9, 127.4, 46.1, 44.0, 37.2, 34.0, 31.9, 27.5, 26.9, 26.2, 22.5, 14.2.

IR ν (cm^{-1}) 2927 (w), 1681 (m), 1588 (s), 1449 (s), 1400 (m), 1276 (w), 1092 (s), 1011 (s), 718 (s), 696 (m), 668 (s).

HRMS (nanochip-ESI/LTQ-Orbitrap) m/z: $[M + H]^+$ Calcd for $C_{33}H_{35}ClNO_3^+$ 528.2300; Found 528.2297.

N-4-(4-bromobenzoyl)-3-pentylcyclohex-1-en-1-yl)-N-(2-phenylacetyl)benzamide (7v)

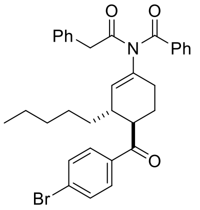

Yield: 43% (24.6 mg), pale oil as a single diastereoisomer, purified by column chromatography on silica gel (petroleum ether/ethyl acetate = 6/1).

^{1}H NMR (500 MHz, $CDCl_3$) δ 7.76-7.72 (m, 2H), 7.63-7.58 (m, 2H), 7.50-7.46 (m, 1H), 7.43-7.39 (m, 2H), 7.38-7.34 (m, 2H), 7.32-7.28 (m, 4H), 7.26-7.24 (m, 1H), 5.45 (brs, 1H), 4.11 (d, *J* = 15.0 Hz, 1H), 4.07 (d, *J* = 14.9 Hz, 1H), 3.06 (ddd, *J* = 11.3, 8.4, 2.9 Hz, 1H), 2.73-2.65 (m, 1H), 2.35-2.27 (m, 1H), 2.17-2.11 (m, 1H), 1.93-1.86 (m, 1H), 1.69-1.62 (m, 1H), 1.19-1.04 (m, 6H), 0.99-0.92 (m, 2H), 0.79 (t, *J* = 7.2 Hz, 3H).

^{13}C NMR (126 MHz, $CDCl_3$) δ 201.6, 174.7, 173.3, 135.9, 135.3, 134.7, 133.0, 132.3, 132.1, 129.9, 129.7, 128.8, 128.6, 128.5, 127.9, 127.4, 46.0, 44.0, 37.2, 34.0, 31.9, 27.5, 26.9, 26.2, 22.5, 14.2.

IR ν (cm^{-1}) 2926 (w), 1680 (m), 1583 (s), 1451 (s), 1397 (m), 1274 (w), 1070 (s), 1008 (s), 710 (m), 671 (s).

HRMS (ESI/QTOF) m/z: $[M + Na]^+$ Calcd for $C_{33}H_{34}BrNNaO_3^+$ 594.1614; Found 594.1618.

N-4-(4-cyanobenzoyl)-3-pentylcyclohex-1-en-1-yl)-N-(2-phenylacetyl)benzamide (7w)

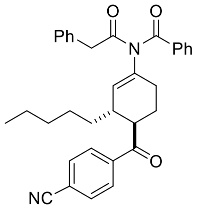

Yield: 58% (30.0 mg), pale oil as a single diastereoisomer, purified by column chromatography on silica gel (petroleum ether/ethyl acetate = 6/1).

^{1}H NMR (500 MHz, $CDCl_3$) δ 7.95-7.94 (m, 2H), 7.77-7.76 (m, 2H), 7.51-7.47 (m, 1H), 7.42-7.39 (m, 2H), 7.38-7.34 (m, 2H), 7.31-7.25 (m, 5H), 5.45 (t, *J* = 2.3 Hz, 1H), 4.11 (d, *J* = 14.9 Hz, 1H), 4.07 (d, *J* = 15.0 Hz, 1H), 3.08 (ddd, *J* = 11.1, 8.3, 2.9 Hz, 1H), 2.73-2.67 (m, 1H), 2.35-2.27 (m, 1H), 2.18-2.12 (m, 1H), 1.93-1.87 (m, 1H), 1.70-1.62 (m, 1H), 1.17-1.03 (m, 6H), 0.99-0.93 (m, 2H), 0.79 (t, *J* = 7.2 Hz, 3H).

^{13}C NMR (126 MHz, $CDCl_3$) δ 201.3, 174.7, 173.3, 139.6, 135.9, 135.8, 134.6, 132.8, 132.6, 132.2, 129.6, 128.8, 128.7, 128.5, 128.0, 127.4, 118.0, 116.6, 46.4, 44.0, 37.1, 34.1, 31.9, 27.4, 26.6, 26.2, 22.5, 14.2.

IR ν (cm^{-1}) 2954 (w), 2927 (w), 2855 (w), 1679 (s), 1405 (w), 1315 (m), 1158 (w), 1128 (m), 1113 (w), 946 (w), 931 (w), 791 (w), 754 (w), 722 (m), 705 (m), 694 (m).

HRMS (ESI/QTOF) m/z: $[M + Na]^+$ Calcd for $C_{34}H_{34}N_2NaO_3^+$ 541.2462; Found 541.2461.

N-3-pentyl-4-(thiophene-3-carbonyl)cyclohex-1-en-1-yl)-N-(2-phenylacetyl)benzamide (7x)

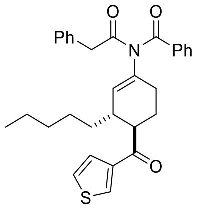

Yield: 60% (30.0 mg), pale oil as 10:1 mixture of diastereoisomers, purified by TLC (petroleum ether/ ethyl acetate = 6/1).

^{1}H NMR (600 MHz, $CDCl_3$) δ 7.67-7.64 (m, 2H), 7.50-7.47 (m, 1H), 7.42-7.40 (m, 2H), 7.37-7.34 (m, 2H), 7.32-7.30 (m, 4H), 7.27-7.25 (m, 1H), 7.13 (dd, *J* = 4.9, 3.8 Hz, 1H), 5.44-5.42 (m, 1H), 4.11 (d, *J* = 14.9 Hz, 1H), 4.07 (d, *J* = 15.0 Hz, 1H), 2.92 (ddd, *J* = 11.7, 9.0, 2.9 Hz, 1H), 2.67 (s, 1H), 2.37-2.29 (m, 1H), 2.17 (ddt, *J* = 17.2, 5.0, 2.4 Hz, 1H), 1.97 (ddt, *J* = 13.2, 5.7, 3.0 Hz, 1H), 1.74 (dtd, *J* = 13.2, 11.3, 5.2 Hz, 1H), 1.17-1.03 (m, 6H), 0.97-0.92 (m, 2H), 0.79 (t, *J* = 7.3 Hz, 3H).

^{13}C NMR (151 MHz, $CDCl_3$) δ 195.6, 174.8, 173.4, 144.3, 136.0, 135.9, 134.7, 134.3, 132.9, 132.1, 131.9, 129.7, 128.8, 128.5, 128.4, 127.9, 127.4, 48.1, 44.0, 37.4, 33.8, 31.9, 27.6, 27.4, 26.0, 22.6, 14.2.

IR ν (cm[-1]) 2926 (w), 1688 (m), 1655 (m), 1450 (s), 1414 (m), 1231 (w), 1131 (s), 856 (s), 783 (s), 721(m), 671(s).

HRMS (ESI/QTOF) m/z: [M + Na]+ Calcd for $C_{31}H_{33}NNaO_3S^+$ 522.2073; Found 522.2077.

N-3-(2-((tert-butyldimethylsilyl)oxy)ethyl)-4-(4-chlorobenzoyl)cyclohex-1-en-1-yl)-N-(2-phenylacetyl)benzamide (7y)

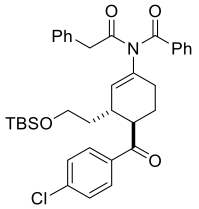

Yield: 60% (37.0 mg), pale oil as 11:1 mixture of diastereoisomers, purified by TLC (petroleum ether/ ethyl acetate = 6/1).

¹H NMR (400 MHz, CDCl3) δ 7.83-7.80 (m, 2H), 7.48-7.45 (m, 1H), 7.44-7.39 (m, 4H), 7.37-7.33 (m, 2H), 7.31-7.28 (m, 4H), 7.26-7.22 (m, 1H), 5.53-5.50 (m, 1H), 4.08 (s, 2H), 3.44 (td, *J* = 6.4, 1.9 Hz, 2H), 3.21 (ddd, *J* = 10.7, 7.9, 3.1 Hz, 1H), 2.86-2.77 (m, 1H), 2.37-2.29 (m, 1H), 2.16-2.07 (m, 1H), 1.92 (dq, *J* = 13.0, 4.7 Hz, 1H), 1.72-1.64 (m, 1H), 1.43-1.36 (m, 1H), 1.33-1.27 (m, 1H), 0.80 (s, 9H), -0.06 (s, 3H), -0.08 (s, 3H).

¹³C NMR (126 MHz, $CDCl_3$) δ 201.01, 174.6, 173.3, 139.8, 135.9, 135.8, 134. 8, 134.7, 132.7, 132.1, 129.8, 129.7, 129.2, 128.8, 128.5, 128.0, 127.4, 61.0, 45.9, 43.9, 37.0, 34.7, 27.3, 26.4, 26.1, 18.4, -5.2, -5.3.

IR ν (cm[-1]) 2926 (w), 1682 (m), 1588 (m), 1400 (s), 1252 (m), 1093 (s), 835 (s), 777 (s), 718 (m), 697 (m).

HRMS (ESI/QTOF) m/z: [M + Na]+ Calcd for $C_{36}H_{42}ClNNaO_4Si^+$ 638.2464; Found 638.2469.

N-4-(4-chlorobenzoyl)-3-(3-cyanopropyl)cyclohex-1-en-1-yl)-N-(2-phenylacetyl)benzamide (7z)

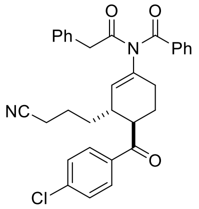

Yield: 38% (20.0 mg), pale oil as 10:1 mixture of diastereoisomers, purified by TLC (petroleum ether/ethyl acetate = 4/1).

¹H NMR (800 MHz, $CDCl_3$) δ 7.83-7.79 (m, 2H), 7.53-7.49 (m, 1H), 7.46-7.44 (m, 2H), 7.43-7.41 (m, 2H), 7.41-7.37 (m, 2H), 7.33-7.28 (m, 4H), 7.27-7.24 (m, 1H), 5.36 (s, 1H), 4.12 (d, *J* = 15.0 Hz, 1H), 4.07 (d, *J* = 15.1 Hz, 1H), 3.03 (dt, *J* = 11.7, 5.8 Hz, 1H), 2.79 (brs, 1H), 2.35-2.28 (m, 1H), 2.19-2.09 (m, 3H), 1.97-1.92 (m, 1H), 1.67-1.60 (m, 1H), 1.42-1.36 (m, 1H), 1.26-1.20 (m, 2H), 1.16-1.09 (m, 1H).

¹³C NMR (201 MHz, $CDCl_3$) δ 200.6, 174.8, 173.8, 140.2, 137.0, 135.7, 134.6, 134.4, 132.4, 131.6, 129.8, 129.6, 129.4, 128.9, 128.6, 128.0, 127.4, 119.3, 45.8, 44.1, 36.1, 33.0, 27.5, 27.0, 22.4, 17.3.

IR ν (cm[-1]) 2930 (w), 1681(m), 1588(s), 1400 (s), 1276 (m), 1091(s), 1011(s), 844 (s), 777 (s), 718 (m), 697 (m).

HRMS (ESI/QTOF) m/z: [M + Na]+ Calcd for $C_{32}H_{29}ClN_2NaO_3^+$ 547.1759; Found 547.1760.

(*E*)-N-(4-phenylbuta-1,3-dien-2-yl)benzamide (8a)

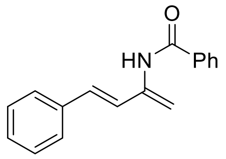

Yield: 83% yield (21.3 mg), pale oil as a single isomer, purified by column chromatography on silica gel (petroleum ether/ethyl acetate = 10/1).

The compound of **8a** was crystallized from CH_2Cl_2 as a colorless crystal.

^{1}H NMR (400 MHz, $CDCl_3$) δ 7.90-7.85 (m, 2H), 7.60-7.55 (m, 1H), 7.53-7.48 (m, 2H), 7.46-7.42 (m, 3H), 7.37-7.32 (m, 2H), 7.30-7.27 (m, 1H), 6.74 (d, *J* = 16.3 Hz, 1H), 6.62 (d, *J* = 16.3 Hz, 1H), 5.98 (s, 1H), 5.20 (s, 1H).

^{13}C NMR (101 MHz, $CDCl_3$) δ 166.3, 138.0, 136.2, 135.1, 132.1, 129.1, 129.0, 128.4, 127.5, 127.2, 127.1, 126.9, 108.1.

IR ν (cm^{-1}) 1706 (s), 1686 (s), 1678 (s), 1361 (w), 1349 (w), 1339 (w), 1314 (m), 1280 (s), 1256 (m), 1176 (w), 1160 (m), 790 (w), 759 (m), 742 (w), 721 (m), 671 (w), 662 (w).

HRMS (ESI/QTOF) m/z: $[M + Na]^+$ Calcd for $C_{17}H_{15}NNaO^+$ 272.1046; Found 272.1050.

N-((1*R*,6*S*)-6-(4-chlorobenzoyl)-1,4,5,6-tetrahydro-[1,1'-biphenyl]-3-yl)benzamide (9a)

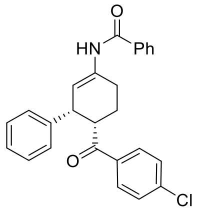

Yield: 48% yield (19.9 mg), pale oil as a single diastereoisomer, purified by column chromatography on silica gel (petroleum ether/ethyl acetate = 3/1). $[\alpha]_D^{24}$ -105.9 (c 1.0, DCM). 93% *ee* (determined by SFC: Chiralpak IC Column, 20% MeOH in supercritical CO_2 as eluent, 2 mL/min; TR = 3.81 (major), 5.16 (minor).

^{1}H NMR (500 MHz, $CDCl_3$) δ 7.86-7.78 (m, 4H), 7.56-7.51 (m, 1H), 7.50-7.43 (m, 4H), 7.17 (s, 1H), 7.16-7.10 (m, 3H), 6.84-6.79 (m, 2H), 6.37 (dd, *J* = 5.4, 1.7 Hz, 1H), 4.16 (t, *J* = 5.6 Hz, 1H), 3.87 (ddd, *J* = 12.1, 5.8, 2.7 Hz, 1H), 2.67-2.51 (m, 2H), 2.18 (dtd, *J* = 13.8, 11.6, 6.2 Hz, 1H), 1.91-1.84 (m, 1H).

^{13}C NMR (126 MHz, $CDCl_3$) δ 200.1, 166.2, 139.6, 139.5, 135.7, 135.2, 134.2, 131.9, 129.9, 129.4, 129.3, 128.9, 128.1, 127.4, 127.1, 114.7, 46.5, 43.5, 27.9, 19.2.

IR ν (cm^{-1}) 1684 (s), 1680 (s), 1675 (s), 1670 (s), 1663 (s), 1653 (s), 1647 (s), 1539 (s), 1534 (s), 1527 (s), 1522 (s), 1517 (s), 1489 (s), 706 (s), 698 (s).

HRMS (ESI/QTOF) m/z: $[M + Na]^+$ Calcd for $C_{26}H_{22}ClNNaO_2^+$ 438.1231; Found 438.1238.

N-((1*R*,6*S*)-6-(4-chlorobenzoyl)-1,4,5,6-tetrahydro-[1,1'-biphenyl]-3-yl)benzamide (*trans*-9a)

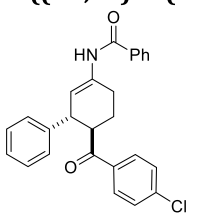

Yield: 10% yield (4.3 mg), pale oil as a single diastereoisomer, purified by column chromatography on silica gel (petroleum ether/ethyl acetate = 3/1). This compound is racemic and was isolated from the reaction carried out in the absence of chiral squaramide **10a**. It was prepared for mechanistic studies.

^{1}H NMR (500 MHz, $CDCl_3$) δ 7.80 (d, *J* = 8.2 Hz, 2H), 7.62 (d, *J* = 8.4 Hz, 1H), 7.55 – 7.51 (m, 1H), 7.49 – 7.43 (m, 2H), 7.30 (d, *J* = 8.5, 2H), 7.25 (d, *J* = 8.4 Hz, 2H), 7.23 – 7.16 (m, 3H), 7.15 – 7.10 (m, 1H), 6.10 (s, 1H), 4.10 (dd, *J* = 9.4, 2.9 Hz, 1H), 3.57 (td, *J* = 9.9, 3.3 Hz, 1H), 2.79 (ddt, *J* = 18.0, 9.8, 4.3 Hz, 1H), 2.55 – 2.48 (m, 1H), 2.09 – 1.96 (m, 2H).
^{13}C NMR (126 MHz, $CDCl_3$) δ 201.9, 166.0, 144.0, 139.6, 135.2, 135.1, 133.6, 131.9, 129.7, 129.0, 128.9, 128.7, 128.3, 127.1, 126.9, 117.0, 49.8, 43.9, 27.6, 26.6.
IR ν (cm^{-1}) 1679 (s), 1680 (s), 1640 (s), 1647 (s), 1539 (s), 1534 (s), 1533 (s), 1522 (s), 1520 (s), 1489 (s), 716 (s), 679 (s).
HRMS (ESI/QTOF) m/z: $[M + Na]^+$ Calcd for $C_{26}H_{22}ClNNaO_2^+$ 438.1231; Found 438.1238.

N-((1*R*,6*S*)-6-(4-fluorobenzoyl)-1,4,5,6-tetrahydro-[1,1'-biphenyl]-3-yl)benzamide (9b)

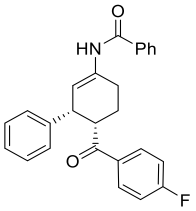

Yield: 48% yield (19.2 mg), pale oil as a single diastereoisomer, purified by column chromatography on silica gel (petroleum ether/ethyl acetate = 3/1). $[\alpha]_D^{24}$ -161.5 (c 0.55, DCM). 90% *ee* (determined by SFC: Chiralpak IC Column, 20% MeOH in supercritical CO_2 as eluent, 2 mL/min; TR = 2.23 (major), 2.86 (minor).
^{1}H NMR (400 MHz, $CDCl_3$) δ 7.95-7.89 (m, 2H), 7.83-7.78 (m, 2H), 7.55-7.50 (m, 1H), 7.48-7.43 (m, 2H), 7.24 (s, 1H), 7.19-7.11 (m, 5H), 6.84-6.78 (m, 2H), 6.37 (dd, *J* = 5.4, 1.8 Hz, 1H), 4.16 (t, *J* = 5.8 Hz, 1H), 3.87 (ddd, *J* = 12.1, 5.8, 2.7 Hz, 1H), 2.65-2.51 (m, 2H), 2.17 (dtd, *J* = 13.6, 11.4, 6.4 Hz, 1H), 1.87 (ddq, *J* = 12.7, 4.4, 2.1 Hz, 1H).
^{13}C NMR (101 MHz, $CDCl_3$) δ 199.7, 166.2, 165.76 (d, *J* = 254.8 Hz), 139.6, 135.2, 134.2, 133.8 (d, *J* = 3.0 Hz), 131.9, 131.1 (d, *J* = 9.2 Hz), 129.4, 128.9, 128.0, 127.3, 127.1, 116.1 (d, *J* = 21.7 Hz), 114.8, 46.4, 43.6, 27.9, 19.3.
^{19}F NMR (377 MHz, $CDCl_3$) δ -105.4.
IR ν (cm-1) 1693 (w), 1656 (s), 1650 (s), 1638 (m), 1632 (w), 1619 (w), 1596 (s), 1579 (m), 1555 (w), 1552 (w), 1547 (m), 1543 (m), 1453 (w), 1445 (w), 1408 (w), 1269 (m), 1011 (w), 907 (m), 810 (w), 800 (w), 729 (m), 717 (m), 710 (m), 682 (w)
HRMS (ESI/QTOF) m/z: $[M + H]^+$ Calcd for $C_{26}H_{23}FNO_2^+$ 400.1707; Found 400.1709.

N-((1*R*,6*S*)-6-(4-fluorobenzoyl)-1,4,5,6-tetrahydro-[1,1'-biphenyl]-3-yl)-4-methoxybenzamide (9c)

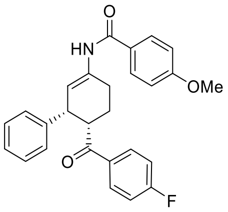

Yield: 70% yield (30.0 mg), pale oil as a single diastereoisomer, purified by column chromatography on silica gel (petroleum ether/ethyl acetate = 3/1). $[\alpha]_D^{24}$ -147.07 (c 0.35, DCM). 82% *ee* (determined by SFC: Chiralpak IC Column, 20% MeOH in supercritical CO_2 as eluent, 2 mL/min; TR = 4.69 (major), 6.39 (minor).
^{1}H NMR (400 MHz, $CDCl_3$) δ 7.95-7.89 (m, 2H), 7.77 (d, *J* = 8.8 Hz, 2H), 7.20-7.09 (m, 6H), 6.94 (d, *J* = 8.6 Hz, 2H), 6.85-6.79 (m, 2H), 6.33 (d, *J* = 5.3 Hz, 1H), 4.16 (t, *J* = 5.1 Hz, 1H), 3.89-3.84 (m, 1H), 3.86 (s, 3H), 2.67-2.50 (m, 2H), 2.17 (dtd, *J* = 13.6, 11.6, 6.3 Hz, 1H), 1.91-1.81 (m, 1H).

^{13}C NMR (101 MHz, $CDCl_3$) δ 199.8, 165.76 (d, *J* = 254.6 Hz), 165.7, 162.6, 139.7, 134.3, 133.8 (d, *J* = 3.0 Hz), 131.1 (d, *J* = 9.1 Hz), 129.4, 129.0, 128.0, 127.4, 127.3, 116.05 (d, *J* = 21.8 Hz), 114.4, 114.1, 55.6, 46.5, 43.6, 28.0, 19.3.

^{19}F NMR (376 MHz, $CDCl_3$) δ -105.5.

IR ν (cm^{-1}) 1695 (w), 1687 (w), 1647 (m), 1642 (m), 1636 (m), 1605 (m), 1539 (m), 1527 (m), 1437 (w), 1409 (w), 1387 (w), 1207 (m), 1189 (m), 1156 (m), 1028 (w), 942 (w), 913 (w), 757 (w), 729 (m), 707 (w).

HRMS (ESI/QTOF) m/z: [M + H]$^+$ Calcd for $C_{27}H_{25}FNO_3^+$ 430.1813; Found 430.1820.

N-((1*R*,6*S*)-6-(4-fluorobenzoyl)-1,4,5,6-tetrahydro-[1,1'-biphenyl]-3-yl)-4-methylbenzamide (9d)

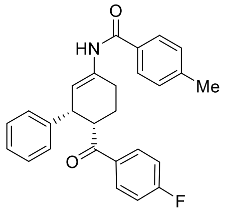

Yield: 56% yield (23.1 mg), pale oil as a single diastereoisomer, purified by column chromatography on silica gel (petroleum ether/ethyl acetate = 3/1). $[\alpha]_D^{24}$ -154.26 (c 0.45, DCM). 86% *ee* (determined by SFC: Chiralpak IC Column, 20% MeOH in supercritical CO_2 as eluent, 2 mL/min; TR = 3.16 (major), 4.46 (minor).

^{1}H NMR (400 MHz, $CDCl_3$) δ 7.95-7.89 (m, 2H), 7.75-7.68 (m, 2H), 7.29-7.22 (m, 2H), 7.20-7.09 (m, 6H), 6.84-6.80 (m, 2H), 6.35 (d, *J* = 5.3 Hz, 1H), 4.16 (t, *J* = 5.6 Hz, 1H), 3.87 (ddd, *J* = 12.1, 5.8, 2.7 Hz, 1H), 2.66-2.50 (m, 2H), 2.41 (s, 3H), 2.27-2.10 (m, 1H), 1.93-1.83 (m, 1H).

^{13}C NMR (101 MHz, $CDCl_3$) δ 199.7, 165.8 (d, *J* = 254.6 Hz), 166.1, 142.4, 139.7, 134.3, 133.8 (d, *J* = 3.1 Hz), 132.3, 131.1 (d, *J* = 9.1 Hz), 129.6, 129.4, 128.0, 127.3, 127.1, 116.1 (d, *J* = 21.8 Hz), 114.6, 46.5, 43.6, 28.0, 21.6, 19.3.

^{19}F NMR (377 MHz, $CDCl_3$) δ -105.5.

IR ν (cm^{-1}) 1695 (w), 1687 (m), 1676 (s), 1671 (s), 1664 (s), 1654 (s), 1647 (s), 1582 (w), 1538 (m), 1452 (w), 1249 (m), 1234 (s), 1208 (m), 1191 (w), 1178 (w), 1156 (m), 1011 (w), 912 (m), 861 (w), 759 (m), 728 (m), 707 (m), 700 (m).

HRMS (ESI/QTOF) m/z: [M + H]$^+$ Calcd for $C_{27}H_{25}FNO_2^+$ 414.1864; Found 414.1857.

4-fluoro-N-((1*R*,6*S*)-6-(4-fluorobenzoyl)-1,4,5,6-tetrahydro-[1,1'-biphenyl]-3-yl)benzamide (9e)

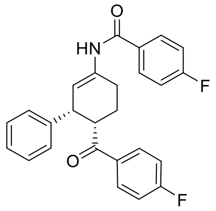

Yield: 53% yield (18.0 mg), pale oil as a single diastereoisomer, purified by column chromatography on silica gel (petroleum ether/ethyl acetate = 3/1). $[\alpha]_D^{24}$ -141.41 (c 0.585, DCM). 84% *ee* (determined by SFC: Chiralpak IC Column, 20% MeOH in supercritical CO_2 as eluent, 2 mL/min; TR = 1.57 (major), 2.06 (minor).

^{1}H NMR (400 MHz, $CDCl_3$) δ 7.95-7.87 (m, 2H), 7.85-7.77 (m, 2H), 7.20-7.10 (m, 8H), 6.84-6.79 (m, 2H), 6.34 (d, *J* = 5.3 Hz, 1H), 4.16 (t, *J* = 5.7 Hz, 1H), 3.87 (ddd, *J* = 12.0, 5.7, 2.7 Hz, 1H), 2.65-2.50 (m, 2H), 2.18 (dtd, *J* = 13.9, 11.5, 6.2 Hz, 1H), 1.92-1.84 (m, 1H).

^{13}C NMR (101 MHz, $CDCl_3$) δ 199.7, 165.8 (d, *J* = 252.5 Hz), 165.1, 165.0 (d, *J* = 252.5 Hz), 139.6, 134.1, 133.8 (d, *J* = 3.0 Hz), 131.4 (d, *J* = 3.1 Hz), 131.1 (d, *J* = 9.1 Hz), 129.5 (d, *J* = 8.9 Hz), 129.3, 128.0, 127.4, 116.1 (d, *J* = 21.8 Hz), 116.0 (d, *J* = 22.0 Hz), 115.0, 46.4, 43.6, 27.9, 19.4.

^{19}F NMR (377 MHz, $CDCl_3$) δ -105.4, -107.6.

IR ν (cm^{-1}) 1695 (w), 1687 (m), 1676 (m), 1671 (m), 1664 (m), 1597 (s), 1549 (w), 1544 (m), 1540 (m), 1518 (w), 1505 (s), 1491 (m), 1272 (m), 1226 (s), 1207 (m), 1181 (w), 1156 (m), 840 (m), 805 (w), 758 (m), 729 (m), 677 (w), 673 (w), 668 (w).

HRMS (ESI/QTOF) m/z: [M + H]$^+$ Calcd for $C_{26}H_{22}F_2NO_2^+$ 418.1613; Found 418.1606.

N-((1*R*,6*S*)-6-acetyl-1,4,5,6-tetrahydro-[1,1'-biphenyl]-3-yl)-4-methoxybenzamide (9f)

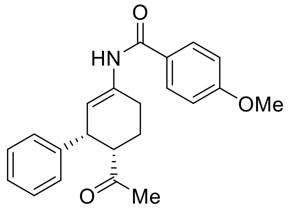

Yield: 55% yield (19.2 mg), pale oil as a single diastereoisomer, purified by column chromatography on silica gel (petroleum ether/ethyl acetate = 5/1). $[\alpha]_D^{24}$ -57.42 (c 0.57, DCM). 87% *ee* (determined by SFC: Chiralpak IC Column, 20% MeOH in supercritical CO_2 as eluent, 2 mL/min; TR = 5.79 (major), 6.89 (minor).

^{1}H NMR (400 MHz, $CDCl_3$) δ 7.77-7.72 (m, 2H), 7.29-7.24 (m, 2H), 7.23-7.17 (m, 3H), 7.09 (s, 1H), 6.95-6.92 (m, 2H), 6.25 (d, *J* = 5.2 Hz, 1H), 4.07 (t, *J* = 5.7 Hz, 1H), 3.85 (s, 3H), 2.96 (ddd, *J* = 12.4, 6.0, 2.9 Hz, 1H), 2.59-2.46 (m, 2H), 2.10-1.99 (m, 1H), 1.90-1.83 (m, 1H), 1.86 (s, 3H).

^{13}C NMR (101 MHz, $CDCl_3$) δ 210.6, 165.6, 162.5, 140.4, 134.3, 129.4 128.9, 128.4, 127.4, 127.3, 114.6, 114.1, 55.6, 52.2, 42.5, 29.3, 27.7, 19.6.

IR ν (cm^{-1}) 1716 (w), 1706 (m), 1701 (m), 1678 (w), 1674 (w), 1605 (m), 1576 (w), 1467 (w), 1463 (w), 1418 (w), 1377 (w), 1370 (w), 1352 (w), 1308 (m), 1286 (w), 1028 (w), 844 (w), 764 (w), 751 (w), 728 (w), 701 (m).

HRMS (ESI/QTOF) m/z: [M + Na]$^+$ Calcd for $C_{22}H_{23}NNaO_3^+$ 372.1570; Found 372.1568.

N-((1*R*,6*S*)-6-(4-fluorobenzoyl)-1,4,5,6-tetrahydro-[1,1'-biphenyl]-3-yl)-2-naphthamide (9g)

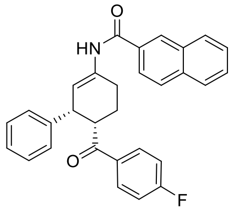

Yield: 49% yield (22.0 mg), pale oil as a single diastereoisomer, purified by column chromatography on silica gel (petroleum ether/ethyl acetate = 3/1). $[\alpha]_D^{24}$ -160.65 (c 0.335, DCM). 84% *ee* (determined by SFC: Chiralpak IC Column, 20% MeOH in supercritical CO_2 as eluent, 2 mL/min; TR = 6.20 (major), 8.83 (minor).

^{1}H NMR (400 MHz, $CDCl_3$) δ 8.31 (s, 1H), 7.96-7.85 (m, 6H), 7.62-7.53 (m, 2H), 7.37 (s, 1H), 7.20-7.12 (m, 5H), 6.87-6.81 (m, 2H), 6.43 (d, *J* = 5.3 Hz, 1H), 4.19 (t, *J* = 5.6 Hz, 1H), 3.90 (ddd, *J* = 12.1, 5.8, 2.7 Hz, 1H), 2.72-2.56 (m, 2H), 2.21 (tdd, *J* = 13.8, 10.7, 6.4 Hz, 1H), 1.95-1.86 (m, 1H).

^{13}C NMR (126 MHz, $CDCl_3$) δ 199.7, 166.2, 165.8 (d, *J* = 254.8 Hz), 139.7, 135.0, 134.3, 133.8 (d, *J* = 3.1 Hz), 132.8, 132.4, 131.1 (d, *J* = 9.0 Hz), 129.4, 129.1, 128.9, 128.1, 128.05, 128.0, 127.5, 127.4, 127.2, 123.7, 116.10 (d, *J* = 21.8 Hz), 114.8, 46.5, 43.6, 28.1, 19.4.

^{19}F NMR (376 MHz, $CDCl_3$) δ -105.4.

IR ν (cm^{-1}) 1742 (w), 1737 (m), 1731 (m), 1708 (w), 1687 (m), 1678 (s), 1672 (s), 1643 (m), 1557 (w), 1552 (w), 1548 (m), 1543 (m), 1491 (m), 1478 (w), 1452 (w), 1358 (w), 1312 (m), 1299 (m), 1285 (m), 1155 (m), 1129 (w), 1011 (w), 940 (w), 906 (w), 870 (w), 858 (m), 844 (m), 825 (m), 699 (s), 685 (w), 680 (w), 676 (w), 669 (w), 652 (w).

HRMS (ESI/QTOF) m/z: $[M + H]^+$ Calcd for $C_{30}H_{25}FNO_2^+$ 450.1864; Found 450.1858.

N-((1*R*,6*S*)-6-(4-fluorobenzoyl)-1,4,5,6-tetrahydro-[1,1'-biphenyl]-3-yl)-[1,1'-biphenyl]-4-carboxamide (9h)

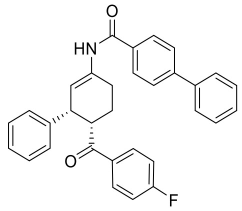

Yield: 57% yield (27.0 mg), pale oil as a single diastereoisomer, purified by column chromatography on silica gel (petroleum ether/ethyl acetate = 3/1). $[\alpha]_D^{24}$ -148.00 (c 0.8, DCM). 90% *ee* (determined by SFC: Chiralpak IC Column, 20% MeOH in supercritical CO_2 as eluent, 2 mL/min; TR = 6.49 (major), 9.55 (minor).

The compound of **9h** was crystallized from Ethyl acetate/Pentane (v:v = 1:5) as a colorless crystal.

^{1}H NMR (400 MHz, $CDCl_3$) δ 7.96-7.86 (m, 4H), 7.71-7.66 (m, 2H), 7.65-7.60 (m, 2H), 7.51-7.45 (m, 2H), 7.43-7.37 (m, 1H), 7.24 (s, 1H), 7.21-7.10 (m, 5H), 6.88-6.76 (m, 2H), 6.40 (d, *J* = 5.3 Hz, 1H), 4.18 (t, *J* = 5.8 Hz, 1H), 3.89 (ddd, *J* = 12.1, 5.8, 2.7 Hz, 1H), 2.70-2.53 (m, 2H), 2.20 (dtd, *J* = 13.7, 11.5, 6.3 Hz, 1H), 1.90 (ddd, *J* = 12.2, 6.2, 3.1 Hz, 1H).

^{13}C NMR (101 MHz, $CDCl_3$) δ 199.7, 165.9, 165.8 (d, *J* = 254.6 Hz), 144.8, 140.0, 139.7, 134.2, 133.84 (d, *J* = 3.0 Hz), 133.8, 131.1 (d, *J* = 9.2 Hz), 129.4, 129.2, 128.3, 128.0, 127.6, 127.6, 127.4, 127.3, 116.1 (d, *J* = 21.8 Hz), 114.8, 46.5, 43.6, 28.0, 19.4.

^{19}F NMR (377 MHz, $CDCl_3$) δ -105.4.

IR ν (cm^{-1}) 1695 (w), 1688 (m), 1683 (s), 1676 (s), 1670 (s), 1642 (m), 1628 (w), 1559 (w), 1506 (s), 1465 (w), 1448 (w), 1155 (m), 1100 (w), 1013 (w), 1008 (w), 943 (w), 911 (m), 780 (w), 760 (m), 746 (s), 728 (s), 707 (m), 698 (s), 673 (w), 669 (w), 664 (w).

HRMS (ESI/QTOF) m/z: $[M + H]^+$ Calcd for $C_{32}H_{27}FNO_2^+$ 476.2020; Found 476.2024.

N-((3*R*,4*S*)-4-(4-fluorobenzoyl)-3-pentylcyclohex-1-en-1-yl)benzamide (9i)

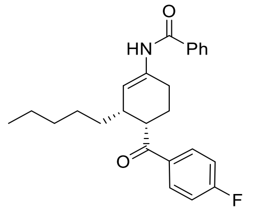

Yield: 43% yield (16.9 mg), pale oil as a single diastereoisomer, purified by column chromatography on silica gel (petroleum ether/ethyl acetate = 4/1). $[\alpha]_D^{24}$ -70.05 (c 0.56, DCM). 88% *ee* (determined by SFC: Chiralpak IC Column, 20% MeOH in supercritical CO_2 as eluent, 2 mL/min; TR = 1.67 (major), 1.93 (minor).

^{1}H NMR (400 MHz, $CDCl_3$) δ 8.06-7.95 (m, 2H), 7.80-7.75 (m, 2H), 7.55-7.49 (m, 1H), 7.48-7.43 (m, 2H), 7.18-7.10 (m, 2H), 7.02 (s, 1H), 6.46 (d, *J* = 5.4 Hz, 1H), 3.61 (ddd, *J* = 11.7, 5.4, 2.7 Hz, 1H), 2.78-2.69 (m, 1H), 2.54-2.42 (m, 1H), 2.37-2.28 (m, 1H), 2.09 (dtd, *J* = 13.7, 11.5, 5.8 Hz, 1H), 1.97-1.89 (m, 1H), 1.36-1.22 (m, 2H), 1.17-0.98 (m, 6H), 0.76 (t, *J* = 6.9 Hz, 3H).

^{13}C NMR (101 MHz, $CDCl_3$) δ 201.2, 166.0, 165.8 (d, *J* = 255.7 Hz), 135.3, 133.2 (d, *J* = 3.0 Hz), 132.6, 131.8, 131.0 (d, *J* = 9.3 Hz), 128.9, 127.0, 116.3, 116.0 (d, *J* = 21.8 Hz), 45.7, 36.6, 31.9, 31.7, 28.1, 27.0, 22.6, 20.0, 14.1.

^{19}F NMR (377 MHz, $CDCl_3$) δ -105.5.

IR ν (cm-1) 2953 (m), 2921 (m), 2899 (w), 2848 (w), 1695 (w), 1676 (s), 1670 (s), 1647 (s), 1623 (w), 1617 (w), 1580 (m), 1467 (w), 1456 (w), 1446 (w), 1369 (m), 1363 (w), 1011 (w), 1001 (w), 950 (w), 709 (s), 691 (m), 680 (w), 675 (w), 670 (w), 665 (w), 656 (w).

HRMS (ESI/QTOF) m/z: [M + Na]+ Calcd for $C_{25}H_{28}FNNaO_2^+$ 416.1996; Found 416.1997.

2-phenyl-1-(((2*E*,3*E*)-4-phenylbut-3-en-2-ylidene)amino)vinyl benzoate (11a)

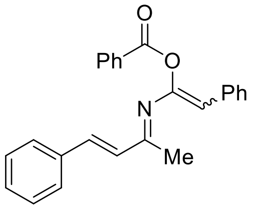

Yield: 95% yield (34.9 mg), pale oil as a 5:1 mixture of isomers, purified by column chromatography on silica gel (petroleum ether/ethyl acetate = 20/1).

^{1}H NMR (400 MHz, $CDCl_3$) δ ^{1}H NMR (400 MHz, $CDCl_3$) δ 8.19-8.11 (m, 2H), 7.64-7.57 (m, 1H), 7.56-7.42 (m, 4H), 7.42-7.25 (m, 8H), 7.20-7.09 (m, 1H), 7.05 (d, *J* = 16.4 Hz, 0.2H), 7.04 (d, *J* = 16.4 Hz, 0.8H), 5.96 (s, 0.2H), 5.94 (s, 0.8H), 2.46 (s, 0.6H), 2.21 (s, 2.4H).

^{13}C NMR (101 MHz, $CDCl_3$) major isomer: δ 171.0, 164.7, 147.9, 139.8, 135.7, 135.0, 133.7, 131.0, 130.3, 129.7, 129.5, 129.0, 128.7, 128.6, 128.4, 127.9, 126.6, 106.9, 18.5.

IR ν (cm-1) 1742 (m), 1701 (w), 1691 (w), 1685 (w), 1667 (w), 1655 (w), 1649 (w), 1430 (w), 1420 (w), 1416 (w), 1369 (w), 1357 (w), 1337 (w), 1327 (w), 1314 (w), 1293 (w), 1281 (w), 1260 (s), 910 (m), 882 (w), 856 (w), 798 (w), 787 (w), 749 (s), 730 (m), 705 (s), 689 (s), 654 (w).

HRMS (ESI/QTOF) m/z: [M + Na]+ Calcd for $C_{25}H_{21}NNaO_2^+$ 390.1464; Found 390.1464.

(*E*)-2-phenyl-N-(4-phenylbuta-1,3-dien-2-yl)acetamide (13a)

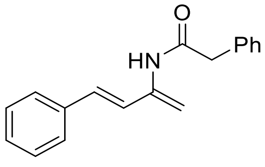

Yield: 81% yield (21.3 mg), pale oil as a single isomer, purified by column chromatography on silica gel (petroleum ether/ethyl acetate = 10/1).

^{1}H NMR (400 MHz, $CDCl_3$) δ 7.52-7.44 (m, 2H), 7.43-7.36 (m, 3H), 7.32-7.28 (m, 2H), 7.26-7.19 (m, 3H), 6.69 (s, 1H), 6.51 (d, *J* = 16.4 Hz, 1H), 5.88 (s, 1H), 5.87 (d, *J* = 16.4 Hz, 1H), 5.00 (s, 1H), 3.77 (s, 2H).

^{13}C NMR (126 MHz, $CDCl_3$) δ 169.5, 137.2, 136.0, 134.8, 130.0, 129.6, 128.9, 128.3, 128.1, 126.7, 126.7, 126.6, 107.3, 45.2.

IR ν (cm-1) 1695 (m), 1689 (m), 1684 (m), 1654 (s), 1647 (m), 1539 (m), 1533 (m), 1526 (s), 1507 (m), 1495 (m), 751 (m), 702 (m), 692 (m).

HRMS (ESI/QTOF) m/z: [M + Na]+ Calcd for $C_{18}H_{17}NNaO^+$ 286.1202; Found 286.1198.

N-((2*E*,4*E*)-nona-2,4-dien-2-yl)-N-(2-phenylacetyl)benzamide (14r)

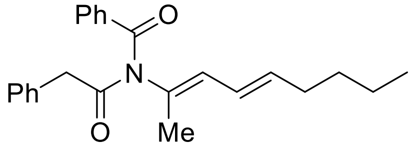

Yield: 46% yield (17.0 mg), pale oil as a single isomer, purified by column chromatography on silica gel (petroleum ether/ethyl acetate = 10/1).

^{1}H NMR (500 MHz, $CDCl_3$) δ 7.47-7.43 (m, 1H), 7.40-7.37 (m, 2H), 7.33-7.27 (m, 7H), 5.93-5.87 (m, 2H), 5.71-5.65 (m, 1H), 4.08 (s, 2H), 2.04 (q, *J* = 7.0 Hz, 2H), 1.83 (s, 3H), 1.35-1.27 (m, 4H), 0.89 (t, *J* = 7.0 Hz, 3H).

^{13}C NMR (126 MHz, $CDCl_3$) δ 174.5, 173.6, 138.8, 135.9, 134.5, 132.0, 131.4, 130.4, 129.9, 128.8, 128.2, 127.8, 127.4, 123.8, 43.9, 32.7, 31.4, 22.4, 21.8, 14.1.

IR ν (cm^{-1}) 2926 (s), 1705 (s), 1686 (s), 1281 (s), 1136 (m), 970(m), 695(s).

HRMS (ESI/QTOF) m/z: $[M + Na]^+$ Calcd for $C_{24}H_{27}NNaO_2^+$ 384.1934; Found 384.1935.

N-3-chloro-6-oxo-6a,7,8,10a-tetrahydro-6H-benzo[c]chromen-9-yl)-N-(2phenylacetyl)benzamide(16a)

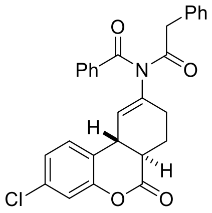

Yield: 45% yield (21.2 mg), pale oil as a single diastereoisomer, purified by column chromatography on silica gel (petroleum ether/ethyl acetate = 6/1).

^{1}H NMR (500 MHz, $CDCl_3$) δ7.44-7.41 (m, 3H), 7.35-7.29 (m, 4H), 7.29-7.26 (m, 3H), 7.06-7.02 (m, 2H), 6.80 (dd, *J* = 8.2, 1.2 Hz, 1H), 5.84 (d, *J* = 1.9 Hz, 1H), 4.10 (apparent s, 2H), 3.42 (dq, *J* = 13.5, 3.0 Hz, 1H), 2.45-2.40 (m, 1H), 2.36-2.31 (m, 2H), 2.19 (td, *J* = 12.8, 2.7 Hz, 1H), 1.77-1.69 (m, 1H).

^{13}C NMR (126 MHz, $CDCl_3$) δ 174.7, 173.0, 169.1, 151.7, 140.3, 135.2, 134.3, 134.0, 132.6, 129.6, 129.0, 128.7, 128.0, 127.6, 124.6, 124.6, 124.5, 124.0, 117.8, 44.3, 39.9, 35.2, 27.3, 22.1.

IR ν (cm^{-1}) 1789 (m), 1780 (m), 1764 (m), 1743 (w), 1714 (m), 1705 (m), 1686 (s), 1677 (m), 1671 (m), 1664 (m), 1407 (w), 1363 (w), 1289 (m), 1274 (m), 1152 (s), 1119 (s), 1097 (m), 1076 (m), 1065 (m), 1038 (w), 1024 (w), 974 (m), 948 (m), 932 (w), 909 (m), 862 (w).

HRMS (ESI/QTOF) m/z: $[M + Na]^+$ Calcd for $C_{28}H_{22}ClNNaO_4^+$ 494.1130; Found 494.1131.

N-3-bromo-6-oxo-6a,7,8,10a-tetrahydro-6H-benzo[c]chromen-9-yl)-N-(2-phenylacetyl)benzamide (16b)

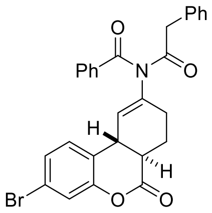

Yield: 46% yield (23.7 mg), pale oil as a single diastereoisomer, purified by column chromatography on silica gel (petroleum ether/ethyl acetate = 6/1).

The compound of **16b** was crystallized from MeOH/Pentane (v:v = 1:5) as a colorless crystal.

^{1}H NMR (500 MHz, $CDCl_3$) δ 7.44-7.39 (m, 3H), 7.33-7.29 (m, 4H), 7.29-7.26 (m, 3H), 7.21-7.16 (m, 2H), 6.74 (dd, *J* = 8.2, 1.3 Hz, 1H), 5.83 (apparent d, *J* = 2.0 Hz, 1H), 4.10 (s, 2H), 3.40 (dq, *J* = 13.3, 3.1 Hz, 1H), 2.45-2.39 (m, 1H), 2.36-2.31 (m, 2H), 2.18 (td, *J* = 12.7, 2.7 Hz, 1H), 1.73 (ddt, *J* = 12.9, 9.9, 8.0 Hz, 1H).

^{13}C NMR (126 MHz, $CDCl_3$) δ 174.7, 173.0, 169.1, 151.7, 140.3, 135.2, 134.3, 132.6, 129.6, 128.9, 128.7, 128.0, 127.6, 127.5, 124.8, 124.6, 124.5, 121.5, 120.6, 44.3, 39.8, 35.2, 27.3, 22.1.

IR ν (cm-1) 1790 (m), 1780 (s), 1764 (m), 1747 (w), 1705 (s), 1697 (s), 1687 (s), 1679 (s), 1577 (w), 1496 (w), 1484 (m), 1449 (w), 1403 (m), 1373 (w), 17 (w), 1301 (m), 1289 (m), 1174 (m), 1153 (m), 1120 (m), 944 (w), 911 (w), 795 (w), 729 (m), 710 (m), 696 (m).

HRMS (ESI/QTOF) m/z: [M + Na]+ Calcd for $C_{28}H_{22}BrNNaO_4^+$ 538.0624; Found 538.0627.

N-3-methoxy-6-oxo-6a,7,8,10a-tetrahydro-6H-benzo[c]chromen-9-yl)-N-(2-phenylacetyl)benzamide (16c)

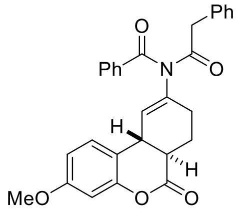

Yield: 40% yield (18.7 mg), pale oil as a single diastereoisomer, purified by column chromatography on silica gel (petroleum ether/ethyl acetate = 6/1).

^{1}H NMR (600 MHz, $CDCl_3$) δ 7.43-7.38 (m, 3H), 7.33-7.25 (m, 7H), 6.79 (d, *J* = 8.3 Hz, 1H), 6.61-6.57 (m, 2H), 5.88 (s, 1H), 4.10 (s, 2H), 3.77 (s, 3H), 3.39 (d, *J* = 13.3 Hz, 1H), 2.43-2.38 (m, 1H), 2.35-2.27 (m, 2H), 2.19 (t, *J* = 12.7 Hz, 1H), 1.75-1.68 (m, 1H).

^{13}C NMR (151 MHz, $CDCl_3$) δ 174.7, 173.1, 170.0, 159.9, 152.1, 139.7, 135.3, 134.4, 132.5, 129.6, 128.9, 128.6, 127.9, 127.5, 125.5, 124.1, 117.3, 110.1, 103.2, 55.7, 44.2, 40.4, 35.0, 27.4, 22.0.

IR ν (cm-1) 1762 (s), 1713 (m), 1686 (s), 1677 (s), 1665 (m), 1624 (m), 1508 (m), 1306 (m), 1290 (s), 1268 (s), 1242 (s), 1227 (s), 1202 (s), 1175 (m), 1159 (s), 1121 (s), 1070 (m), 721 (m), 713 (m), 705 (m), 696 (m).

HRMS (nanochip-ESI/LTQ-Orbitrap) m/z: [M + H]+ Calcd for $C_{29}H_{26}NO_5^+$ 468.1805; Found 468.1805.

N-3-methyl-6-oxo-6a,7,8,10a-tetrahydro-6H-benzo[c]chromen-9-yl)-N-(2-phenylacetyl)benzamide (16d)

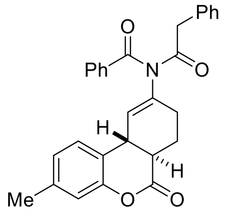

Yield: 45% yield (20.3 mg), pale oil as a single diastereoisomer, purified by column chromatography on silica gel (petroleum ether/ethyl acetate = 6/1).

^{1}H NMR (800 MHz, $CDCl_3$) δ 7.43-7.39 (m, 3H), 7.34-7.28 (m, 4H), 7.28-7.26 (m, 3H), 6.86 (dd, *J* = 7.9, 1.7 Hz, 1H), 6.85 (d, *J* = 1.7 Hz, 1H), 6.79 (dd, *J* = 7.7, 1.2 Hz, 1H), 5.91 (t, *J* = 2.3 Hz, 1H), 4.10 (s, 2H), 3.44-3.40 (m, 1H), 2.44-2.40 (m, 1H), 2.37-2.27 (m, 2H), 2.31 (s, 3H), 2.20 (td, *J* = 13.0, 2.7 Hz, 1H), 1.75-1.70 (m, 1H).

^{13}C NMR (201 MHz, $CDCl_3$) δ 174.7, 173.1, 170.2, 151.2, 139.7, 138.9, 135.3, 134.4, 132.5, 129.7, 128.9, 128.6, 128.0, 127.5, 125.4, 125.2, 123.3, 122.3, 117.8, 44.3, 40.2, 35.3, 27.5, 22.2, 21.2.

IR ν (cm-1) 1763 (m), 1751 (m), 1714 (m), 1677 (m), 1671 (m), 1664 (m), 1625 (w), 1507 (w), 1495 (w), 1413 (w), 1364 (w), 1343 (w), 1227 (s), 1086 (m), 1011 (w), 1002 (w), 943 (w), 933 (w), 909 (m), 842 (w), 821 (w), 799 (w), 790 (w), 775 (w), 768 (w), 719 (s), 663 (m).

HRMS (ESI/QTOF) m/z: [M + Na]+ Calcd for $C_{29}H_{25}NNaO_4^+$ 474.1676; Found 474.1668.

5. Copies of the SFC chromatograms

SFC chromotagram of compound 9a

IC, 20% MeOH in supercritical CO_2, v = 2 mL·min-1, λ = 270 nm.

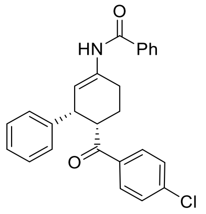

9a

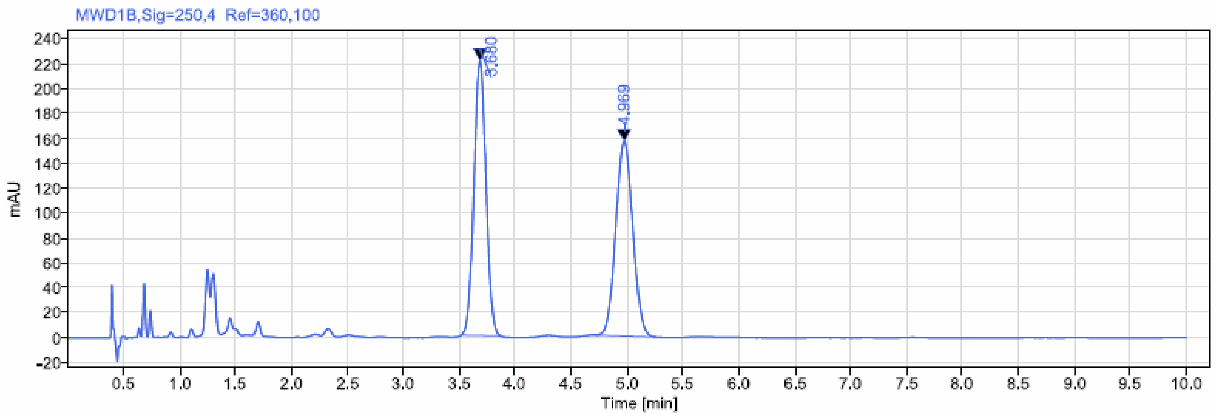

Signal: MWD1B,Sig=250,4 Ref=360,100

RT [min]	Type	Width [min]	Area	Height	Area%	Name
3,680	MM m	0,35	1663,97	220,79	50,09	
4,969	MM m	0,57	1658,29	156,27	49,91	
		**Sum**	**3322,26**			

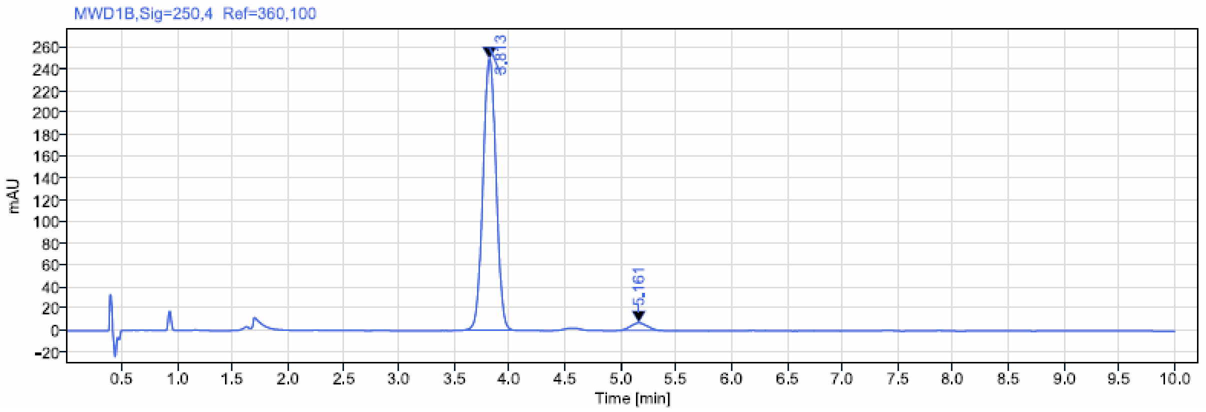

Signal: MWD1B,Sig=250,4 Ref=360,100

RT [min]	Type	Width [min]	Area	Height	Area%	Name
3,813	MM m	0,49	2026,82	249,45	96,52	
5,161	MM m	0,37	73,02	7,03	3,48	
		**Sum**	**2099,85**			

SFC chromotagram of compound 9b

IC, 20% MeOH in supercritical CO_2, v = 2 mL·min-1, λ = 270 nm.

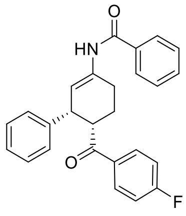

9b

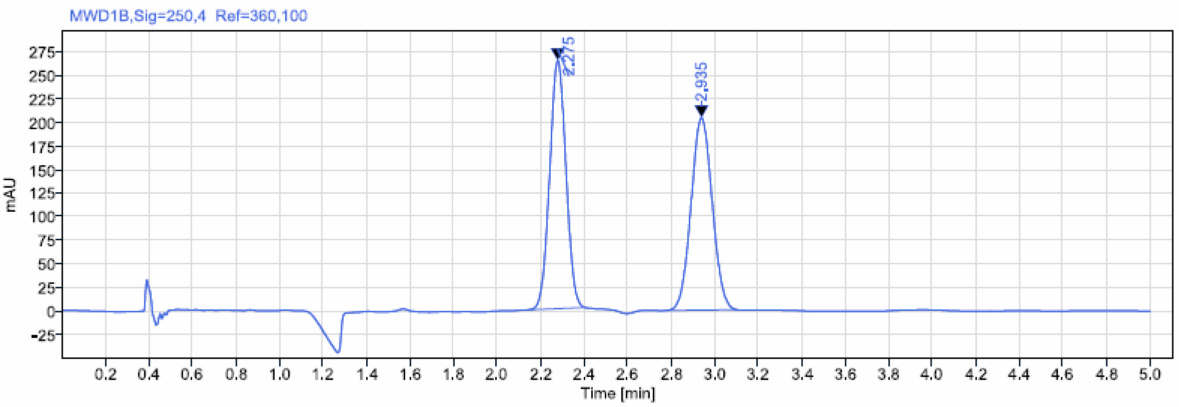

Signal: MWD1B,Sig=250,4 Ref=360,100

RT [min]	Type	Width [min]	Area	Height	Area%	Name
2,275	MM m	0,28	1374,16	263,39	49,88	
2,935	MM m	0,32	1380,95	204,05	50,12	
		**Sum**	**2755,11**			

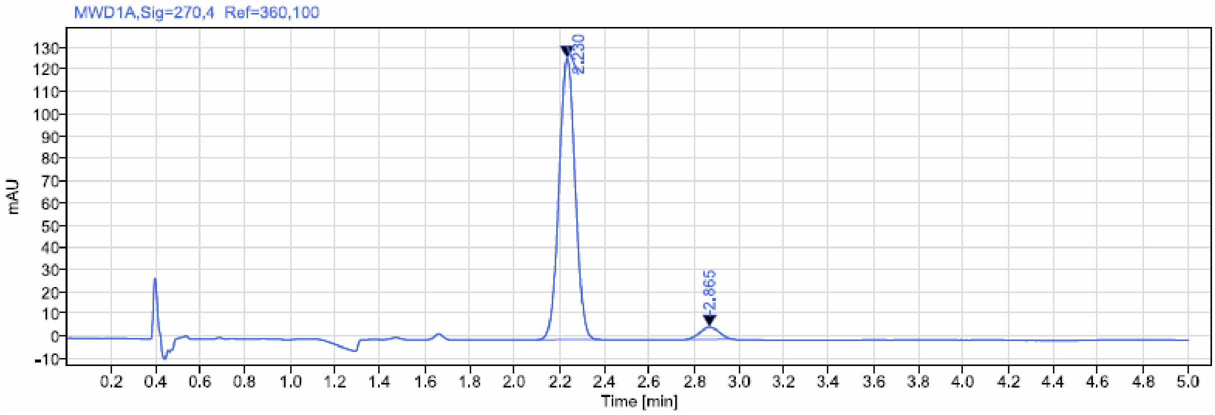

Signal: MWD1A,Sig=270,4 Ref=360,100

RT [min]	Type	Width [min]	Area	Height	Area%	Name
2,230	MM m	0,28	633,69	126,39	95,14	
2,865	MM m	0,21	32,40	5,39	4,86	
		**Sum**	**666,09**			

SFC chromotagram of compound 9c

IC, 20% MeOH in supercritical CO_2, v = 2 mL·min-1, λ = 270 nm.

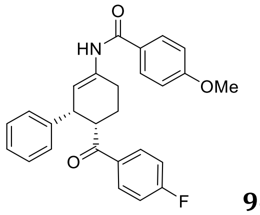

9c

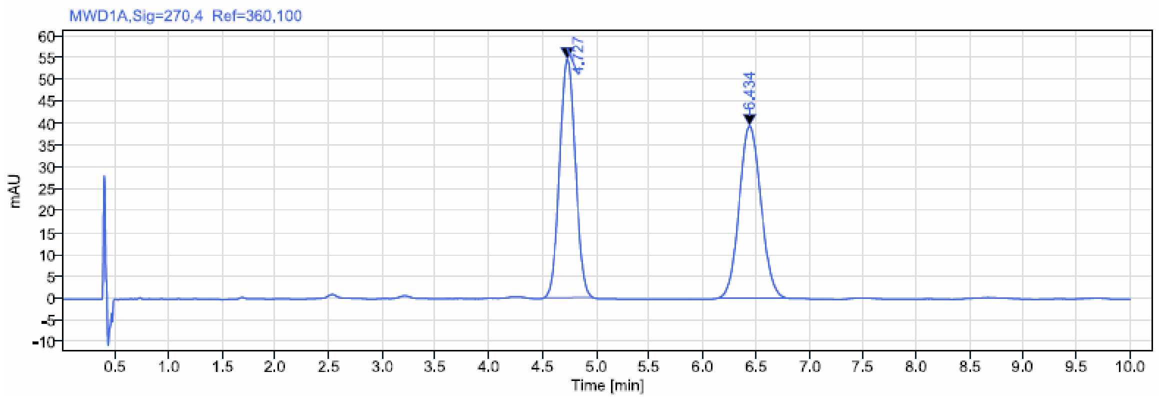

Signal:	MWD1A,Sig=270,4 Ref=360,100					
RT [min]	Type	Width [min]	Area	Height	Area%	Name
4,727	MM m	0,45	556,13	54,38	50,07	
6,434	MM m	0,65	554,60	39,28	49,93	
		**Sum**	**1110,72**			

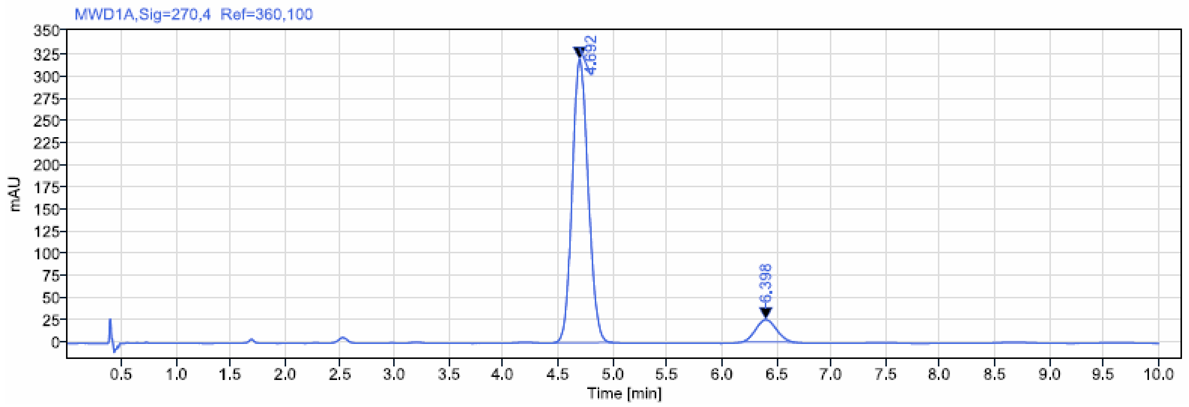

Signal:	MWD1A,Sig=270,4 Ref=360,100					
RT [min]	Type	Width [min]	Area	Height	Area%	Name
4,692	MM m	0,54	3254,13	318,20	90,77	
6,398	MM m	0,48	331,05	25,19	9,23	
		**Sum**	**3585,18**			

SFC chromotagram of compound 9d

IC, 20% MeOH in supercritical CO_2, v = 2 mL·min-1, λ = 270 nm.

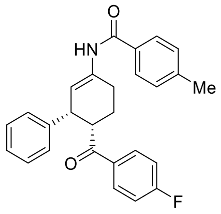

9d

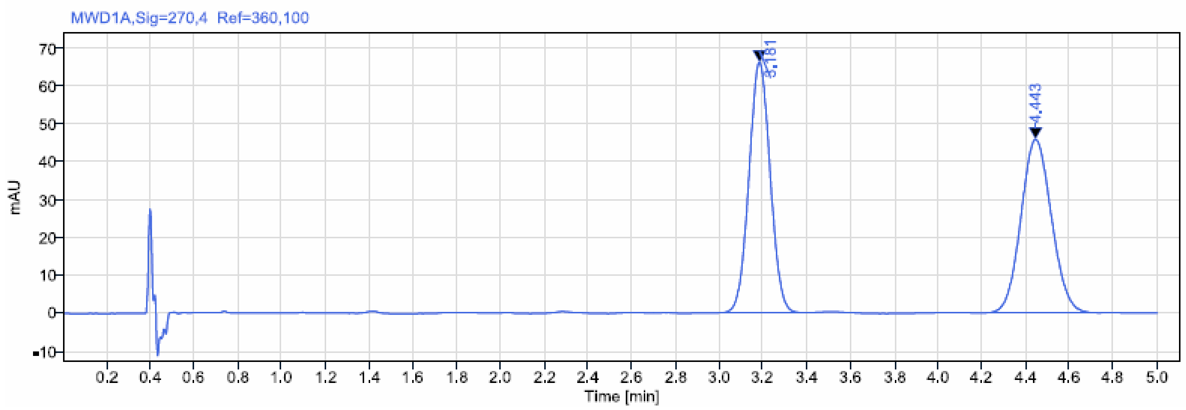

Signal:	MWD1A,Sig=270,4 Ref=360,100					
**RT [min]**	**Type**	**Width [min]**	**Area**	**Height**	**Area%**	**Name**
3,181	MM m	0,36	446,79	66,08	50,04	
4,443	MM m	0,48	446,08	45,92	49,96	
		**Sum**	**892,87**			

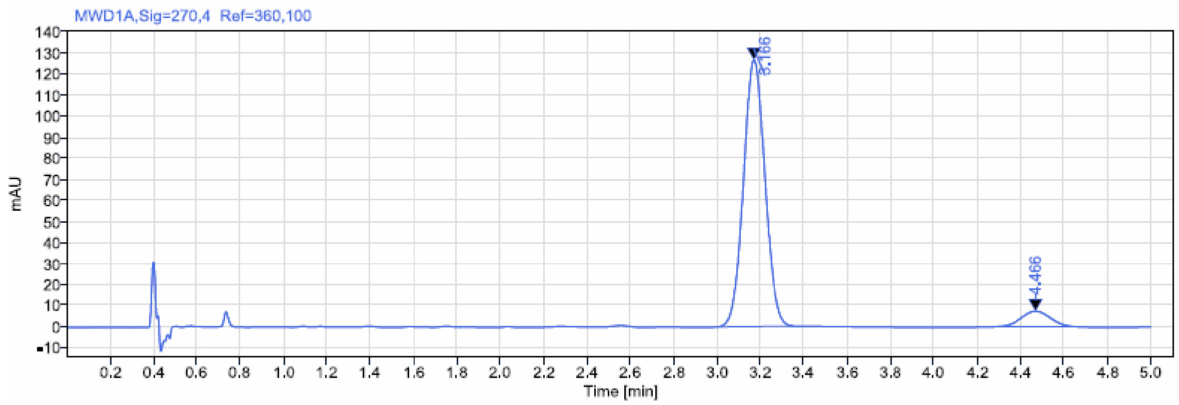

Signal:	MWD1A,Sig=270,4 Ref=360,100					
**RT [min]**	**Type**	**Width [min]**	**Area**	**Height**	**Area%**	**Name**
3,166	MM m	0,35	871,64	126,51	93,09	
4,466	MM m	0,33	64,74	7,15	6,91	
		**Sum**	**936,38**			

SFC chromotagram of compound 9e

IC, 20% MeOH in supercritical CO_2, v = 2 mL·min-1, λ = 270 nm.

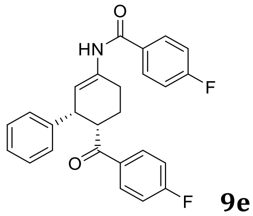

9e

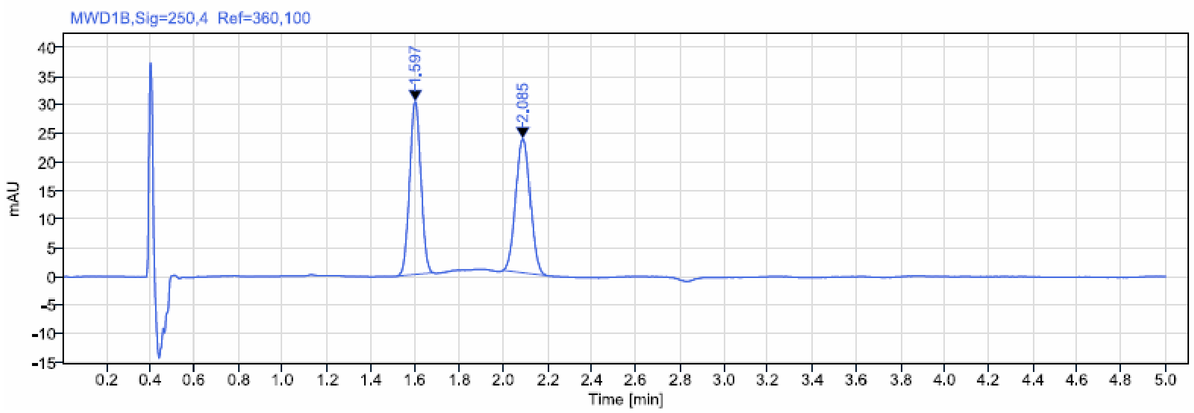

Signal: MWD1B,Sig=250,4 Ref=360,100

RT [min]	Type	Width [min]	Area	Height	Area%	Name
1,597	MM m	0,16	108,37	30,16	50,25	
2,085	MM m	0,20	107,31	23,37	49,75	
		**Sum**	**215,68**			

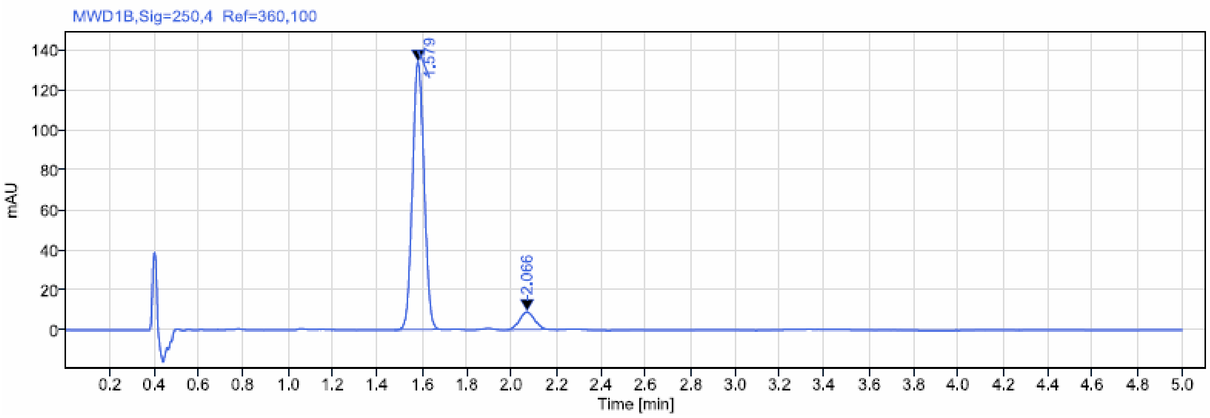

Signal: MWD1B,Sig=250,4 Ref=360,100

RT [min]	Type	Width [min]	Area	Height	Area%	Name
1,579	MM m	0,21	481,47	133,67	92,10	
2,066	MM m	0,18	41,30	8,93	7,90	
		**Sum**	**522,77**			

SFC chromotagram of compound 9f

IC, 20% MeOH in supercritical CO_2, v = 2 mL·min-1, λ = 270 nm.

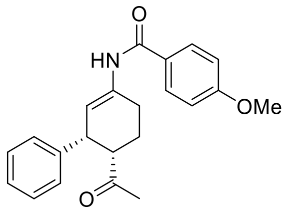

9f

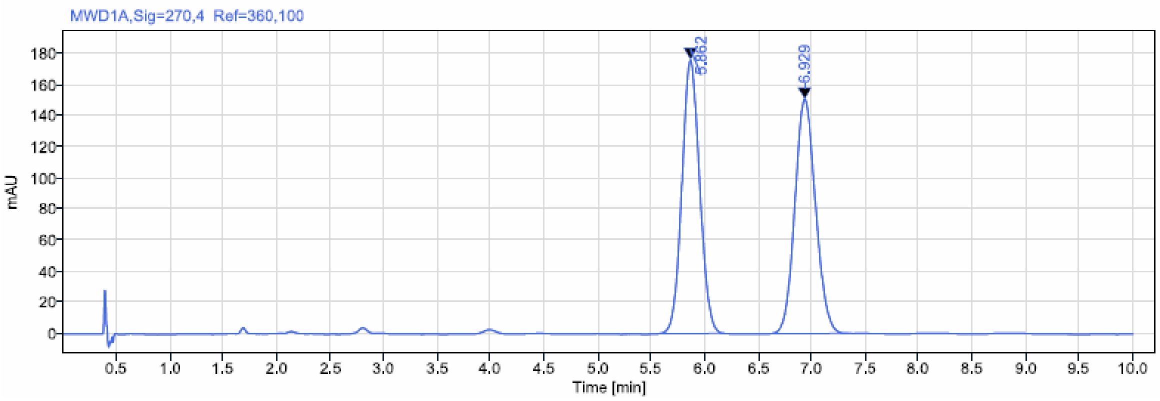

Signal: MWD1A,Sig=270,4 Ref=360,100

RT [min]	Type	Width [min]	Area	Height	Area%	Name
5,862	MM m	0,61	2040,21	175,79	50,15	
6,929	MM m	0,71	2027,90	150,25	49,85	
		Sum	4068,11			

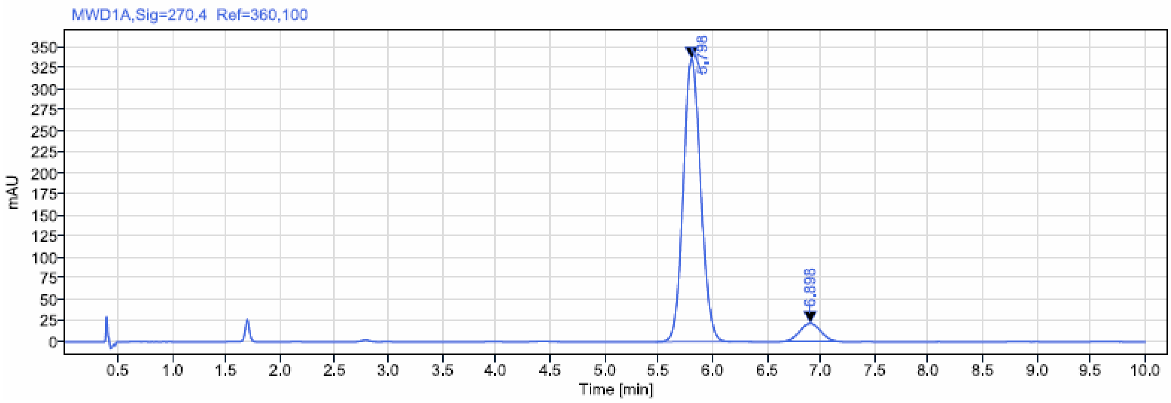

Signal: MWD1A,Sig=270,4 Ref=360,100

RT [min]	Type	Width [min]	Area	Height	Area%	Name
5,798	MM m	0,70	3812,22	335,72	93,54	
6,898	MM m	0,44	263,44	20,57	6,46	
		Sum	4075,66			

SFC chromotagram of compound 9g

IC, 20% MeOH in supercritical CO_2, v = 2 mL·min-1, λ = 270 nm.

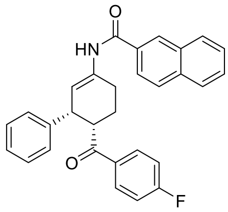

9g

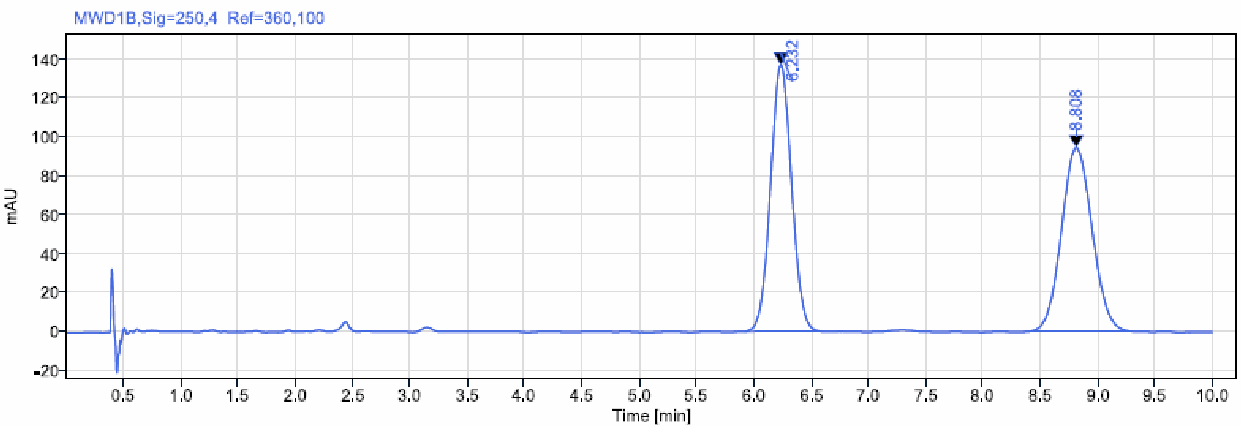

Signal:	MWD1B,Sig=250,4 Ref=360,100					
RT [min]	Type	Width [min]	Area	Height	Area%	Name
6,232	MM m	0,65	1745,79	136,76	50,05	
8,808	MM m	0,84	1742,15	94,00	49,95	
		**Sum**	**3487,94**			

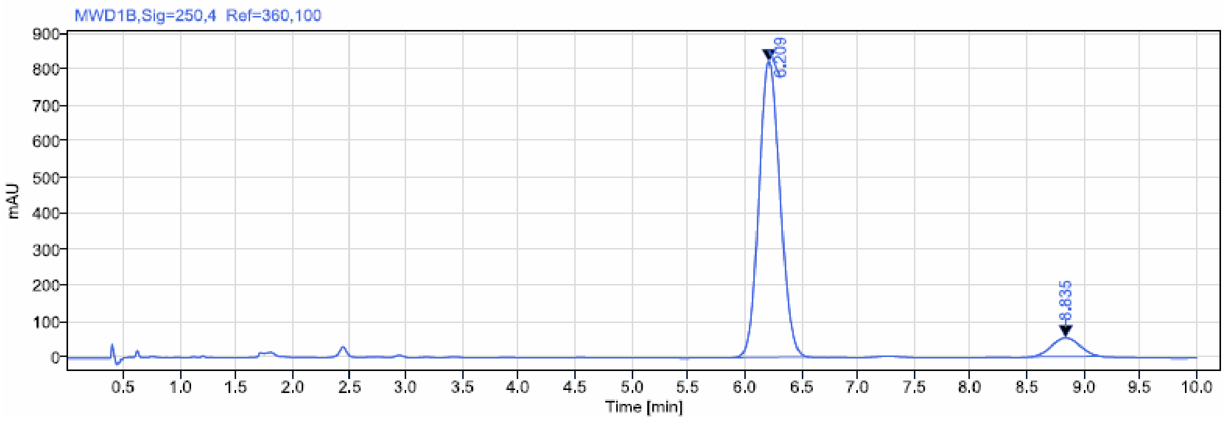

Signal:	MWD1B,Sig=250,4 Ref=360,100					
RT [min]	Type	Width [min]	Area	Height	Area%	Name
6,209	MM m	0,70	10702,56	820,01	92,17	
8,835	MM m	0,61	909,70	52,23	7,83	
		**Sum**	**11612,26**			

SFC chromotagram of compound 9h

IC, 20% MeOH in supercritical CO_2, v = 2 mL·min-1, λ = 270 nm.

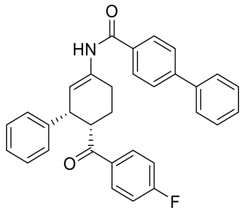

9h

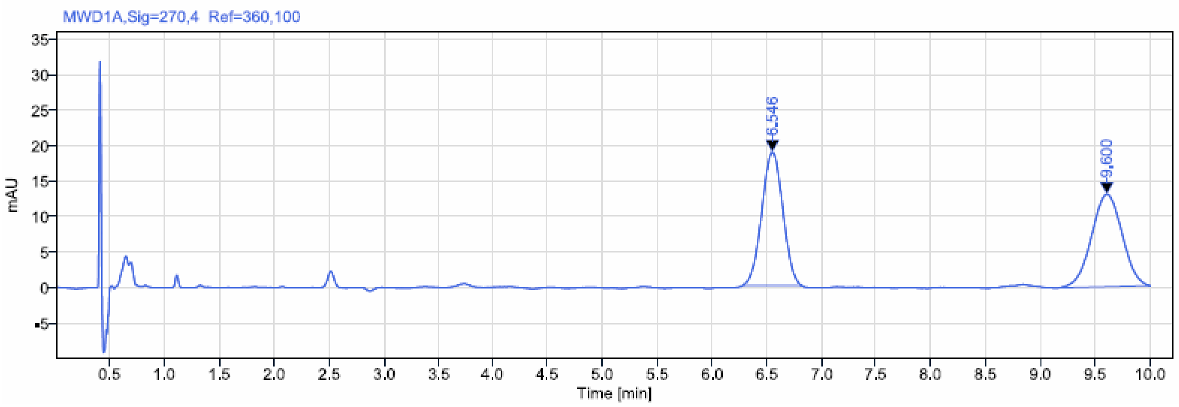

Signal: MWD1A,Sig=270,4 Ref=360,100

RT [min]	Type	Width [min]	Area	Height	Area%	Name
6,546	MM m	0,54	250,24	18,80	49,84	
9,600	MM m	0,83	251,80	12,98	50,16	
		Sum	502,03			

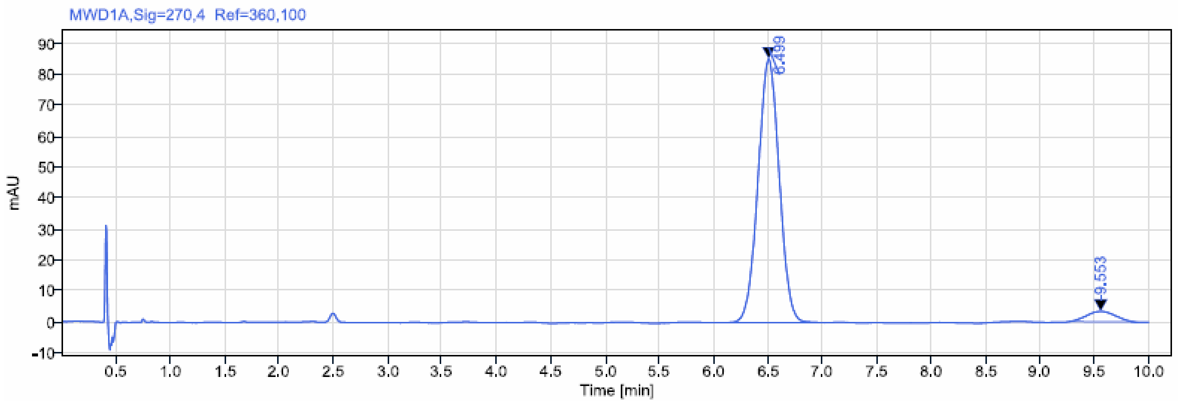

Signal: MWD1A,Sig=270,4 Ref=360,100

RT [min]	Type	Width [min]	Area	Height	Area%	Name
6,499	MM m	0,81	1175,18	85,10	94,92	
9,553	MM m	0,63	62,93	3,40	5,08	
		Sum	1238,11			

SFC chromotagram of compound 9i

IC, 20% MeOH in supercritical CO_2, v = 2 mL·min-1, λ = 270 nm.

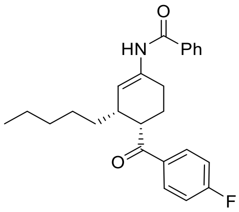

9i

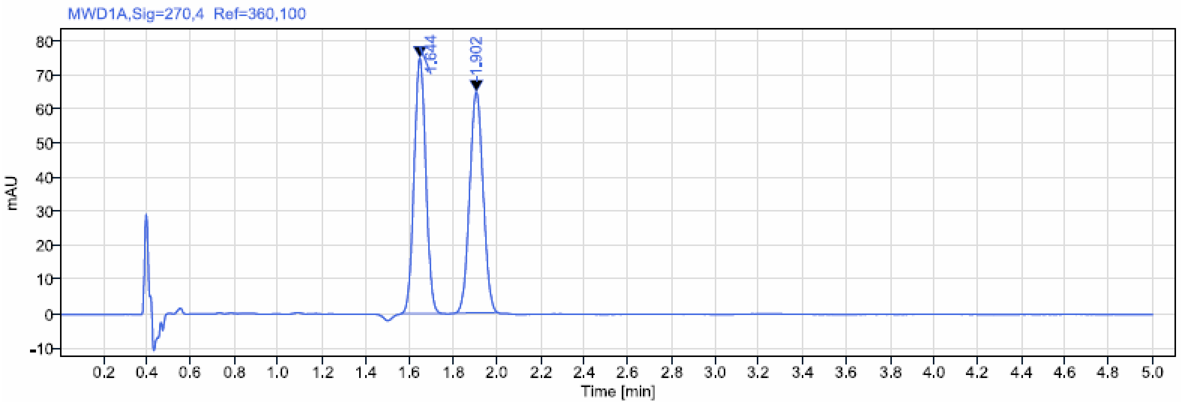

Signal: MWD1A,Sig=270,4 Ref=360,100

RT [min]	Type	Width [min]	Area	Height	Area%	Name
1,644	MM m	0,18	279,56	74,89	50,20	
1,902	MM m	0,19	277,36	64,70	49,80	
		**Sum**	**556,93**			

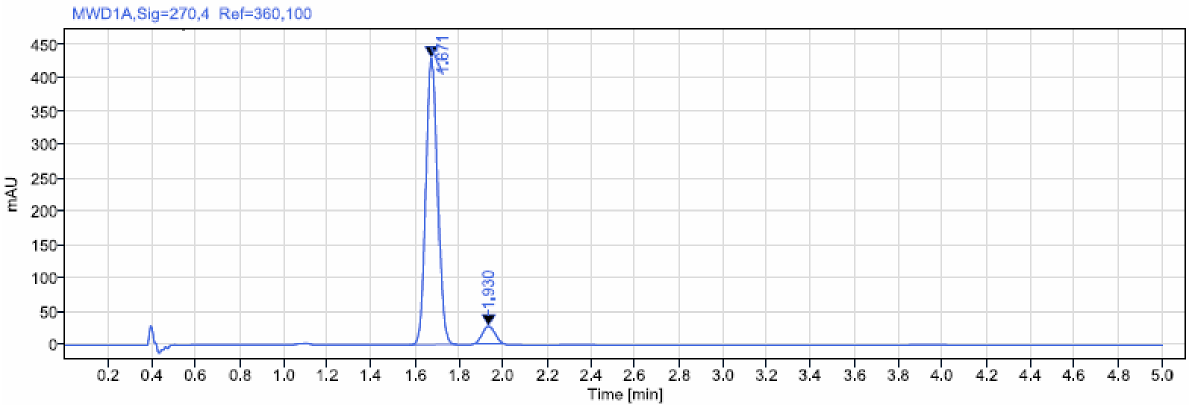

Signal: MWD1A,Sig=270,4 Ref=360,100

RT [min]	Type	Width [min]	Area	Height	Area%	Name
1,671	MM m	0,21	1592,97	429,63	94,06	
1,930	MM m	0,13	100,53	25,94	5,94	
		**Sum**	**1693,50**			

6. Crystallographic data

R_1=4.01%

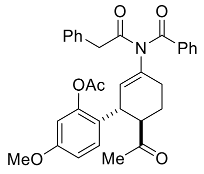

7k

Crystal Data and Experimental of **7k** (CCDC number **2260903**).

The ellipsoids are displayed at 50% probability level.

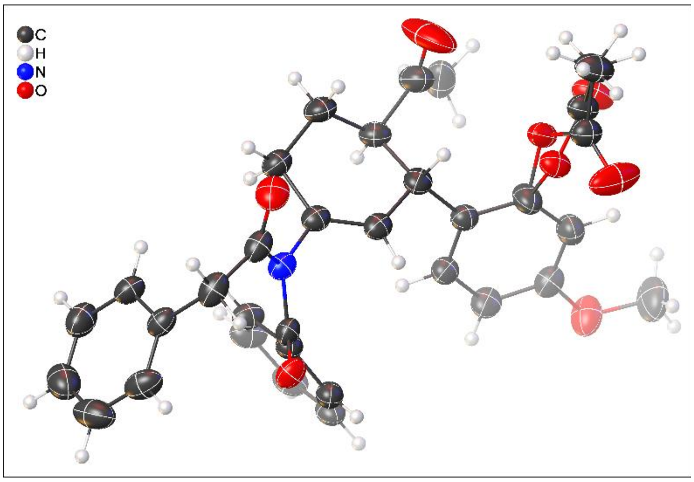

**Compound**	**7k**
Formula	$C_{32}H_{31}NO_6$
$D_{calc.}$/ g cm^{-3}	1.192
μ/mm^{-1}	0.669
Formula Weight	525.58
Colour	colourless
Shape	needle-shaped
Size/mm^3	0.66×0.03×0.03
T/K	140.00(10)
Crystal System	orthorhombic
Space Group	*Pccn*
a/Å	32.0562(6)
b/Å	22.3858(4)
c/Å	8.15990(12)
α/°	90
β/°	90
γ/°	90
V/Å^3	5855.57(16)
Z	8
Z'	1
Wavelength/Å	1.54184
Radiation type	Cu$K\alpha$
Θ_{min}/°	2.407
Θ_{max}/°	74.921
Measured Refl's.	41291
Indep't Refl's	5868
Refl's I≥2σ(I)	4780
R_{int}	0.0264
Parameters	383
Restraints	91
Largest Peak/e Å^{-3}	0.245
Deepest Hole/e Å^{-3}	-0.236
GooF	1.031
wR_2 (all data)	0.1079
wR_2	0.1014
R_1 (all data)	0.0507
R_1	0.0401
CCDC number	2260903

Experimental. The compound of **7k** was crystallized from Ethyl acetate/Pentane (v:v = 1:4) as a colorless crystal**.** A suitable crystal with dimensions 0.66 × 0.03 × 0.03 mm^3 was selected and mounted on an XtaLAB Synergy R, DW system, HyPix-Arc 150 diffractometer. The crystal was kept at a steady T = 140.00(10) K during data collection. The structure was solved with the ShelXT 2018/2 (Sheldrick, 2015) solution program using dual methods and by using Olex2 1.5 (Dolomanov et al., 2009) as the graphical interface. The model was refined with ShelXL 2019/3 (Sheldrick, 2015) using full matrix least squares minimisation on $\boldsymbol{F^2}$.[9-10]

Crystal Data. $C_{32}H_{31}NO_6$, M_r = 525.58, orthorhombic, *Pccn* (No. 56), a = 32.0562(6) Å, b = 22.3858(4) Å, c = 8.15990(12) Å, $\alpha = \beta = \gamma$ = 90°, V = 5855.57(16) Å^3, T = 140.00(10) K, Z = 8, Z' = 1, μ(Cu Kα) = 0.669, 41291 reflections measured, 5868 unique (R_{int} = 0.0264) which were used in all calculations. The final wR_2 was 0.1079 (all data) and R_1 was 0.0401 (I≥2σ(I)).

Structure Quality Indicators

**Reflections:**	d min (Cu\a) 2Θ=149.8° 0.80	I/σ(I) CIF 54.9	Rint m=7.58 2.64%	Full 135.4° 98% to 149.8° 99.9
**Refinement:**	Shift CIF 0.001	Max Peak CIF 0.2	Min Peak CIF -0.2	GooF CIF 1.031

A colourless needle-shaped crystal with dimensions 0.66 × 0.03 × 0.03 mm^3 was mounted. Data were collected using an XtaLAB Synergy R, DW system, HyPix-Arc 150 diffractometer operating at T = 140.00(10) K.

Data were measured using ω scans with Cu K_α radiation. The diffraction pattern was indexed and the total number of runs and images was based on the strategy calculation from the program CrysAlisPro 1.171.42.90a (Rigaku OD, 2023). The maximum resolution achieved was Θ = 74.921° (0.80 Å).

The unit cell was refined using CrysAlisPro 1.171.42.90a (Rigaku OD, 2023) on 15647 reflections, 38% of the observed reflections.

Data reduction, scaling and absorption corrections were performed using CrysAlisPro 1.171.42.90a (Rigaku OD, 2023). The final completeness is 99.90 % out to 74.921° in Θ. A Gaussian absorption correction was performed using CrysAlisPro 1.171.42.90a (Rigaku Oxford Diffraction, 2023) Numerical absorption correction based on Gaussian integration over a multifaceted crystal model. Empirical absorption correction using spherical harmonics as implemented in SCALE3 ABSPACK scaling algorithm. The absorption coefficient μ of this material is 0.669 mm^{-1} at this wavelength (λ = 1.54184Å) and the minimum and maximum transmissions are 0.630 and 1.000.

The structure was solved in the space group *Pccn* (# 56) by the ShelXT 2018/2 (Sheldrick, 2015) structure solution program using dual methods and refined by full matrix least squares minimisation on $\boldsymbol{F}^2$ using version 2019/3 of ShelXL 2019/3 (Sheldrick, 2015). All non-hydrogen atoms were refined anisotropically. Hydrogen atom positions were calculated geometrically and refined using the riding model.

There is a single formula unit in the asymmetric unit, which is represented by the reported sum formula. In other words: Z is 8 and Z' is 1. The moiety formula is $C_{32}H_{31}NO_6$, 0.5[C_5H_{12}].

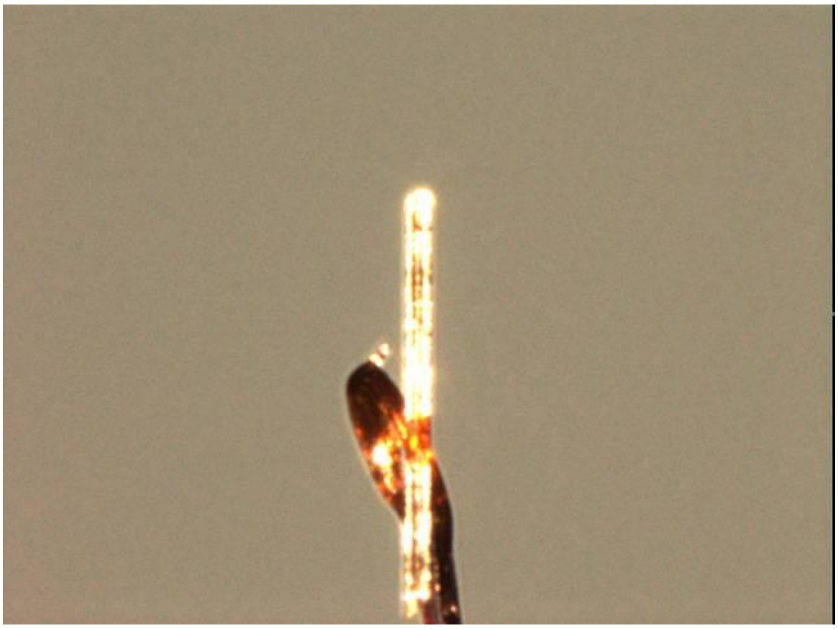

Supplementary Figure 10: Image of the Crystal on the Diffractometer.

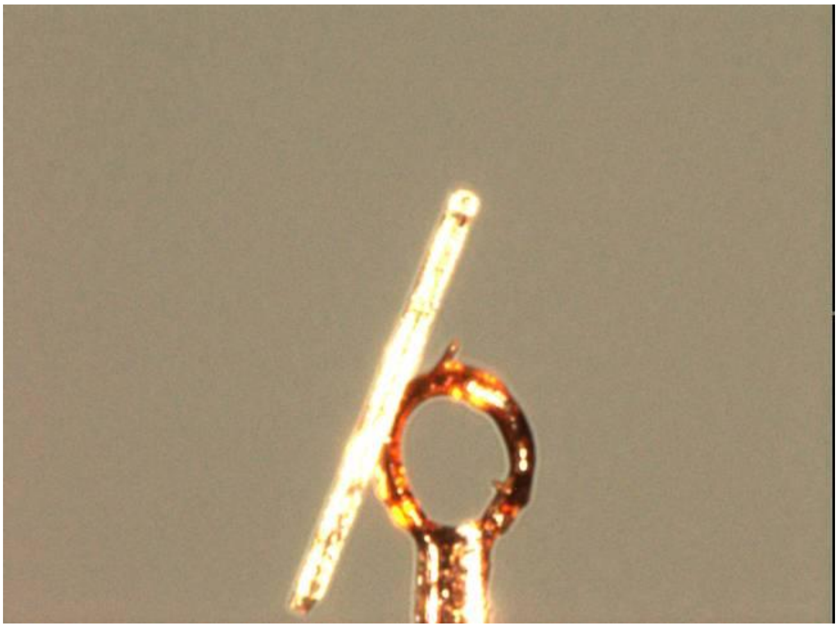

Supplementary Figure 11: Image of the Crystal on the Diffractometer.

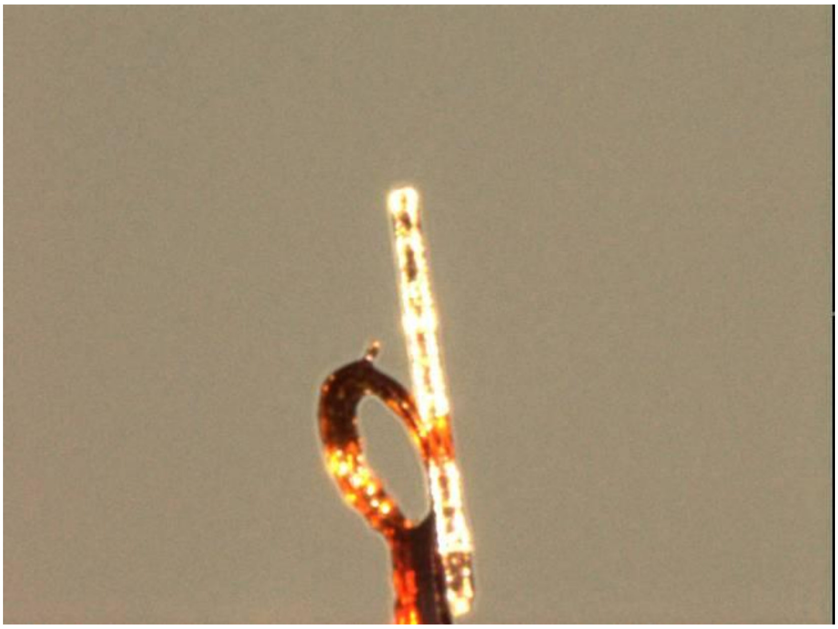

Supplementary Figure 12: Image of the Crystal on the Diffractometer.

Data Plots: Diffraction Data

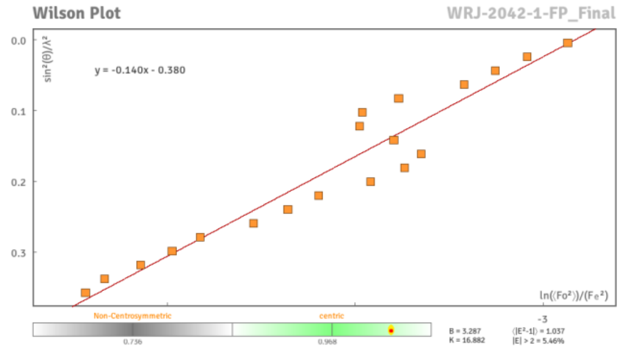

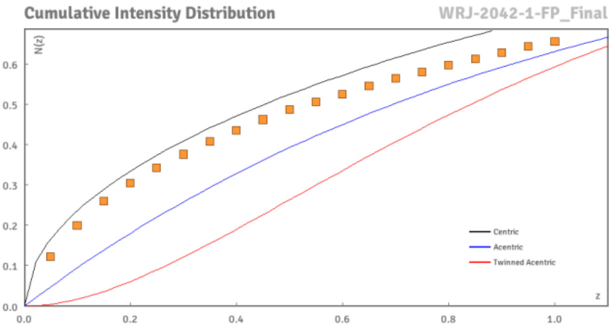

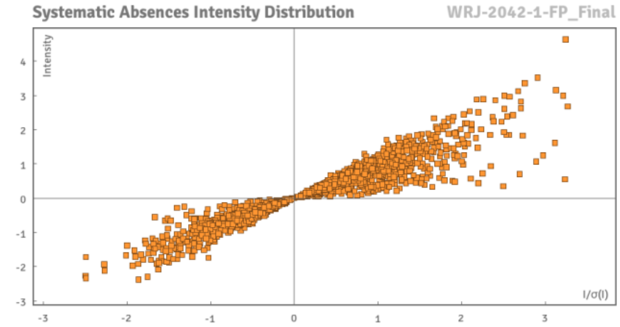

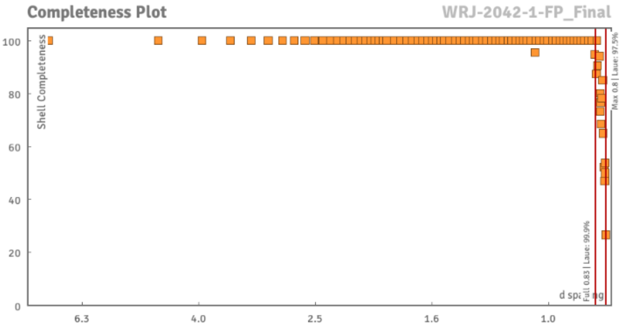

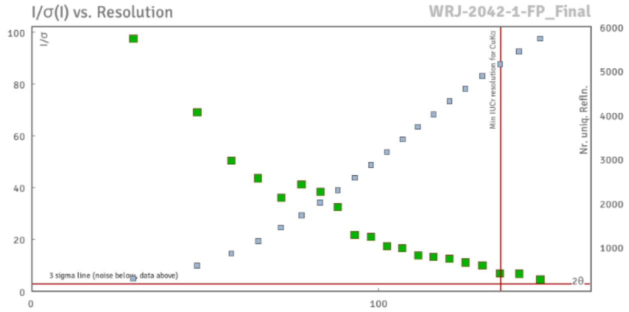

Data Plots: Refinement and Data

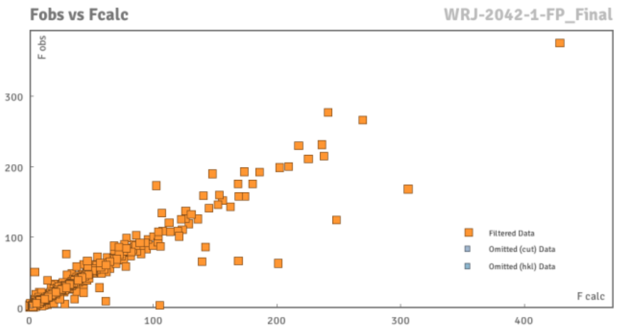

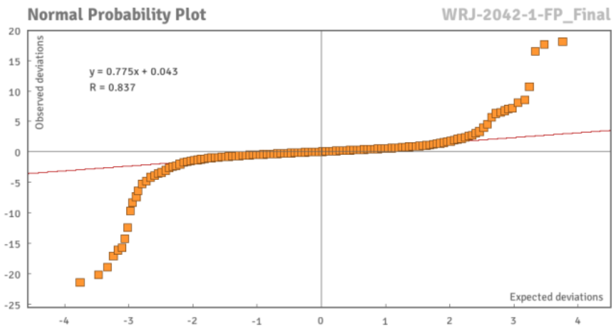

Reflection Statistics

Total reflections (after filtering)	44459	Unique reflections	5868
Completeness	0.975	Mean I/σ	28.84
hkl_{max} collected	(38, 28, 7)	hkl_{min} collected	(-39, -25, -10)
hkl_{max} used	(39, 28, 10)	hkl_{min} used	(0, 0, 0)
Lim d_{max} collected	100.0	Lim d_{min} collected	0.77
d_{max} used	18.35	d_{min} used	0.8
Friedel pairs	5952	Friedel pairs merged	1
Inconsistent equivalents	2	R_{int}	0.0264
R_{sigma}	0.0182	Intensity transformed	0
Omitted reflections	0	Omitted by user (OMIT hkl)	0
Multiplicity	(14591, 6850, 2556, 931, 397, 192, 94, 49, 40, 14, 7, 1)	Maximum multiplicity	35
Removed systematic absences	3168	Filtered off (Shel/OMIT)	0

Table S8: Fractional Atomic Coordinates (×10^4) and Equivalent Isotropic Displacement Parameters (Å^2×10^3) for **7k.** U_{eq} is defined as 1/3 of the trace of the orthogonalised U_{ij}.

Atom	x	y	z	U_{eq}
O1	3345.8(5)	7283.7(8)	3096(2)	110.1(6)
O2A	3129.7(6)	5929.4(7)	2571(2)	45.2(5)
O2B	3315.2(8)	5630.7(11)	2008(3)	37.5(6)
O3A	3239.7(9)	5199.6(8)	782(2)	82.0(8)
O3B	2660.4(8)	5985.5(13)	2124(3)	56.5(8)
O4	2814.3(4)	4328.3(5)	6311.9(13)	59.1(3)
O5	5377.7(3)	5372.4(4)	5915.3(11)	40.2(2)
O6	5238.5(3)	6845.6(5)	3310.4(12)	51.8(3)
N1	5031.9(3)	6249.8(4)	5399.7(12)	33.8(2)
C1	3755.9(4)	6807.4(6)	5118.7(17)	41.0(3)
C2	3879.8(4)	6215.7(6)	4250.6(15)	36.9(3)
C3	4332.2(4)	6067.4(6)	4533.9(14)	34.0(3)
C4	4607.7(4)	6449.1(5)	5139.8(15)	34.2(3)
C5	4508.9(4)	7081.0(6)	5612.3(18)	41.9(3)
C6	4095.2(5)	7277.8(6)	4873.9(19)	44.8(3)
C7	3345.1(5)	7025.9(7)	4403(2)	57.2(4)
C8	2953.6(5)	6912.0(8)	5312(2)	61.0(4)
C9	3599.1(4)	5699.1(5)	4746.6(15)	34.5(3)
C10	3264.3(5)	5517.4(7)	3808.6(18)	50.8(4)
C11	2993.6(4)	5063.0(6)	4257.1(18)	45.6(3)
C12	3059.4(4)	4777.6(6)	5727.7(16)	40.0(3)
C13	3395.2(5)	4944.2(6)	6705.2(16)	43.4(3)
C14	3657.8(4)	5397.4(6)	6212.9(15)	36.4(3)
C15A	3161.1(9)	5707.8(11)	1027(3)	41.7(5)
C15B	2976.0(14)	5921.2(17)	1383(5)	41.9(8)

Atom	x	y	z	U_{eq}
C16	3086.1(6)	6160.3(8)	-266.1(19)	57.6(4)
C17	2492.5(7)	4111.6(9)	5263(2)	75.9(6)
C18	5103.1(4)	5717.1(5)	6297.7(15)	32.3(3)
C19	4825.8(4)	5610.8(5)	7730.1(15)	32.5(3)
C20	4694.4(4)	5028.4(6)	8047.3(16)	37.0(3)
C21	4447.9(4)	4912.0(6)	9400.3(16)	42.7(3)
C22	4340.1(5)	5366.3(7)	10460.5(17)	46.3(3)
C23	4480.7(5)	5942.4(7)	10184.9(17)	46.8(3)
C24	4722.0(4)	6066.0(6)	8813.7(15)	39.4(3)
C25	5342.3(4)	6515.0(6)	4415.6(16)	39.4(3)
C26	5793.9(4)	6405.2(6)	4819.2(17)	43.2(3)
C27	5913.7(4)	6434.2(5)	6613.4(17)	37.8(3)
C28	6208.3(5)	6042.6(6)	7244(2)	52.7(4)
C29	6331.9(5)	6079.3(8)	8869(3)	67.1(5)
C30	6156.9(6)	6498.4(8)	9888(2)	64.1(5)
C31	5861.0(6)	6885.3(7)	9287.2(19)	54.1(4)
C32	5742.6(4)	6858.1(6)	7660.5(17)	41.6(3)

Table S9: Anisotropic Displacement Parameters (×10[4]) for **7k**. The anisotropic displacement factor exponent takes the form: $-2\pi^2[h^2a^{*2} \times U_{11} + \ldots + 2hka^* \times b^* \times U_{12}]$

Atom	U_{11}	U_{22}	U_{33}	U_{23}	U_{13}	U_{12}
O1	74.3(9)	131.9(14)	124.3(13)	85.6(12)	-28.5(9)	-0.5(9)
O2A	62.2(10)	34.7(8)	38.7(9)	-4.2(7)	-16.0(8)	12.4(7)
O2B	43.8(13)	43.2(13)	25.6(12)	3.3(10)	2.5(10)	6.5(11)
O3A	158(2)	46.7(11)	41.5(10)	0.8(8)	22.3(12)	28.0(12)
O3B	51.6(16)	74.1(18)	44.0(15)	9.0(13)	4.0(12)	24.5(13)
O4	66.0(7)	63.7(7)	47.5(6)	5.6(5)	5.0(5)	-29.2(6)
O5	42.8(5)	31.9(4)	45.9(5)	-7.1(4)	9.7(4)	-0.6(4)
O6	63.2(7)	53.5(6)	38.9(5)	11.6(5)	6.6(5)	-14.4(5)
N1	39.2(6)	31.2(5)	30.9(5)	1.5(4)	7.2(4)	-3.3(4)
C1	45.8(8)	36.5(7)	40.7(7)	9.5(6)	0.3(6)	3.9(6)
C2	45.6(7)	37.5(6)	27.6(6)	6.7(5)	-1.2(5)	-2.3(5)
C3	44.1(7)	33.4(6)	24.4(6)	3.0(5)	4.5(5)	-1.2(5)
C4	43.1(7)	32.3(6)	27.1(6)	4.7(5)	5.2(5)	-0.5(5)
C5	49.1(8)	31.0(6)	45.6(8)	0.3(6)	1.3(6)	-1.6(5)
C6	53.8(8)	31.4(6)	49.3(8)	8.9(6)	0.0(6)	1.0(6)
C7	54.6(9)	43.1(8)	74.1(11)	13.9(8)	-12.4(8)	3.5(7)
C8	45.3(8)	56.9(9)	80.9(12)	-21.7(9)	-8.6(8)	6.2(7)
C9	39.3(7)	34.0(6)	30.3(6)	2.0(5)	1.2(5)	1.5(5)
C10	56.0(9)	53.3(8)	43.1(8)	15.1(7)	-14.5(7)	-11.0(7)
C11	42.4(8)	47.4(8)	47.0(8)	3.2(7)	-7.3(6)	-5.9(6)
C12	43.2(7)	38.7(7)	38.1(7)	-2.6(6)	10.5(6)	-4.9(6)
C13	57.5(8)	44.5(7)	28.4(6)	6.1(6)	2.6(6)	-7.1(6)
C14	43.2(7)	36.6(6)	29.3(6)	1.5(5)	0.0(5)	-1.4(5)
C15A	49.4(13)	39.7(12)	36.0(12)	-0.5(10)	2.8(11)	5.8(10)
C15B	53.0(17)	39.2(16)	33.5(16)	-1.9(13)	-0.7(15)	10.1(14)
C16	72.7(10)	57.7(9)	42.3(8)	9.2(7)	-3.6(7)	18.1(8)
C17	73.0(12)	79.4(12)	75.2(12)	7.8(10)	-7.3(10)	-40.0(11)
C18	37.1(6)	28.9(6)	30.8(6)	-3.5(5)	2.3(5)	-4.4(5)
C19	35.3(6)	33.3(6)	28.9(6)	3.3(5)	1.0(5)	0.4(5)
C20	41.2(7)	35.7(6)	34.2(7)	4.1(5)	-3.4(5)	-3.1(5)
C21	44.4(7)	46.6(7)	37.1(7)	13.8(6)	-3.7(6)	-8.2(6)
C22	45.8(8)	61.0(9)	32.1(7)	14.1(6)	5.8(6)	2.8(7)
C23	58.4(9)	51.0(8)	31.1(7)	4.8(6)	9.6(6)	13.0(7)
C24	50.7(8)	36.2(6)	31.3(6)	4.5(5)	5.9(6)	5.6(6)
C25	51.3(8)	35.3(6)	31.7(6)	-3.4(5)	10.6(6)	-10.5(6)
C26	44.3(7)	40.5(7)	44.9(8)	-7.0(6)	17.4(6)	-8.5(6)
C27	35.4(7)	30.0(6)	47.9(8)	-1.6(5)	10.3(6)	-4.4(5)

Atom	U_{11}	U_{22}	U_{33}	U_{23}	U_{13}	U_{12}
C28	42.5(8)	40.3(7)	75.2(11)	6.5(7)	16.1(8)	5.2(6)
C29	48.2(9)	66.1(11)	87.0(13)	31.8(10)	-4.0(9)	2.3(8)
C30	70.5(11)	66.2(11)	55.7(10)	16.6(9)	-11.8(9)	-17.4(9)
C31	73.1(11)	43.2(8)	46.1(8)	-5.6(7)	0.9(8)	-7.6(7)
C32	49.4(8)	30.4(6)	45.1(8)	-1.8(6)	3.8(6)	1.4(5)

Table S10: Bond Lengths in Å for **7k**.

Atom	Atom	Length/Å
O1	C7	1.213(2)
O2A	C10	1.434(2)
O2A	C15A	1.358(3)
O2B	C10	1.500(3)
O2B	C15B	1.366(5)
O3A	C15A	1.182(3)
O3B	C15B	1.187(5)
O4	C12	1.3624(16)
O4	C17	1.426(2)
O5	C18	1.2116(15)
O6	C25	1.2131(17)
N1	C4	1.4468(17)
N1	C18	1.4181(16)
N1	C25	1.4098(16)
C1	C2	1.5536(18)
C1	C6	1.5267(19)
C1	C7	1.521(2)
C2	C3	1.5057(19)
C2	C9	1.5202(17)
C3	C4	1.3245(18)
C4	C5	1.5001(18)
C5	C6	1.522(2)
C7	C8	1.480(2)

Atom	Atom	Length/Å
C9	C10	1.3793(19)
C9	C14	1.3869(17)
C10	C11	1.386(2)
C11	C12	1.376(2)
C12	C13	1.391(2)
C13	C14	1.3781(18)
C15A	C16	1.482(3)
C15B	C16	1.491(4)
C18	C19	1.4875(17)
C19	C20	1.3944(17)
C19	C24	1.3897(18)
C20	C21	1.3825(18)
C21	C22	1.379(2)
C22	C23	1.385(2)
C23	C24	1.3881(18)
C25	C26	1.505(2)
C26	C27	1.515(2)
C27	C28	1.388(2)
C27	C32	1.3896(18)
C28	C29	1.387(3)
C29	C30	1.373(3)
C30	C31	1.375(2)
C31	C32	1.382(2)

Table S11: Bond Angles in for **7k**.

Atom	Atom	Atom	Angle/°
C15A	O2A	C10	113.34(18)
C15B	O2B	C10	111.1(2)
C12	O4	C17	117.28(12)
C18	N1	C4	119.09(10)
C25	N1	C4	116.75(10)
C25	N1	C18	122.34(11)
C6	C1	C2	110.26(11)
C7	C1	C2	108.67(12)
C7	C1	C6	110.16(11)
C3	C2	C1	111.35(11)
C3	C2	C9	111.19(10)
C9	C2	C1	112.08(10)
C4	C3	C2	123.87(12)
N1	C4	C5	116.84(11)
C3	C4	N1	118.85(11)
C3	C4	C5	124.30(12)
C4	C5	C6	110.80(12)
C5	C6	C1	111.68(11)
O1	C7	C1	119.27(16)
O1	C7	C8	121.63(16)
C8	C7	C1	119.10(14)

Atom	Atom	Atom	Angle/°
C10	C9	C2	122.48(11)
C10	C9	C14	116.15(12)
C14	C9	C2	121.33(11)
C9	C10	O2A	115.81(13)
C9	C10	O2B	114.15(15)
C9	C10	C11	123.84(13)
C11	C10	O2A	117.99(14)
C11	C10	O2B	116.79(16)
C12	C11	C10	118.36(13)
O4	C12	C11	124.03(13)
O4	C12	C13	116.34(12)
C11	C12	C13	119.63(12)
C14	C13	C12	120.19(12)
C13	C14	C9	121.82(13)
O2A	C15A	C16	113.5(2)
O3A	C15A	O2A	121.6(2)
O3A	C15A	C16	124.9(2)
O2B	C15B	C16	108.7(3)
O3B	C15B	O2B	123.1(3)
O3B	C15B	C16	128.2(3)
O5	C18	N1	121.29(11)

Atom	Atom	Atom	Angle/°
O5	C18	C19	122.31(11)
N1	C18	C19	116.39(10)
C20	C19	C18	118.43(11)
C24	C19	C18	121.71(11)
C24	C19	C20	119.68(11)
C21	C20	C19	119.81(12)
C22	C21	C20	120.34(13)
C21	C22	C23	120.23(13)
C22	C23	C24	119.87(13)
C23	C24	C19	120.01(13)
O6	C25	N1	119.12(13)

Atom	Atom	Atom	Angle/°
O6	C25	C26	121.76(12)
N1	C25	C26	119.05(12)
C25	C26	C27	116.64(11)
C28	C27	C26	120.25(13)
C28	C27	C32	118.17(14)
C32	C27	C26	121.56(12)
C29	C28	C27	120.77(15)
C30	C29	C28	120.20(16)
C29	C30	C31	119.73(16)
C30	C31	C32	120.29(16)
C31	C32	C27	120.81(14)

Table S12: Torsion Angles in for **7k**.

Atom	Atom	Atom	Atom	Angle/°
O2A	C10	C11	C12	-161.32(15)
O2B	C10	C11	C12	153.37(17)
O4	C12	C13	C14	-179.60(13)
O5	C18	C19	C20	-38.37(18)
O5	C18	C19	C24	136.78(13)
O6	C25	C26	C27	133.77(13)
N1	C4	C5	C6	165.10(11)
N1	C18	C19	C20	142.15(12)
N1	C18	C19	C24	-42.69(17)
N1	C25	C26	C27	-43.03(17)
C1	C2	C3	C4	-13.26(16)
C1	C2	C9	C10	97.62(15)
C1	C2	C9	C14	-79.89(15)
C2	C1	C6	C5	-61.44(15)
C2	C1	C7	O1	-79.4(2)
C2	C1	C7	C8	99.92(16)
C2	C3	C4	N1	178.31(10)
C2	C3	C4	C5	-0.57(19)
C2	C9	C10	O2A	-15.4(2)
C2	C9	C10	O2B	29.0(2)
C2	C9	C10	C11	-177.50(14)
C2	C9	C14	C13	177.37(13)
C3	C2	C9	C10	-137.01(14)
C3	C2	C9	C14	45.47(16)
C3	C4	C5	C6	-16.00(18)
C4	N1	C18	O5	142.08(12)
C4	N1	C18	C19	-38.44(15)
C4	N1	C25	O6	-7.18(17)
C4	N1	C25	C26	169.71(11)
C4	C5	C6	C1	46.53(16)
C6	C1	C2	C3	42.91(14)
C6	C1	C2	C9	168.18(11)
C6	C1	C7	O1	41.5(2)
C6	C1	C7	C8	-139.18(14)
C7	C1	C2	C3	163.74(11)
C7	C1	C2	C9	-70.98(14)
C7	C1	C6	C5	178.62(13)
C9	C2	C3	C4	-139.03(12)
C9	C10	C11	C12	0.5(2)
C10	O2A	C15A	O3A	9.3(4)
C10	O2A	C15A	C16	-170.95(18)
C10	O2B	C15B	O3B	-11.0(5)
C10	O2B	C15B	C16	165.5(2)
C10	C9	C14	C13	-0.3(2)

Atom	Atom	Atom	Atom	Angle/°
C10	C11	C12	O4	179.49(14)
C10	C11	C12	C13	-0.9(2)
C11	C12	C13	C14	0.8(2)
C12	C13	C14	C9	-0.1(2)
C14	C9	C10	O2A	162.28(14)
C14	C9	C10	O2B	-153.40(15)
C14	C9	C10	C11	0.1(2)
C15A	O2A	C10	C9	118.3(2)
C15A	O2A	C10	C11	-78.4(2)
C15B	O2B	C10	C9	-129.3(2)
C15B	O2B	C10	C11	75.2(3)
C17	O4	C12	C11	5.4(2)
C17	O4	C12	C13	-174.18(15)
C18	N1	C4	C3	-51.46(15)
C18	N1	C4	C5	127.51(12)
C18	N1	C25	O6	157.29(12)
C18	N1	C25	C26	-25.82(17)
C18	C19	C20	C21	177.87(12)
C18	C19	C24	C23	-176.54(12)
C19	C20	C21	C22	-1.7(2)
C20	C19	C24	C23	-1.4(2)
C20	C21	C22	C23	-0.5(2)
C21	C22	C23	C24	1.7(2)
C22	C23	C24	C19	-0.7(2)
C24	C19	C20	C21	2.62(19)
C25	N1	C4	C3	113.54(13)
C25	N1	C4	C5	-67.50(14)
C25	N1	C18	O5	-22.04(18)
C25	N1	C18	C19	157.44(11)
C25	C26	C27	C28	142.60(13)
C25	C26	C27	C32	-39.07(18)
C26	C27	C28	C29	177.59(13)
C26	C27	C32	C31	-178.81(13)
C27	C28	C29	C30	1.3(2)
C28	C27	C32	C31	-0.4(2)
C28	C29	C30	C31	-0.6(3)
C29	C30	C31	C32	-0.7(2)
C30	C31	C32	C27	1.2(2)
C32	C27	C28	C29	-0.8(2)

Table S13: Hydrogen Fractional Atomic Coordinates (×10^4) and Equivalent Isotropic Displacement Parameters (Å^2×10^3) for **7k**. U_{eq} is defined as 1/3 of the trace of the orthogonalised U_{ij}.

Atom	x	y	z	U_{eq}
H1	3719.49	6730.77	6317.46	49
H2	3842.32	6278.55	3046.23	44
H3	4425.58	5677.05	4262.07	41
H5A	4734.09	7348.31	5222.78	50
H5B	4494.66	7112.82	6821.08	50
H6A	4132.6	7352.11	3687	54
H6B	4006.19	7656.92	5391.48	54
H8A	2944.18	7166.73	6288	92
H8B	2942.99	6491.53	5643.6	92
H8C	2714.44	7002.37	4606.18	92
H11	2768.21	4951.48	3565.97	55
H13	3443.69	4744.95	7715.38	52
H14	3885.25	5506.06	6896.83	44
H16A	3335.61	6198.15	-952.17	86
H16B	3023.62	6546.34	244.34	86

Atom	x	y	z	U_{eq}
H16C	2849.46	6035.61	-945.25	86
H16D	2834.07	6312.02	-803.57	86
H16E	3208	5840.72	-932.72	86
H16F	3288.57	6485.36	-146.2	86
H17A	2616.89	3948.8	4260.43	114
H17B	2303.7	4440.04	4979.03	114
H17C	2335.76	3797.02	5826.73	114
H20	4774.12	4712.44	7335.83	44
H21	4352.23	4517.01	9600.76	51
H22	4168.82	5283.62	11383.1	56
H23	4412.22	6252.47	10932.74	56
H24	4816.22	6461.89	8615.99	47
H26A	5963.88	6702.09	4217.12	52
H26B	5870.76	6005.34	4397.91	52
H28	6326.82	5745.87	6553.99	63
H29	6538.18	5813.8	9279.2	81
H30	6240.04	6520.85	11004.21	77
H31	5737.38	7172.56	9993.24	65
H32	5541.71	7132.48	7253	50

Table S14: Atomic Occupancies for all atoms that are not fully occupied in **7k**.

Atom	Occupancy	Atom	Occupancy	Atom	Occupancy	Atom	Occupancy
O2A	0.610(2)	O3B	0.390(2)	H16A	0.610(2)	H16D	0.390(2)
O2B	0.390(2)	C15A	0.610(2)	H16B	0.610(2)	H16E	0.390(2)
O3A	0.610(2)	C15B	0.390(2)	H16C	0.610(2)	H16F	0.390(2)

Table S15: Solvent masking (PLATON/SQUEEZE) information for **7k**.

No	x	y	z	V	e	Content
1	0.250	0.250	-0.983	301.9	75.3	2pentane
2	0.750	0.750	-0.403	301.9	75.3	2pentane

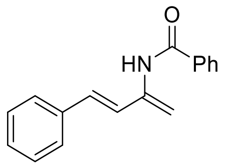

8a

R_1=2.3

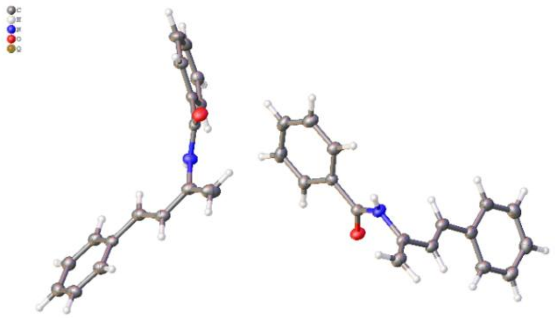

Crystal Data and Experimental of **8a** (CCDC number **2324899**).

The ellipsoids are displayed at 50% probability level.

**Compound**	**8a**
Formula	$C_{17}H_{15}NO$
$D_{calc.}$/ g cm^{-3}	1.234
μ/mm^{-1}	0.601
Formula Weight	249.30
Colour	clear pale colourless
Shape	needle
Size/mm^3	0.61×0.07×0.04
T/K	140.00(10)
Crystal System	orthorhombic
Flack Parameter	0.33(18)
Hooft Parameter	0.06(5)
Space Group	$Pca2_1$
a/Å	9.96338(8)
b/Å	10.35204(10)
c/Å	26.0239(3)
α/°	90
β/°	90
γ/°	90
V/Å^3	2684.14(5)
Z	8
Z'	2
Wavelength/Å	1.54184
Radiation type	Cu K_α
Θ_{min}/°	3.397
Θ_{max}/°	75.648
Measured Refl's.	29175
Indep't Refl's	5359
Refl's I≥2 σ(I)	5198
R_{int}	0.0217
Parameters	465
Restraints	1
Largest Peak	0.141
Deepest Hole	-0.139
GooF	1.045
wR_2 (all data)	0.0589
wR_2	0.0584
R_1 (all data)	0.0246
R_1	0.0236

Experimental. The compound of **8a** was crystallized from CH_2Cl_2 as a colorless crystal. A suitable crystal with dimensions 0.61 × 0.07 × 0.04 mm^3 was selected and mounted on a XtaLAB Synergy R, DW system, HyPix-Arc 150 diffractometer. The crystal was kept at a steady T = 140.00(10) K during data collection. The structure was solved with the ShelXT 2018/2 (Sheldrick, 2018) solution program using dual methods and by using Olex2 1.5 (Dolomanov et al., 2009) as the graphical interface. The model was refined with ShelXL 2019/3 (Sheldrick, 2015) using full matrix least squares minimisation on F^2.[9-10]

Crystal Data. $C_{17}H_{15}NO$, M_r = 249.30, orthorhombic, $Pca2_1$ (No. 29), a = 9.96338(8) Å, b = 10.35204(10) Å, c = 26.0239(3) Å, $\alpha = \beta = \gamma$ = 90°, V = 2684.14(5) Å^3, T = 140.00(10) K, Z = 8, Z' = 2, μ(Cu K_α) = 0.601, 29175 reflections measured, 5359 unique (R_{int} = 0.0217) which were used in all calculations. The final wR_2 was 0.0589 (all data) and R_1 was 0.0236 (I≥2 σ(I)).

Structure Quality Indicators

**Reflections:**	d min (CuKα) 2Θ=151.3° 0.80	I/σ(I) 61.9	Rint m=5.84 2.17%	Full 135.4° 99% to 151.3° 100
**Refinement:**	Shift 0.001	Max Peak 0.1	Min Peak -0.1	GooF 1.045

A clear pale colourless needle-shaped crystal with dimensions 0.61 × 0.07 × 0.04 mm^3 was mounted. Data were collected using a XtaLAB Synergy R, DW system, HyPix-Arc 150 diffractometer operating at T = 140.00(10) K.

Data were measured using ω scans with Cu K_α radiation. The diffraction pattern was indexed and the total number of runs and images was based on the strategy calculation from the program CrysAlisPro system (CCD 43.98a 64-bit (release 24-11-2023)). The maximum resolution that was achieved was Θ = 75.648° (0.80 Å).

The unit cell was refined using CrysAlisPro 1.171.43.99a (Rigaku OD, 2023) on 19839 reflections, 68% of the observed reflections.

Data reduction, scaling and absorption corrections were performed using CrysAlisPro 1.171.43.99a (Rigaku OD, 2023). The final completeness is 100.00 % out to 75.648° in Θ. A gaussian absorption correction was performed using CrysAlisPro 1.171.43.99a (Rigaku Oxford Diffraction, 2023). The numerical absorption correction was based on gaussian integration over a multifaceted crystal model. The empirical absorption correction was done using spherical harmonics, implemented in SCALE3 ABSPACK scaling algorithm. The absorption coefficient μ of this crystal is 0.601 mm^{-1} at this wavelength (λ = 1.54184Å) and the minimum and maximum transmissions are 0.761 and 1.000.

The structure was solved and the space group $Pca2_1$ (# 29) determined by the ShelXT 2018/2 (Sheldrick, 2018) structure solution program using dual methods and refined by full matrix least squares minimisation on $\boldsymbol{F}^2$ using version 2019/3 of ShelXL (Sheldrick, 2015). All non-hydrogen atoms were refined anisotropically. Hydrogen atom positions were calculated geometrically and refined using the riding model.

_refine_special_details: Refined as a 2-component inversion twin.

The value of Z' is 2. This means that there are two independent molecules in the asymmetric unit. The moiety formula is $C_{17}H_{15}NO$.

The Flack parameter was refined to 0.33(18). Determination of absolute structure using Bayesian statistics on Bijvoet differences using the Olex2 results in 0.06(5). This structure is in chiral space group, but there are no chiral atoms. Note: The Flack parameter is used to determine chirality of the crystal studied, the value should be near 0, a value of 1 means that the stereochemistry is wrong and the model should be inverted. A value of 0.5 means that the crystal consists of a racemic mixture of the two enantiomers.

Data Plots: Diffraction Data

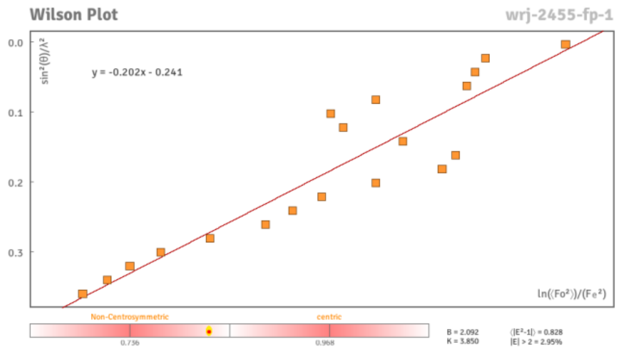

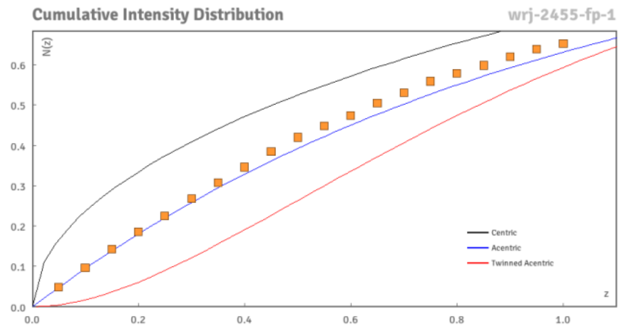

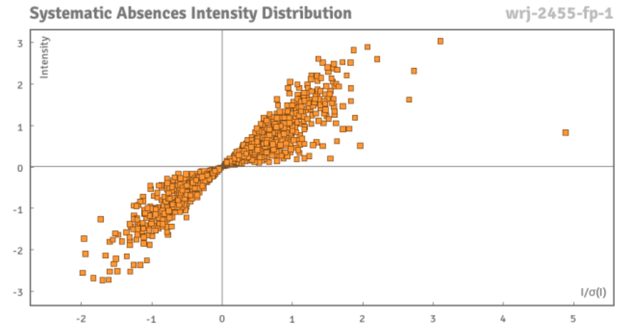

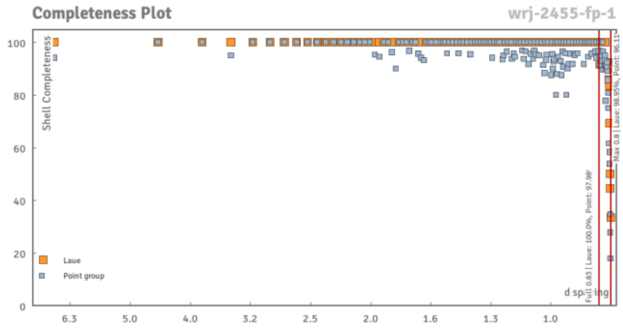

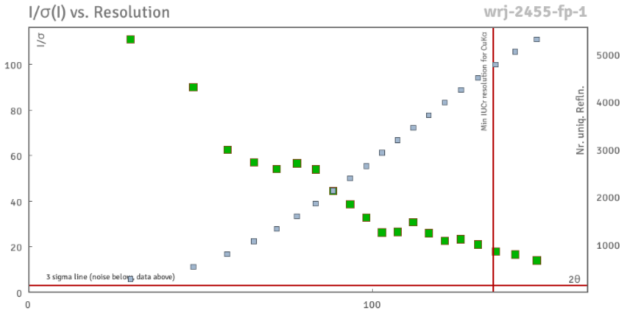

Data Plots: Refinement and Data

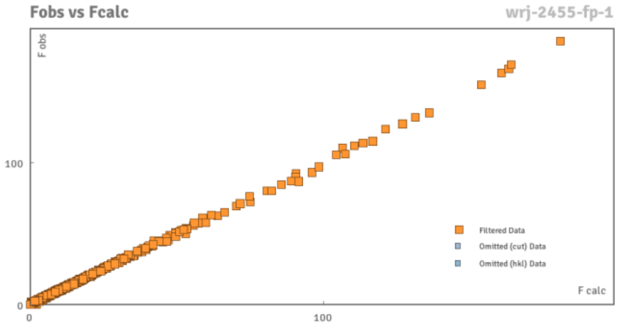

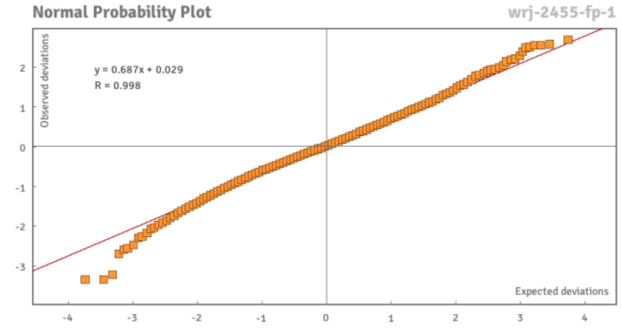

Reflection Statistics

Total reflections (after filtering)	31319	Unique reflections	5359
Completeness	0.961	Mean I/σ	41.9
hkl_{max} collected	(12, 12, 32)	hkl_{min} collected	(-10, -12, -32)
hkl_{max} used	(12, 12, 32)	hkl_{min} used	(0, 0, -32)
Lim d_{max} collected	100.0	Lim d_{min} collected	0.77
d_{max} used	26.02	d_{min} used	0.8

Friedel pairs	3864	Friedel pairs merged	0
Inconsistent equivalents	0	R_{int}	0.0217
R_{sigma}	0.0161	Intensity transformed	0
Omitted reflections	0	Omitted by user (OMIT hkl)	0
Multiplicity	(5453, 4524, 1954, 686, 352, 253, 145, 96, 100, 56, 55, 39, 19, 10, 11, 2, 2)	Maximum multiplicity	28
Removed systematic absences	2144	Filtered off (Shel/OMIT)	0

Table S16: Fractional Atomic Coordinates (×10[4]) and Equivalent Isotropic Displacement Parameters (Å^2×10^3) for **8a**. U_{eq} is defined as 1/3 of the trace of the orthogonalised U_{ij}.

Atom	x	y	z	U_{eq}
O1	3194.4(10)	333.2(11)	4140.6(5)	28.1(3)
N1	5289.7(14)	-334.4(13)	3931.4(6)	25.1(3)
C1	4413.3(15)	540.3(15)	4118.3(6)	22.3(3)
C2	4978.5(15)	1799.1(15)	4303.3(6)	22.2(3)
C3	4131.7(16)	2597.2(16)	4587.5(7)	27.4(3)
C4	4568.0(19)	3796.7(17)	4755.6(7)	33.2(4)
C5	5853.6(18)	4208.7(16)	4639.3(7)	32.0(4)
C6	6707.2(17)	3417.4(17)	4363.3(8)	32.3(4)
C7	6277.5(16)	2214.8(16)	4194.0(7)	27.9(3)
C8	4875.8(15)	-1600.8(16)	3775.5(7)	25.8(3)
C9	5358.5(18)	-2620.7(17)	4026.4(8)	32.5(4)
C10	3977.5(16)	-1731.6(16)	3336.9(7)	26.8(3)
C11	3643.5(16)	-809.9(17)	3002.8(7)	28.3(3)
C12	2702.7(16)	-976.5(16)	2572.3(7)	26.9(3)
C13	1772.0(17)	-1989.8(18)	2553.2(7)	32.1(4)
C14	911.0(18)	-2124.7(19)	2138.9(8)	35.4(4)
C15	950.8(19)	-1252.2(19)	1736.1(7)	35.2(4)
C16	1850(2)	-233.0(19)	1751.5(7)	36.2(4)
C17	2712.9(19)	-90.3(17)	2165.0(7)	32.9(4)
O2	7141.7(10)	5016.9(11)	5835.8(5)	29.6(3)
N2	4944.7(13)	4709.3(13)	6035.5(6)	25.1(3)
C18	5975.5(14)	5405.5(15)	5844.5(6)	22.4(3)
C19	5637.7(15)	6730.4(15)	5649.0(6)	22.9(3)
C20	4387.4(16)	7064.5(15)	5450.7(7)	24.5(3)
C21	4151.1(18)	8310.2(16)	5275.1(7)	30.2(4)
C22	5152.6(19)	9238.4(17)	5308.7(7)	33.0(4)
C23	6398.6(19)	8908.1(18)	5501.5(8)	35.3(4)
C24	6654.1(16)	7658.7(17)	5666.3(7)	29.8(4)
C25	5047.5(15)	3471.7(16)	6272.8(7)	27.7(3)
C26	5864.1(19)	2553.5(18)	6095.8(9)	40.2(5)
C27	4208.8(17)	3280.2(16)	6725.2(7)	28.4(4)
C28	3394.4(16)	4163.6(16)	6933.8(7)	27.1(3)
C29	2558.6(16)	4006.7(15)	7392.6(6)	25.7(3)
C30	2719.6(16)	3002.1(17)	7746.0(7)	30.3(4)
C31	1906.4(18)	2929.6(18)	8178.1(7)	32.6(4)
C32	912.1(18)	3842.7(18)	8263.3(7)	32.6(4)
C33	734.4(18)	4837.5(17)	7913.8(7)	31.4(4)
C34	1559.0(17)	4925.3(16)	7485.9(7)	28.4(4)

Table S17: Anisotropic Displacement Parameters (×10[4]) for **8a**. The anisotropic displacement factor exponent takes the form: $-2\pi^2[h^2a^{*2}U_{11}+ ... +2hka^*b^*U_{12}]$

Atom	U_{11}	U_{22}	U_{33}	U_{23}	U_{13}	U_{12}
O1	18.3(5)	27.3(6)	38.6(6)	-2.0(5)	1.1(5)	-1.5(4)
N1	17.0(6)	23.7(6)	34.4(8)	-5.0(5)	-1.7(5)	-0.9(5)

Atom	U_{11}	U_{22}	U_{33}	U_{23}	U_{13}	U_{12}
C1	18.9(7)	23.5(7)	24.3(8)	1.3(6)	-0.6(6)	1.0(6)
C2	21.0(7)	22.3(7)	23.3(7)	1.4(6)	-3.3(6)	1.3(6)
C3	25.2(8)	28.9(8)	28.1(8)	-1.6(7)	-0.1(7)	2.5(6)
C4	37.0(9)	30.2(9)	32.3(9)	-8.7(7)	-3.4(7)	7.2(7)
C5	38.2(9)	23.9(8)	33.9(9)	-3.4(7)	-9.3(8)	-0.9(7)
C6	26.9(8)	27.7(9)	42.4(10)	0.4(7)	-3.9(8)	-2.5(7)
C7	22.7(7)	24.0(8)	36.9(9)	0.0(7)	-0.2(7)	1.0(6)
C8	19.4(7)	24.1(8)	34.0(9)	-5.7(6)	2.8(6)	0.1(6)
C9	30.4(8)	26.2(8)	40.8(10)	-3.3(7)	-3.2(7)	2.2(7)
C10	22.6(7)	23.7(8)	34.1(9)	-5.3(7)	1.4(7)	-0.1(6)
C11	25.6(8)	26.5(8)	32.9(9)	-3.7(7)	3.1(7)	-3.0(6)
C12	24.6(8)	26.0(8)	30.0(8)	-2.6(6)	3.1(6)	0.8(6)
C13	29.3(9)	28.9(9)	38.0(10)	3.6(7)	-1.8(7)	-1.9(7)
C14	29.1(8)	32.9(9)	44.2(11)	-3.5(8)	-6.4(8)	-2.5(7)
C15	32.6(9)	39.9(10)	33.1(10)	-7.2(8)	-5.8(7)	5.8(8)
C16	42.0(10)	36.5(10)	30.1(9)	3.6(8)	3.3(8)	4.2(8)
C17	35.2(9)	29.4(8)	34.0(9)	-0.7(7)	3.8(8)	-4.1(7)
O2	15.6(5)	30.6(6)	42.6(7)	-2.4(5)	-1.5(5)	1.7(4)
N2	16.3(6)	24.7(7)	34.3(7)	3.8(5)	1.4(5)	2.2(5)
C18	17.8(7)	24.4(7)	24.9(7)	-3.8(6)	-0.8(6)	0.6(6)
C19	20.9(7)	24.6(8)	23.1(7)	-0.6(6)	3.7(6)	-0.2(6)
C20	21.5(7)	24.3(8)	27.9(7)	-2.3(6)	0.5(6)	1.2(6)
C21	31.4(8)	29.3(8)	29.8(8)	0.8(7)	-0.6(7)	5.3(7)
C22	44.1(10)	26.7(8)	28.2(9)	5.6(7)	2.9(7)	-0.2(7)
C23	36.8(9)	31.4(9)	37.6(10)	4.6(8)	3.6(8)	-10.8(8)
C24	23.4(8)	32.7(9)	33.2(9)	3.4(7)	0.5(7)	-4.1(7)
C25	21.2(7)	24.9(8)	36.9(9)	4.1(7)	-2.6(7)	-1.7(6)
C26	33.6(10)	27.5(9)	59.5(13)	6.1(9)	9.3(9)	4.4(7)
C27	25.4(8)	24.7(8)	35.1(9)	6.0(7)	-3.6(6)	-2.4(6)
C28	24.2(8)	25.9(8)	31.2(8)	5.2(7)	-4.7(6)	-3.6(6)
C29	22.7(7)	26.2(8)	28.3(8)	2.5(6)	-3.9(6)	-4.9(6)
C30	25.9(8)	28.8(8)	36.1(9)	6.3(7)	-3.3(7)	-0.6(7)
C31	32.6(9)	32.3(9)	32.8(9)	9.6(7)	-2.5(7)	-4.9(7)
C32	32.2(9)	36.8(9)	28.7(9)	1.8(7)	0.4(7)	-5.4(7)
C33	31.3(9)	30.6(9)	32.3(9)	-1.2(7)	-1.4(7)	-1.0(7)
C34	30.0(8)	24.9(8)	30.3(8)	4.3(7)	-5.3(7)	-2.0(7)

Table S18: Bond Lengths in Åfor **8a**.

Atom	Atom	Length/Å
O1	C1	1.2345(18)
N1	C1	1.349(2)
N1	C8	1.433(2)
C1	C2	1.499(2)
C2	C3	1.393(2)
C2	C7	1.393(2)
C3	C4	1.386(2)
C4	C5	1.384(3)
C5	C6	1.382(3)
C6	C7	1.388(2)
C8	C9	1.331(2)
C8	C10	1.457(2)
C10	C11	1.333(3)
C11	C12	1.471(2)
C12	C13	1.401(2)
C12	C17	1.402(2)
C13	C14	1.385(3)
C14	C15	1.384(3)
C15	C16	1.385(3)
C16	C17	1.385(3)
O2	C18	1.2298(19)
N2	C18	1.349(2)
N2	C25	1.426(2)
C18	C19	1.501(2)
C19	C20	1.392(2)
C19	C24	1.397(2)
C20	C21	1.388(2)
C21	C22	1.388(3)
C22	C23	1.382(3)
C23	C24	1.386(3)
C25	C26	1.333(3)
C25	C27	1.457(3)
C27	C28	1.338(3)
C28	C29	1.465(2)

Atom	Atom	Length/Å
C29	C30	1.398(2)
C29	C34	1.398(2)
C30	C31	1.388(3)
C31	C32	1.387(3)
C32	C33	1.385(3)
C33	C34	1.387(3)

Table S19: Bond Angles for **8a**.

Atom	Atom	Atom	Angle/°
C1	N1	C8	122.00(13)
O1	C1	N1	122.49(14)
O1	C1	C2	120.36(14)
N1	C1	C2	117.14(13)
C3	C2	C1	117.29(14)
C7	C2	C1	123.50(15)
C7	C2	C3	119.18(15)
C4	C3	C2	120.58(16)
C5	C4	C3	119.83(17)
C6	C5	C4	120.04(16)
C5	C6	C7	120.44(16)
C6	C7	C2	119.92(16)
N1	C8	C10	118.92(14)
C9	C8	N1	118.87(15)
C9	C8	C10	122.18(15)
C11	C10	C8	126.72(16)
C10	C11	C12	124.89(16)
C13	C12	C11	122.46(16)
C13	C12	C17	117.90(16)
C17	C12	C11	119.64(15)
C14	C13	C12	120.88(17)
C15	C14	C13	120.39(17)
C14	C15	C16	119.60(17)
C15	C16	C17	120.35(17)
C16	C17	C12	120.86(16)
C18	N2	C25	125.78(13)
O2	C18	N2	123.45(15)
O2	C18	C19	120.29(14)
N2	C18	C19	116.24(13)
C20	C19	C18	123.60(14)
C20	C19	C24	119.33(15)
C24	C19	C18	117.07(14)
C21	C20	C19	120.31(15)
C22	C21	C20	120.02(16)
C23	C22	C21	119.83(16)
C22	C23	C24	120.54(16)
C23	C24	C19	119.92(16)
N2	C25	C27	115.53(14)
C26	C25	N2	122.32(17)
C26	C25	C27	122.15(16)
C28	C27	C25	125.64(15)
C27	C28	C29	126.85(15)
C30	C29	C28	123.63(15)
C30	C29	C34	118.26(15)
C34	C29	C28	118.11(15)
C31	C30	C29	120.42(16)
C32	C31	C30	120.64(16)
C33	C32	C31	119.53(17)
C32	C33	C34	120.01(17)
C33	C34	C29	121.13(15)

Table S20: Torsion Angles for **8a**.

Atom	Atom	Atom	Atom	Angle/°
O1	C1	C2	C3	12.3(2)
O1	C1	C2	C7	-165.97(16)
N1	C1	C2	C3	-167.61(15)
N1	C1	C2	C7	14.2(2)
N1	C8	C10	C11	11.5(3)
C1	N1	C8	C9	-116.70(18)
C1	N1	C8	C10	65.4(2)
C1	C2	C3	C4	-177.65(16)
C1	C2	C7	C6	177.50(16)
C2	C3	C4	C5	0.2(3)
C3	C2	C7	C6	-0.7(3)
C3	C4	C5	C6	-1.0(3)
C4	C5	C6	C7	1.0(3)
C5	C6	C7	C2	-0.1(3)
C7	C2	C3	C4	0.7(2)
C8	N1	C1	O1	-4.1(3)

Atom	Atom	Atom	Atom	Angle/°
C8	N1	C1	C2	175.79(15)
C8	C10	C11	C12	-178.31(15)
C9	C8	C10	C11	-166.34(18)
C10	C11	C12	C13	19.0(3)
C10	C11	C12	C17	-161.70(17)
C11	C12	C13	C14	-179.30(17)
C11	C12	C17	C16	179.17(17)
C12	C13	C14	C15	-0.4(3)
C13	C12	C17	C16	-1.5(3)
C13	C14	C15	C16	-0.6(3)
C14	C15	C16	C17	0.5(3)
C15	C16	C17	C12	0.6(3)
C17	C12	C13	C14	1.4(3)
O2	C18	C19	C20	153.00(16)
O2	C18	C19	C24	-26.4(2)
N2	C18	C19	C20	-28.5(2)
N2	C18	C19	C24	152.14(16)
N2	C25	C27	C28	-1.4(2)
C18	N2	C25	C26	-40.2(3)
C18	N2	C25	C27	139.24(16)
C18	C19	C20	C21	-179.76(16)
C18	C19	C24	C23	-178.65(16)
C19	C20	C21	C22	-1.6(3)
C20	C19	C24	C23	2.0(3)
C20	C21	C22	C23	2.1(3)
C21	C22	C23	C24	-0.6(3)
C22	C23	C24	C19	-1.5(3)
C24	C19	C20	C21	-0.4(2)
C25	N2	C18	O2	3.0(3)
C25	N2	C18	C19	-175.41(15)
C25	C27	C28	C29	-178.87(16)
C26	C25	C27	C28	178.05(18)
C27	C28	C29	C30	15.0(3)
C27	C28	C29	C34	-166.03(17)
C28	C29	C30	C31	178.71(16)
C28	C29	C34	C33	-179.82(15)
C29	C30	C31	C32	0.7(3)
C30	C29	C34	C33	-0.8(2)
C30	C31	C32	C33	-0.2(3)
C31	C32	C33	C34	-0.9(3)
C32	C33	C34	C29	1.4(3)
C34	C29	C30	C31	-0.2(2)

Table S21: Hydrogen Fractional Atomic Coordinates (×10[4]) and Equivalent Isotropic Displacement Parameters (Å[2]×10[3]) for **8a**. U_{eq} is defined as 1/3 of the trace of the orthogonalised U_{ij}.

Atom	x	y	z	U_{eq}
H1	6130(20)	-250(20)	4004(8)	36(6)
H3	3230(20)	2293(19)	4658(8)	29(5)
H4	3950(20)	4350(20)	4955(9)	43(6)
H5	6170(20)	5030(20)	4754(8)	37(5)
H6	7660(20)	3720(20)	4288(9)	50(6)
H7	6900(20)	1670(20)	3997(8)	41(6)
H9A	5980(20)	-2510(20)	4335(9)	36(5)
H9B	5120(20)	-3510(20)	3905(9)	41(6)
H10	3620(20)	-2590(20)	3302(8)	33(5)
H11	4100(20)	40(20)	3025(8)	31(5)

Atom	x	y	z	U_{eq}
H13	1750(20)	-2600(20)	2826(9)	40(6)
H14	260(20)	-2800(20)	2139(9)	44(6)
H15	340(20)	-1360(20)	1446(10)	44(6)
H16	1880(20)	390(20)	1481(10)	50(7)
H17	3380(20)	610(20)	2191(9)	45(6)
H2	4140(20)	5050(20)	6026(9)	34(5)
H20	3680(20)	6430(20)	5442(8)	32(5)
H21	3230(20)	8520(20)	5134(9)	41(6)
H22	4950(20)	10130(20)	5195(9)	42(6)
H23	7120(30)	9580(20)	5522(9)	51(7)
H24	7560(20)	7410(20)	5802(9)	38(5)
H26A	6420(20)	2690(20)	5786(9)	39(6)
H26B	5930(20)	1720(20)	6288(10)	59(7)
H27	4290(20)	2410(20)	6898(8)	32(5)
H28	3290(20)	5020(20)	6779(8)	30(5)
H30	3430(20)	2360(20)	7690(8)	37(5)
H31	2070(20)	2200(20)	8431(9)	43(6)
H32	300(20)	3770(20)	8572(9)	42(6)
H33	0(20)	5470(20)	7986(8)	33(5)
H34	1470(20)	5680(20)	7247(8)	34(5)

Table S22: Hydrogen Bond information for **8a**.

D	H	A	d(D-H)/Å	d(H-A)/Å	d(D-A)/Å	D-H-A/deg
N1	H1	O1[1]	0.86(2)	2.09(2)	2.9448(17)	171(2)
N2	H2	O2[2]	0.88(2)	2.05(2)	2.8548(17)	151.9(19)

––––

[1]1/2+x,-y,+z; [2]-1/2+x,1-y,+

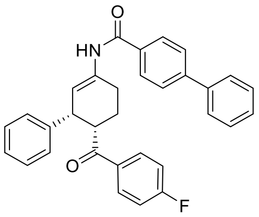

9h

R_1=4.45%

Crystal Data and Experimental of **9h** (CCDC number **2324850**).

The ellipsoids are displayed at 50% probability level.

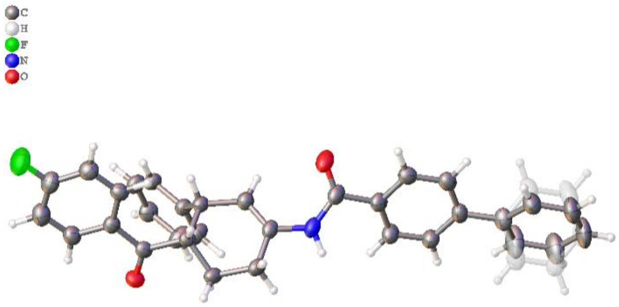

Experimental. The compound of **9h** was crystallized from Ethyl acetate/Pentane (v:v = 1:5) as a colorless crystal. A suitable crystal with dimensions 0.60 × 0.11 × 0.07 mm³ was selected and mounted on a SuperNova, Dual, Cu at home/near, Atlas diffractometer. The crystal was kept at a steady *T* = 139.99(10) K during data collection. The structure was solved with the ShelXT (Sheldrick, 2015) solution program using dual methods and by using Olex2 1.5 (Dolomanov et al., 2009) as the graphical interface. The model was refined with ShelXL 2019/3 (Sheldrick, 2015) using full matrix least squares minimisation on $\boldsymbol{F}^2$.[9-10]

Crystal Data. $C_{33.5}H_{29}FNO_{2.5}$, M_r = 504.58, monoclinic, $P2_1$ (No. 4), a = 14.6064(7) Å, b = 5.7232(3) Å, c = 15.4483(8) Å, β = 93.703(5)°, α = γ = 90°, V = 1288.71(11) Å³, T = 139.99(10) K, Z = 2, Z' = 1, μ(Cu K$_\alpha$) = 0.692, 10514 reflections measured, 4878 unique (R_{int} = 0.0453) which were used in all calculations. The final wR_2 was 0.1219 (all data) and R_1 was 0.0445 (I≥2 σ(I)).

**Compound**	**9h**
Formula	$C_{33.5}H_{29}FNO_{2.5}$
$D_{calc.}$/ g cm^{-3}	1.300
μ/mm^{-1}	0.692
Formula Weight	504.58
Colour	clear pale colourless
Shape	prism
Size/mm³	0.60×0.11×0.07
T/K	139.99(10)
Crystal System	monoclinic
Flack Parameter	-0.02(18)
Hooft Parameter	-0.02(18)
Space Group	$P2_1$
a/Å	14.6064(7)
b/Å	5.7232(3)
c/Å	15.4483(8)
α/°	90
β/°	93.703(5)
γ/°	90
V/Å³	1288.71(11)
Z	2
Z'	1
Wavelength/Å	1.54184
Radiation type	Cu K$_\alpha$
Θ_{min}/°	3.032
Θ_{max}/°	72.416
Measured Refl's.	10514
Indep't Refl's	4878
Refl's I≥2 σ(I)	4138
R_{int}	0.0453
Parameters	360
Restraints	2
Largest Peak	0.140
Deepest Hole	-0.166
GooF	1.028
wR_2 (all data)	0.1219
wR_2	0.1140
R_1 (all data)	0.0552
R_1	0.0445

Structure Quality Indicators

**Reflections:**	d min (CuKα) 2Θ=144.8° 0.81	I/σ(I) 19.8	Rint m=2.16 4.53%	Full 135.4° 98% to 144.8° 99.8
**Refinement:**	Shift 0.000	Max Peak 0.1	Min Peak -0.2	GooF 1.028	Hooft -.02(18)

A clear pale colourless prism-shaped crystal with dimensions 0.60 × 0.11 × 0.07 mm^3 was mounted. Data were collected using a SuperNova, Dual, Cu at home/near, Atlas diffractometer operating at T = 139.99(10) K.

Data were measured using ω scans with Cu K_α radiation. The diffraction pattern was indexed and the total number of runs and images was based on the strategy calculation from the program CrysAlisPro system (CCD 43.94a 64-bit (release 20-10-2023)). The maximum resolution that was achieved was Θ = 72.416° (0.81 Å).

The unit cell was refined using CrysAlisPro 1.171.43.94a (Rigaku OD, 2023) on 4710 reflections, 45% of the observed reflections.

Data reduction, scaling and absorption corrections were performed using CrysAlisPro 1.171.43.94a (Rigaku OD, 2023). The final completeness is 99.80 % out to 72.416° in Θ. A gaussian absorption correction was performed using CrysAlisPro 1.171.43.94a (Rigaku Oxford Diffraction, 2023). The numerical absorption correction was based on gaussian integration over a multifaceted crystal model. The empirical absorption correction was done using spherical harmonics, implemented in SCALE3 ABSPACK scaling algorithm. The absorption coefficient μ of this crystal is 0.692 mm^{-1} at this wavelength (λ = 1.54184Å) and the minimum and maximum transmissions are 0.552 and 1.000.

The structure was solved and the space group $P2_1$ (# 4) determined by the ShelXT (Sheldrick, 2015) structure solution program using dual methods and refined by full matrix least squares minimisation on $\boldsymbol{F^2}$ using version 2019/3 of ShelXL 2019/3 (Sheldrick, 2015). All non-hydrogen atoms were refined anisotropically. Most hydrogen atom positions were calculated geometrically and refined using the riding model, but some hydrogen atoms were refined freely.

_smtbx_masks_special_details: A solvent mask was calculated and 29 electrons were found in a volume of 108$Å^3$ in 1 void per unit cell. This is consistent with the presence of 0.5[C3H6O] per Asymmetric Unit which account for 32 electrons per unit cell.

There is a single formula unit in the asymmetric unit, which is represented by the reported sum formula. In other words: Z is 2 and Z' is 1. The moiety formula is $C_{32}H_{26}FNO_2$, 0.5[C_3H_6O].

The Flack parameter was refined to -0.02(18). Determination of absolute structure using Bayesian statistics on Bijvoet differences using the Olex2 results in -0.02(18). The chiral atoms in this structure are: C3(R), C4(S). Note: The Flack parameter is used to determine chirality of the crystal studied, the value should be near 0, a value of 1 means that the stereochemistry is wrong and the model should be inverted. A value of 0.5 means that the crystal consists of a racemic mixture of the two enantiomers.

Data Plots: Diffraction Data

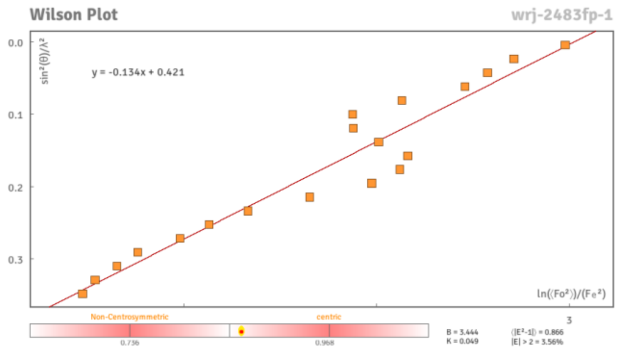

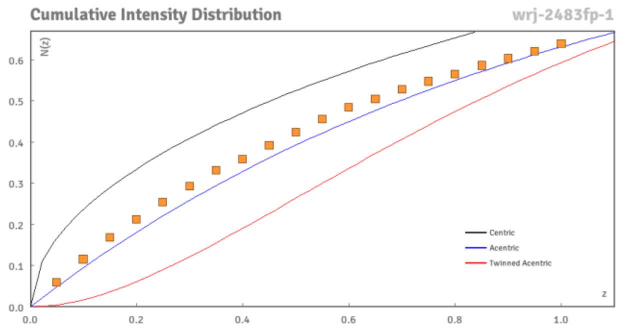

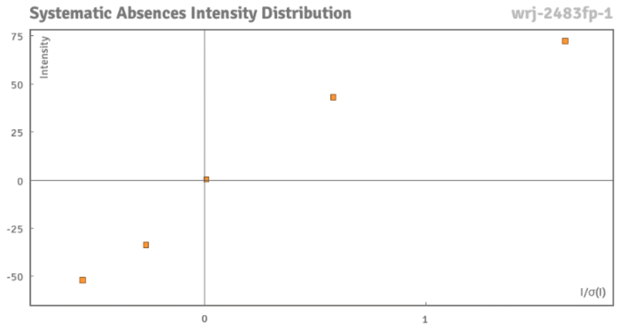

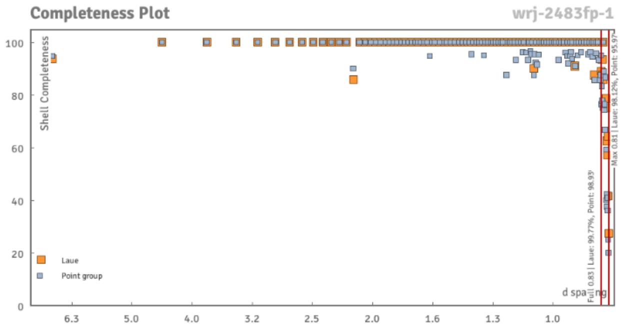

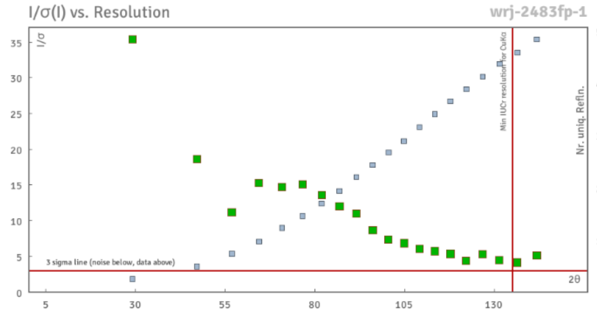

Data Plots: Refinement and Data

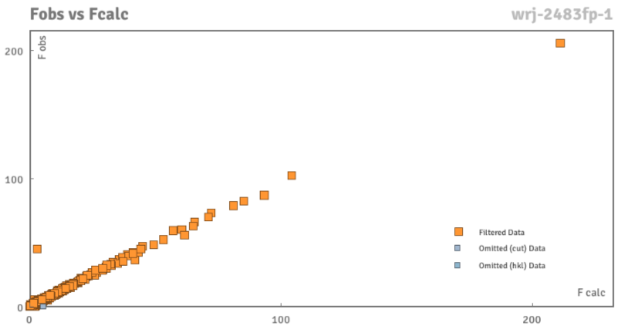

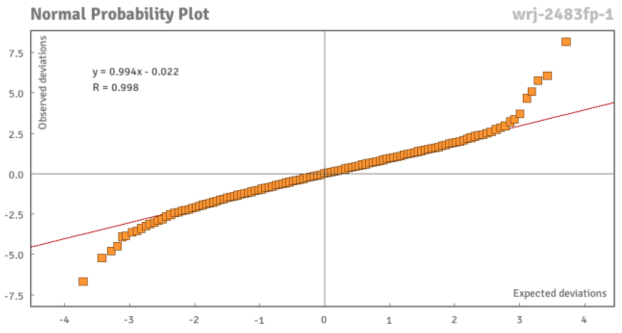

Reflection Statistics

Total reflections (after filtering)	10521	Unique reflections	4878
Completeness	0.96	Mean I/σ	10.78
hkl$_{max}$ collected	(18, 6, 18)	hkl$_{min}$ collected	(-15, -6, -19)
hkl$_{max}$ used	(18, 6, 19)	hkl$_{min}$ used	(-18, -6, 0)
Lim d$_{max}$ collected	100.0	Lim d$_{min}$ collected	0.77
d$_{max}$ used	14.58	d$_{min}$ used	0.81

Friedel pairs	1714	Friedel pairs merged	0
Inconsistent equivalents	10	R_{int}	0.0453
R_{sigma}	0.0505	Intensity transformed	0
Omitted reflections	0	Omitted by user (OMIT hkl)	1
Multiplicity	(3674, 1803, 582, 259, 65, 20, 2)	Maximum multiplicity	8
Removed systematic absences	6	Filtered off (Shel/OMIT)	0

Table S23: Fractional Atomic Coordinates (×10^4) and Equivalent Isotropic Displacement Parameters (Å^2×10^3) for **9h.** U_{eq} is defined as 1/3 of the trace of the orthogonalised U_{ij}.

Atom	x	y	z	U_{eq}
F1	1530.1(18)	8684(5)	8795.7(15)	77.6(7)
O1	4850.3(18)	8535(5)	2933.6(18)	61.6(7)
O2	2082.8(14)	1328(4)	5876.9(14)	44.5(5)
N1	4688.5(18)	4600(5)	3099.6(18)	45.1(6)
C1	4144.5(19)	4465(5)	3840(2)	38.9(7)
C2	3480.1(19)	5975(6)	3980(2)	39.5(6)
C3	2843.4(18)	5750(5)	4707.5(19)	35.5(6)
C4	3213.7(18)	3926(5)	5394.8(19)	34.7(6)
C5	3604(2)	1784(5)	4974(2)	42.5(7)
C6	4383(2)	2410(5)	4405(2)	43.2(7)
C7	1866.7(19)	5213(5)	4334.7(19)	37.3(6)
C8	1143.0(19)	6563(5)	4596(2)	39.9(6)
C9	250(2)	6087(6)	4285(2)	45.8(7)
C10	62(2)	4288(6)	3721(2)	44.4(7)
C11	778(2)	2941(7)	3442(2)	55.4(9)
C12	1675(2)	3429(7)	3745(2)	54.2(9)
C13	2472.3(18)	3198(5)	5990.7(19)	36.7(6)
C14	2229.2(19)	4744(5)	6718(2)	37.5(6)
C15	2666(2)	6850(5)	6913(2)	41.2(7)
C16	2439(2)	8191(6)	7612(2)	47.0(7)
C17	1757(2)	7373(7)	8112(2)	52.0(8)
C18	1301(2)	5305(7)	7940(2)	56.0(9)
C19	1538(2)	4007(6)	7241(2)	45.7(7)
C20	4958(2)	6541(6)	2673(2)	44.4(7)
C21	5425.7(19)	6064(6)	1853(2)	42.2(7)
C22	5278(2)	4064(6)	1356(2)	46.8(8)
C23	5733(2)	3738(6)	604(2)	47.3(7)
C24	6353(2)	5395(6)	328(2)	44.6(7)
C25	6479(2)	7407(6)	822(2)	44.1(7)
C26	6019(2)	7767(6)	1570(2)	42.7(7)
C27	6772(4)	4918(10)	-541(3)	43(2)
C28	6919(5)	2651(9)	-823(4)	58(2)
C29	7375(5)	2268(8)	-1572(4)	58(2)
C30	7685(5)	4153(11)	-2040(3)	64(3)
C31	7538(5)	6420(10)	-1758(4)	84(3)
C32	7082(5)	6803(8)	-1008(4)	69(3)
C33	6954(4)	5073(15)	-398(4)	46(3)
C38	7781(4)	6230(11)	-476(4)	63(2)
C37	8286(4)	5835(13)	-1194(4)	74(3)
C36	7964(5)	4283(16)	-1835(4)	76(4)
C35	7136(6)	3126(17)	-1758(4)	78(4)
C34	6631(4)	3520(18)	-1040(5)	75(4)

Table S24: Anisotropic Displacement Parameters (×10^4) for **9h.** The anisotropic displacement factor exponent takes the form: $-2\pi^2[h^2a^{*2} \times U_{11} + ... + 2hka^* \times b^* \times U_{12}]$

Atom	U_{11}	U_{22}	U_{33}	U_{23}	U_{13}	U_{12}
F1	78.3(16)	89.7(18)	67.5(13)	-23.7(13)	24.8(12)	-8.6(14)
O1	55.1(14)	55.0(15)	78.3(16)	-23.7(14)	30.4(12)	-13.8(12)
O2	38.1(10)	35.3(11)	60.9(13)	-0.8(10)	8.7(9)	-8.6(9)
N1	35.7(13)	48.1(17)	52.8(15)	-10.1(12)	14.4(12)	0.7(11)
C1	27.3(13)	41.9(16)	48.1(16)	-8.8(12)	8.2(12)	-3.7(11)
C2	31.3(13)	41.0(17)	46.9(16)	-0.8(13)	7.0(12)	0.5(12)
C3	28.5(13)	31.4(15)	47.3(15)	-1.7(11)	7.6(11)	-0.8(10)
C4	24.4(12)	29.6(14)	50.4(16)	1.3(12)	5.3(11)	-2.8(10)
C5	31.0(13)	32.1(16)	65.1(19)	-1.9(13)	9.2(13)	0.1(11)
C6	31.7(14)	37.6(16)	61.2(19)	-7.9(13)	9.3(13)	2.0(12)
C7	29.8(13)	36.6(15)	46.0(15)	0.2(12)	6.8(12)	2.3(11)
C8	32.7(13)	35.7(15)	51.5(16)	-1.8(13)	5.1(12)	5.1(12)
C9	31.9(14)	46.1(17)	59.9(19)	-3.3(14)	6.7(13)	9.1(13)
C10	27.9(13)	50.8(18)	54.2(17)	-0.4(14)	-0.6(12)	1.2(12)
C11	38.3(17)	60(2)	67(2)	-23.6(17)	0.1(15)	2.3(15)
C12	33.0(15)	59(2)	71(2)	-22.2(18)	4.4(15)	7.0(15)
C13	25.8(13)	35.5(16)	49.1(16)	4.6(12)	3.4(11)	0.5(11)
C14	28.5(13)	39.3(16)	44.7(15)	2.9(12)	2.7(11)	2.5(11)
C15	35.5(14)	39.7(17)	48.6(16)	4.2(13)	3.0(13)	-1.6(12)
C16	43.2(16)	48.2(19)	49.3(17)	-2.6(14)	1.4(13)	-2.4(14)
C17	48.2(18)	62(2)	46.3(17)	-5.8(15)	7.5(15)	2.0(16)
C18	45.1(18)	66(2)	58(2)	2.1(17)	12.7(16)	-3.6(17)
C19	35.0(15)	45.2(18)	57.5(18)	3.3(14)	8.1(13)	-3.7(13)
C20	30.0(13)	48.5(19)	55.7(18)	-12.0(15)	9.5(13)	-4.8(13)
C21	27.5(13)	45.4(17)	54.5(17)	-2.7(14)	8.5(12)	-1.1(12)
C22	37.2(15)	47.6(19)	57.2(18)	-7.2(15)	13.9(14)	-8.1(13)
C23	49.1(17)	43.4(18)	50.6(17)	-2.4(14)	14.1(14)	-1.0(14)
C24	38.4(15)	48.6(18)	47.9(17)	8.5(14)	11.5(13)	4.7(13)
C25	35.6(15)	45.7(18)	51.4(17)	9.3(14)	6.4(13)	2.2(13)
C26	33.1(14)	38.2(16)	56.8(18)	1.5(14)	2.9(13)	-0.8(12)
C27	31(3)	54(5)	44(4)	10(4)	5(3)	0(3)
C28	58(5)	74(5)	42(4)	11(4)	10(4)	7(4)
C29	62(5)	64(5)	48(5)	-1(4)	13(4)	7(4)
C30	65(6)	82(7)	47(4)	-15(4)	12(5)	-16(5)
C31	106(8)	92(7)	58(5)	-6(5)	36(5)	-43(6)
C32	88(6)	60(5)	62(4)	1(4)	34(4)	-17(4)
C33	39(4)	63(6)	35(4)	2(4)	0(4)	-2(4)
C38	65(5)	56(5)	72(5)	2(4)	27(4)	-12(4)
C37	69(6)	82(7)	76(6)	7(5)	38(5)	-11(5)
C36	64(7)	119(11)	48(5)	8(6)	26(5)	-11(6)
C35	78(7)	115(11)	43(5)	-8(6)	14(5)	-29(8)
C34	57(6)	131(10)	37(5)	-22(6)	6(4)	-37(7)

Table *S25*: Bond Lengths in Åfor **9h**.

Atom	Atom	Length/Å
F1	C17	1.354(4)
O1	C20	1.223(4)
O2	C13	1.219(4)
N1	C1	1.436(4)
N1	C20	1.363(5)
C1	C2	1.328(4)
C1	C6	1.492(5)
C2	C3	1.510(4)
C3	C4	1.561(4)
C3	C7	1.535(4)
C4	C5	1.516(4)
C4	C13	1.524(4)
C5	C6	1.525(4)
C7	C8	1.390(4)
C7	C12	1.385(5)
C8	C9	1.387(4)
C9	C10	1.366(5)
C10	C11	1.390(4)
C11	C12	1.391(5)
C13	C14	1.491(4)
C14	C15	1.388(4)
C14	C19	1.399(4)

Atom	Atom	Length/Å
C15	C16	1.383(5)
C16	C17	1.381(5)
C17	C18	1.376(6)
C18	C19	1.373(5)
C20	C21	1.503(4)
C21	C22	1.387(5)
C21	C26	1.393(4)
C22	C23	1.388(4)
C23	C24	1.397(5)
C24	C25	1.387(5)
C24	C27	1.535(5)
C24	C33	1.480(5)
C25	C26	1.389(5)

Atom	Atom	Length/Å
C27	C28	1.3900
C27	C32	1.3900
C28	C29	1.3900
C29	C30	1.3900
C30	C31	1.3900
C31	C32	1.3900
C33	C38	1.3900
C33	C34	1.3900
C38	C37	1.3900
C37	C36	1.3900
C36	C35	1.3900
C35	C34	1.3900

Table S26: Bond Angles infor **9h**.

Atom	Atom	Atom	Angle/˚
C20	N1	C1	128.4(3)
N1	C1	C6	112.8(3)
C2	C1	N1	122.9(3)
C2	C1	C6	124.3(3)
C1	C2	C3	123.9(3)
C2	C3	C4	111.1(2)
C2	C3	C7	109.9(2)
C7	C3	C4	113.2(2)
C5	C4	C3	111.9(2)
C5	C4	C13	109.8(2)
C13	C4	C3	111.4(2)
C4	C5	C6	111.9(2)
C1	C6	C5	111.5(3)
C8	C7	C3	119.1(3)
C12	C7	C3	122.5(3)
C12	C7	C8	118.4(3)
C9	C8	C7	120.4(3)
C10	C9	C8	121.0(3)
C9	C10	C11	119.4(3)
C10	C11	C12	119.7(3)
C7	C12	C11	121.0(3)
O2	C13	C4	119.7(3)
O2	C13	C14	119.9(3)
C14	C13	C4	120.4(2)
C15	C14	C13	123.4(3)
C15	C14	C19	118.4(3)
C19	C14	C13	118.2(3)
C16	C15	C14	121.4(3)
C17	C16	C15	117.8(3)
F1	C17	C16	118.1(3)
F1	C17	C18	119.1(3)
C18	C17	C16	122.8(3)
C19	C18	C17	118.3(3)

Atom	Atom	Atom	Angle/˚
C18	C19	C14	121.3(3)
O1	C20	N1	123.6(3)
O1	C20	C21	121.5(3)
N1	C20	C21	114.9(3)
C22	C21	C20	123.6(3)
C22	C21	C26	118.8(3)
C26	C21	C20	117.6(3)
C21	C22	C23	120.5(3)
C22	C23	C24	121.3(3)
C23	C24	C27	116.7(4)
C23	C24	C33	125.1(4)
C25	C24	C23	117.4(3)
C25	C24	C27	125.6(3)
C25	C24	C33	117.2(4)
C24	C25	C26	121.8(3)
C25	C26	C21	120.1(3)
C28	C27	C24	121.2(4)
C28	C27	C32	120.0
C32	C27	C24	118.5(4)
C27	C28	C29	120.0
C30	C29	C28	120.0
C29	C30	C31	120.0
C30	C31	C32	120.0
C31	C32	C27	120.0
C38	C33	C24	124.5(5)
C38	C33	C34	120.0
C34	C33	C24	115.5(5)
C37	C38	C33	120.0
C36	C37	C38	120.0
C35	C36	C37	120.0
C36	C35	C34	120.0
C35	C34	C33	120.0

Table S27: Torsion Angles infor **9h**.

Atom	Atom	Atom	Atom	Angle/˚
F1	C17	C18	C19	179.6(3)
O1	C20	C21	C22	-155.0(3)

Atom	Atom	Atom	Atom	Angle/°
O1	C20	C21	C26	23.1(5)
O2	C13	C14	C15	177.2(3)
O2	C13	C14	C19	-1.4(4)
N1	C1	C2	C3	174.3(3)
N1	C1	C6	C5	-159.1(3)
N1	C20	C21	C22	26.3(5)
N1	C20	C21	C26	-155.6(3)
C1	N1	C20	O1	8.7(5)
C1	N1	C20	C21	-172.7(3)
C1	C2	C3	C4	14.3(4)
C1	C2	C3	C7	-111.8(3)
C2	C1	C6	C5	18.6(4)
C2	C3	C4	C5	-41.2(3)
C2	C3	C4	C13	-164.6(2)
C2	C3	C7	C8	-129.2(3)
C2	C3	C7	C12	50.7(4)
C3	C4	C5	C6	58.6(3)
C3	C4	C13	O2	102.1(3)
C3	C4	C13	C14	-78.6(3)
C3	C7	C8	C9	-178.6(3)
C3	C7	C12	C11	177.8(3)
C4	C3	C7	C8	105.9(3)
C4	C3	C7	C12	-74.2(4)
C4	C5	C6	C1	-45.6(4)
C4	C13	C14	C15	-2.0(4)
C4	C13	C14	C19	179.4(3)
C5	C4	C13	O2	-22.4(4)
C5	C4	C13	C14	156.8(3)
C6	C1	C2	C3	-3.1(5)
C7	C3	C4	C5	83.0(3)
C7	C3	C4	C13	-40.4(3)
C7	C8	C9	C10	0.2(5)
C8	C7	C12	C11	-2.2(5)
C8	C9	C10	C11	-1.1(5)
C9	C10	C11	C12	0.4(6)
C10	C11	C12	C7	1.3(6)
C12	C7	C8	C9	1.5(5)
C13	C4	C5	C6	-177.2(3)
C13	C14	C15	C16	-177.7(3)
C13	C14	C19	C18	177.7(3)
C14	C15	C16	C17	-0.3(5)
C15	C14	C19	C18	-1.0(5)
C15	C16	C17	F1	-179.7(3)
C15	C16	C17	C18	-0.1(5)
C16	C17	C18	C19	0.0(6)
C17	C18	C19	C14	0.5(5)
C19	C14	C15	C16	0.9(4)
C20	N1	C1	C2	33.1(5)
C20	N1	C1	C6	-149.2(3)
C20	C21	C22	C23	179.9(3)
C20	C21	C26	C25	179.1(3)
C21	C22	C23	C24	0.4(5)
C22	C21	C26	C25	-2.6(5)
C22	C23	C24	C25	-1.6(5)
C22	C23	C24	C27	-176.5(4)
C22	C23	C24	C33	172.0(5)
C23	C24	C25	C26	0.8(5)
C23	C24	C27	C28	-31.1(6)
C23	C24	C27	C32	154.8(4)
C23	C24	C33	C38	-154.5(5)
C23	C24	C33	C34	26.7(7)

Atom	Atom	Atom	Atom	Angle/°
C24	C25	C26	C21	1.4(5)
C24	C27	C28	C29	-174.1(6)
C24	C27	C32	C31	174.3(6)
C24	C33	C38	C37	-178.7(7)
C24	C33	C34	C35	178.8(7)
C25	C24	C27	C28	154.6(4)
C25	C24	C27	C32	-19.6(6)
C25	C24	C33	C38	19.1(7)
C25	C24	C33	C34	-159.7(4)
C26	C21	C22	C23	1.8(5)
C27	C24	C25	C26	175.1(4)
C27	C28	C29	C30	0.0
C28	C27	C32	C31	0.0
C28	C29	C30	C31	0.0
C29	C30	C31	C32	0.0
C30	C31	C32	C27	0.0
C32	C27	C28	C29	0.0
C33	C24	C25	C26	-173.4(4)
C33	C38	C37	C36	0.0
C38	C33	C34	C35	0.0
C38	C37	C36	C35	0.0
C37	C36	C35	C34	0.0
C36	C35	C34	C33	0.0
C34	C33	C38	C37	0.0

Table S28: Hydrogen Fractional Atomic Coordinates (×10[4]) and Equivalent Isotropic Displacement Parameters (Å[2]×10[3]) for **9h**. U_{eq} is defined as 1/3 of the trace of the orthogonalised U_{ij}.

Atom	x	y	z	U_{eq}
H2	3400.69	7274.57	3600.12	47
H3	2826.53	7301.36	5004.1	43
H4	3720.74	4681.41	5760.25	42
H5A	3110.23	991.45	4615.85	51
H5B	3834.01	680.28	5431.07	51
H6A	4942.08	2766.93	4778.32	52
H6B	4518.7	1052.54	4037.12	52
H8	1260.42	7818.91	4989.37	48
H9	-237.61	7027.07	4467.44	55
H10	-552.92	3959.92	3520.1	53
H11	655.46	1691.14	3046.76	66
H12	2164.29	2525.09	3544.11	65
H15	3131.07	7382.35	6557.91	49
H16	2742.69	9627.37	7744.75	56
H18	834.14	4787.81	8295.89	67
H19	1225.55	2581.67	7110.23	55
H22	4861.9	2907.37	1532.31	56
H23	5620.09	2361.34	270.33	57
H25	6891.9	8569.62	644.7	53
H26	6108.2	9176.37	1888.6	51
H28	6707.19	1362.22	-503.34	70
H29	7475.45	717.84	-1764.84	69
H30	7996.81	3891.08	-2551.76	77
H31	7749.9	7708.73	-2077.18	101
H32	6981.64	8353.16	-815.68	82
H38	8001.69	7290.9	-37.37	76
H37	8851.55	6626.52	-1246.75	89
H36	8308.59	4013.57	-2326.25	91

Atom	x	y	z	U_{eq}
H35	6915.75	2064.98	-2196.39	94
H34	6065.88	2729.33	-987.01	90
H1	4820(30)	3120(80)	2940(30)	56(11)

Table S29: Atomic Occupancies for all atoms that are not fully occupied in **9h**.

Atom	Occupancy	Atom	Occupancy	Atom	Occupancy
C27	0.523(7)	C31	0.523(7)	H37	0.477(7)
C28	0.523(7)	H31	0.523(7)	C36	0.477(7)
H28	0.523(7)	C32	0.523(7)	H36	0.477(7)
C29	0.523(7)	H32	0.523(7)	C35	0.477(7)
H29	0.523(7)	C33	0.477(7)	H35	0.477(7)
C30	0.523(7)	C38	0.477(7)	C34	0.477(7)
H30	0.523(7)	H38	0.477(7)	H34	0.477(7)
		C37	0.477(7)		

Table S30: Solvent masking (PLATON/SQUEEZE) information for **9h**.

No	x	y	z	V	e	Content
1	0.000	-0.678	0.000	107.6	29.4	1C3H6O

R_1=3.58%

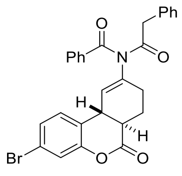

16b

Crystal Data and Experimental of **16b** (CCDC number **2324851**).

The ellipsoids are displayed at 50% probability level.

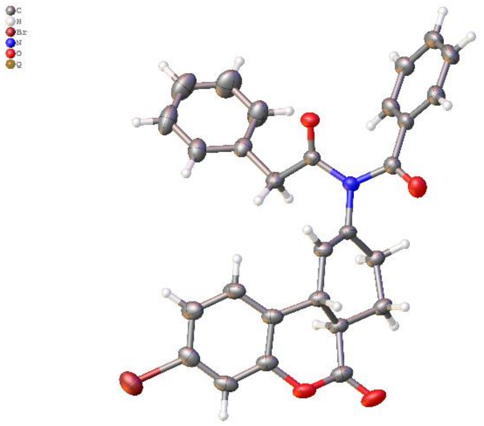

Experimental. The compound of **16b** was crystallized from MeOH/Pentane (v:v = 1:5) as a colorless crystal. A suitable crystal with dimensions 0.54 × 0.53 × 0.24 mm^3 was selected and mounted on a SuperNova, Dual, Cu at home/near, AtlasS2 diffractometer. The crystal was kept at a steady T = 140.00(10) K during data collection. The structure was solved with the ShelXS (Sheldrick, 2008) solution program using heavy methods and by using Olex2 1.5 (Dolomanov et al., 2009) as the graphical interface. The model was refined with ShelXL 2019/3 (Sheldrick, 2015) using full matrix least squares minimisation on F^2.[10-11]

Crystal Data. $C_{29}H_{24}BrNO_{4.25}$, M_r = 534.40, tetragonal, $P4_2/n$ (No. 86), a = 18.77145(10) Å, b = 18.77145(10) Å, c = 13.80505(11) Å, $\alpha = \beta = \gamma = 90°$, V = 4864.45(7) Å^3, T = 140.00(10) K, Z = 8, Z' = 1, μ(Cu K_α) = 2.613, 24832 reflections measured, 4784 unique (R_{int} = 0.0190) which were used in all calculations. The final wR_2 was 0.0911 (all data) and R_1 was 0.0358 (I≥2 σ(I)).

**Compound**	**16b**
Formula	$C_{29}H_{24}BrNO_{4.25}$
$D_{calc.}$/ g cm^{-3}	1.459
μ/mm^{-1}	2.613
Formula Weight	534.40
Colour	clear pale colourless
Shape	prism
Size/mm^3	0.54×0.53×0.24
T/K	140.00(10)
Crystal System	tetragonal
Space Group	$P4_2/n$
a/Å	18.77145(10)
b/Å	18.77145(10)
c/Å	13.80505(11)
α/°	90
β/°	90
γ/°	90
V/Å^3	4864.45(7)
Z	8
Z'	1
Wavelength/Å	1.54184
Radiation type	Cu K_α
Θ_{min}/°	3.330
Θ_{max}/°	72.723
Measured Refl's.	24832
Indep't Refl's	4784
Refl's I≥2 σ(I)	4664
R_{int}	0.0190
Parameters	307
Restraints	0
Largest Peak	0.560
Deepest Hole	-0.554
GooF	1.097
wR_2 (all data)	0.0911
wR_2	0.0907
R_1 (all data)	0.0365
R_1	0.0358

Structure Quality Indicators

**Reflections:**	d min (CuKα) 2Θ=145.4° 0.81	I/σ(I) 86.5	Rint m=5.29 1.90%	Full 135.4° 99% to 145.4° 100
**Refinement:**	Shift -0.001	Max Peak 0.6	Min Peak -0.6	GooF 1.097

A clear pale colourless prism-shaped crystal with dimensions 0.54 × 0.53 × 0.24 mm^3 was mounted. Data were collected using a SuperNova, Dual, Cu at home/near, AtlasS2 diffractometer operating at T = 140.00(10) K.

Data were measured using ω scans with Cu K$_\alpha$ radiation. The diffraction pattern was indexed and the total number of runs and images was based on the strategy calculation from the program CrysAlisPro 1.171.42.90a (Rigaku OD, 2023). The maximum resolution that was achieved was Θ = 72.723° (0.81 Å).

The unit cell was refined using CrysAlisPro 1.171.42.90a (Rigaku OD, 2023) on 16725 reflections, 67% of the observed reflections.

Data reduction, scaling and absorption corrections were performed using CrysAlisPro 1.171.42.90a (Rigaku OD, 2023). The final completeness is 100.00 % out to 72.723° in Θ. A gaussian absorption correction was performed using CrysAlisPro 1.171.42.90a (Rigaku Oxford Diffraction, 2023). The numerical absorption correction was based on gaussian integration over a multifaceted crystal model. The empirical absorption correction was done using spherical harmonics, implemented in SCALE3 ABSPACK scaling algorithm. The absorption coefficient μ of this crystal is 2.613 mm^{-1} at this wavelength (λ = 1.54184Å) and the minimum and maximum transmissions are 0.175 and 1.000.

The structure was solved and the space group $P4_2/n$ (# 86) determined by the ShelXS (Sheldrick, 2008) structure solution program using heavy methods and refined by full matrix least squares minimisation on $\boldsymbol{F}^2$ using version 2019/3 of ShelXL (Sheldrick, 2015). All non-hydrogen atoms were refined anisotropically. Hydrogen atom positions were calculated geometrically and refined using the riding model.

There is a single formula unit in the asymmetric unit, which is represented by the reported sum formula. In other words: Z is 8 and Z' is 1. The moiety formula is $C_{28}H_{22}BrNO_4$, 0.25[C_4H_8O].

Data Plots: Diffraction Data

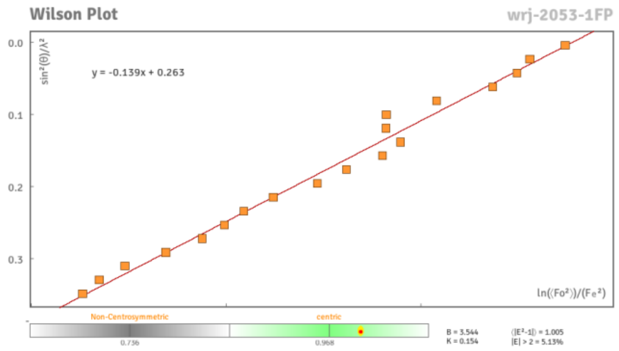

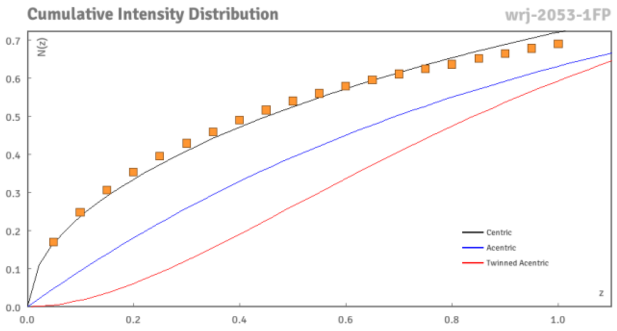

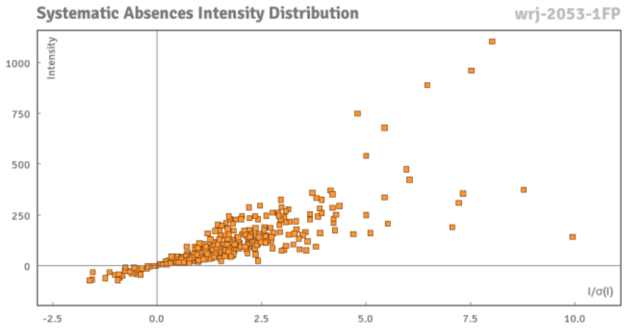

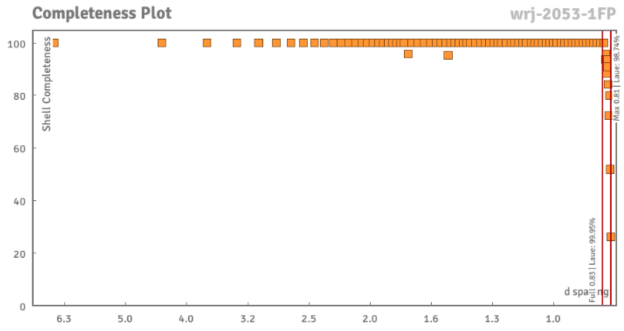

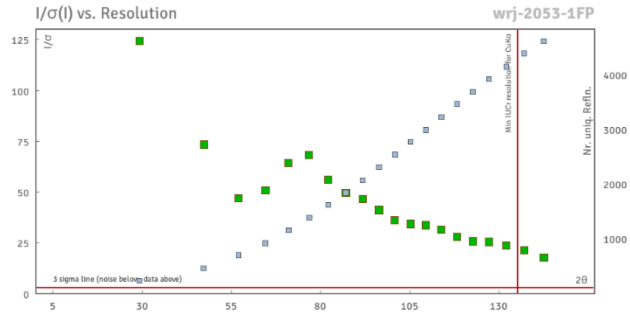

Data Plots: Refinement and Data

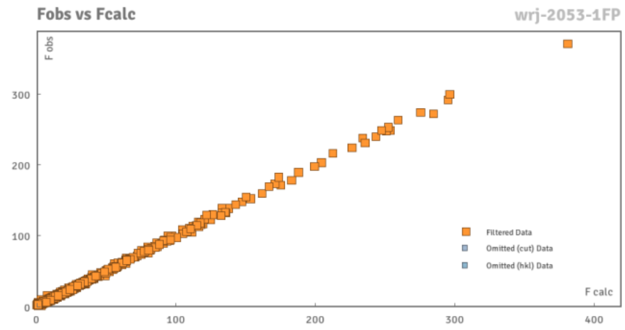

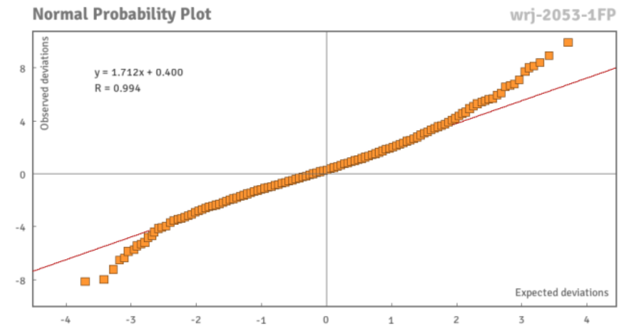

Reflection Statistics

Total reflections (after filtering)	25331	Unique reflections	4784
Completeness	0.987	Mean I/σ	45.27
hkl_{max} collected	(22, 23, 16)	hkl_{min} collected	(-18, -17, -17)
hkl_{max} used	(16, 23, 17)	hkl_{min} used	(-15, 0, 0)
Lim d_{max} collected	100.0	Lim d_{min} collected	0.77
d_{max} used	13.81	d_{min} used	0.81
Friedel pairs	433	Friedel pairs merged	1
Inconsistent equivalents	5	R_{int}	0.019
R_{sigma}	0.0116	Intensity transformed	0
Omitted reflections	0	Omitted by user (OMIT hkl)	0
Multiplicity	(9717, 3996, 1316, 517, 227, 75, 3)	Maximum multiplicity	18
Removed systematic absences	499	Filtered off (Shel/OMIT)	0

Table S31: Fractional Atomic Coordinates (×10^4) and Equivalent Isotropic Displacement Parameters (Å^2×10^3) for **16b.** U_{eq} is defined as 1/3 of the trace of the orthogonalised U_{ij}.

Atom	x	y	z	U_{eq}
Br1	3487.3(2)	4020.8(2)	10333.0(2)	51.72(10)

Atom	x	y	z	U_{eq}
O1	5592.0(7)	4785.9(7)	4060.4(9)	32.0(3)
O2	7449.3(8)	4994.8(9)	5248.9(10)	42.7(3)
O3	7339.7(9)	3575.4(10)	9507.9(11)	52.6(4)
O4	6227.1(8)	3902.2(8)	9769.2(9)	40.0(3)
N1	6407.5(8)	4385.8(8)	5154.8(10)	28.7(3)
C1	5731.9(9)	4382.8(10)	4716.0(12)	27.3(4)
C2	5218.5(10)	3830.1(10)	5102.2(14)	34.0(4)
C3	4627.4(10)	3651.0(10)	4403.1(15)	34.6(4)
C4	3920.9(11)	3704.6(12)	4672.5(18)	43.9(5)
C5	3381.5(12)	3529.7(14)	4024(2)	58.4(7)
C6	3546.6(14)	3298.5(14)	3107(2)	61.5(7)
C7	4243.1(16)	3235.3(17)	2834(2)	67.8(8)
C8	4780.5(13)	3416.7(14)	3476.1(19)	53.3(6)
C9	7005.9(10)	4722.8(11)	4733.4(13)	32.5(4)
C10	7097.4(10)	4668.9(11)	3663.5(13)	32.7(4)
C11	6835.7(10)	4093.1(11)	3131.3(14)	35.2(4)
C12	6947.7(11)	4061.6(12)	2139.8(14)	39.5(4)
C13	7328.1(11)	4592.4(13)	1683.2(15)	43.9(5)
C14	7612.6(11)	5148.5(13)	2210.2(16)	46.3(5)
C15	7499.9(11)	5189.7(12)	3207.1(15)	39.8(4)
C16	6490.0(9)	4204.2(10)	6168.8(12)	28.9(4)
C17	7002.1(10)	3609.1(10)	6378.5(13)	32.5(4)
C18	7238.6(10)	3623.3(11)	7438.5(14)	35.1(4)
C19	6597.9(10)	3731.1(10)	8093.8(13)	31.8(4)
C20	6774.2(12)	3717.4(11)	9158.2(14)	38.5(4)
C21	5566.1(11)	4106.9(10)	9381.8(13)	34.4(4)
C22	4978.6(12)	4011.1(10)	9969.0(14)	37.5(4)
C23	4323.9(12)	4191.4(11)	9590.8(14)	39.0(4)
C24	4251.4(12)	4471.4(12)	8668.8(15)	42.0(5)
C25	4856.0(12)	4581.1(11)	8109.9(14)	38.8(4)
C26	5524.4(11)	4398.1(10)	8455.7(13)	33.1(4)
C27	6212.1(11)	4436.9(10)	7892.8(13)	32.2(4)
C28	6142.3(10)	4578.7(10)	6831.8(13)	31.4(4)

Table S32: Anisotropic Displacement Parameters (×10^4) for **16b.** The anisotropic displacement factor exponent takes the form: $-2\pi^2[h^2a^{*2} \times U_{11} + \ldots + 2hka^* \times b^* \times U_{12}]$

Atom	U_{11}	U_{22}	U_{33}	U_{23}	U_{13}	U_{12}
Br1	58.53(16)	52.18(15)	44.46(15)	-5.0(1)	16.06(10)	-3.86(10)
O1	34.7(7)	37.0(7)	24.4(6)	5.2(5)	-0.4(5)	2.6(5)
O2	34.4(7)	57.7(9)	36.0(7)	-7.0(6)	1.0(6)	-7.5(6)
O3	53.3(9)	71.1(11)	33.4(8)	1.9(7)	-18.0(7)	10.7(8)
O4	51.3(8)	48.2(8)	20.5(6)	1.1(5)	-8.2(6)	3.2(7)
N1	28.5(7)	37.0(8)	20.5(7)	1.0(6)	0.9(6)	0.5(6)
C1	29.3(8)	32.5(9)	20.0(8)	-1.1(6)	2.0(6)	3.4(7)
C2	32.9(9)	37.0(10)	32.2(9)	8.9(8)	-3.2(8)	-0.7(7)
C3	32.5(9)	29.2(9)	42.2(10)	3.3(8)	-4.4(8)	1.4(7)
C4	33.6(10)	41.0(11)	57.1(13)	-1.5(9)	0.8(9)	-2.3(8)
C5	33.2(11)	50.8(13)	91(2)	-6.6(13)	-9.8(12)	-1.3(9)
C6	52.8(14)	54.0(14)	77.9(18)	-13.8(13)	-26.0(13)	-2.5(11)
C7	63.7(17)	76.9(19)	62.8(16)	-28.0(14)	-13.6(13)	-4.5(14)
C8	40.6(12)	62.5(15)	56.9(14)	-20.4(12)	-3.5(10)	1.5(10)
C9	29.6(9)	39.7(10)	28.3(9)	-1.0(7)	2.7(7)	1.8(7)
C10	27.8(8)	43.1(10)	27.3(9)	0.7(8)	4.0(7)	3.2(7)
C11	34.3(9)	40.9(10)	30.3(9)	-0.8(8)	6.4(7)	2.6(8)
C12	39.0(10)	49.0(11)	30.4(10)	-5.1(8)	3.6(8)	7.2(9)
C13	38.0(10)	65.3(14)	28.3(9)	3.1(9)	8.0(8)	9.7(10)

Atom	U_{11}	U_{22}	U_{33}	U_{23}	U_{13}	U_{12}
C14	36.4(10)	61.0(14)	41.6(11)	13.1(10)	11.3(9)	-0.6(10)
C15	32.9(10)	47.4(11)	39.0(11)	3.6(9)	2.9(8)	-3.0(8)
C16	30.5(9)	34.8(9)	21.4(8)	2.1(7)	-3.6(7)	-0.4(7)
C17	32.8(9)	37.4(10)	27.3(9)	-1.8(7)	-3.9(7)	4.5(7)
C18	34.5(9)	40.4(10)	30.4(9)	0.2(8)	-8.5(8)	4.5(8)
C19	36.9(9)	33.1(9)	25.2(9)	0.2(7)	-7.3(7)	2.8(7)
C20	47.6(11)	39.9(10)	28.0(9)	0.3(8)	-10.3(8)	1.5(9)
C21	47.9(11)	31.6(9)	23.7(8)	-3.0(7)	-4.9(8)	2.5(8)
C22	59.6(12)	30.0(9)	23.0(8)	-1.8(7)	1.5(8)	0.0(8)
C23	49.6(12)	35.1(10)	32.2(10)	-6.3(8)	8.8(8)	0.6(9)
C24	46.7(11)	47.0(11)	32.2(10)	-3.1(9)	2.3(9)	13.1(9)
C25	49.8(11)	42.7(11)	23.9(9)	-0.2(8)	1.0(8)	13.7(9)
C26	45.8(11)	31.2(9)	22.2(8)	-2.2(7)	-1.0(7)	5.1(8)
C27	42.8(10)	30.8(9)	23.0(8)	-1.6(7)	-3.9(7)	3.1(8)
C28	36.6(9)	32.5(9)	25.1(8)	2.4(7)	-1.5(7)	4.2(7)

Table S33: Bond Lengths in Åfor **16b**.

Atom	Atom	Length/Å
Br1	C23	1.902(2)
O1	C1	1.209(2)
O2	C9	1.208(2)
O3	C20	1.196(3)
O4	C20	1.373(3)
O4	C21	1.405(2)
N1	C1	1.406(2)
N1	C9	1.414(2)
N1	C16	1.449(2)
C1	C2	1.513(3)
C2	C3	1.509(3)
C3	C4	1.381(3)
C3	C8	1.383(3)
C4	C5	1.391(3)
C5	C6	1.374(4)
C6	C7	1.366(4)
C7	C8	1.385(4)
C9	C10	1.490(2)
C10	C11	1.396(3)

Atom	Atom	Length/Å
C10	C15	1.387(3)
C11	C12	1.386(3)
C12	C13	1.378(3)
C13	C14	1.380(3)
C14	C15	1.395(3)
C16	C17	1.502(2)
C16	C28	1.326(3)
C17	C18	1.529(2)
C18	C19	1.518(3)
C19	C20	1.506(2)
C19	C27	1.535(2)
C21	C22	1.380(3)
C21	C26	1.393(3)
C22	C23	1.377(3)
C23	C24	1.384(3)
C24	C25	1.388(3)
C25	C26	1.386(3)
C26	C27	1.508(3)
C27	C28	1.494(2)

Table S34: Bond Angles in°for **16b**.

Atom	Atom	Atom	Angle/°
C20	O4	C21	119.72(14)
C1	N1	C9	122.76(14)
C1	N1	C16	120.79(14)
C9	N1	C16	114.69(14)
O1	C1	N1	121.08(16)
O1	C1	C2	123.70(16)
N1	C1	C2	115.19(15)
C3	C2	C1	113.32(15)
C4	C3	C2	121.19(19)
C4	C3	C8	118.2(2)
C8	C3	C2	120.65(19)
C3	C4	C5	120.6(2)

Atom	Atom	Atom	Angle/°
C6	C5	C4	120.2(2)
C7	C6	C5	119.8(2)
C6	C7	C8	120.0(3)
C3	C8	C7	121.2(2)
O2	C9	N1	119.59(16)
O2	C9	C10	122.21(17)
N1	C9	C10	117.95(16)
C11	C10	C9	122.25(18)
C15	C10	C9	117.71(18)
C15	C10	C11	119.89(18)
C12	C11	C10	119.96(19)
C13	C12	C11	119.9(2)

Atom	Atom	Atom	Angle/°
C12	C13	C14	120.41(19)
C13	C14	C15	120.2(2)
C10	C15	C14	119.5(2)
N1	C16	C17	115.44(15)
C28	C16	N1	119.31(16)
C28	C16	C17	125.19(16)
C16	C17	C18	110.93(15)
C19	C18	C17	110.03(15)
C18	C19	C27	112.39(16)
C20	C19	C18	113.89(16)
C20	C19	C27	107.14(15)
O3	C20	O4	118.17(18)
O3	C20	C19	126.3(2)
O4	C20	C19	115.50(17)
C22	C21	O4	116.52(17)
C22	C21	C26	123.04(19)
C26	C21	O4	120.43(18)
C23	C22	C21	117.27(18)
C22	C23	Br1	119.41(15)
C22	C23	C24	122.0(2)
C24	C23	Br1	118.57(17)
C23	C24	C25	119.2(2)
C26	C25	C24	120.80(18)
C21	C26	C27	116.34(17)
C25	C26	C21	117.68(19)
C25	C26	C27	125.83(17)
C26	C27	C19	105.60(15)
C28	C27	C19	111.86(15)
C28	C27	C26	116.01(16)
C16	C28	C27	122.61(17)

Table S35: Torsion Angles in for **16b**.

Atom	Atom	Atom	Atom	Angle/°
Br1	C23	C24	C25	-178.33(16)
O1	C1	C2	C3	19.8(3)
O2	C9	C10	C11	146.2(2)
O2	C9	C10	C15	-29.3(3)
O4	C21	C22	C23	-178.32(17)
O4	C21	C26	C25	179.22(17)
O4	C21	C26	C27	3.4(3)
N1	C1	C2	C3	-158.26(16)
N1	C9	C10	C11	-28.1(3)
N1	C9	C10	C15	156.44(18)
N1	C16	C17	C18	161.05(16)
N1	C16	C28	C27	-178.50(17)
C1	N1	C9	O2	146.52(19)
C1	N1	C9	C10	-39.0(3)
C1	N1	C16	C17	124.35(18)
C1	N1	C16	C28	-58.2(2)
C1	C2	C3	C4	-125.1(2)
C1	C2	C3	C8	56.0(3)
C2	C3	C4	C5	-179.2(2)
C2	C3	C8	C7	178.5(2)
C3	C4	C5	C6	0.3(4)
C4	C3	C8	C7	-0.4(4)
C4	C5	C6	C7	0.5(4)
C5	C6	C7	C8	-1.2(5)
C6	C7	C8	C3	1.2(5)
C8	C3	C4	C5	-0.3(3)
C9	N1	C1	O1	-15.2(3)
C9	N1	C1	C2	162.86(17)
C9	N1	C16	C17	-70.4(2)
C9	N1	C16	C28	107.1(2)
C9	C10	C11	C12	-178.69(18)
C9	C10	C15	C14	178.43(19)
C10	C11	C12	C13	1.1(3)
C11	C10	C15	C14	2.8(3)
C11	C12	C13	C14	1.4(3)
C12	C13	C14	C15	-1.9(3)
C13	C14	C15	C10	-0.2(3)
C15	C10	C11	C12	-3.3(3)

Atom	Atom	Atom	Atom	Angle/°
C16	N1	C1	O1	148.86(17)
C16	N1	C1	C2	-33.1(2)
C16	N1	C9	O2	-18.4(3)
C16	N1	C9	C10	156.01(16)
C16	C17	C18	C19	45.6(2)
C17	C16	C28	C27	-1.3(3)
C17	C18	C19	C20	177.24(16)
C17	C18	C19	C27	-60.7(2)
C18	C19	C20	O3	-6.5(3)
C18	C19	C20	O4	172.07(17)
C18	C19	C27	C26	169.56(15)
C18	C19	C27	C28	42.5(2)
C19	C27	C28	C16	-11.6(3)
C20	O4	C21	C22	154.90(18)
C20	O4	C21	C26	-25.9(3)
C20	C19	C27	C26	-64.61(19)
C20	C19	C27	C28	168.34(17)
C21	O4	C20	O3	176.81(19)
C21	O4	C20	C19	-1.9(3)
C21	C22	C23	Br1	176.35(14)
C21	C22	C23	C24	-1.3(3)
C21	C26	C27	C19	41.1(2)
C21	C26	C27	C28	165.58(17)
C22	C21	C26	C25	-1.6(3)
C22	C21	C26	C27	-177.46(17)
C22	C23	C24	C25	-0.7(3)
C23	C24	C25	C26	1.6(3)
C24	C25	C26	C21	-0.5(3)
C24	C25	C26	C27	174.93(19)
C25	C26	C27	C19	-134.4(2)
C25	C26	C27	C28	-9.9(3)
C26	C21	C22	C23	2.5(3)
C26	C27	C28	C16	-132.9(2)
C27	C19	C20	O3	-131.4(2)
C27	C19	C20	O4	47.1(2)
C28	C16	C17	C18	-16.3(3)

Table S36: Hydrogen Fractional Atomic Coordinates (×10[4]) and Equivalent Isotropic Displacement Parameters (Å[2]×10[3]) for **16b**. U_{eq} is defined as 1/3 of the trace of the orthogonalised U_{ij}.

Atom	x	y	z	U_{eq}
H2A	5486.26	3389.9	5255.31	41
H2B	5006.44	4008.36	5712.47	41
H4	3802.27	3862.23	5306.19	53
H5	2897.14	3570.38	4215.75	70
H6	3177.23	3182.72	2663.71	74
H7	4358.99	3067.04	2204.57	81
H8	5263.33	3379.46	3276.01	64
H11	6581.11	3723.18	3448.57	42
H12	6762.39	3674.39	1775.22	47
H13	7394.83	4575.27	1001.42	53
H14	7886.29	5504.48	1892.79	56
H15	7697.17	5571.48	3570.67	48
H17A	7424.38	3655.33	5953.56	39
H17B	6770.96	3146.81	6235.71	39
H18A	7477.37	3168.95	7603.4	42
H18B	7583.81	4015.17	7538.51	42

Atom	x	y	z	U_{eq}
H19	6254.48	3335.27	7963.48	38
H22	5023.94	3828.41	10607.72	45
H24	3793.53	4586.96	8421.53	50
H25	4811.06	4784.25	7482.56	47
H27	6505.45	4828.75	8178.44	39
H28	5836.7	4952.09	6624.2	38

Table S37: Solvent masking (PLATON/SQUEEZE) information for **16b**.

No	x	y	z	V	e	Content
1	0.250	0.250	0.750	189.0	40.7	1thf
2	0.750	0.750	0.250	189.0	40.7	1thf

7. Copies of the NMR spectra

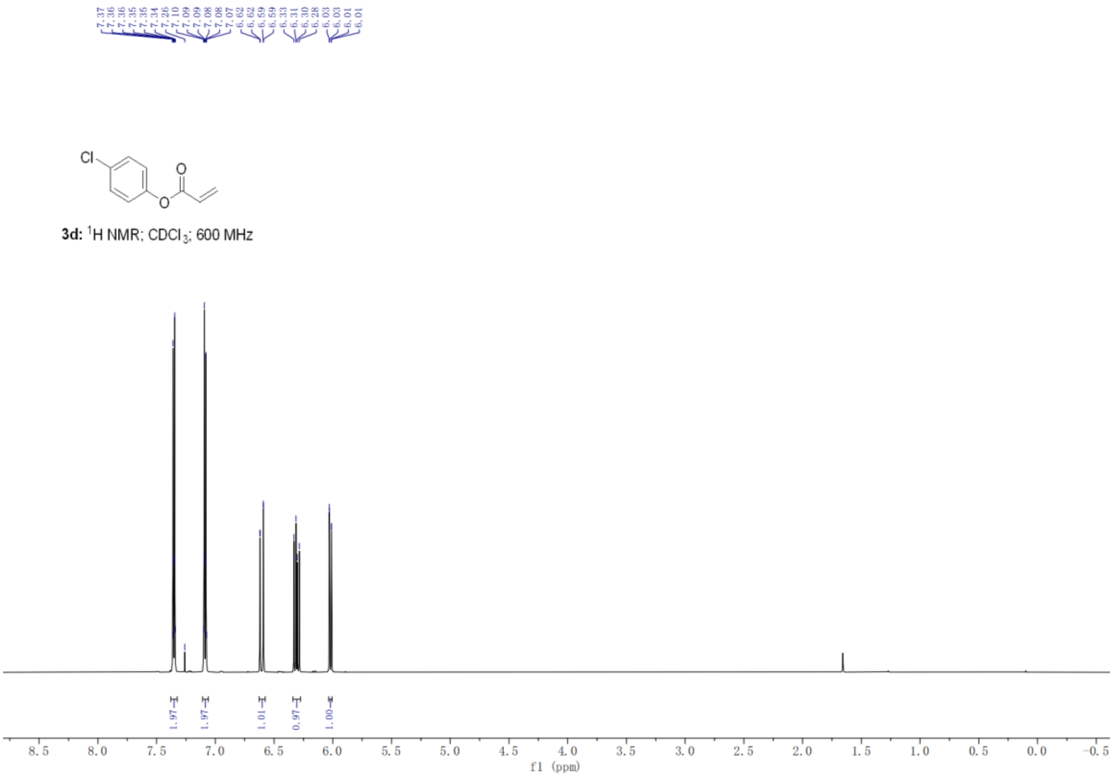

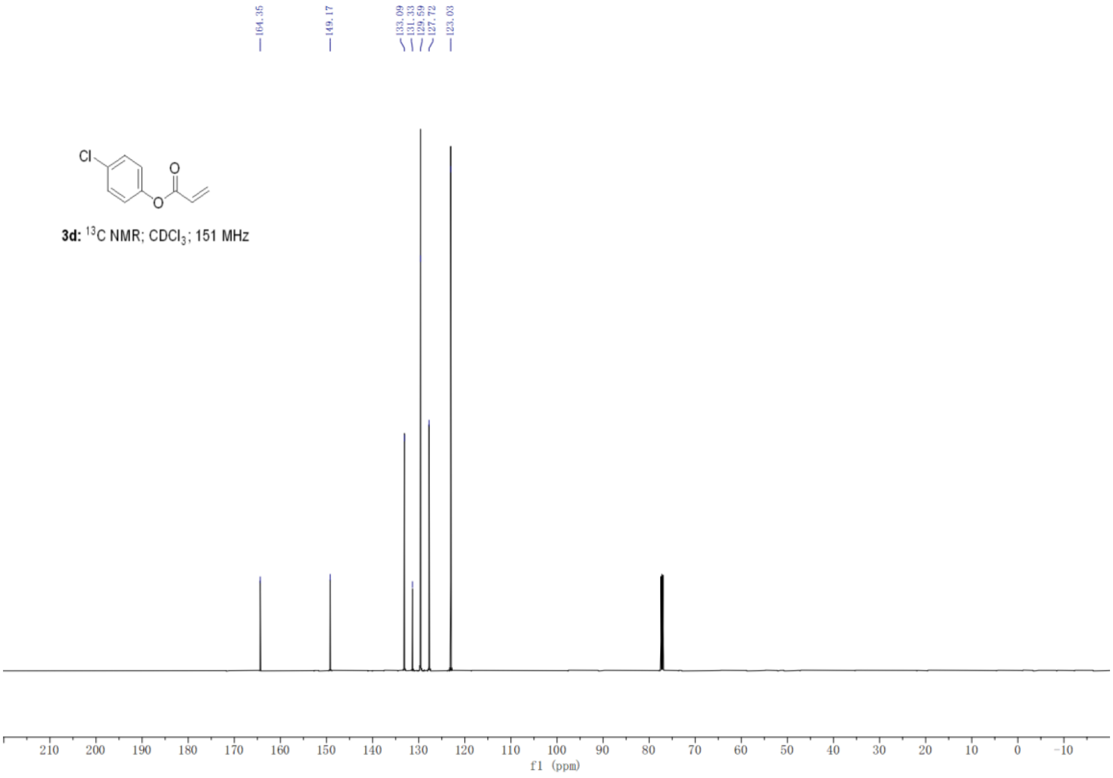

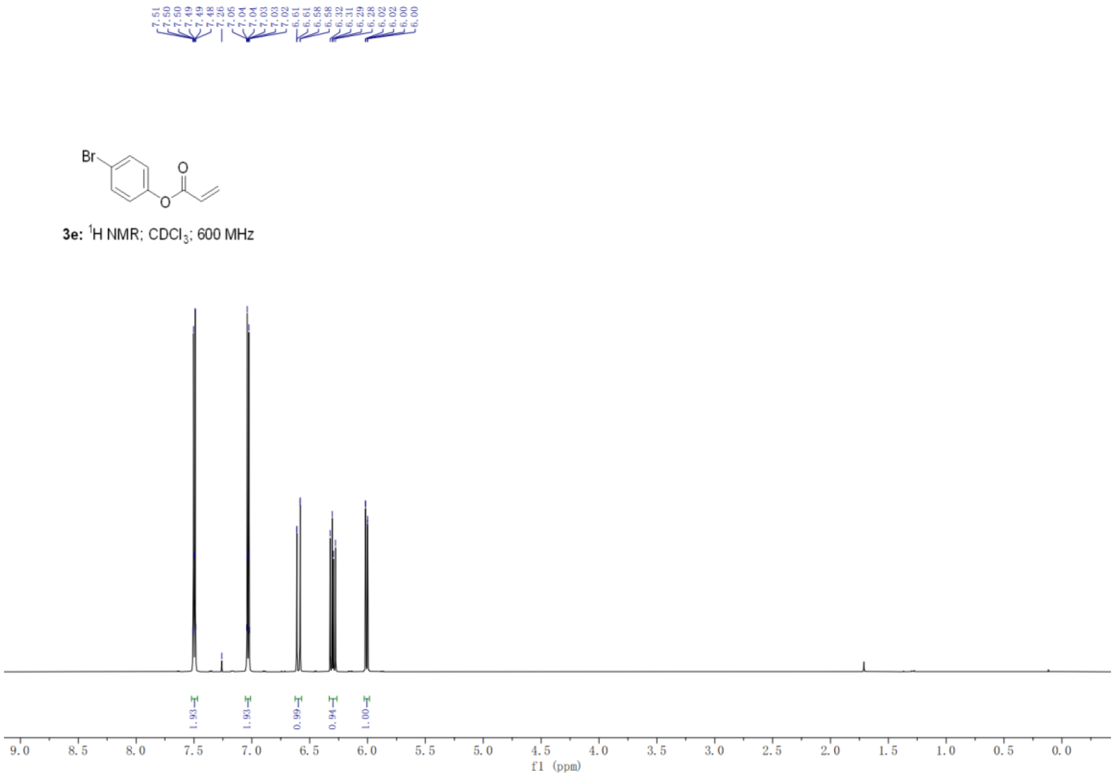
7.51
7.50
7.49
7.49
7.48
7.26
7.05
7.04
7.04
7.03
7.03
7.02
6.61
6.61
6.58
6.58
6.32
6.31
6.29
6.28
6.02
6.02
6.00
6.00
Br
O
O
3e: ¹H NMR; CDCl3; 600 MHz
1.93
1.93
0.99
0.94
1.00
f1 (ppm)

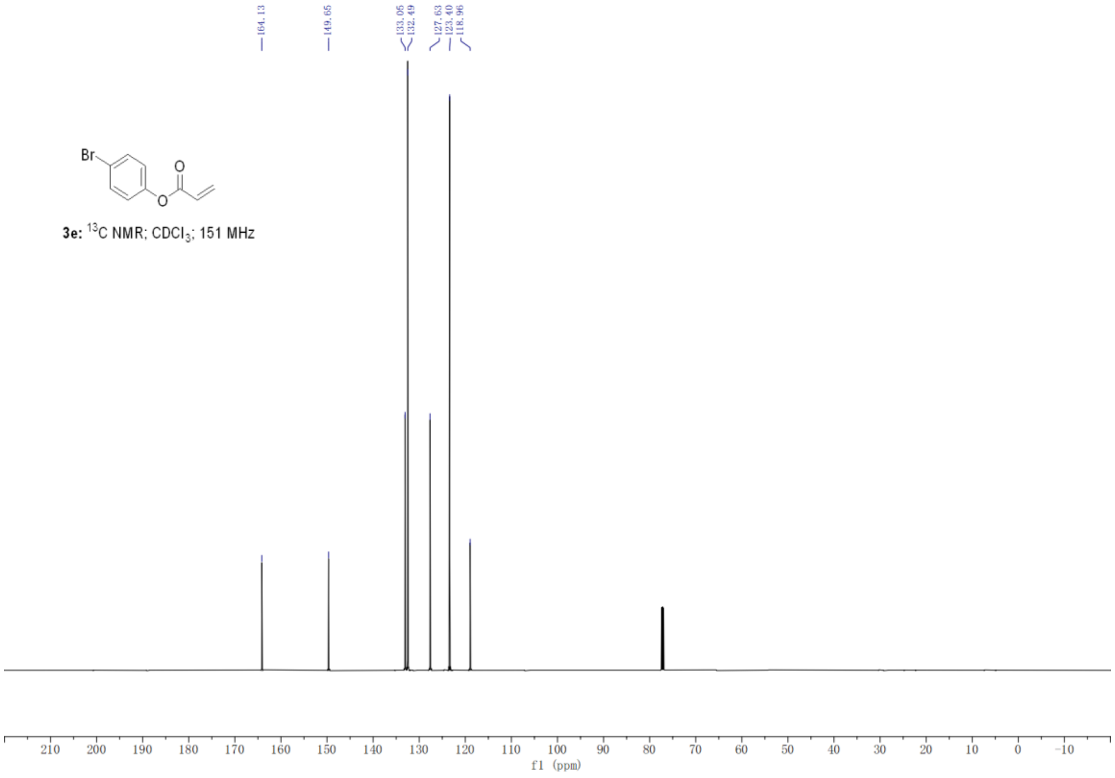
164.13
149.65
133.05
132.49
127.63
123.40
118.96
Br
O
O
3e: ¹³C NMR; CDCl3; 151 MHz
f1 (ppm)

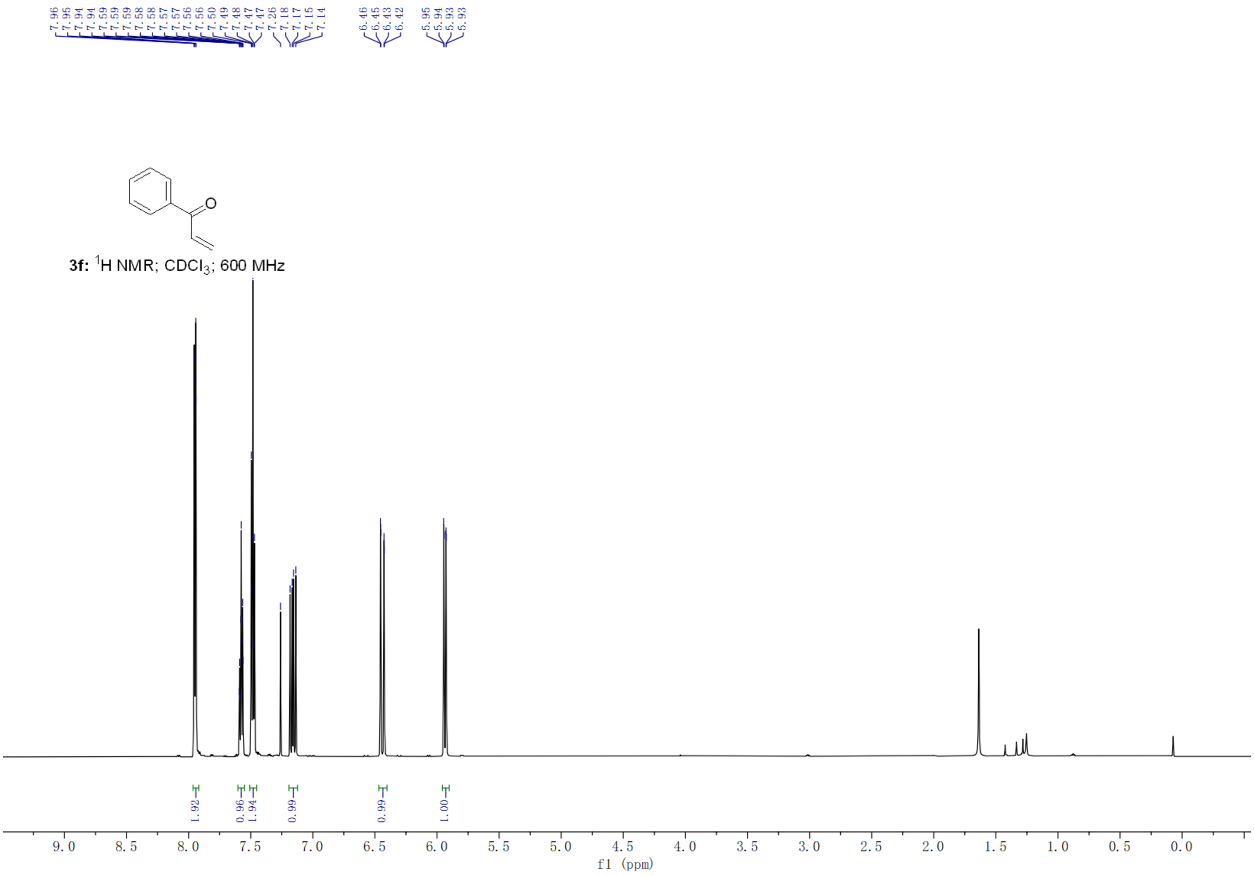
7.96
7.95
7.94
7.94
7.59
7.59
7.58
7.58
7.57
7.57
7.56
7.56
7.50
7.49
7.48
7.47
7.47
7.26
7.18
7.17
7.15
7.14
6.46
6.45
6.43
6.42
5.95
5.94
5.93
5.93
O
3f: 1H NMR; CDCl3; 600 MHz
1.92
0.96
1.94
0.99
0.99
1.00
9.0
8.5
8.0
7.5
7.0
6.5
6.0
5.5
5.0
4.5
4.0
3.5
3.0
2.5
2.0
1.5
1.0
0.5
0.0
f1 (ppm)

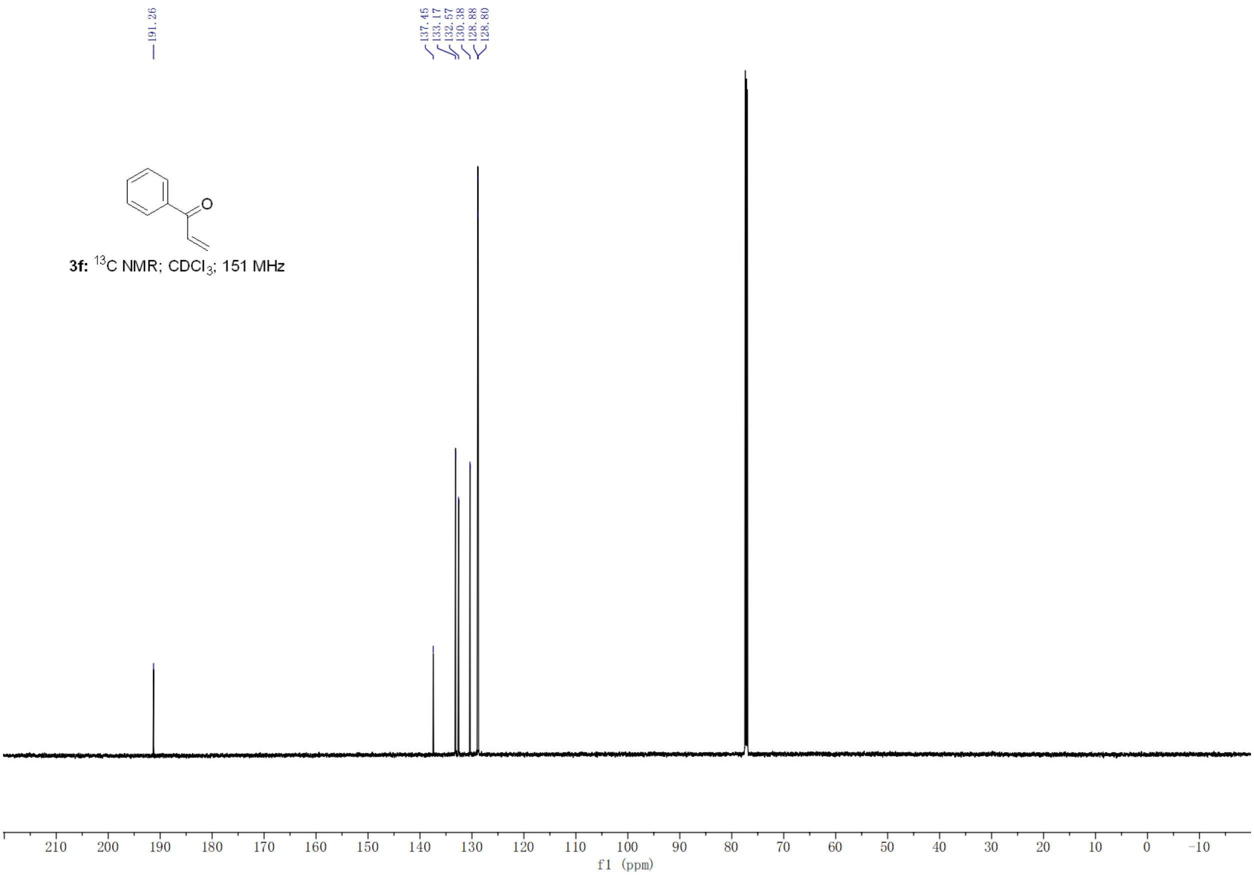
191.26
137.45
133.17
132.57
130.38
128.88
128.80
O
3f: 13C NMR; CDCl3; 151 MHz
210
200
190
180
170
160
150
140
130
120
110
100
90
80
70
60
50
40
30
20
10
0
-10
f1 (ppm)

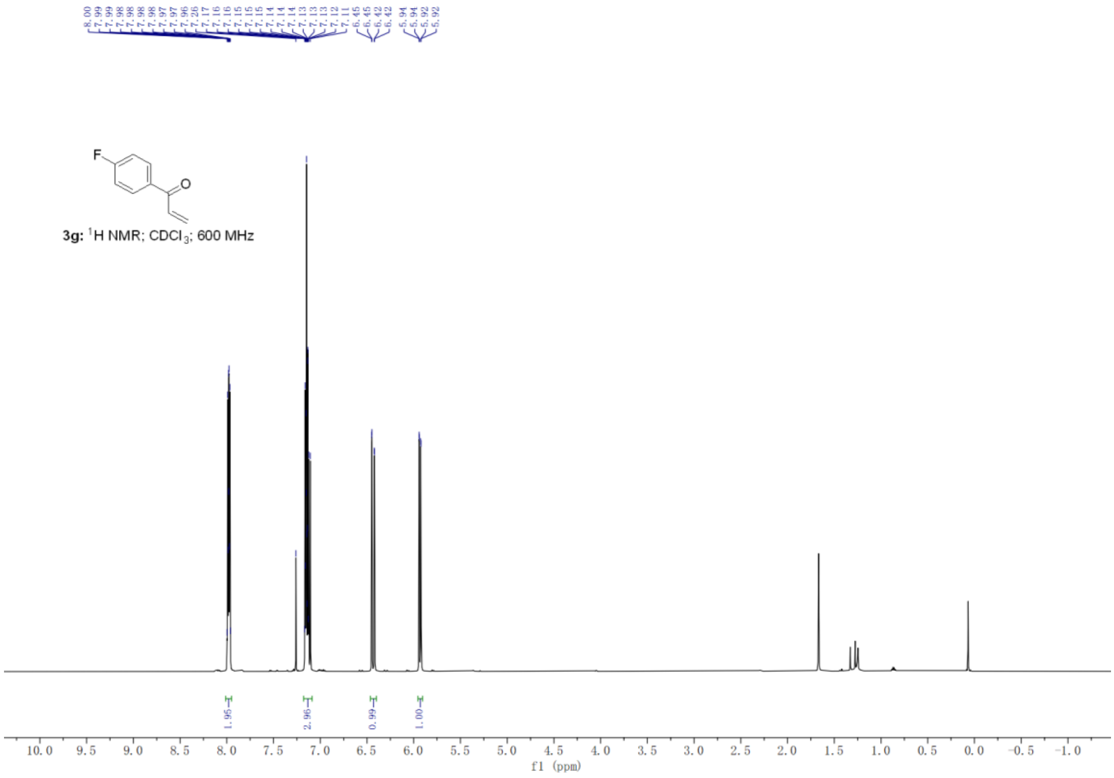
3g: 1H NMR; CDCl3; 600 MHz

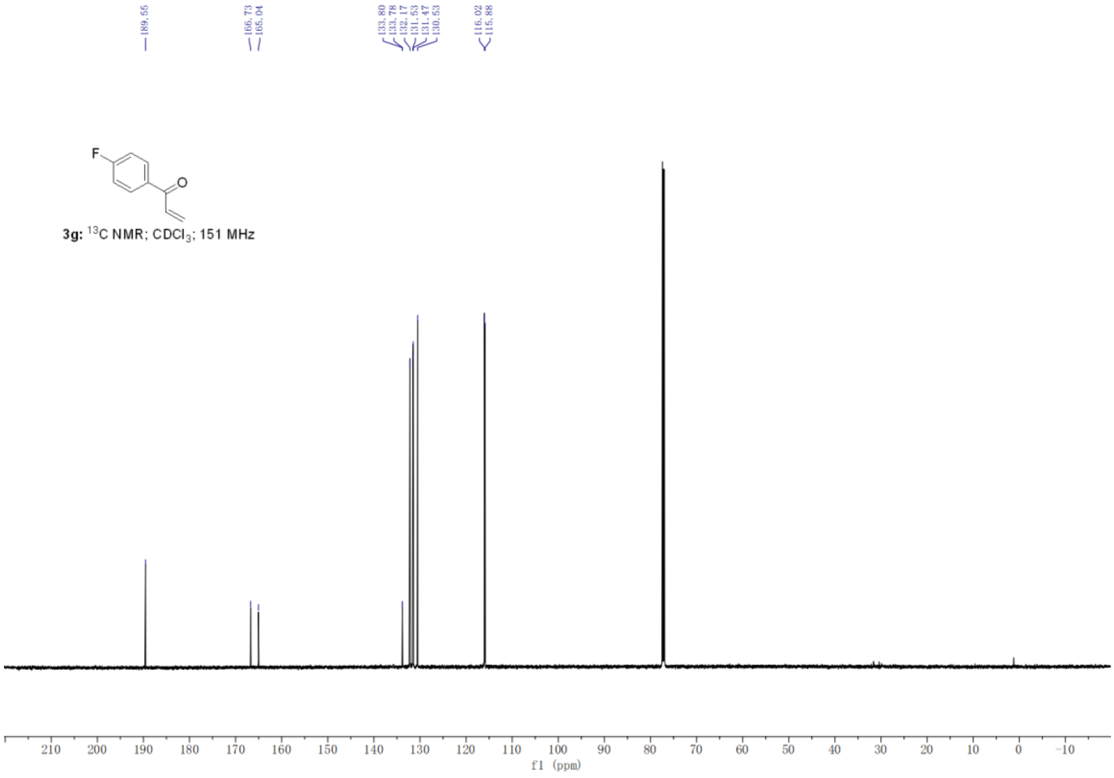
3g: 13C NMR; CDCl3; 151 MHz

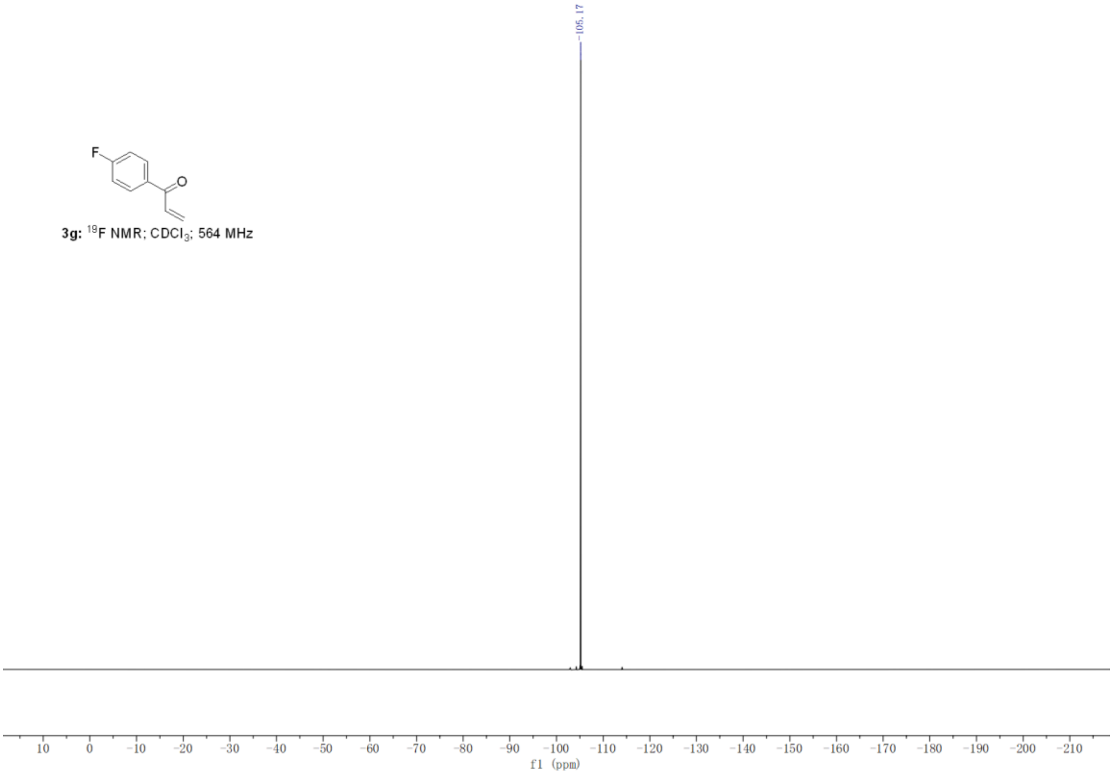

3g: ^{19}F NMR; $CDCl_3$; 564 MHz

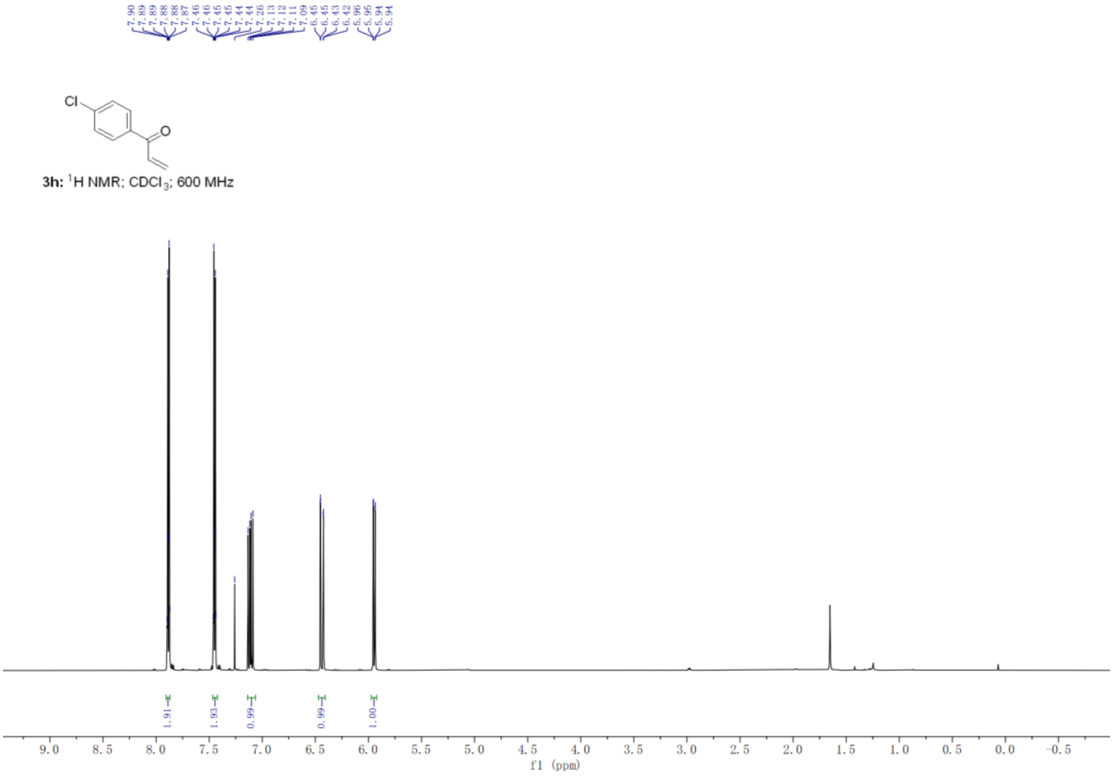

3h: ^{1}H NMR; $CDCl_3$; 600 MHz

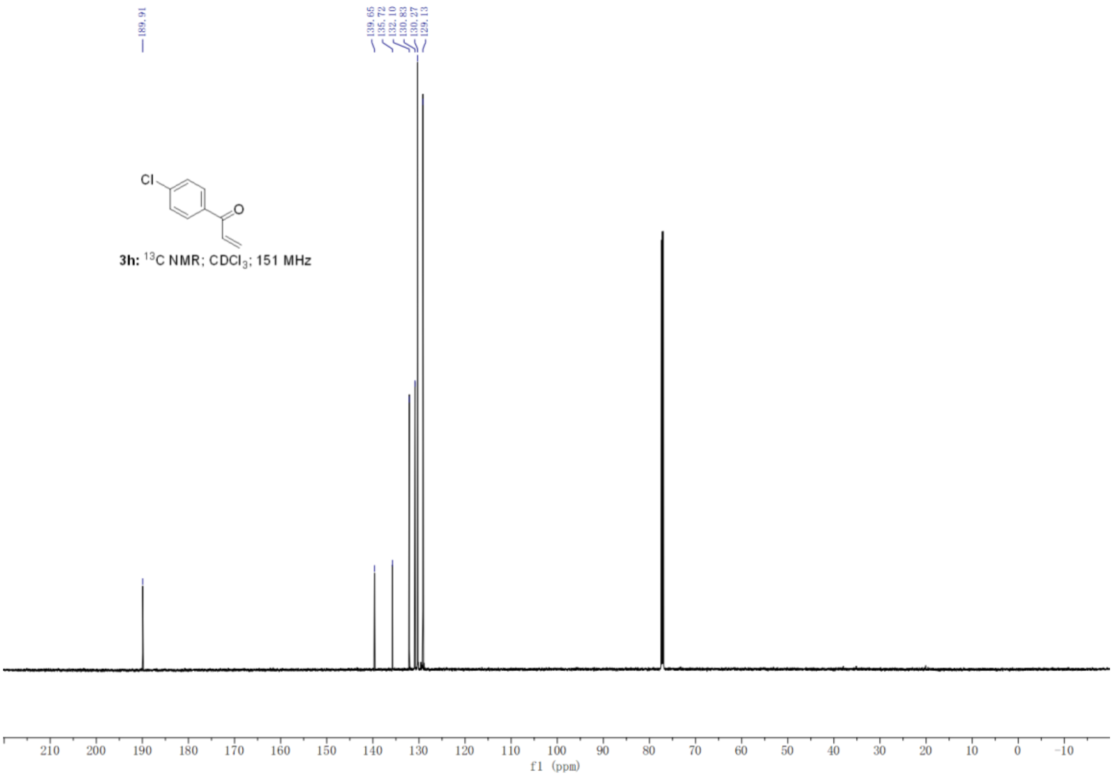
189.91
139.65
135.72
132.10
130.83
130.27
129.13
Cl
O
3h: ^{13}C NMR; $CDCl_3$; 151 MHz
210 200 190 180 170 160 150 140 130 120 110 100 90 80 70 60 50 40 30 20 10 0 -10
f1 (ppm)

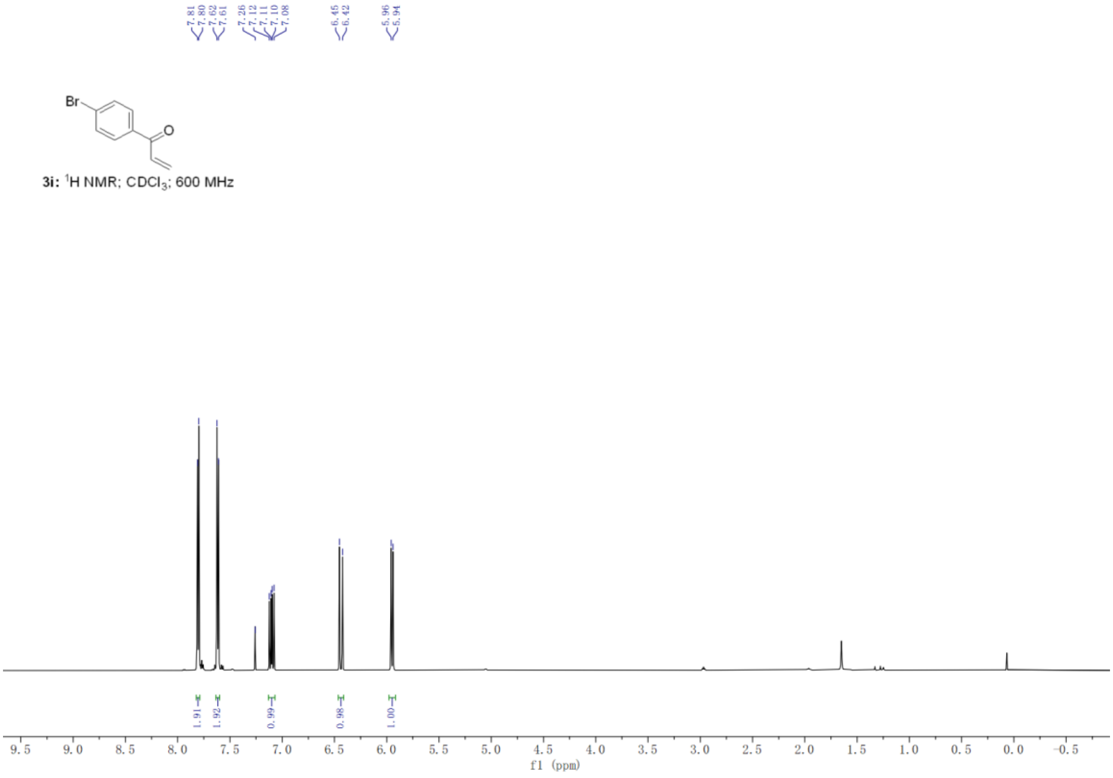
7.81
7.80
7.62
7.61
7.26
7.12
7.11
7.10
7.08
6.45
6.42
5.96
5.94
Br
O
3i: ^{1}H NMR; $CDCl_3$; 600 MHz
1.91
1.92
0.99
0.98
1.00
9.5 9.0 8.5 8.0 7.5 7.0 6.5 6.0 5.5 5.0 4.5 4.0 3.5 3.0 2.5 2.0 1.5 1.0 0.5 0.0 -0.5
f1 (ppm)

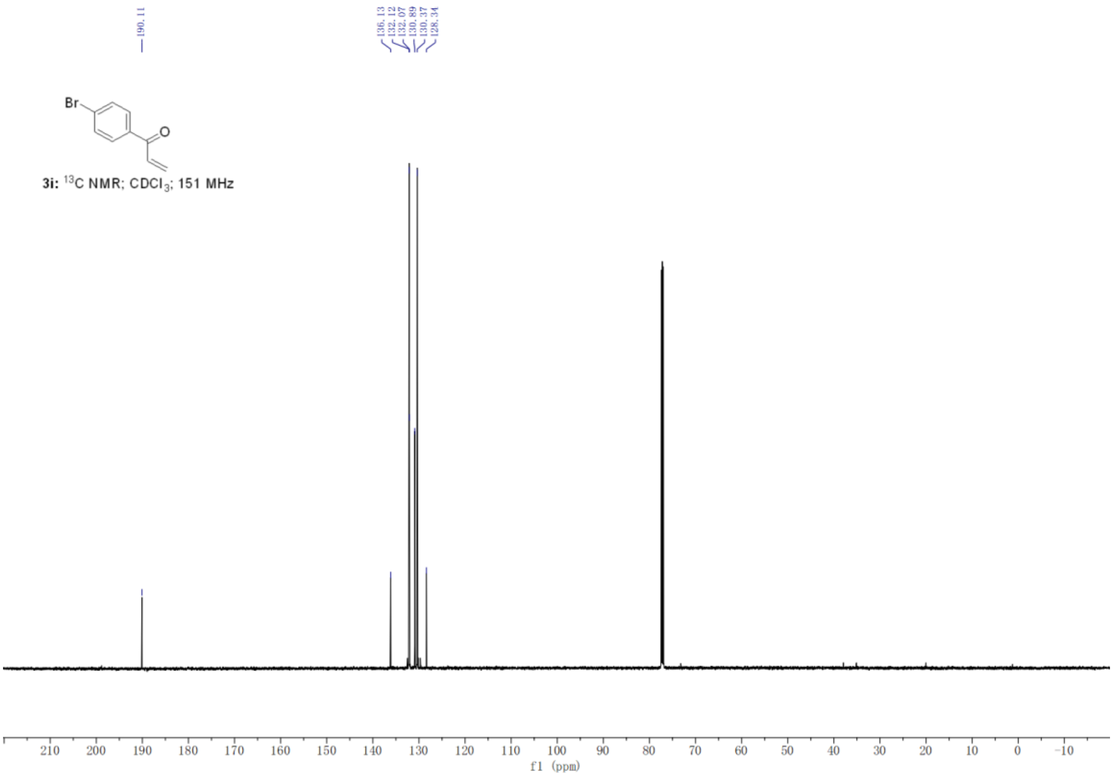
190.11
136.13
132.12
132.07
130.89
130.37
128.34
Br
O
3i: 13C NMR; CDCl3; 151 MHz
210 200 190 180 170 160 150 140 130 120 110 100 90 80 70 60 50 40 30 20 10 0 -10
f1 (ppm)

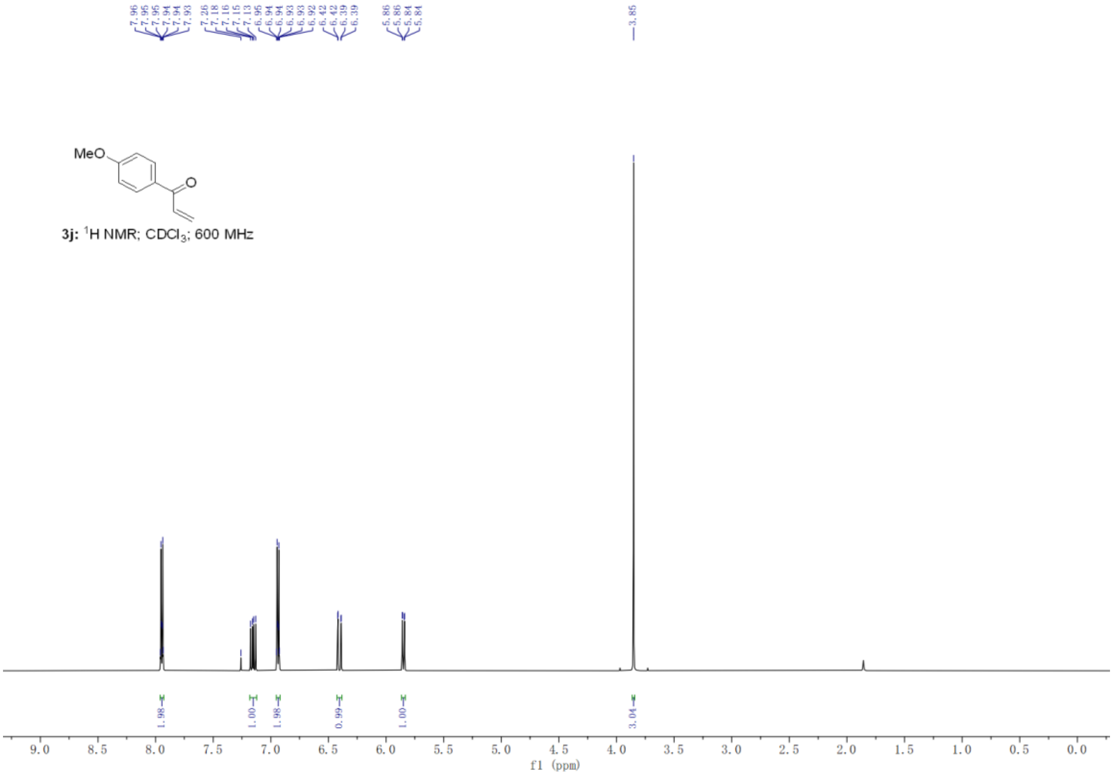
7.96
7.95
7.95
7.94
7.94
7.93
7.26
7.18
7.16
7.15
7.13
6.95
6.94
6.94
6.93
6.93
6.92
6.42
6.42
6.39
6.39
5.86
5.86
5.84
5.84
3.85
MeO
O
3j: 1H NMR; CDCl3; 600 MHz
1.98
1.00
1.98
0.99
1.00
3.04
9.0 8.5 8.0 7.5 7.0 6.5 6.0 5.5 5.0 4.5 4.0 3.5 3.0 2.5 2.0 1.5 1.0 0.5 0.0
f1 (ppm)

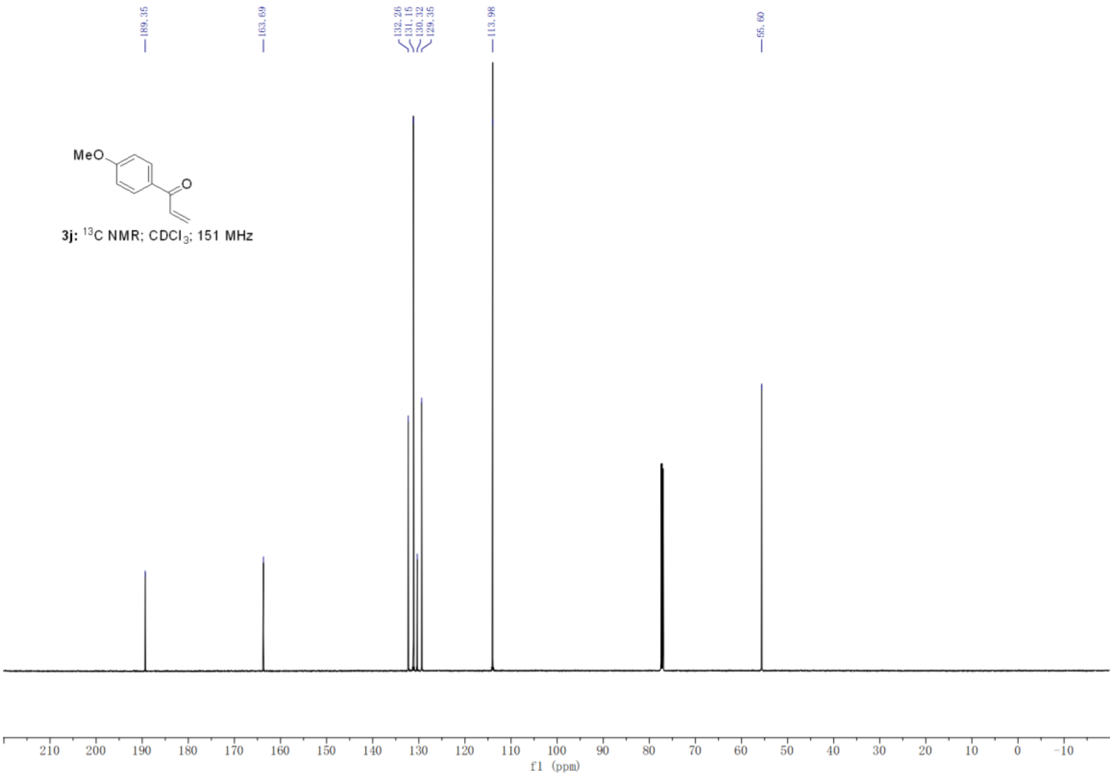
189.35
163.69
132.26
131.15
130.32
129.35
113.98
55.60
MeO
O
3j: 13C NMR; CDCl3; 151 MHz
210 200 190 180 170 160 150 140 130 120 110 100 90 80 70 60 50 40 30 20 10 0 −10
f1 (ppm)

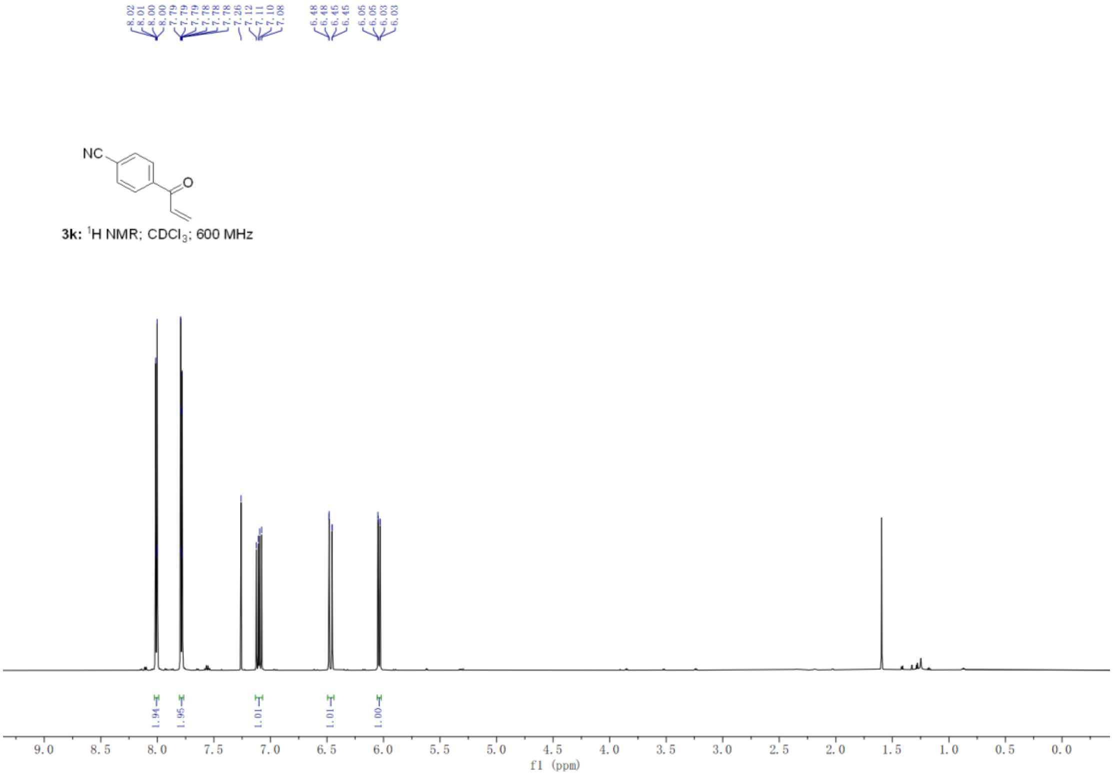
8.02
8.01
8.00
8.00
7.79
7.79
7.79
7.78
7.78
7.78
7.26
7.12
7.11
7.10
7.08
6.48
6.48
6.45
6.45
6.05
6.05
6.03
6.03
NC
O
3k: 1H NMR; CDCl3; 600 MHz
1.94
1.95
1.01
1.01
1.00
9.0 8.5 8.0 7.5 7.0 6.5 6.0 5.5 5.0 4.5 4.0 3.5 3.0 2.5 2.0 1.5 1.0 0.5 0.0
f1 (ppm)

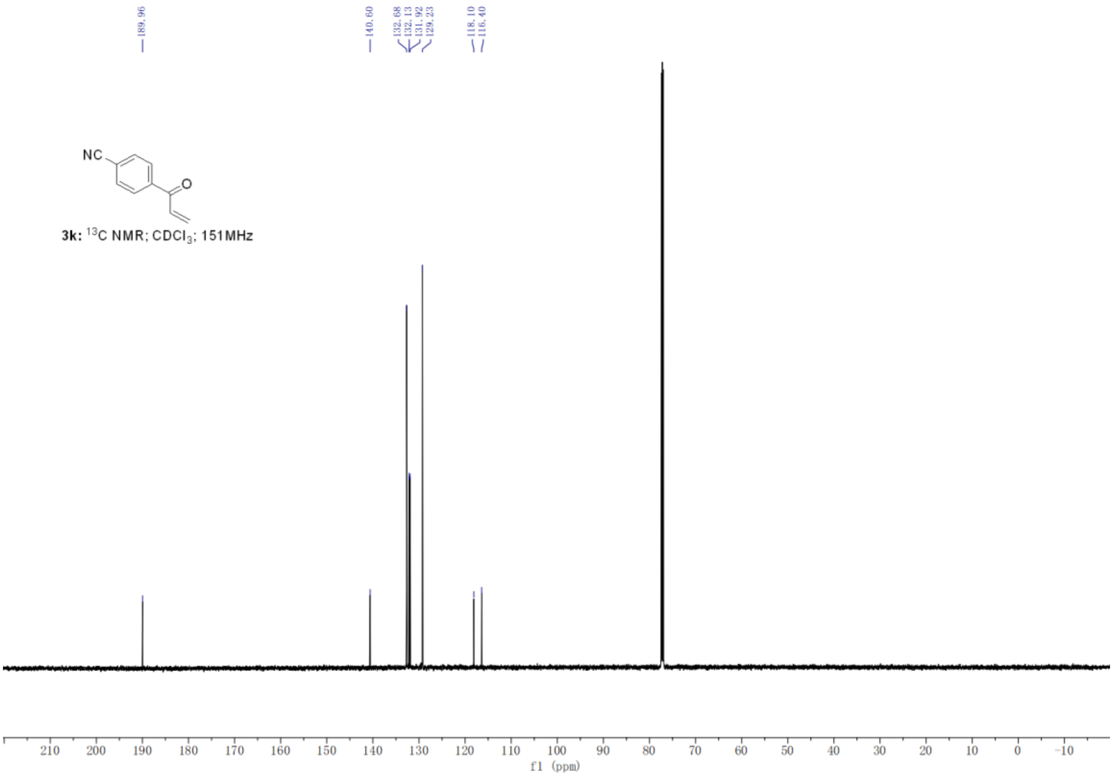
189.96
140.60
132.68
132.13
131.92
129.23
118.10
116.40
NC
O
3k: ^{13}C NMR; $CDCl_3$; 151MHz
210 200 190 180 170 160 150 140 130 120 110 100 90 80 70 60 50 40 30 20 10 0 -10
f1 (ppm)

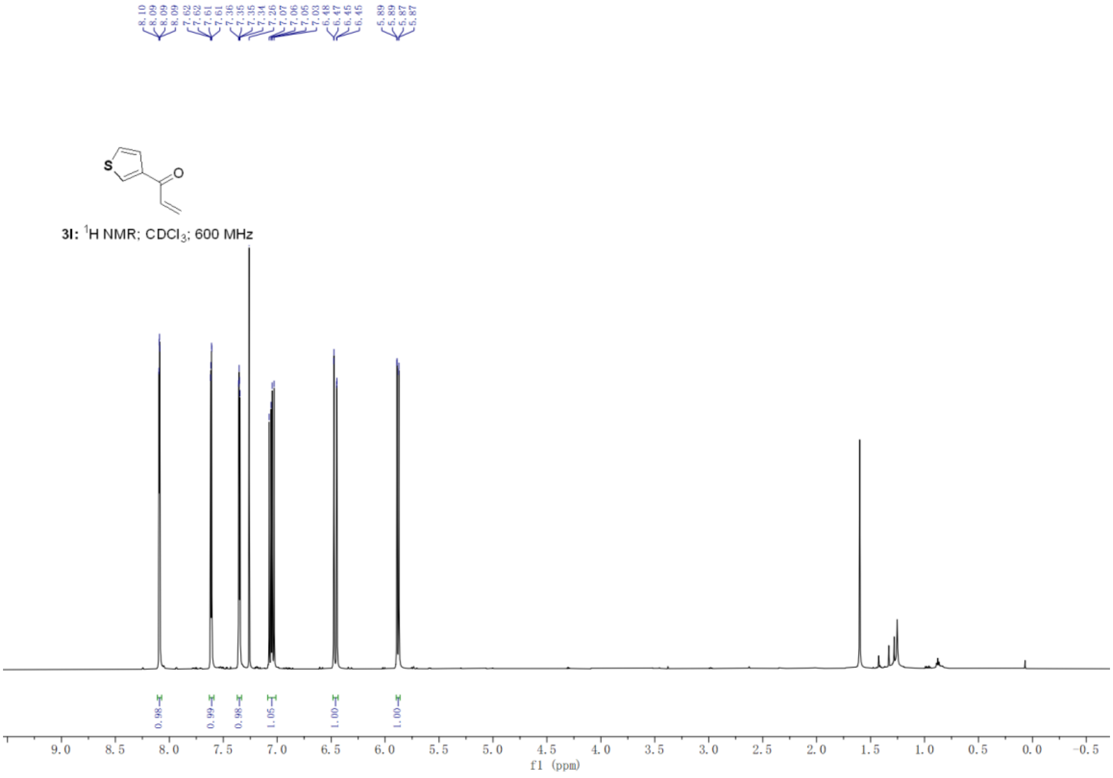
8.10
8.09
8.09
8.09
7.62
7.62
7.61
7.61
7.36
7.35
7.35
7.34
7.26
7.07
7.06
7.05
7.03
6.48
6.47
6.45
6.45
5.89
5.89
5.87
5.87
S
O
3l: ^{1}H NMR; $CDCl_3$; 600 MHz
0.98
0.99
0.98
1.05
1.00
1.00
9.0 8.5 8.0 7.5 7.0 6.5 6.0 5.5 5.0 4.5 4.0 3.5 3.0 2.5 2.0 1.5 1.0 0.5 0.0 -0.5
f1 (ppm)

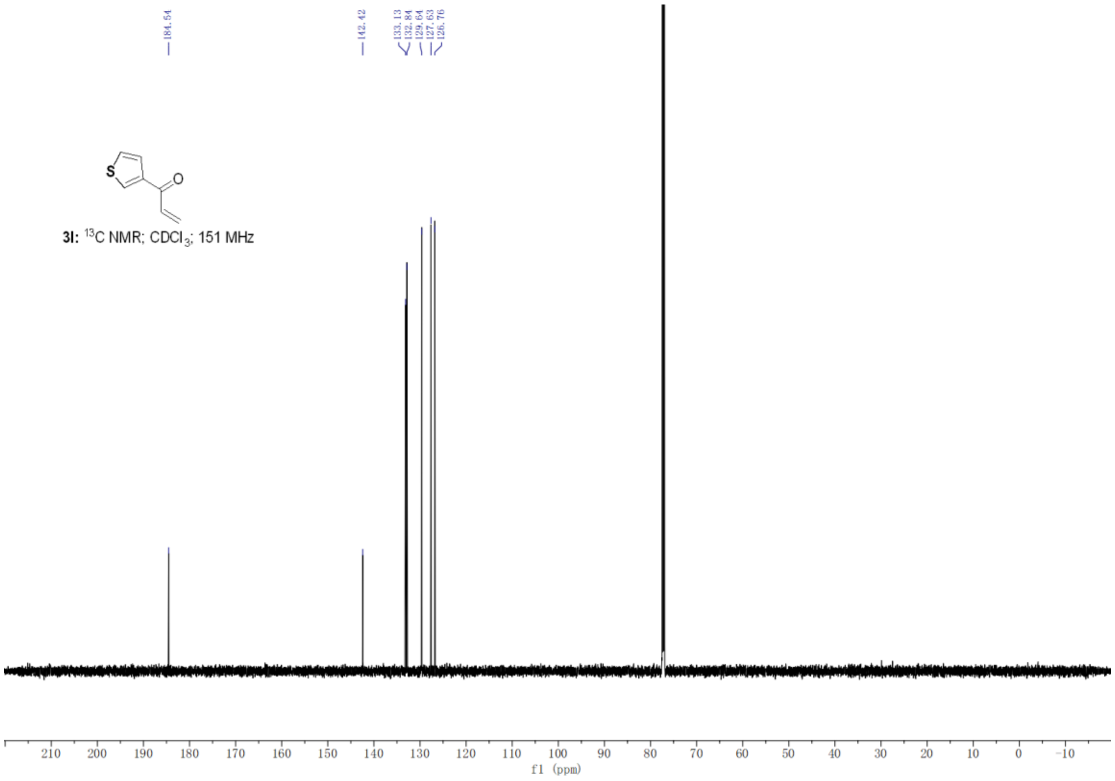
184.54
142.42
133.13
132.84
129.64
127.63
126.76
3l: ¹³C NMR; CDCl₃; 151 MHz
210 200 190 180 170 160 150 140 130 120 110 100 90 80 70 60 50 40 30 20 10 0 -10
f1 (ppm)

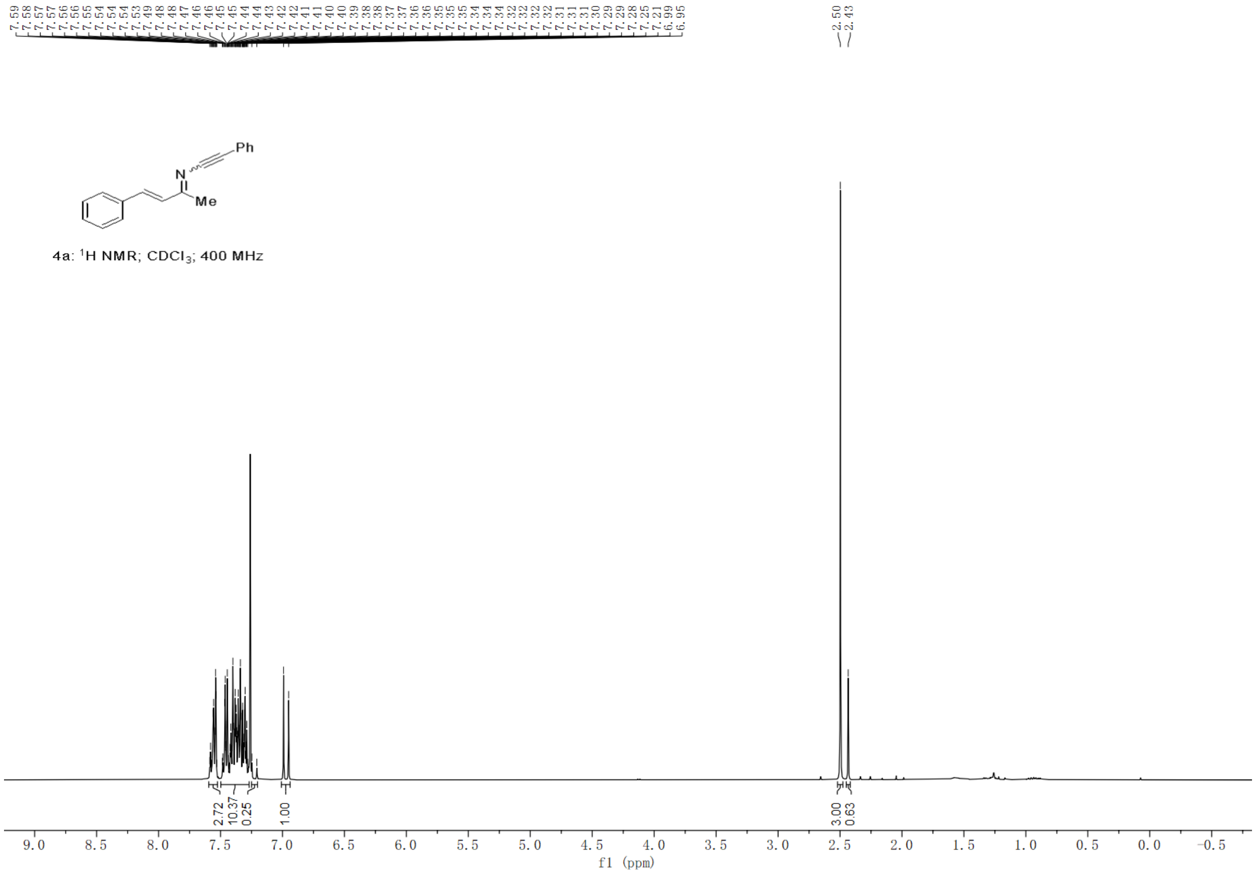
7.59 7.58 7.57 7.57 7.56 7.56 7.55 7.54 7.54 7.53 7.49 7.48 7.48 7.47 7.46 7.46 7.45 7.45 7.44 7.44 7.43 7.42 7.42 7.41 7.40 7.40 7.39 7.38 7.38 7.37 7.36 7.36 7.35 7.35 7.34 7.34 7.34 7.32 7.32 7.32 7.32 7.31 7.31 7.31 7.30 7.29 7.29 7.28 7.25 7.21 6.99 6.95
2.50
2.43
Ph
N
Me
4a: ¹H NMR; CDCl₃; 400 MHz
2.72
10.37
0.25
1.00
3.00
0.63
9.0 8.5 8.0 7.5 7.0 6.5 6.0 5.5 5.0 4.5 4.0 3.5 3.0 2.5 2.0 1.5 1.0 0.5 0.0 -0.5
f1 (ppm)

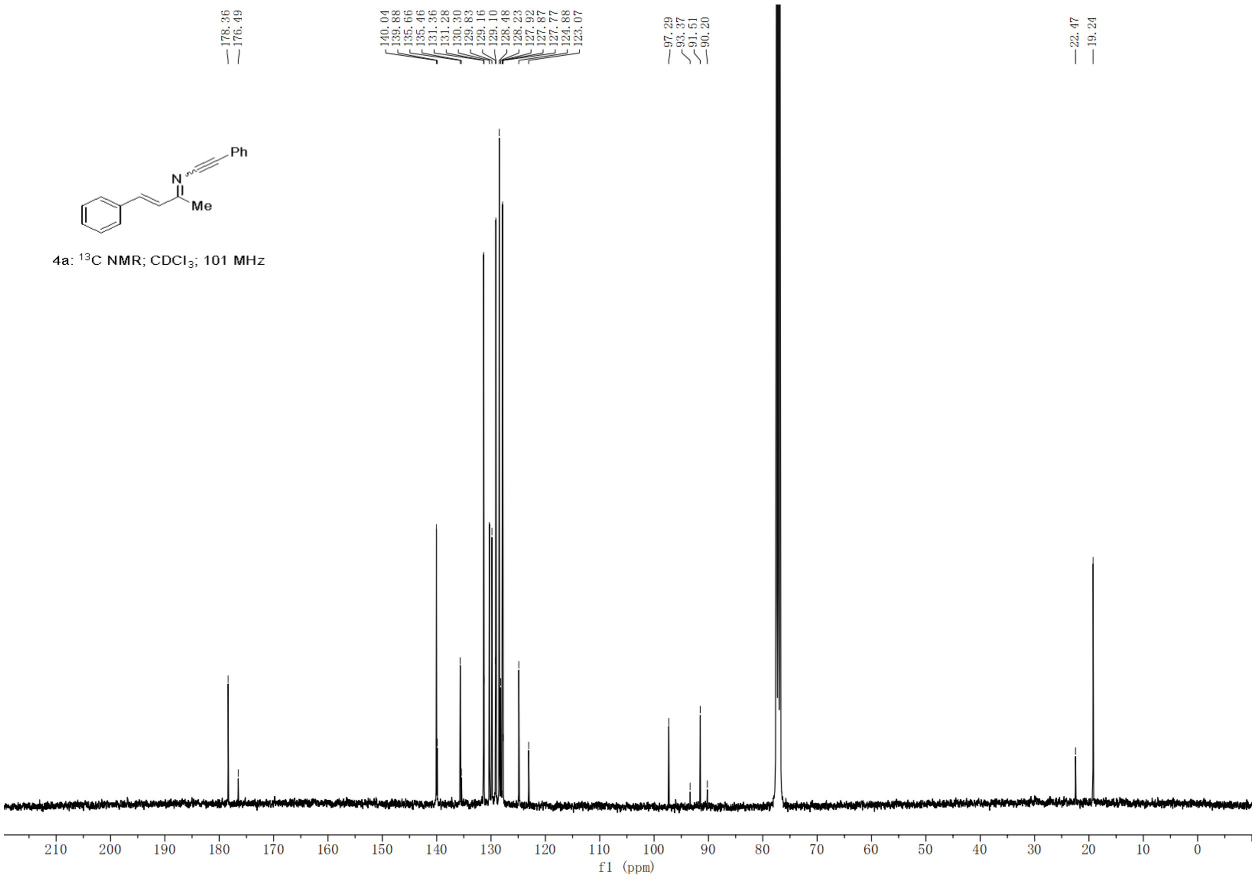

4a: ^{13}C NMR; $CDCl_3$; 101 MHz

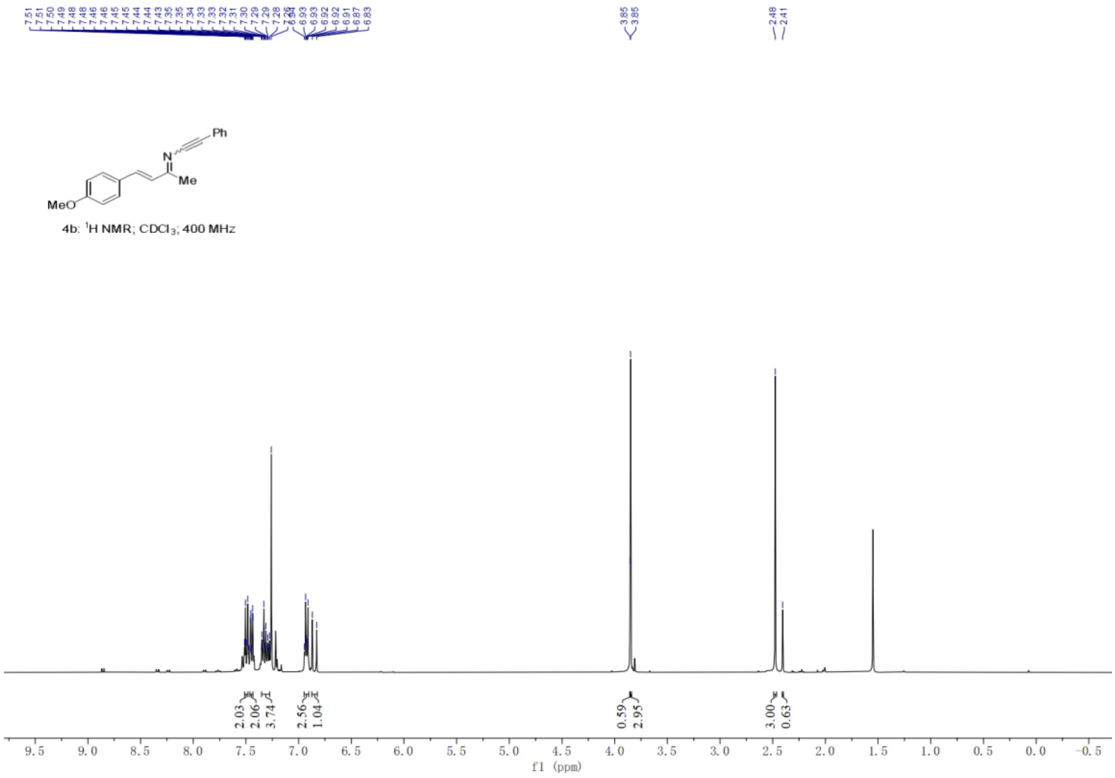

4b: ^{1}H NMR; $CDCl_3$; 400 MHz

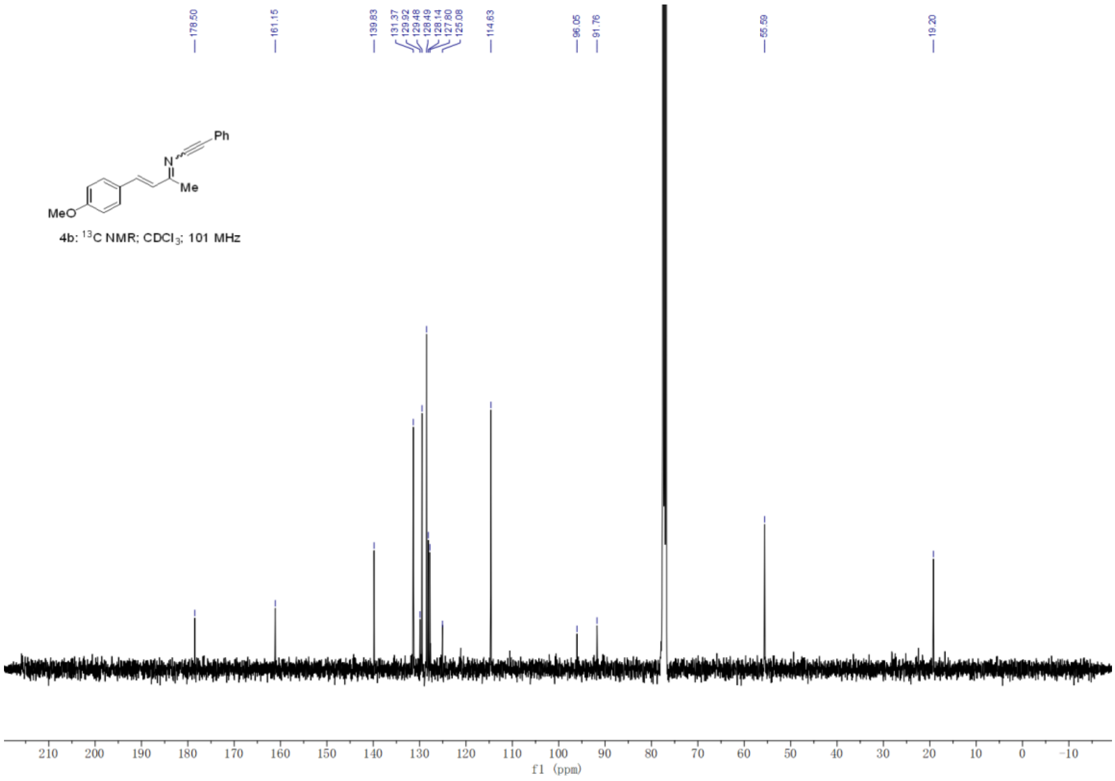
178.50
161.15
139.83
131.37
129.92
129.48
128.49
128.14
127.80
125.08
114.63
96.05
91.76
55.59
19.20
Ph
N
Me
MeO
4b: 13C NMR; CDCl3; 101 MHz
210 200 190 180 170 160 150 140 130 120 110 100 90 80 70 60 50 40 30 20 10 0 -10
f1 (ppm)

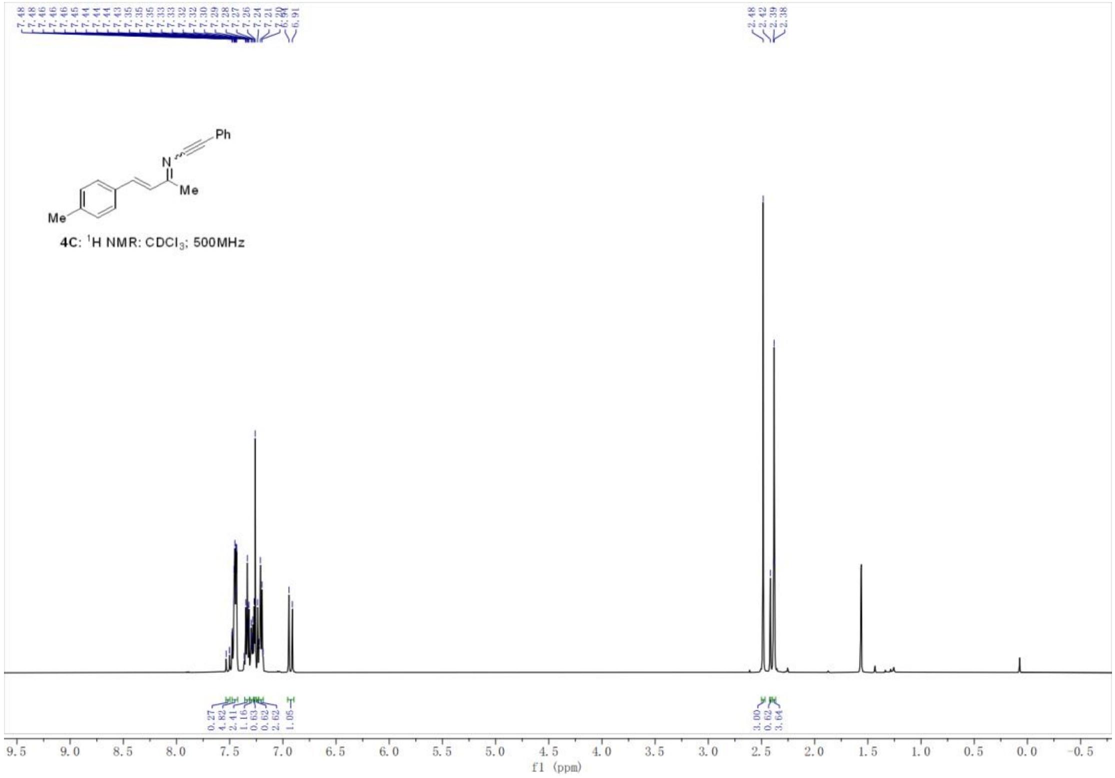
7.48
7.46
7.46
7.45
7.44
7.44
7.43
7.35
7.35
7.33
7.32
7.32
7.30
7.29
7.28
7.27
7.26
7.24
7.21
6.91
2.48
2.42
2.39
2.38
Ph
N
Me
Me
4C: 1H NMR: CDCl3; 500MHz
0.27
4.82
2.41
1.16
0.63
0.62
2.62
1.05
3.00
0.62
3.64
9.5 9.0 8.5 8.0 7.5 7.0 6.5 6.0 5.5 5.0 4.5 4.0 3.5 3.0 2.5 2.0 1.5 1.0 0.5 0.0 -0.5
f1 (ppm)

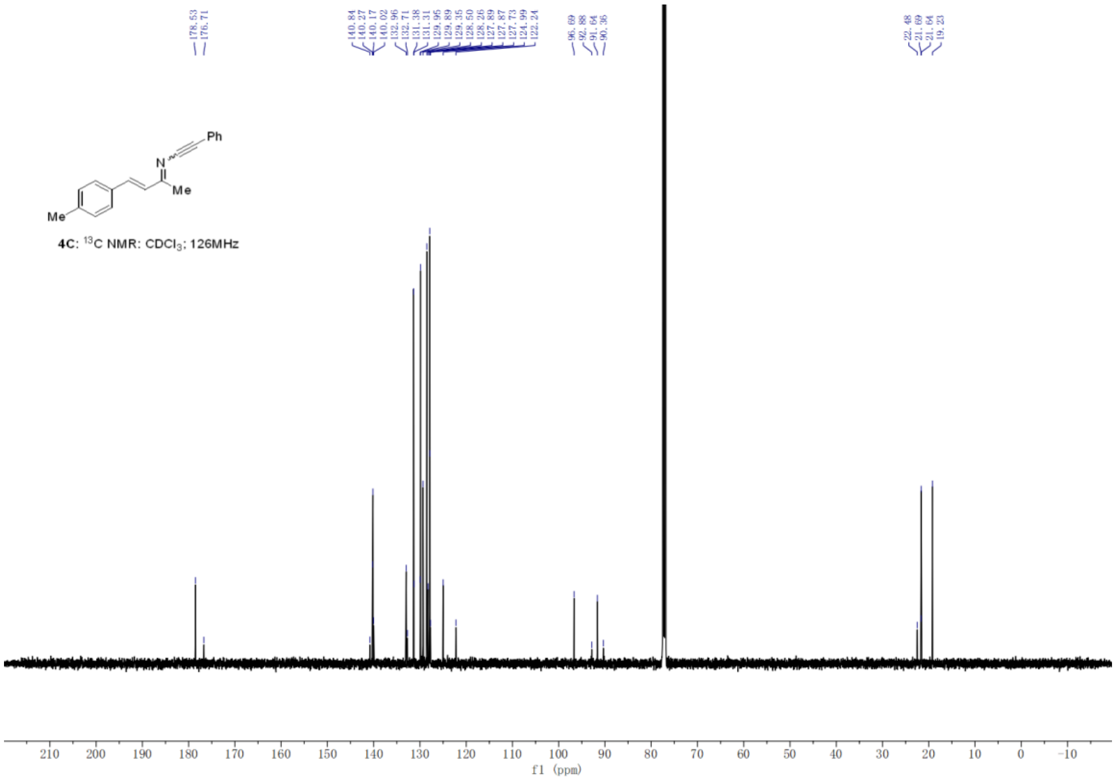

4C: ^{13}C NMR: $CDCl_3$; 126MHz

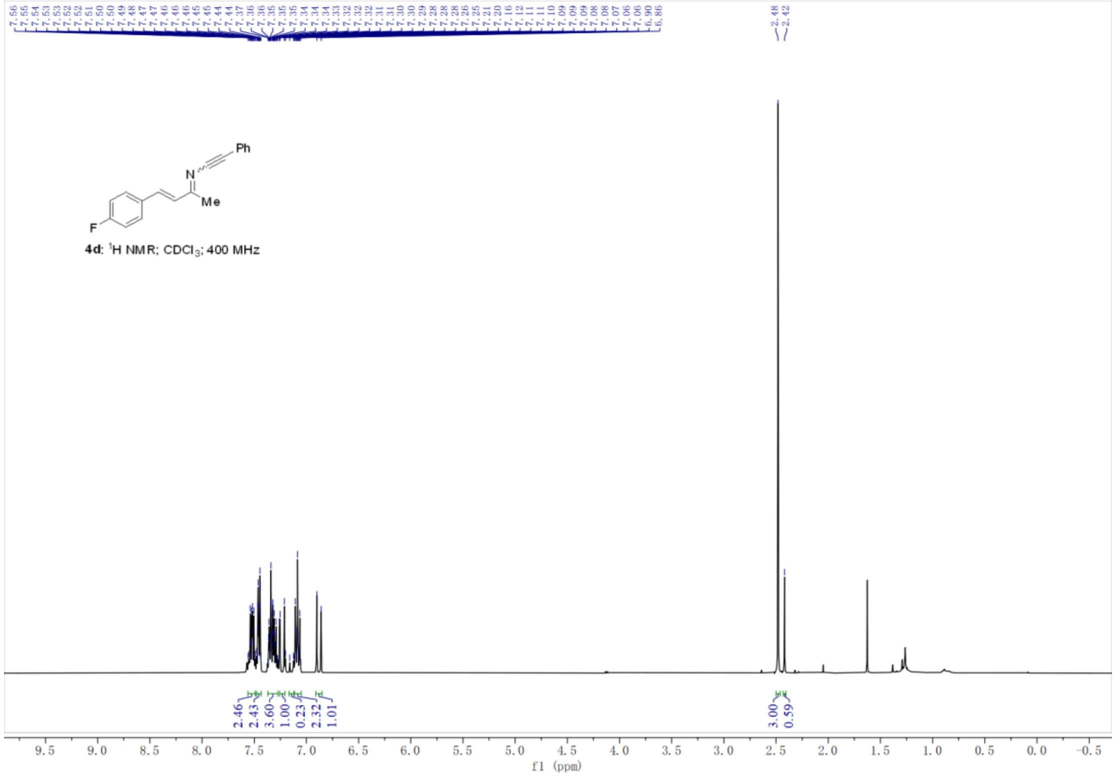

4d: ^{1}H NMR; $CDCl_3$; 400 MHz

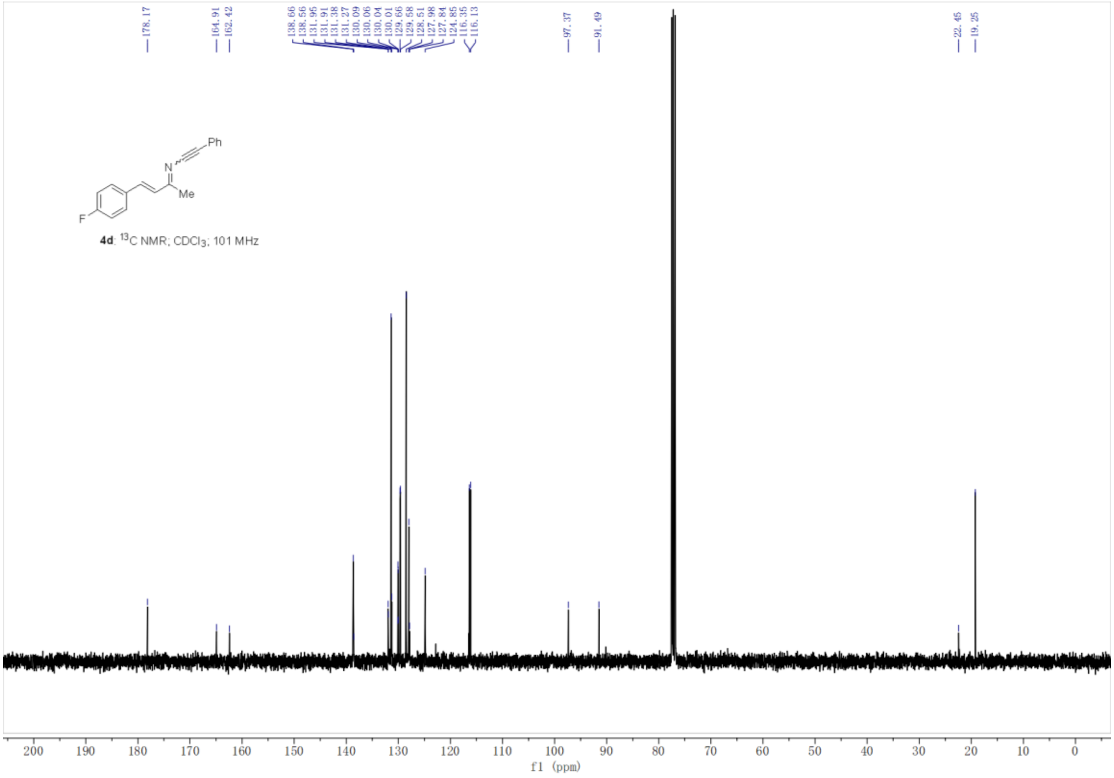
178.17
164.91
162.42
138.66
138.56
131.95
131.91
131.38
131.27
130.09
130.06
130.04
130.01
129.66
129.58
128.51
127.98
127.84
124.85
116.35
116.13
97.37
91.49
22.45
19.25
Ph
N
Me
F
4d: 13C NMR; CDCl3; 101 MHz
f1 (ppm)

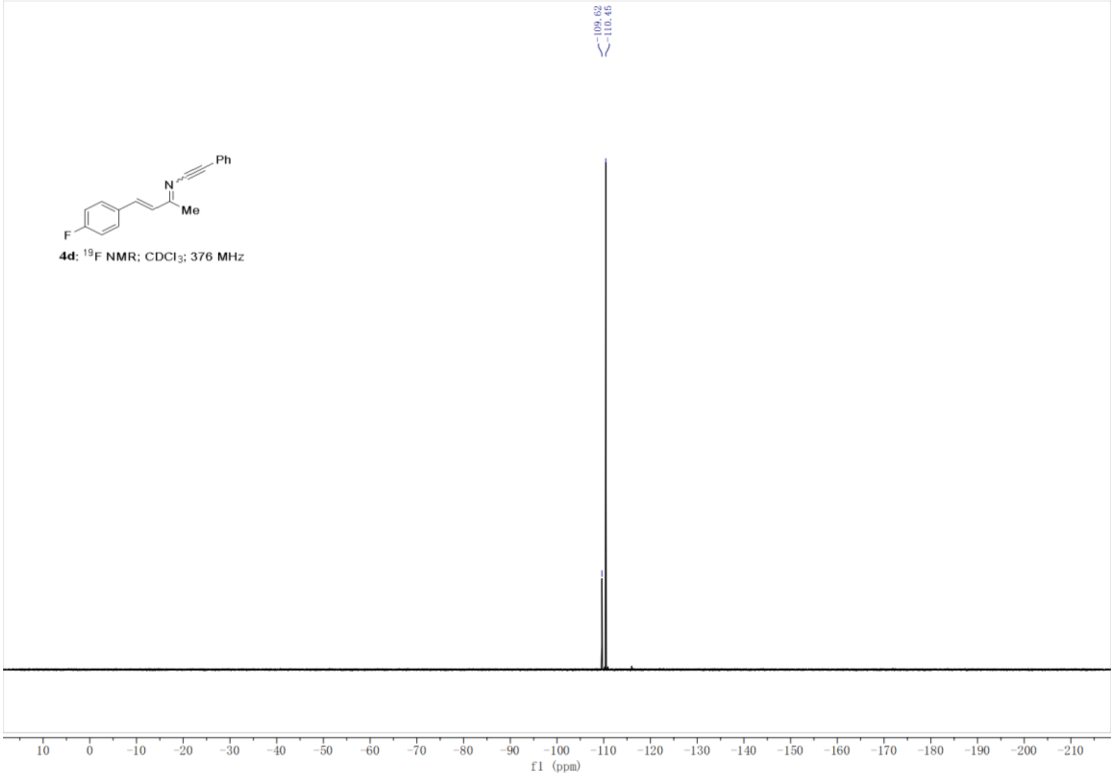
-109.62
-110.45
Ph
N
Me
F
4d: 19F NMR; CDCl3; 376 MHz
f1 (ppm)

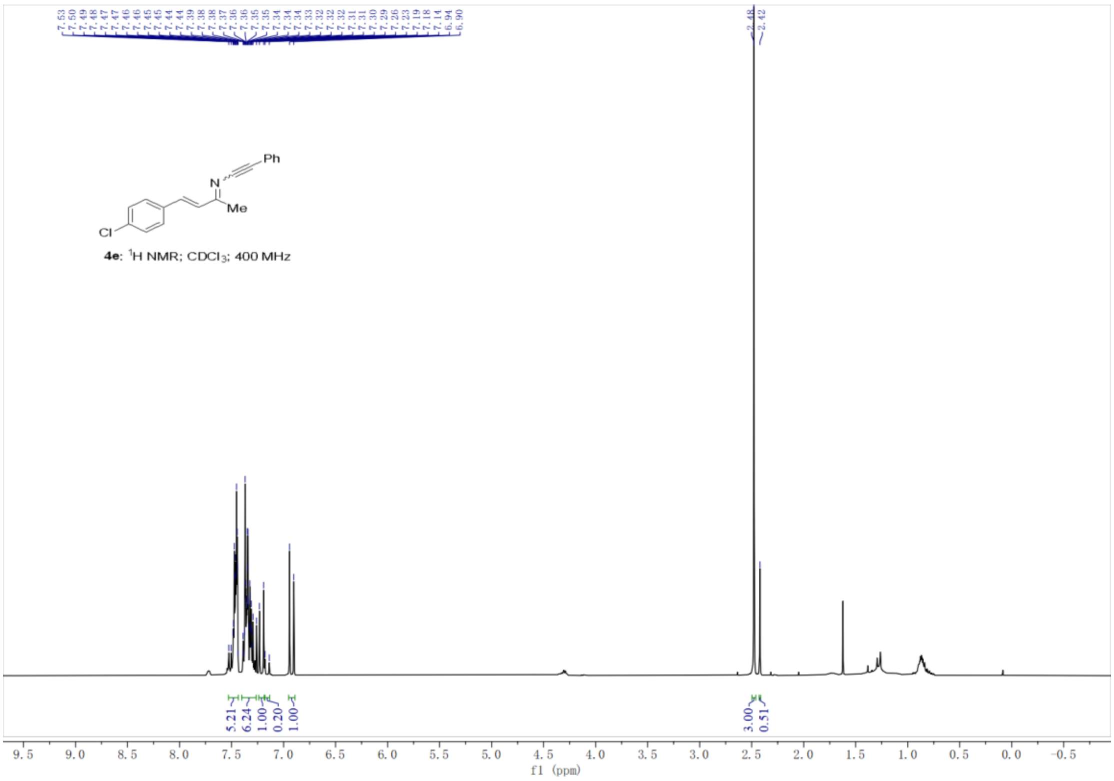
4e: 1H NMR; CDCl3; 400 MHz
f1 (ppm)

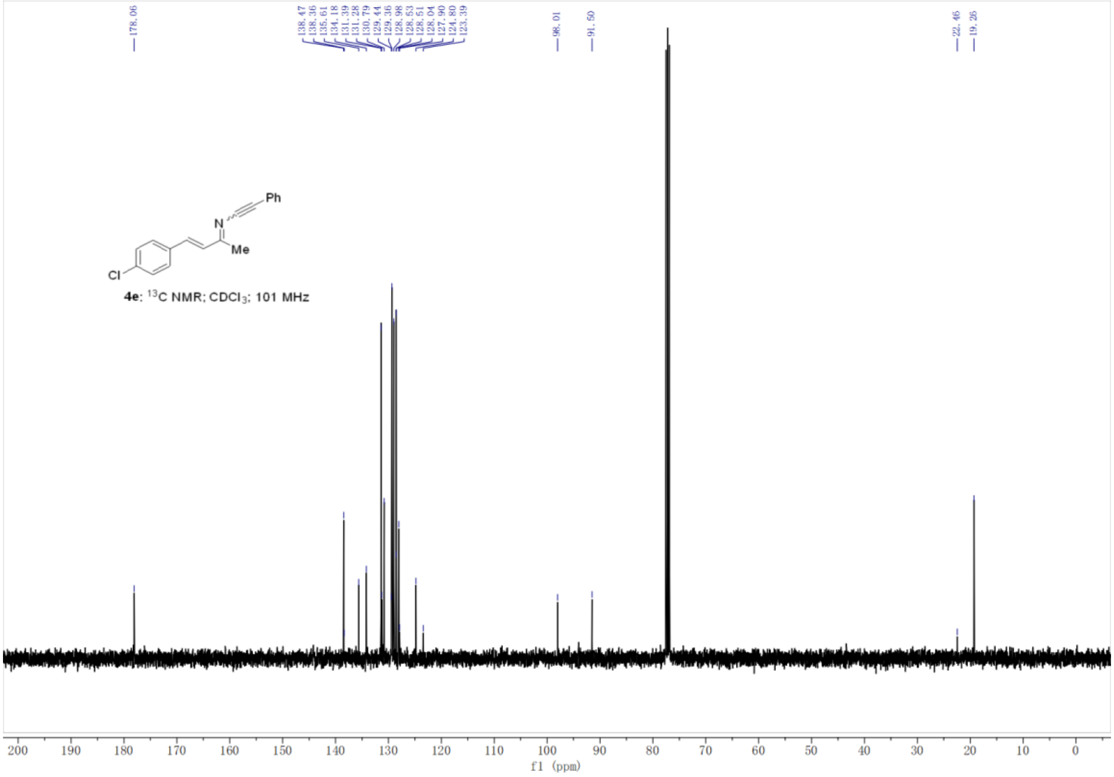
4e: 13C NMR; CDCl3; 101 MHz
f1 (ppm)

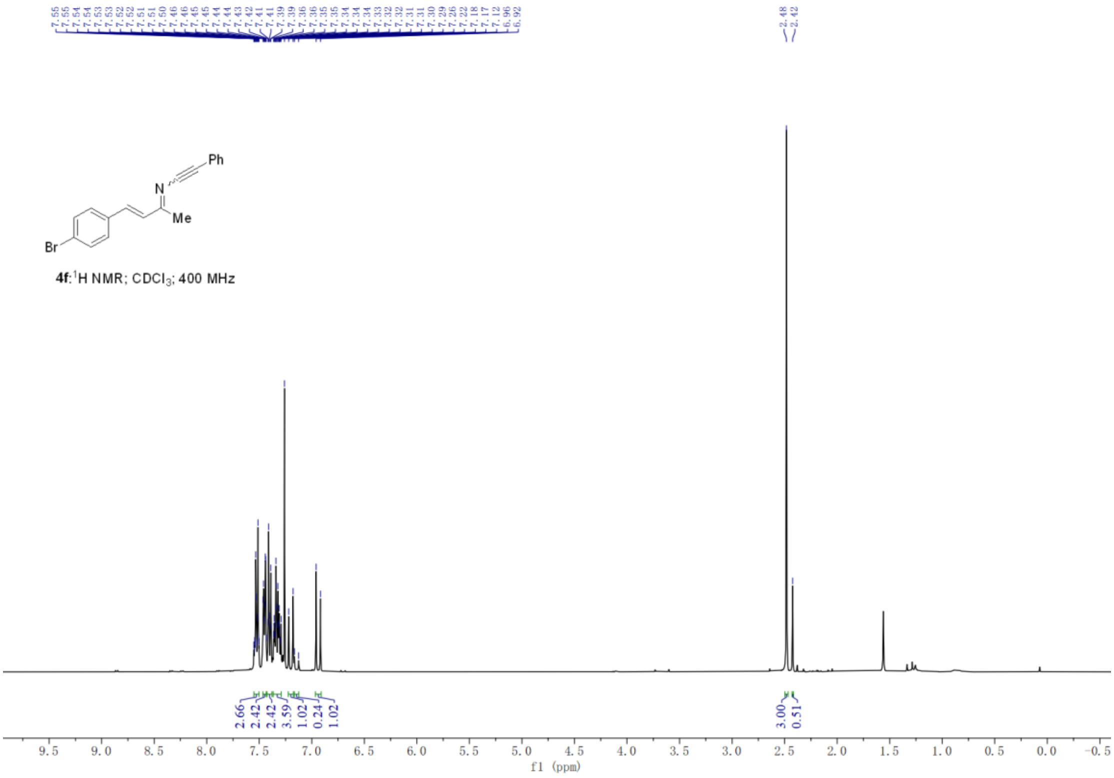
7.55 7.55 7.54 7.53 7.53 7.52 7.52 7.51 7.51 7.50 7.46 7.45 7.45 7.44 7.44 7.43 7.42 7.41 7.41 7.39 7.39 7.36 7.36 7.35 7.35 7.34 7.34 7.34 7.33 7.32 7.32 7.31 7.31 7.30 7.29 7.26 7.22 7.18 7.17 7.12 6.96 6.92
2.48 2.42
Ph
N
Me
Br
4f: 1H NMR; CDCl3; 400 MHz
2.66 2.42 2.42 3.59 1.02 0.24 1.02
3.00 0.51
f1 (ppm)

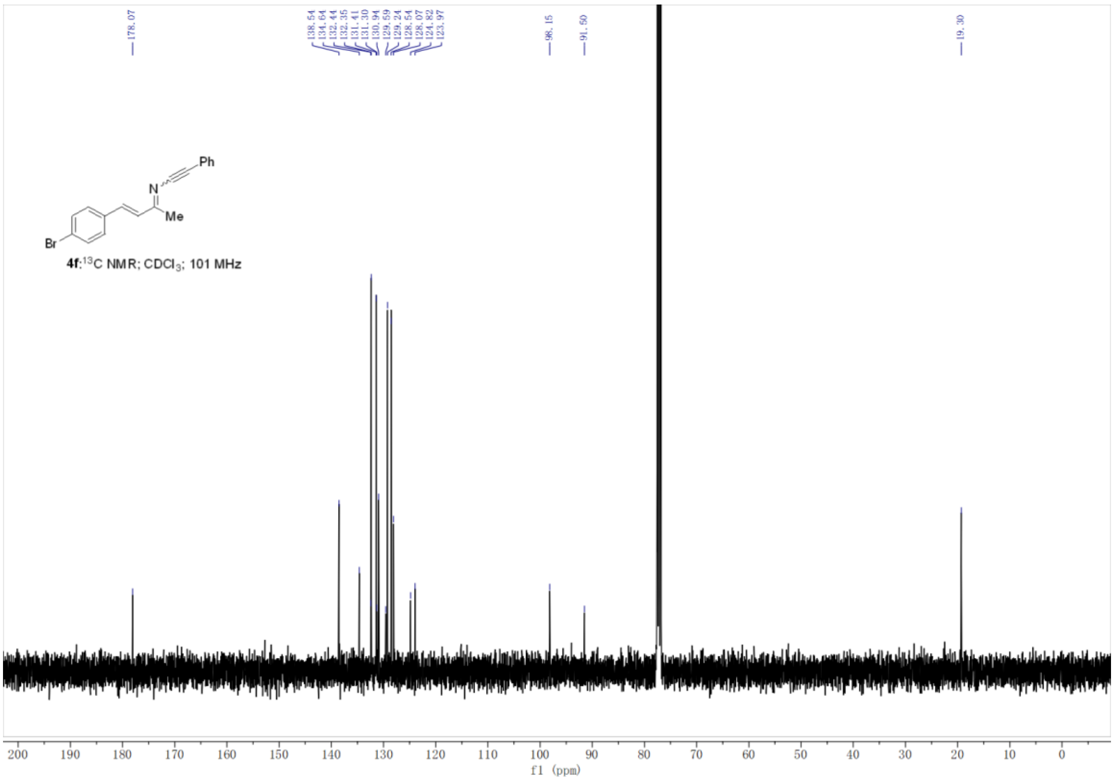
178.07
138.54 134.64 132.44 132.35 131.41 131.30 130.94 129.59 129.24 128.54 128.07 124.82 123.97
98.15
91.50
19.30
Ph
N
Me
Br
4f: 13C NMR; CDCl3; 101 MHz
f1 (ppm)

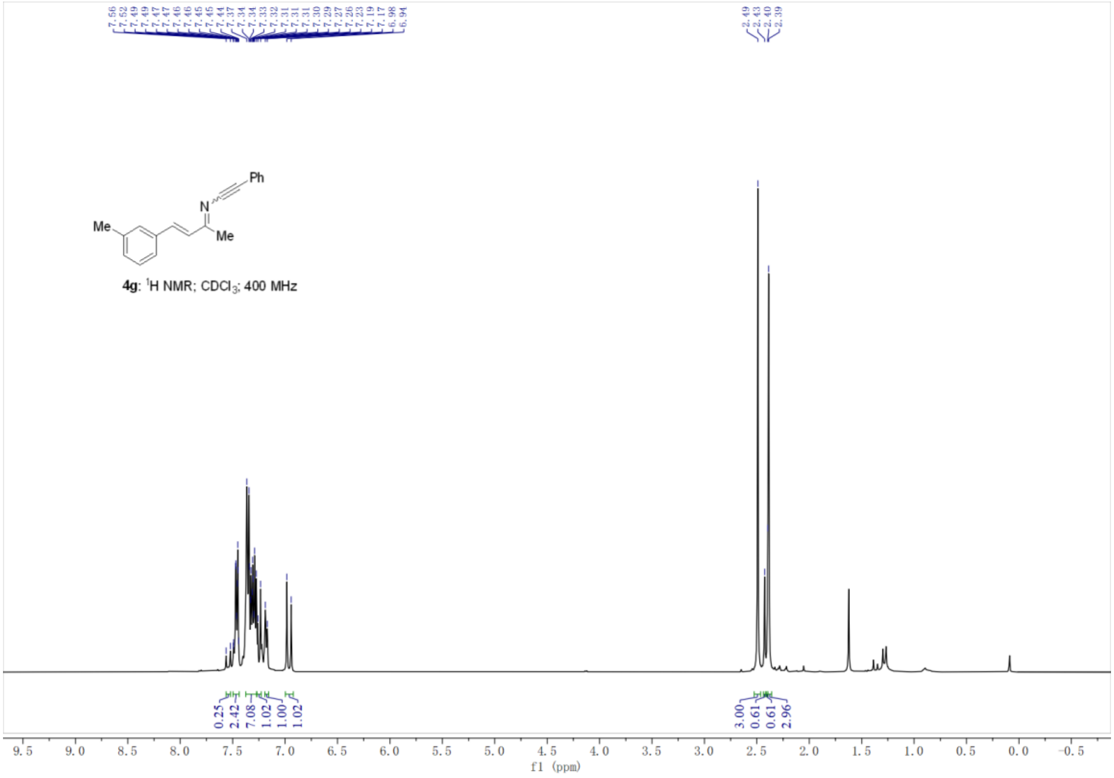

7.56
7.52
7.49
7.47
7.47
7.46
7.45
7.45
7.44
7.37
7.34
7.34
7.33
7.32
7.31
7.31
7.30
7.29
7.27
7.26
7.23
7.19
7.17
6.98
6.94
2.49
2.43
2.40
2.39
Me
N
Ph
Me
4g: 1H NMR; CDCl3; 400 MHz
0.25
2.42
7.08
1.02
1.00
1.02
3.00
0.61
0.61
2.96
f1 (ppm)

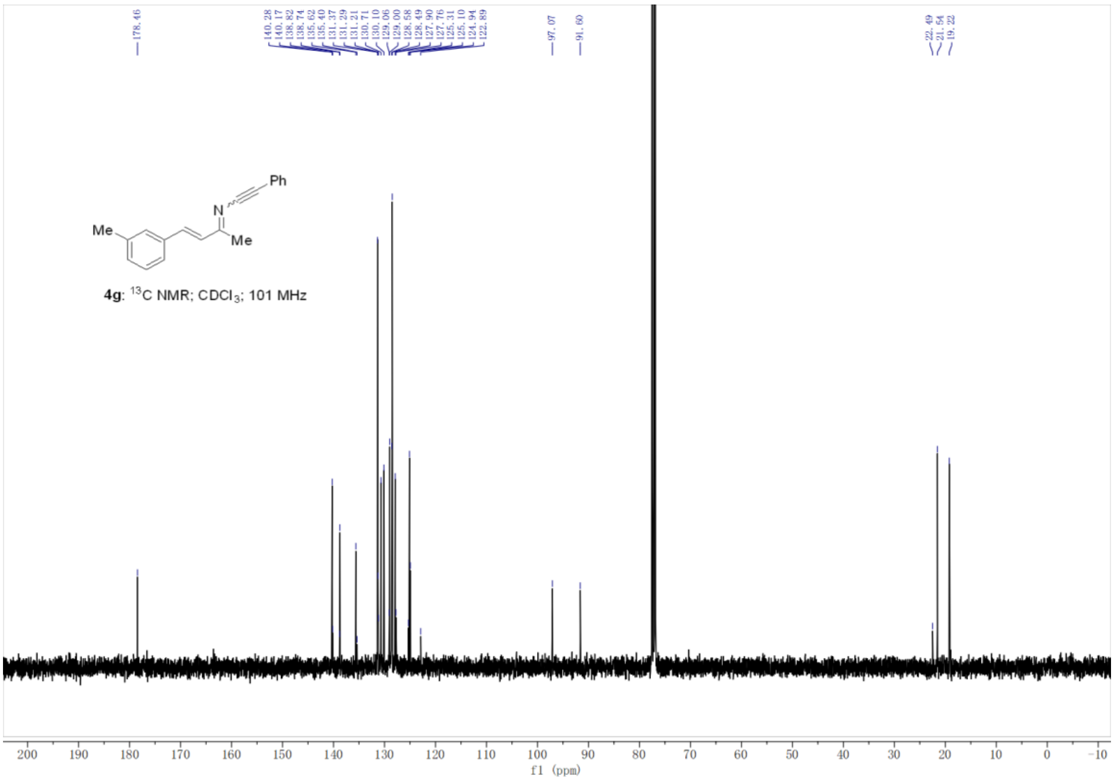

178.46
140.28
140.17
138.82
138.74
135.62
135.40
131.37
131.29
131.21
130.71
130.10
129.06
129.00
128.58
128.49
127.90
127.76
125.31
125.10
124.94
122.89
97.07
91.60
22.49
21.54
19.22
Me
N
Ph
Me
4g: 13C NMR; CDCl3; 101 MHz
f1 (ppm)

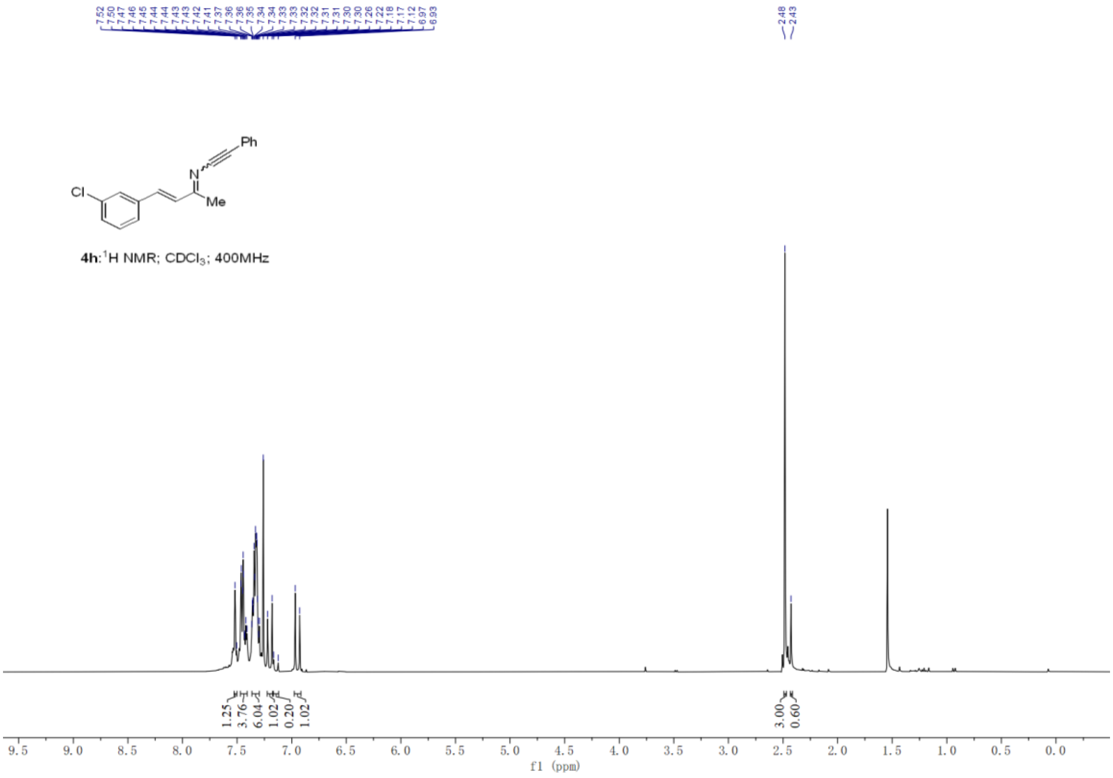
Ph
N
Cl
Me
4h: 1H NMR; CDCl3; 400MHz
f1 (ppm)

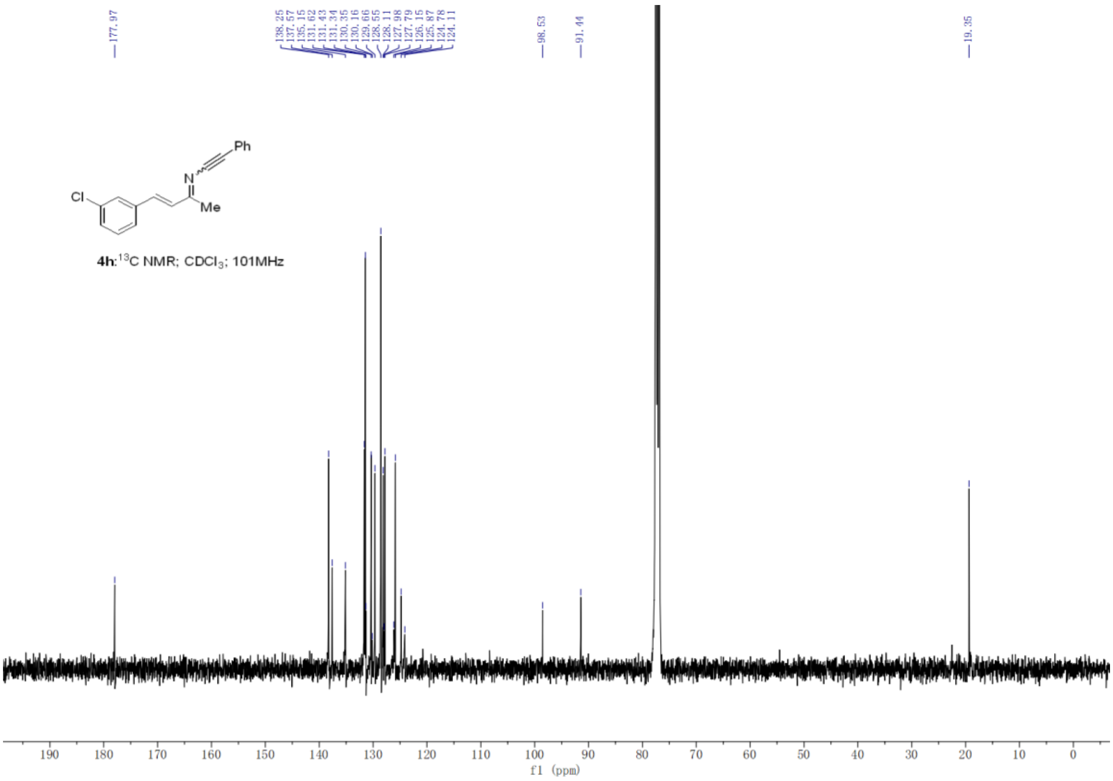
177.97
98.53
91.44
19.35
Ph
N
Cl
Me
4h: 13C NMR; CDCl3; 101MHz
f1 (ppm)

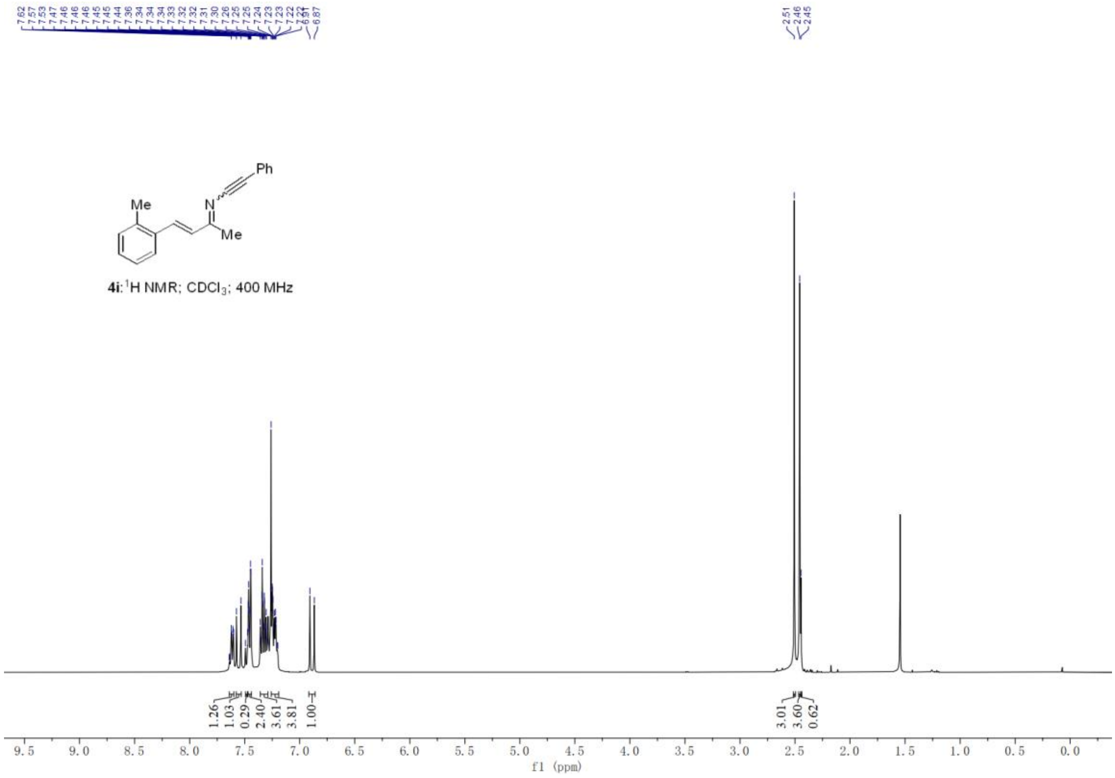
4i: ¹H NMR; CDCl3; 400 MHz
f1 (ppm)

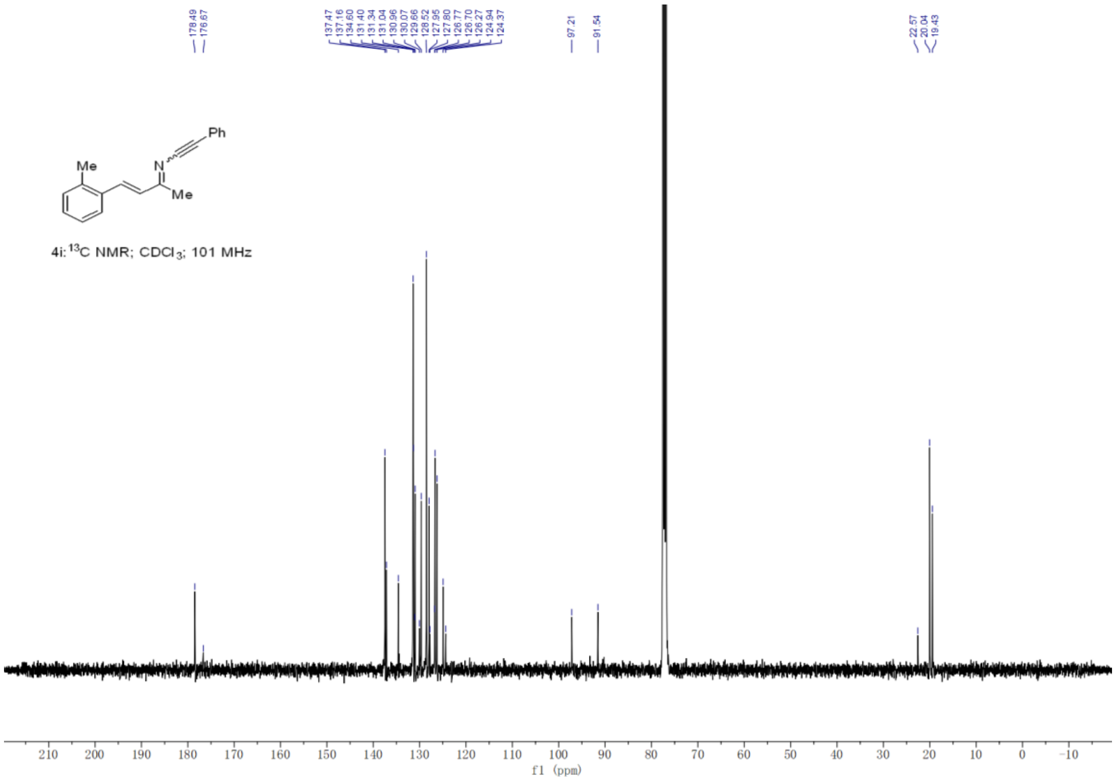
4i: ¹³C NMR; CDCl3; 101 MHz
f1 (ppm)

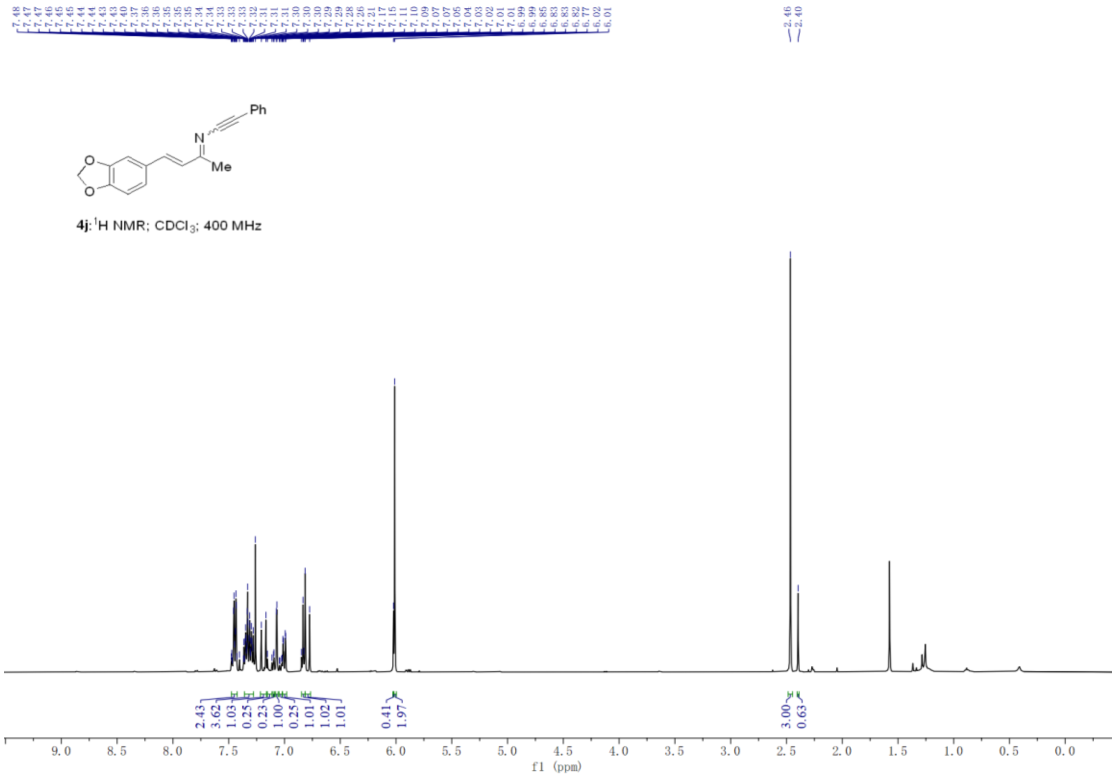

4j: ^{1}H NMR; $CDCl_3$; 400 MHz

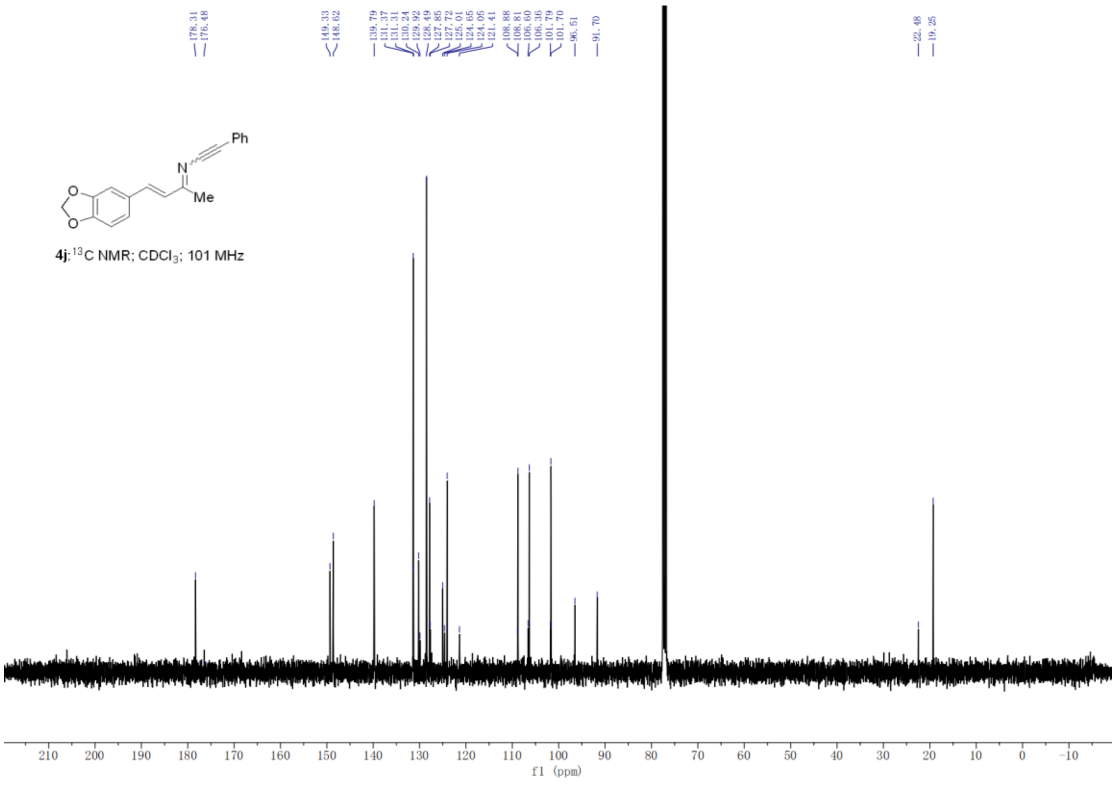

4j: ^{13}C NMR; $CDCl_3$; 101 MHz

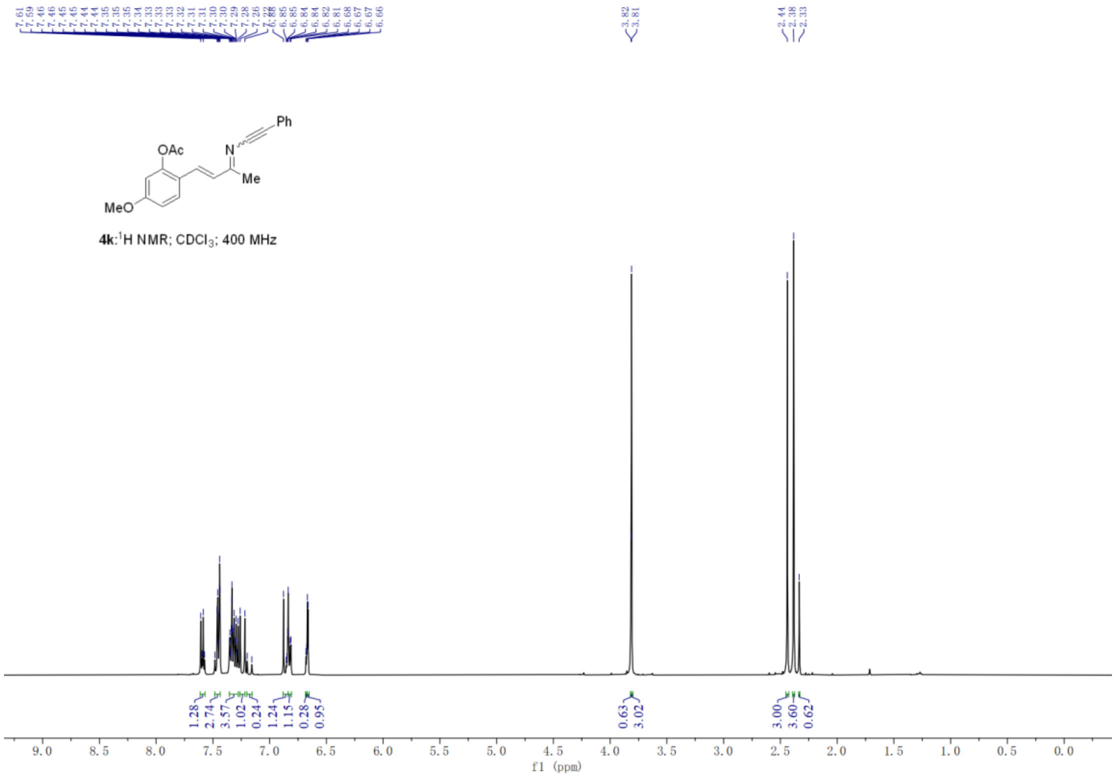

4k: ¹H NMR; $CDCl_3$; 400 MHz

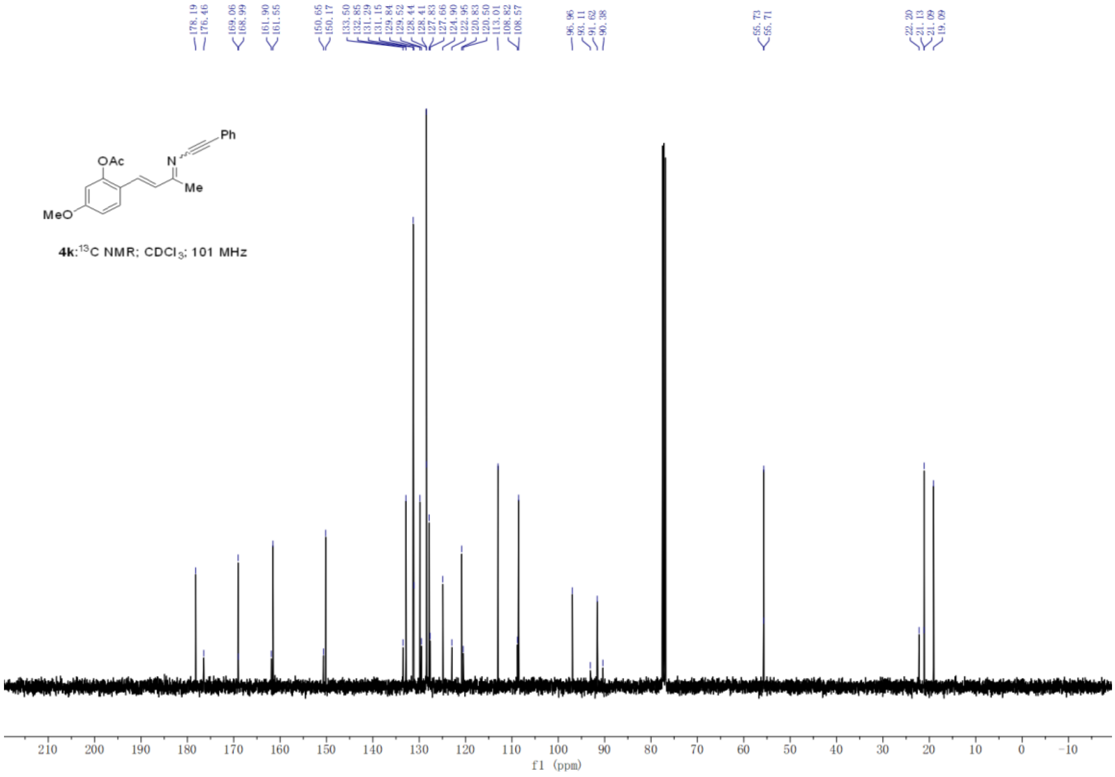

4k: ¹³C NMR; $CDCl_3$; 101 MHz

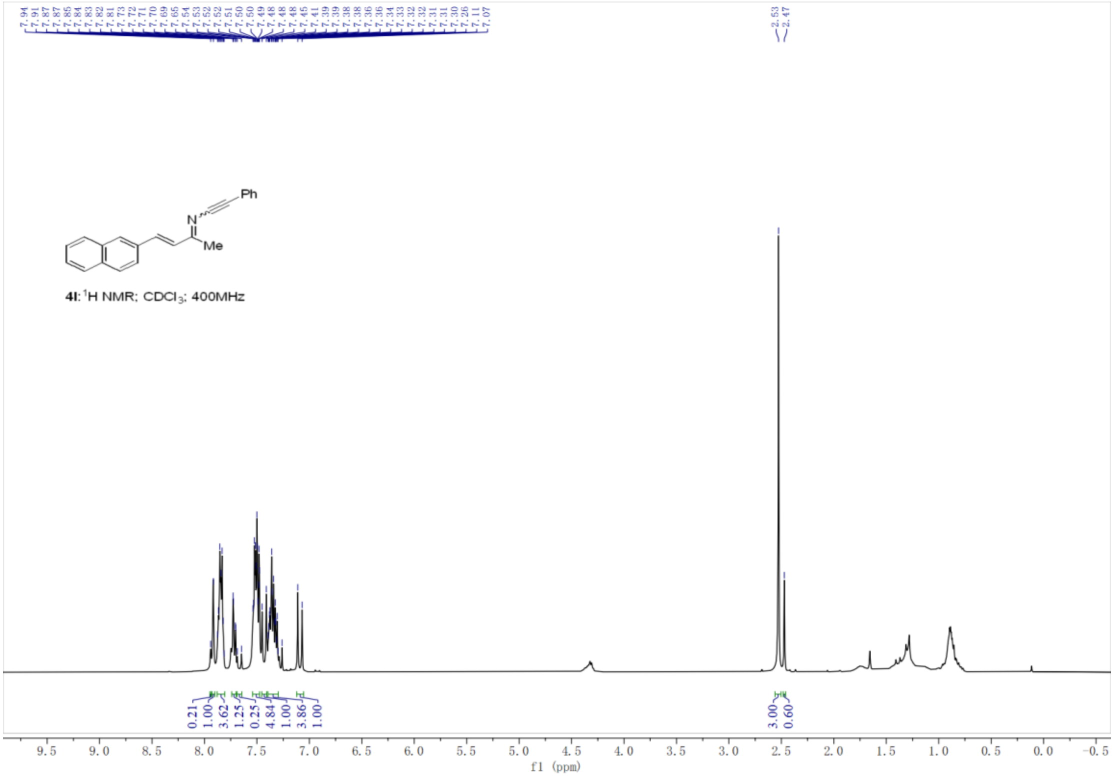
7.94
7.91
7.87
7.85
7.84
7.83
7.82
7.81
7.73
7.72
7.71
7.70
7.69
7.65
7.54
7.53
7.52
7.52
7.51
7.50
7.50
7.49
7.48
7.48
7.48
7.45
7.41
7.39
7.39
7.38
7.38
7.36
7.36
7.34
7.33
7.32
7.32
7.31
7.31
7.30
7.26
7.11
7.07
2.53
2.47
Ph
N
Me
4l: 1H NMR; CDCl3; 400MHz
0.21
1.00
3.62
1.25
0.25
4.84
1.00
3.86
1.00
3.00
0.60
9.5 9.0 8.5 8.0 7.5 7.0 6.5 6.0 5.5 5.0 4.5 4.0 3.5 3.0 2.5 2.0 1.5 1.0 0.5 0.0 -0.5
f1 (ppm)

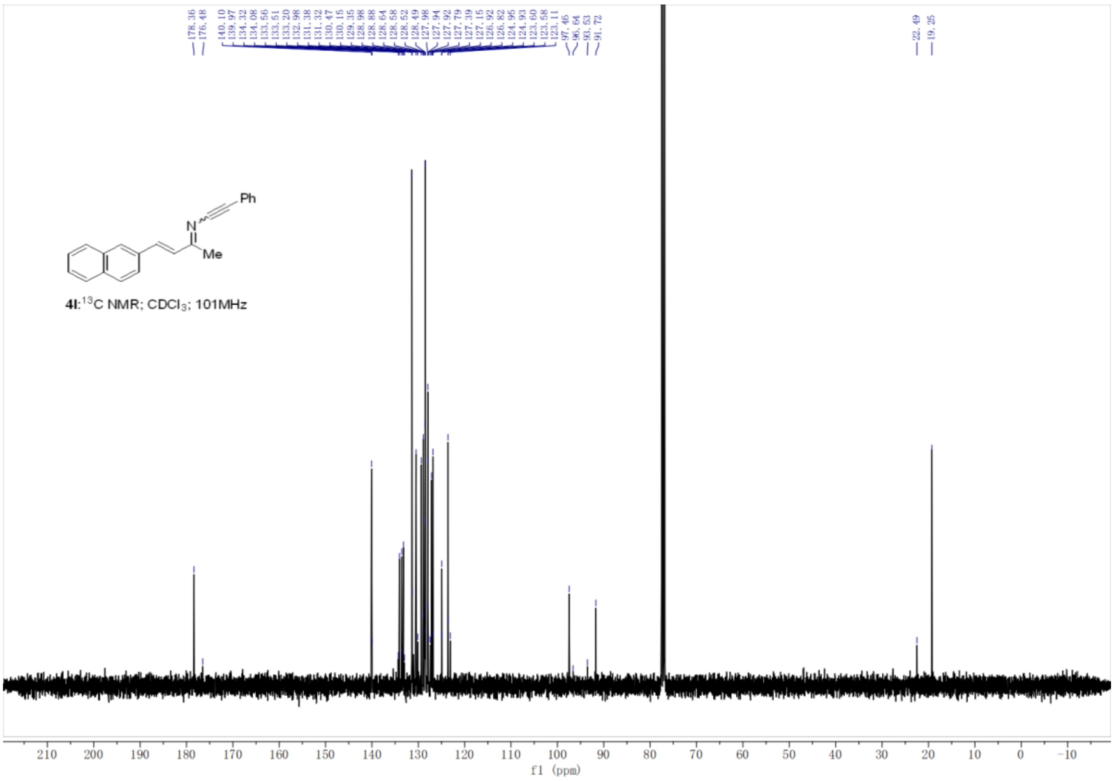
178.36
176.48
140.10
139.97
134.32
134.08
133.56
133.51
133.20
132.98
131.38
131.32
130.47
130.15
129.35
128.98
128.88
128.64
128.58
128.52
128.49
127.98
127.94
127.92
127.79
127.39
127.15
126.92
126.82
124.95
124.93
123.60
123.58
123.11
97.46
96.64
93.53
91.72
22.49
19.25
Ph
N
Me
4l: 13C NMR; CDCl3; 101MHz
210 200 190 180 170 160 150 140 130 120 110 100 90 80 70 60 50 40 30 20 10 0 -10
f1 (ppm)

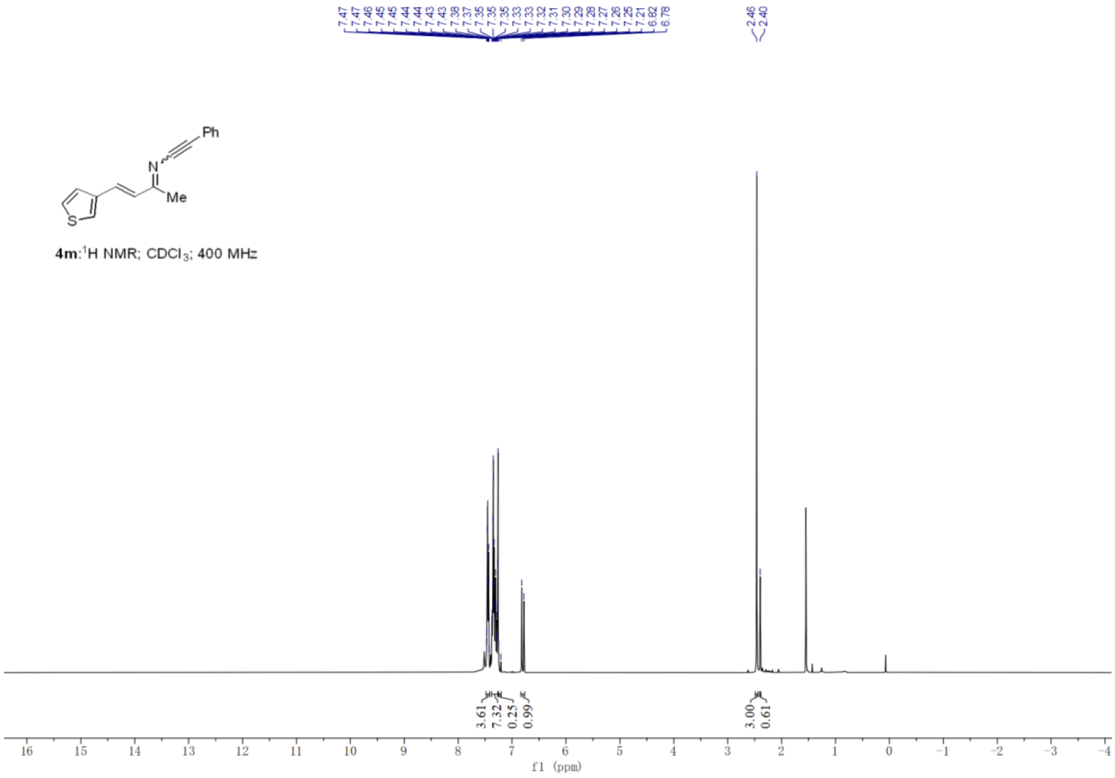
7.47 7.47 7.46 7.45 7.45 7.44 7.44 7.43 7.43 7.38 7.37 7.35 7.35 7.35 7.33 7.33 7.32 7.31 7.30 7.29 7.28 7.27 7.26 7.25 7.21 6.82 6.78
2.46 2.40
Ph
N
Me
S
4m: 1H NMR; CDCl3; 400 MHz
3.61 7.32 0.25 0.99
3.00 0.61
f1 (ppm)

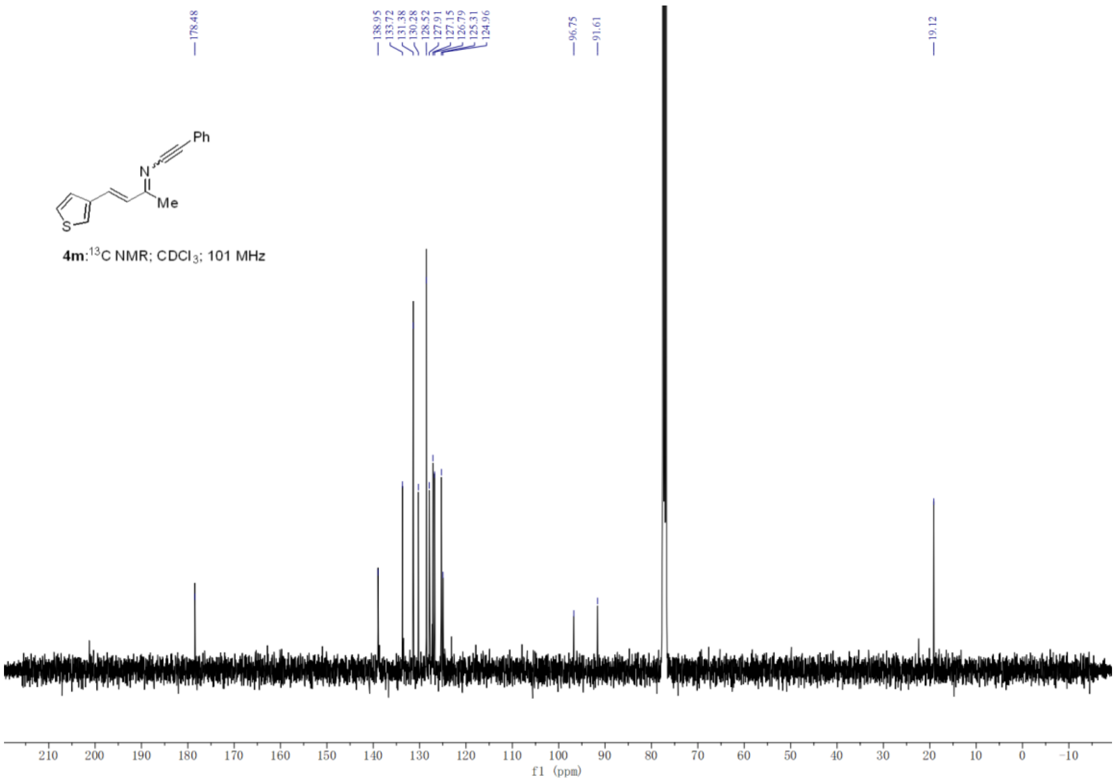
178.48
138.95 133.72 131.38 130.28 128.52 127.91 127.15 126.79 125.31 124.96
96.75 91.61
19.12
Ph
N
Me
S
4m: 13C NMR; CDCl3; 101 MHz
f1 (ppm)

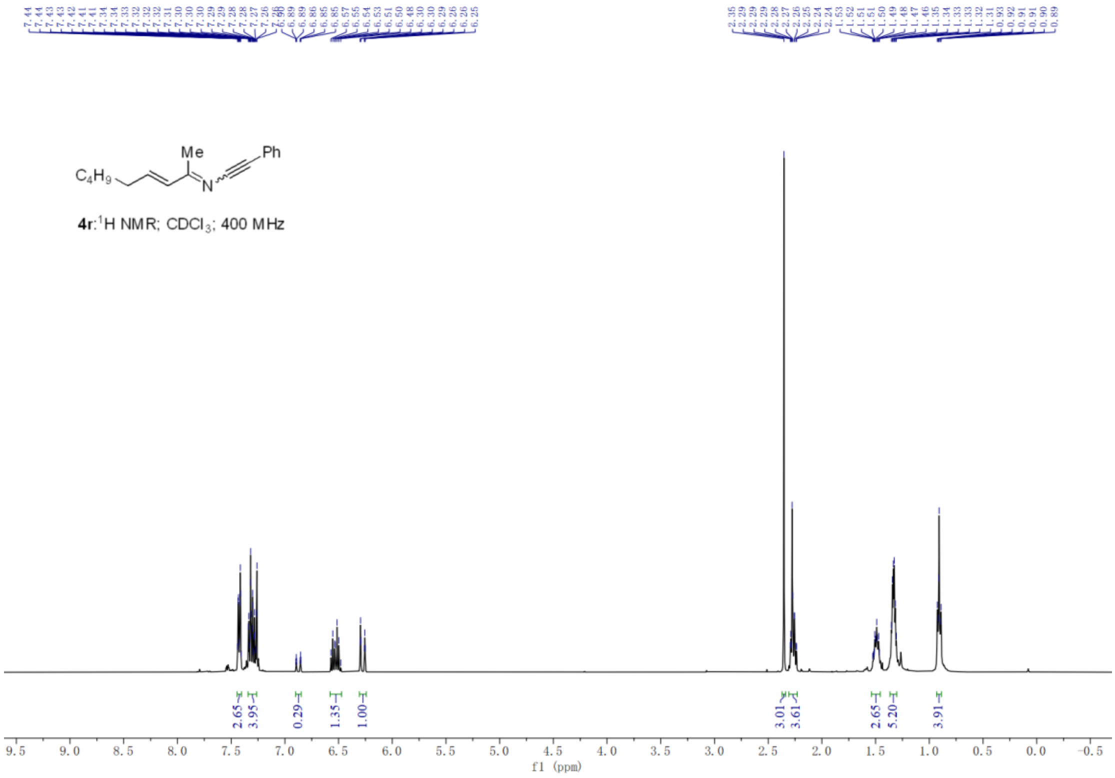
Me
Ph
C_4H_9
N
4r: ¹H NMR; CDCl₃; 400 MHz
f1 (ppm)

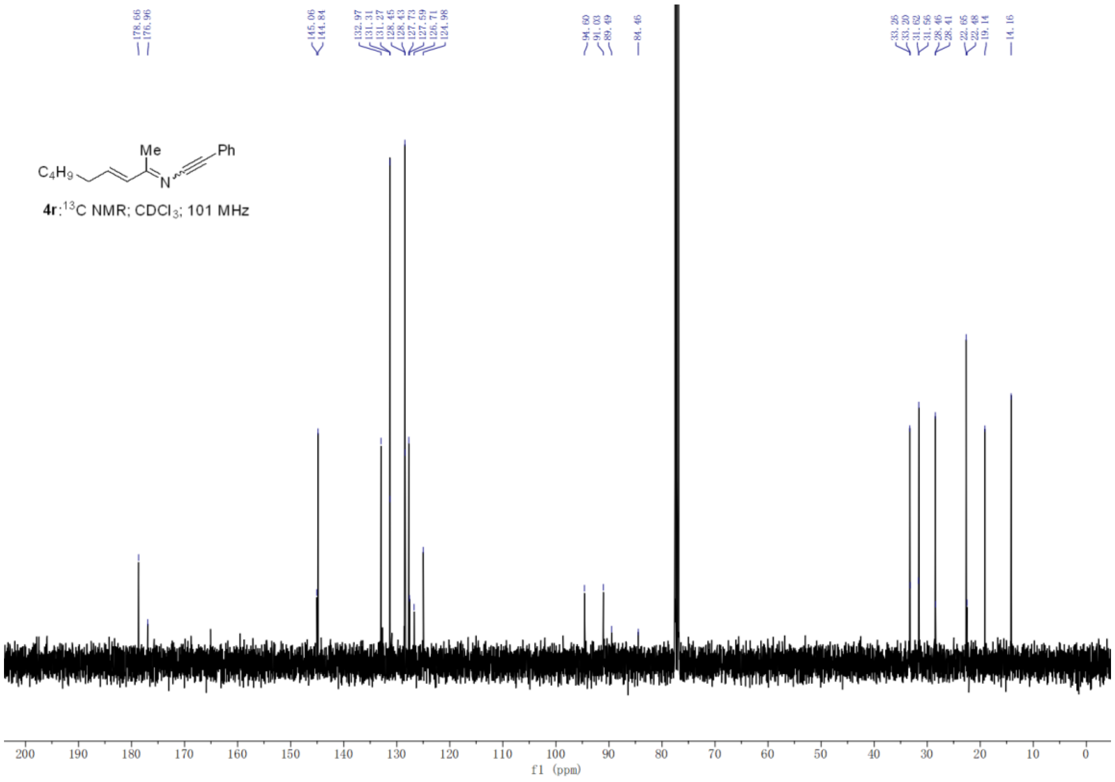
Me
Ph
C_4H_9
N
4r: ¹³C NMR; CDCl₃; 101 MHz
f1 (ppm)

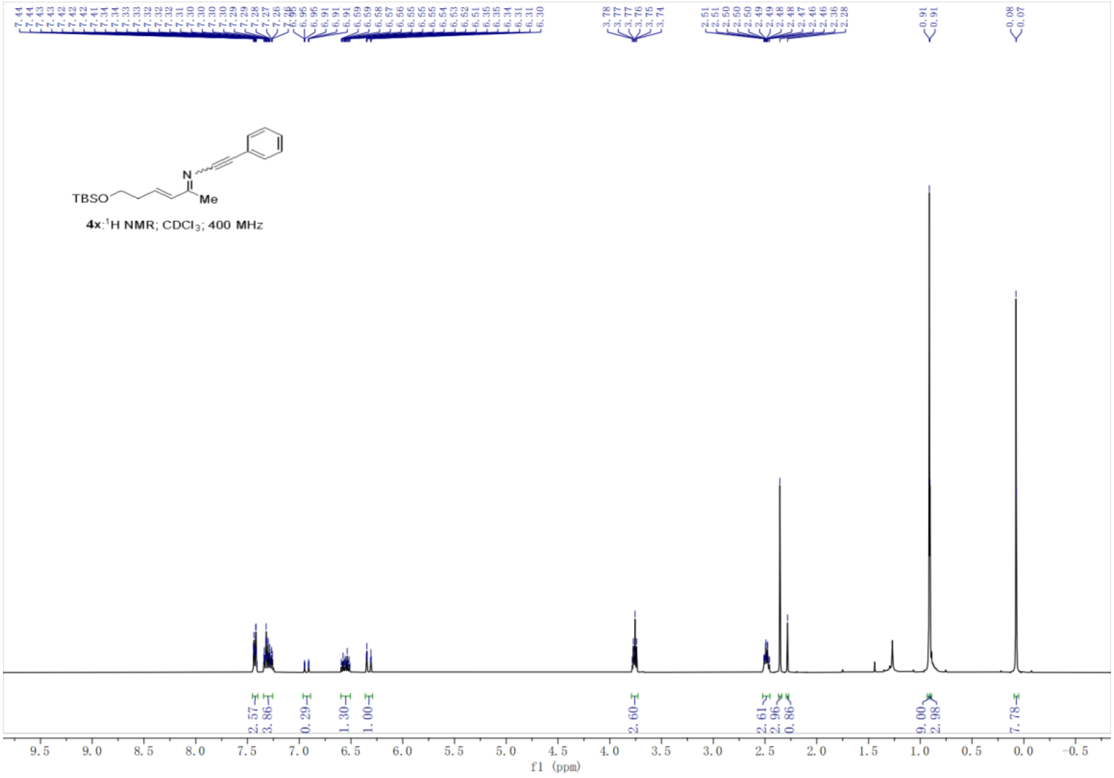
TBSO
N
Me
4x: ¹H NMR; CDCl₃; 400 MHz
f1 (ppm)

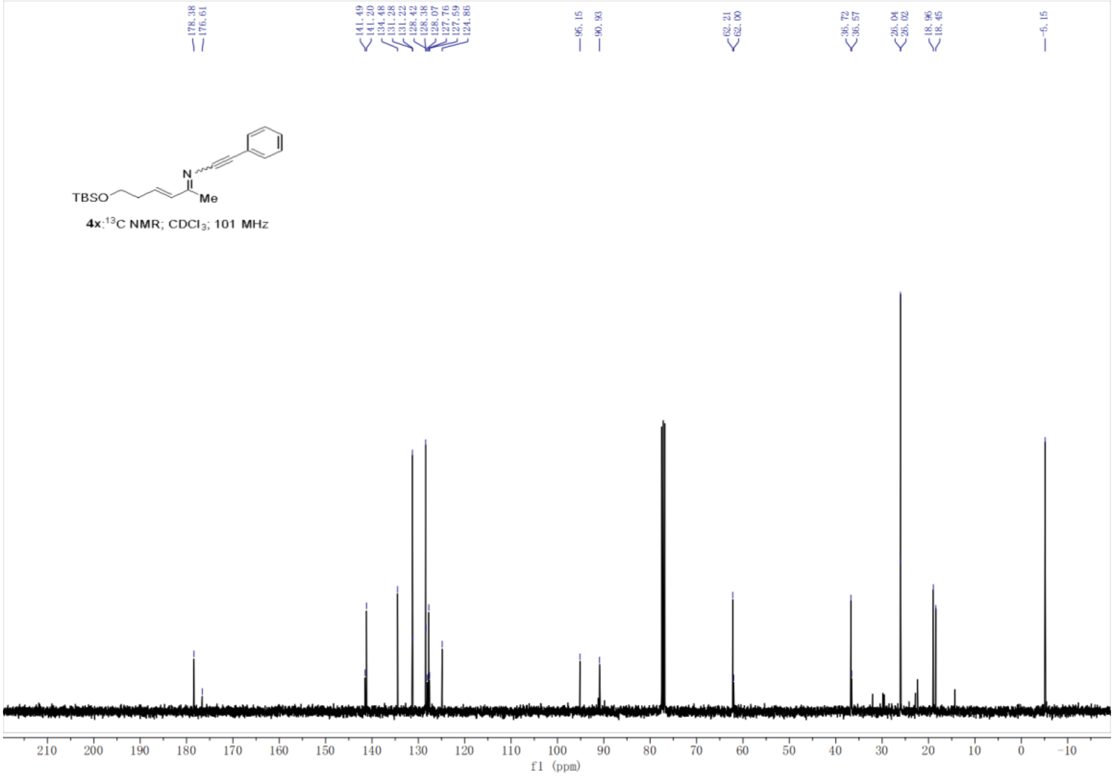
TBSO
N
Me
4x: ¹³C NMR; CDCl₃; 101 MHz
f1 (ppm)

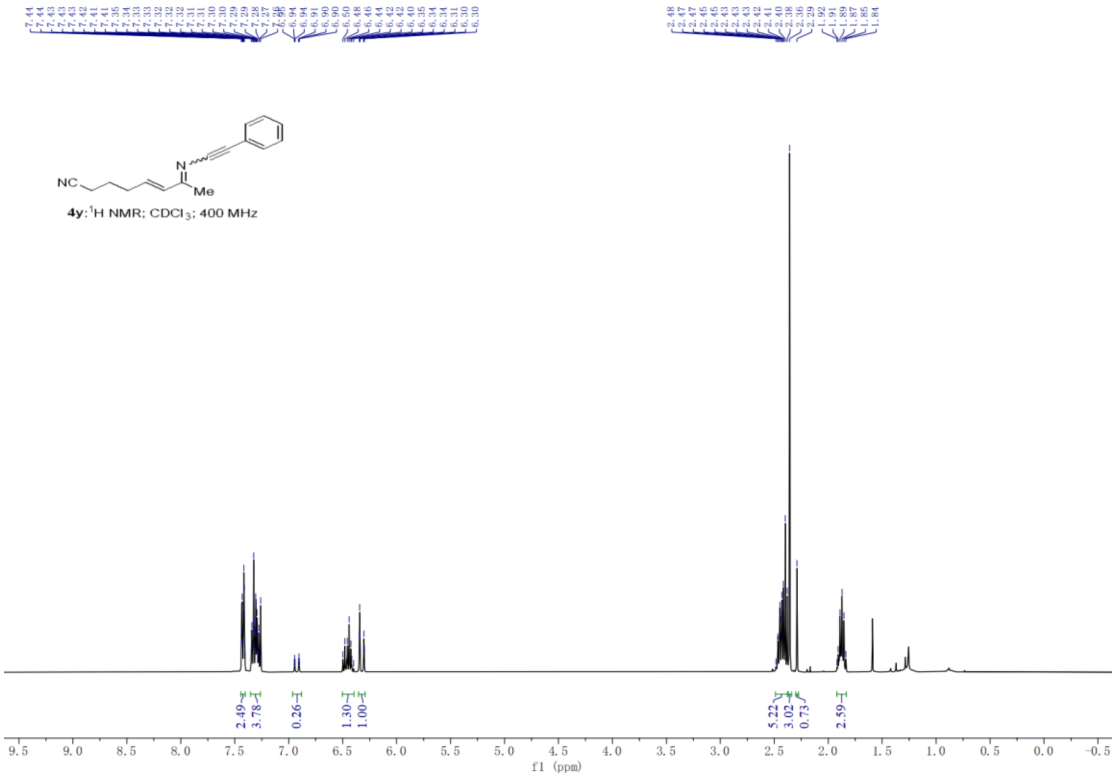
4y: 1H NMR; CDCl3; 400 MHz
f1 (ppm)

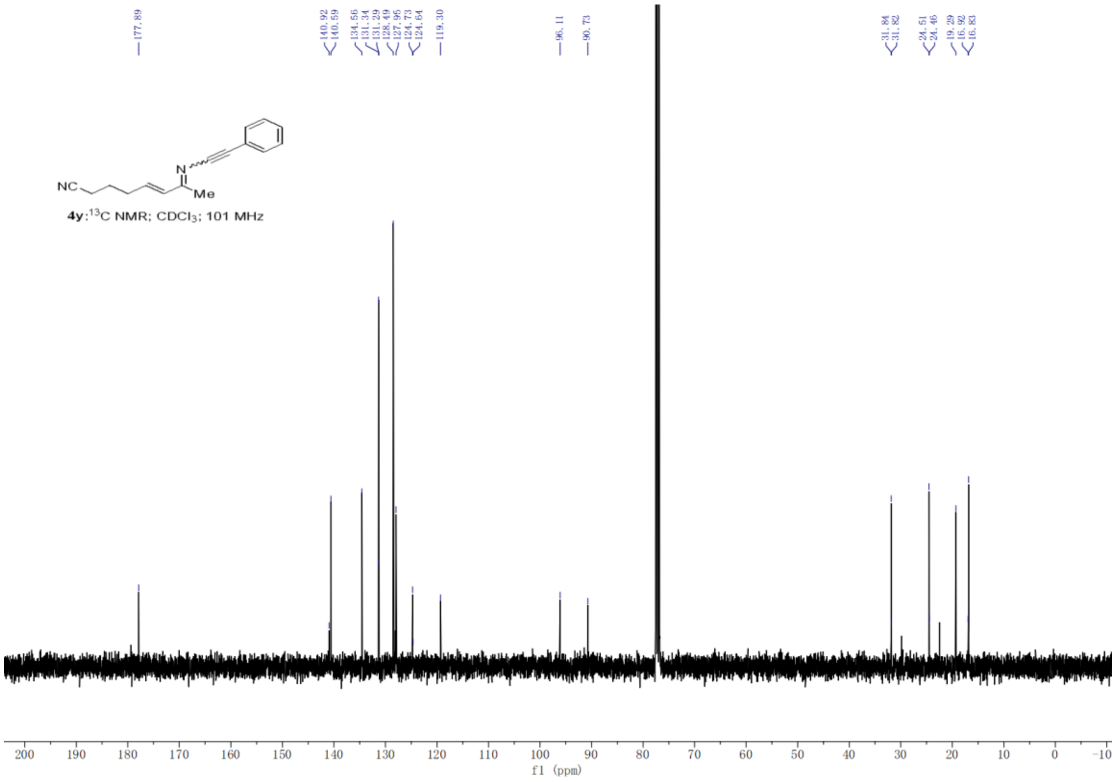
4y: 13C NMR; CDCl3; 101 MHz
f1 (ppm)

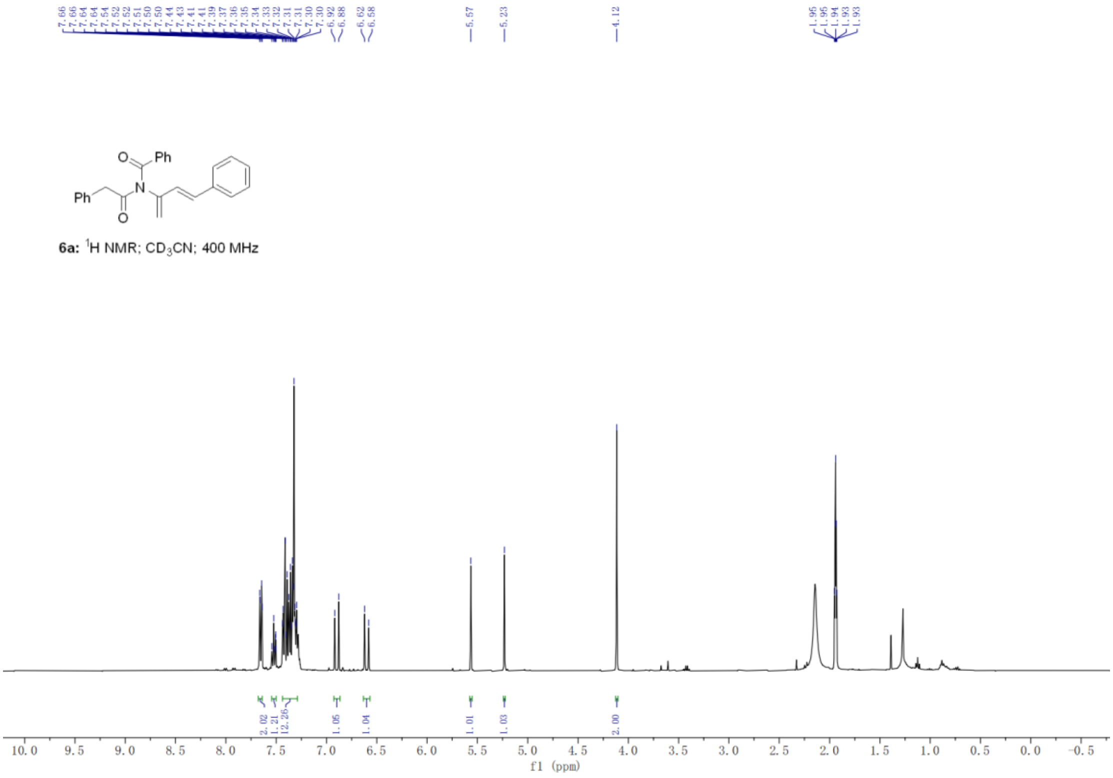

6a: ^{1}H NMR; CD_3CN; 400 MHz

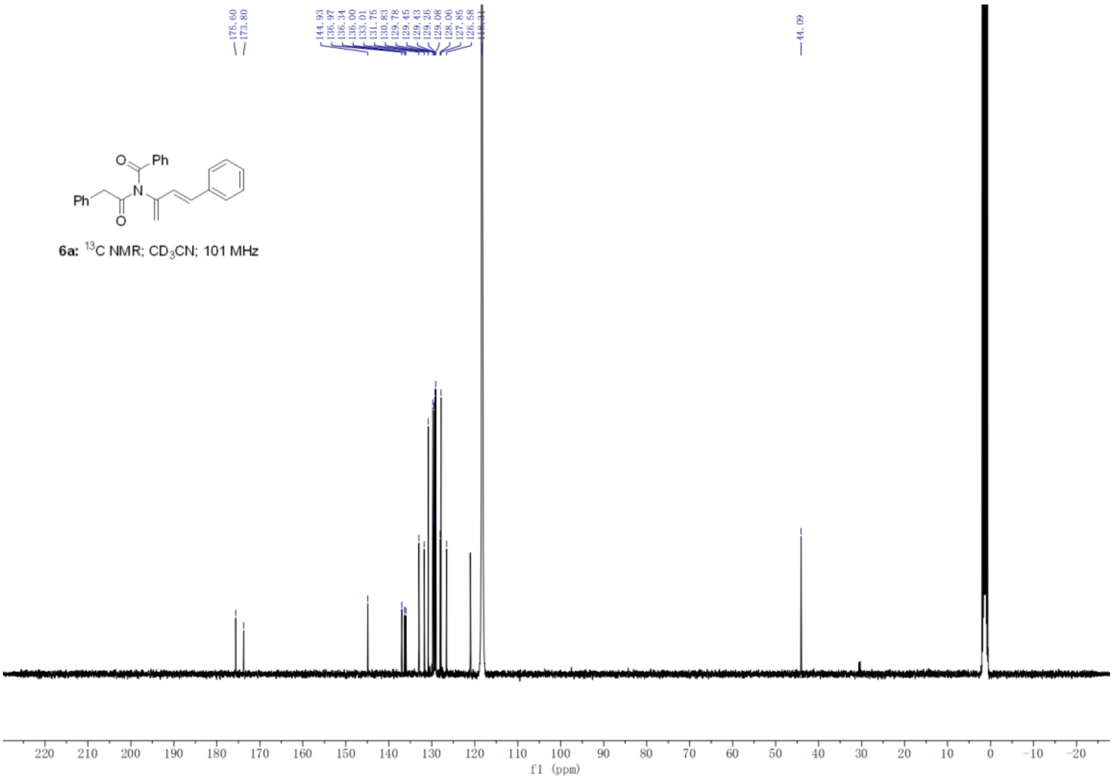

6a: ^{13}C NMR; CD_3CN; 101 MHz

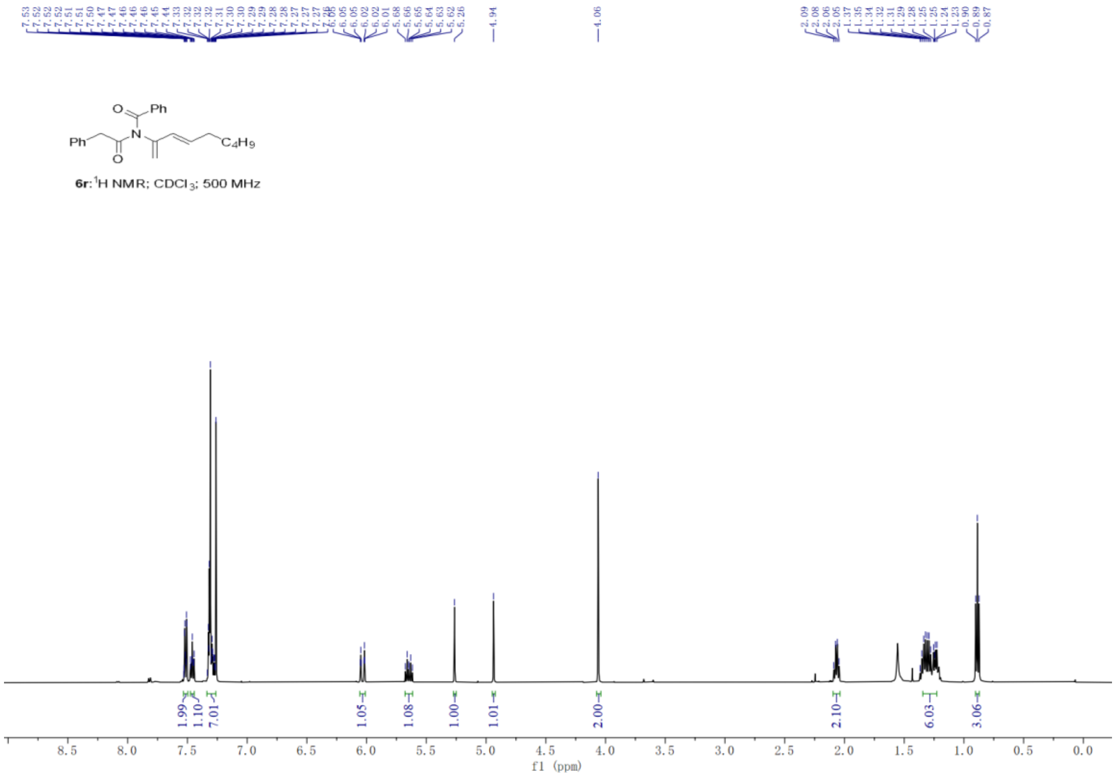

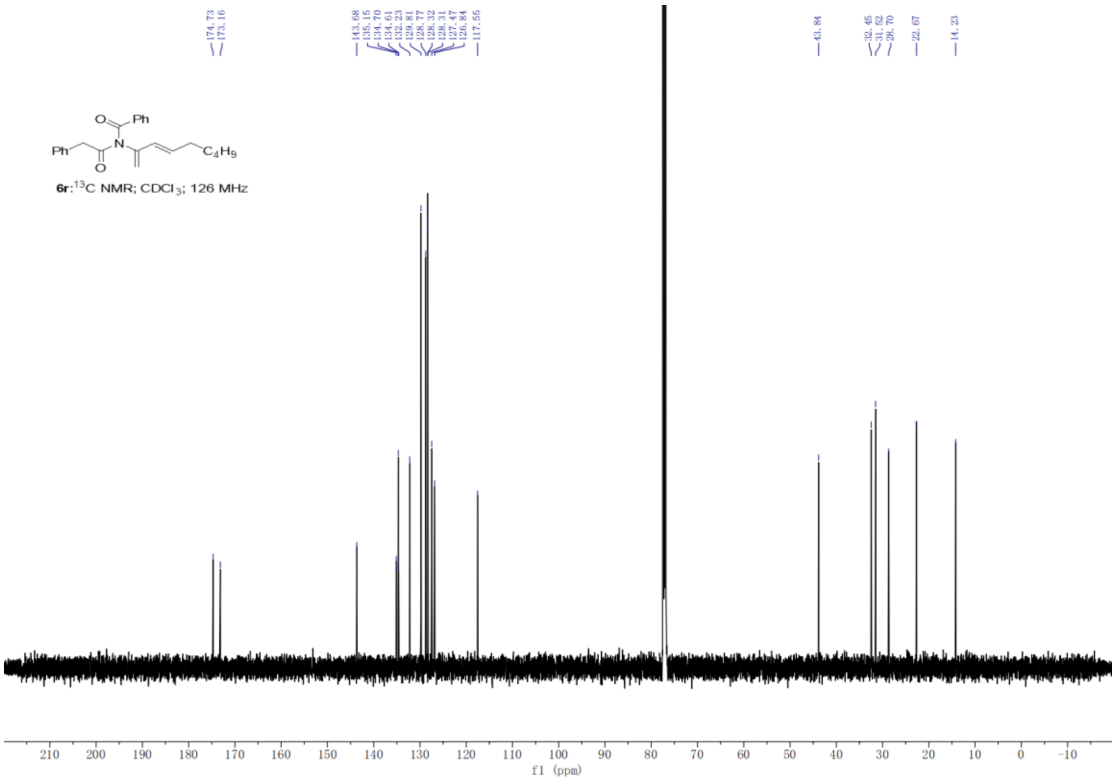

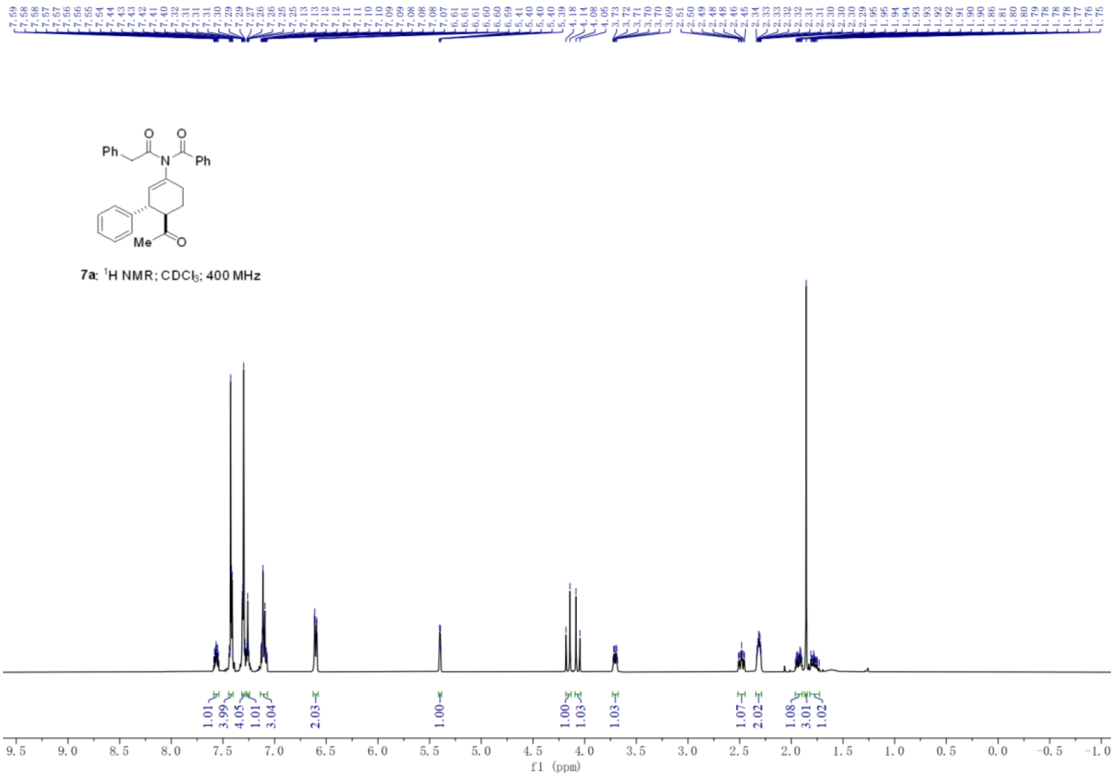

7a: ^{1}H NMR; $CDCl_3$; 400 MHz

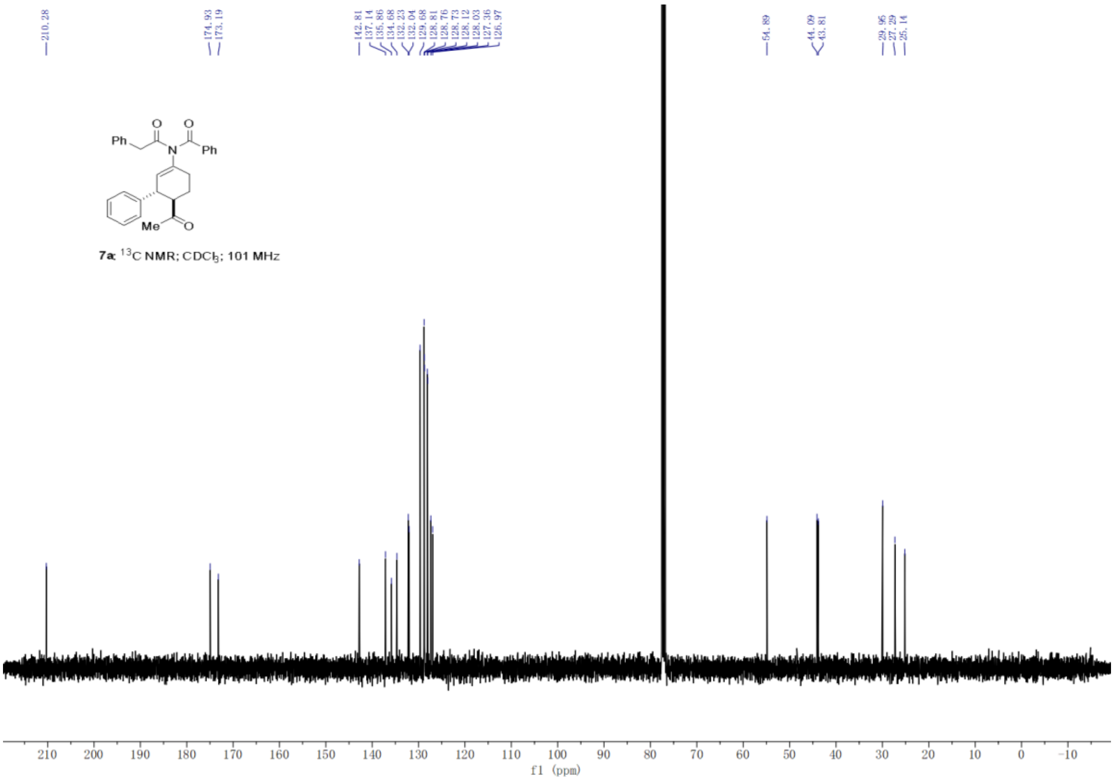

7a: ^{13}C NMR; $CDCl_3$; 101 MHz

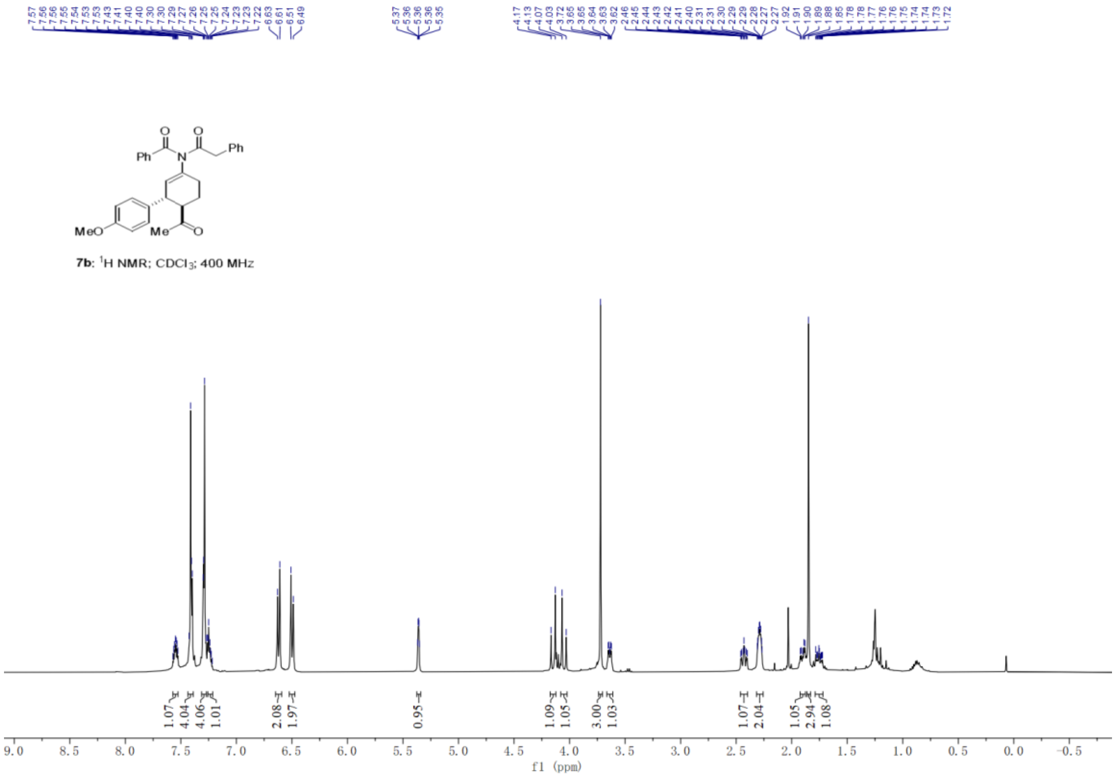
7b: ¹H NMR; CDCl₃; 400 MHz
f1 (ppm)

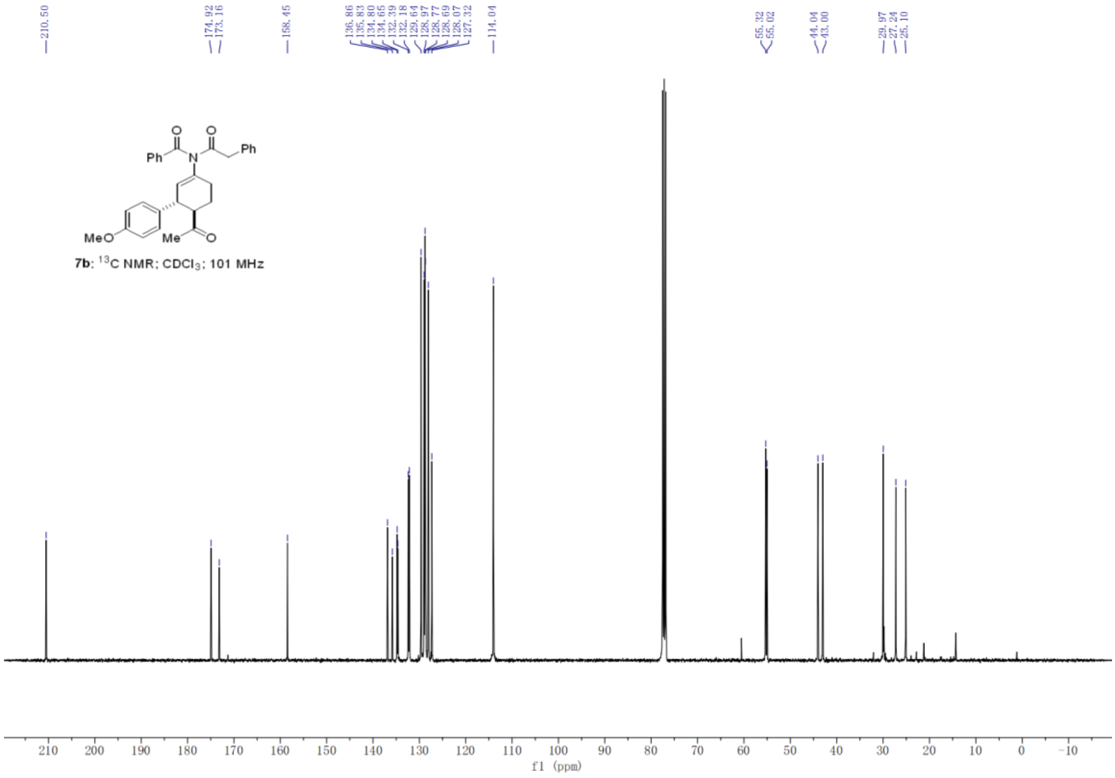
7b: ¹³C NMR; CDCl₃; 101 MHz
f1 (ppm)

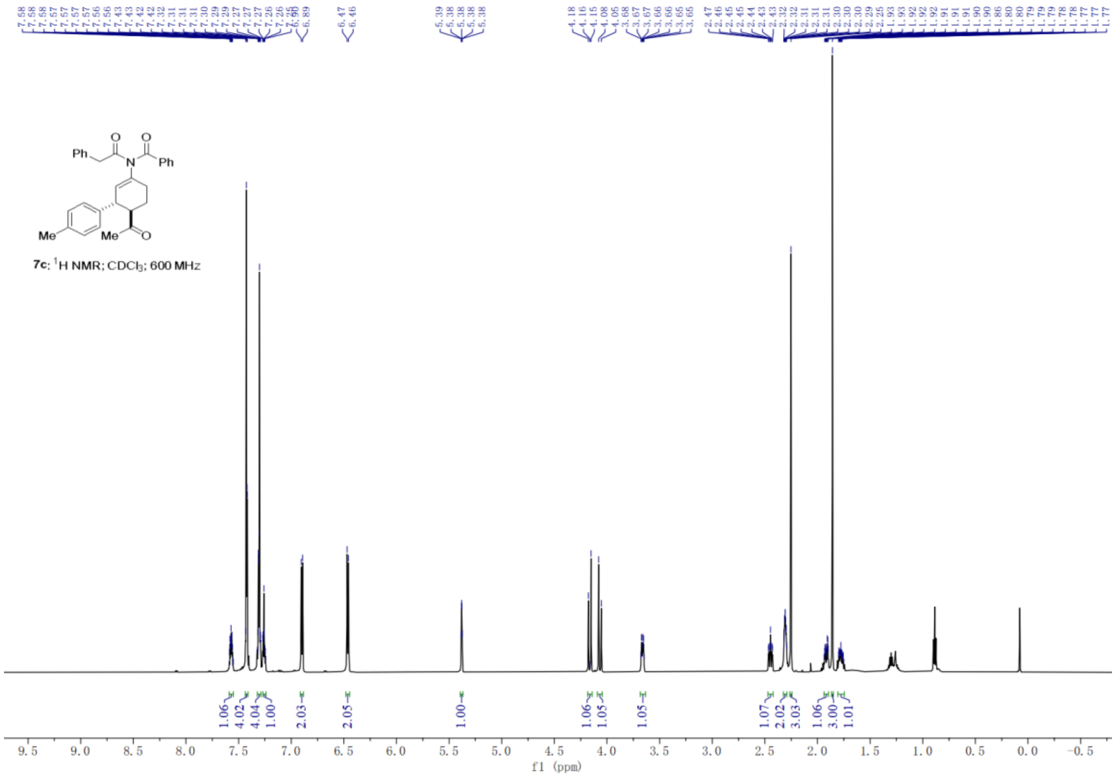
7c; 1H NMR; CDCl3; 600 MHz
f1 (ppm)

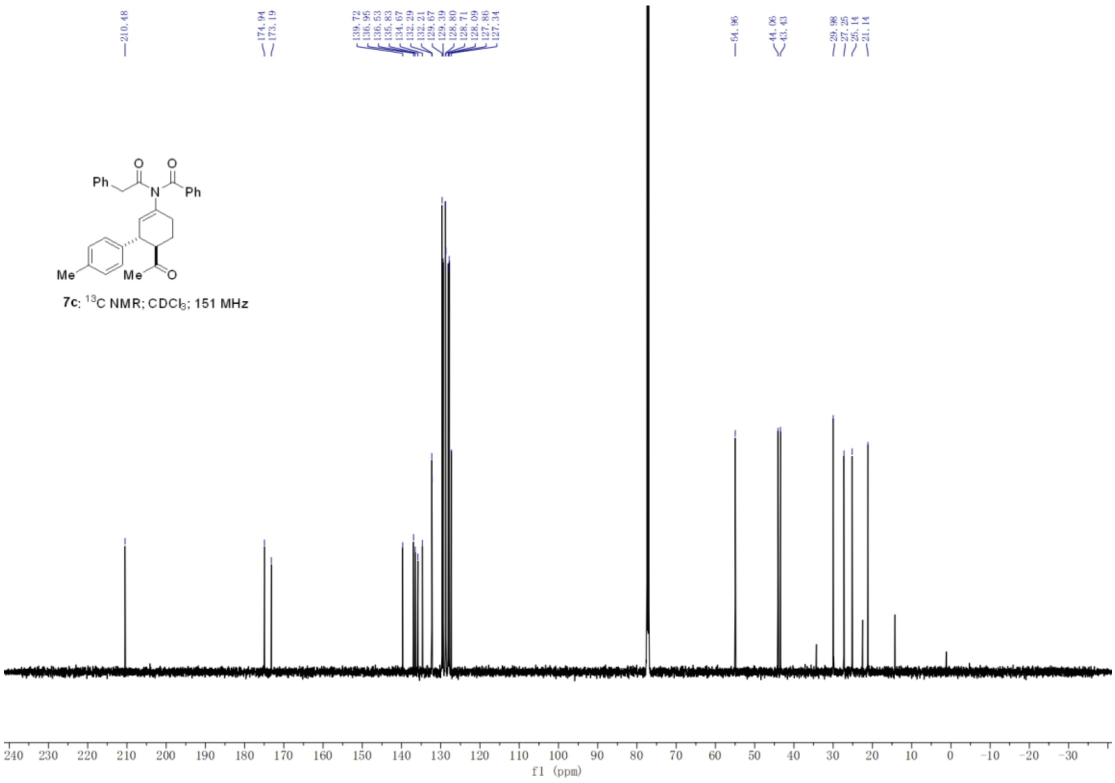
7c; 13C NMR; CDCl3; 151 MHz
f1 (ppm)

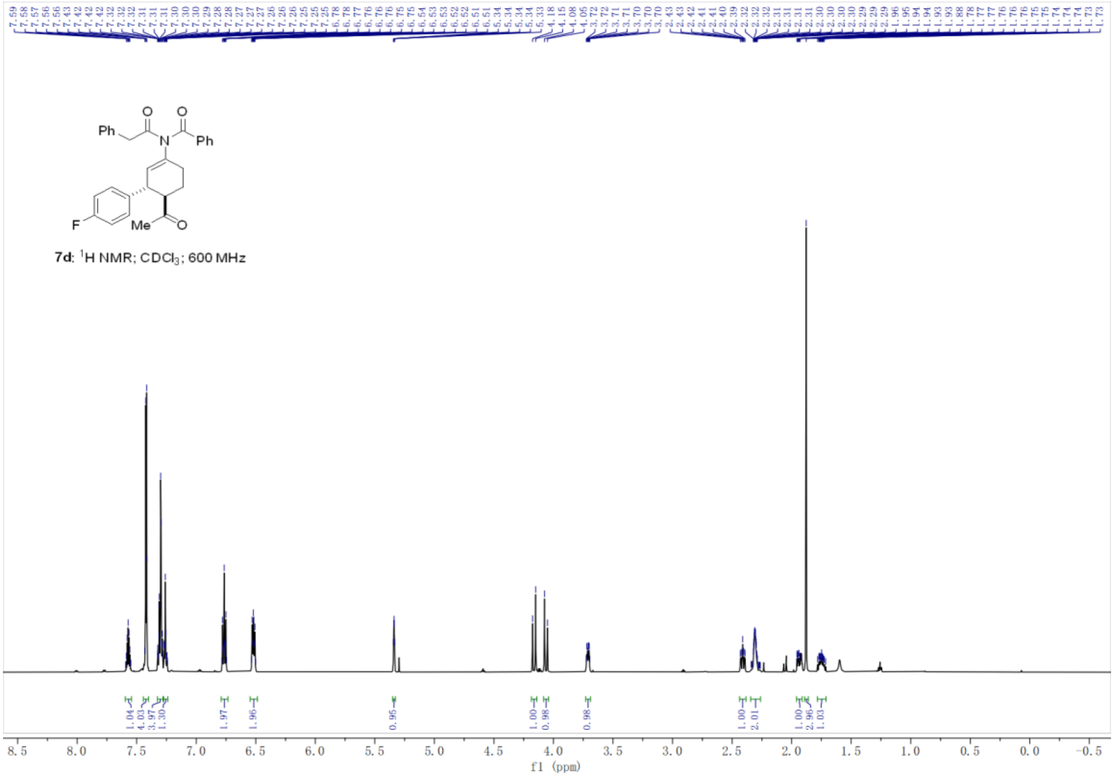
7d: 1H NMR; CDCl3; 600 MHz
f1 (ppm)

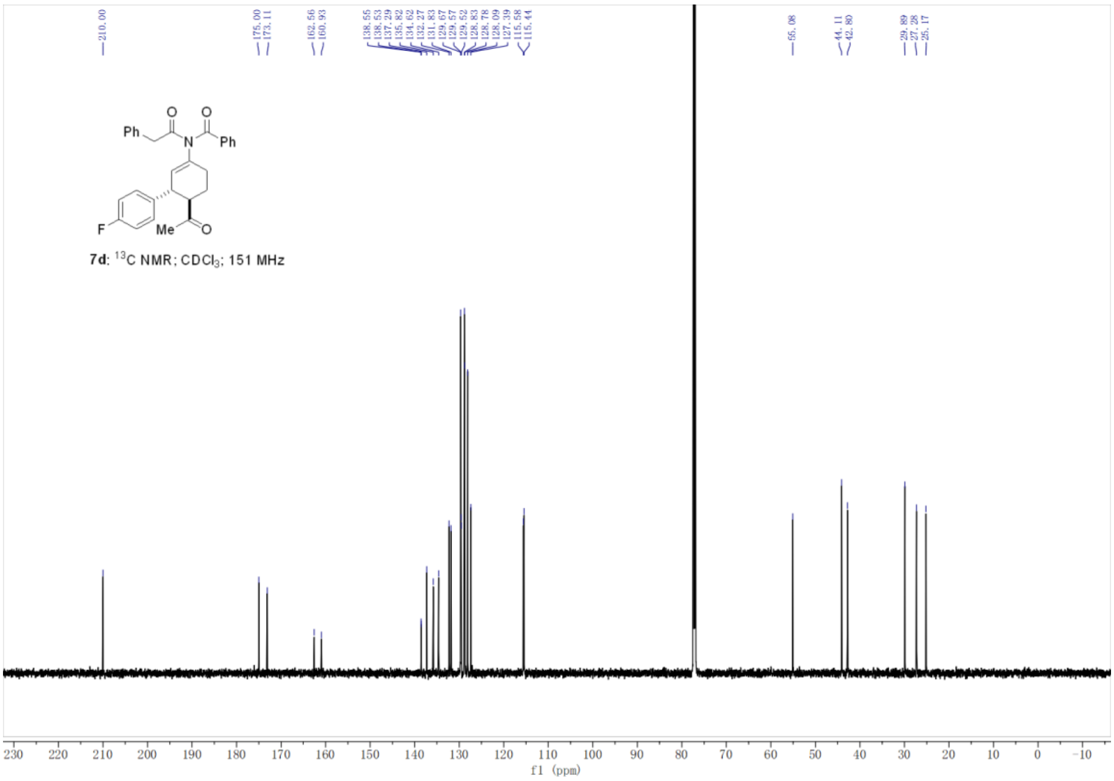
7d: 13C NMR; CDCl3; 151 MHz
f1 (ppm)

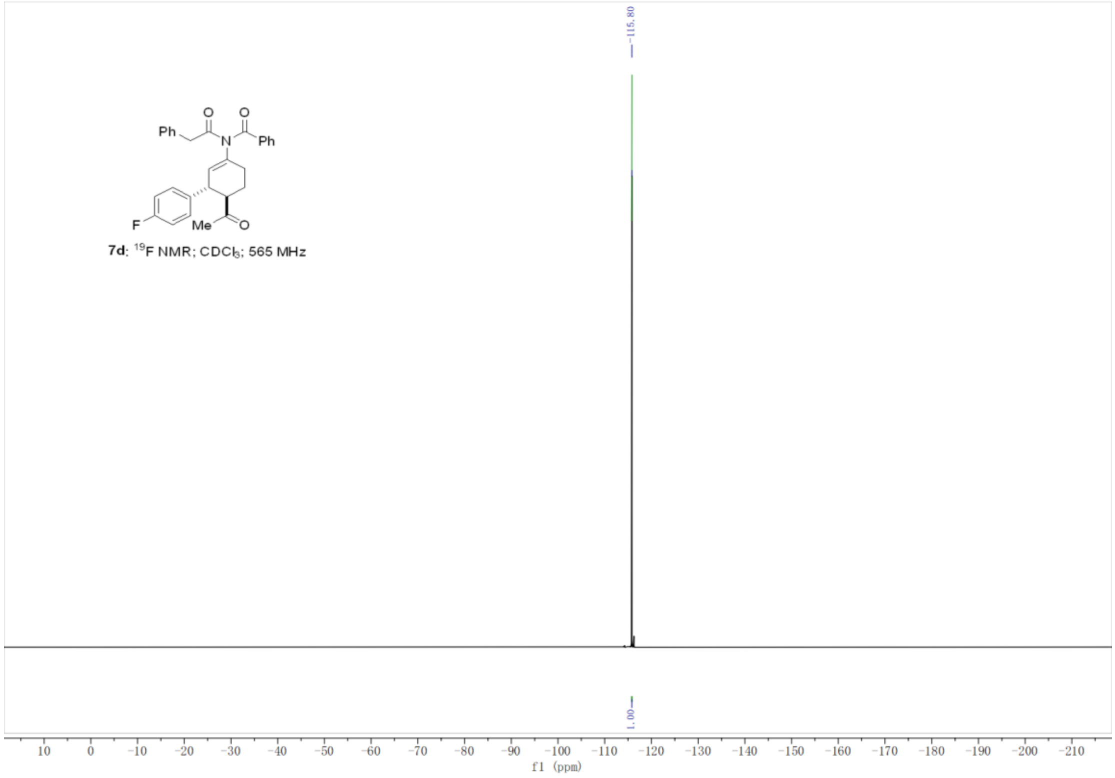
Ph
Ph
N
F
Me
7d: 19F NMR; CDCl3; 565 MHz
f1 (ppm)

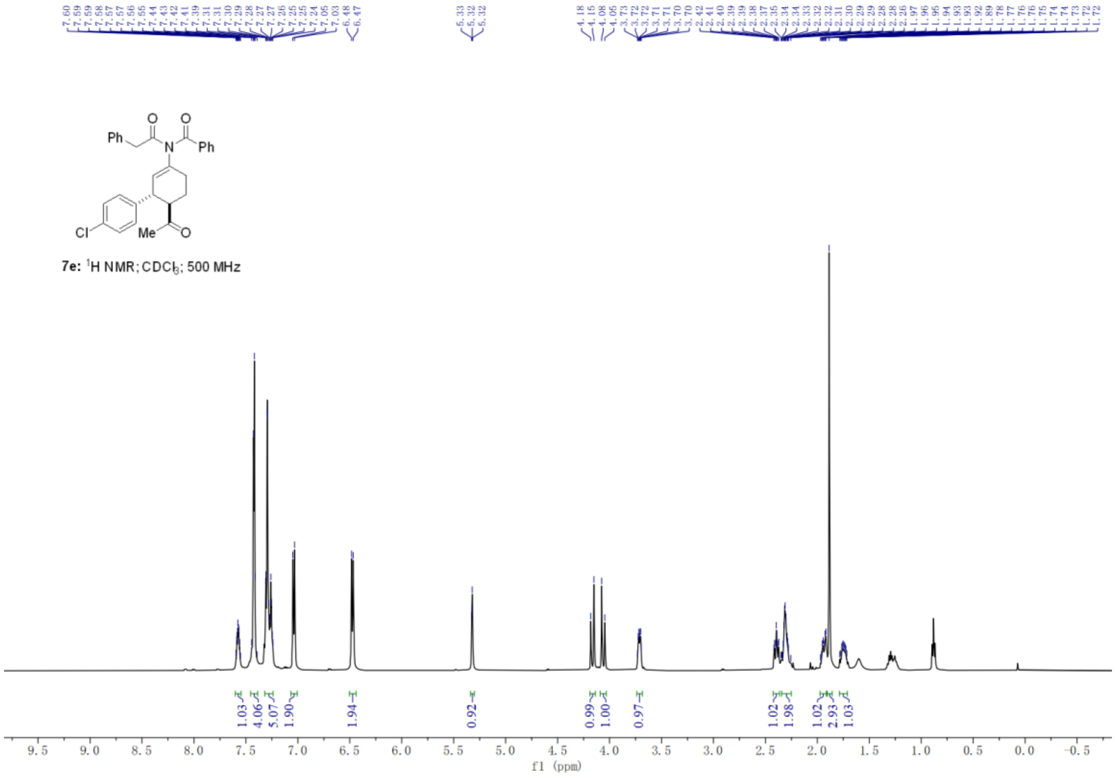
Ph
Ph
N
Cl
Me
7e: 1H NMR; CDCl3; 500 MHz
f1 (ppm)

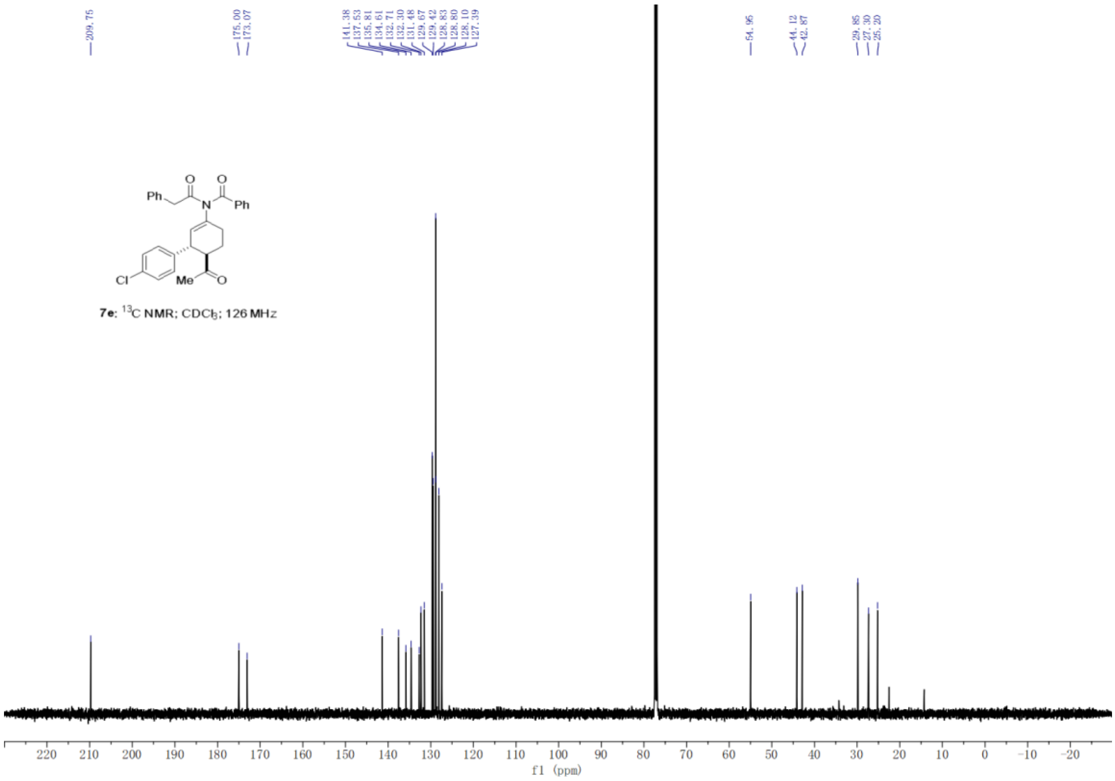
7e: ^{13}C NMR; CDCl$_3$; 126 MHz
f1 (ppm)

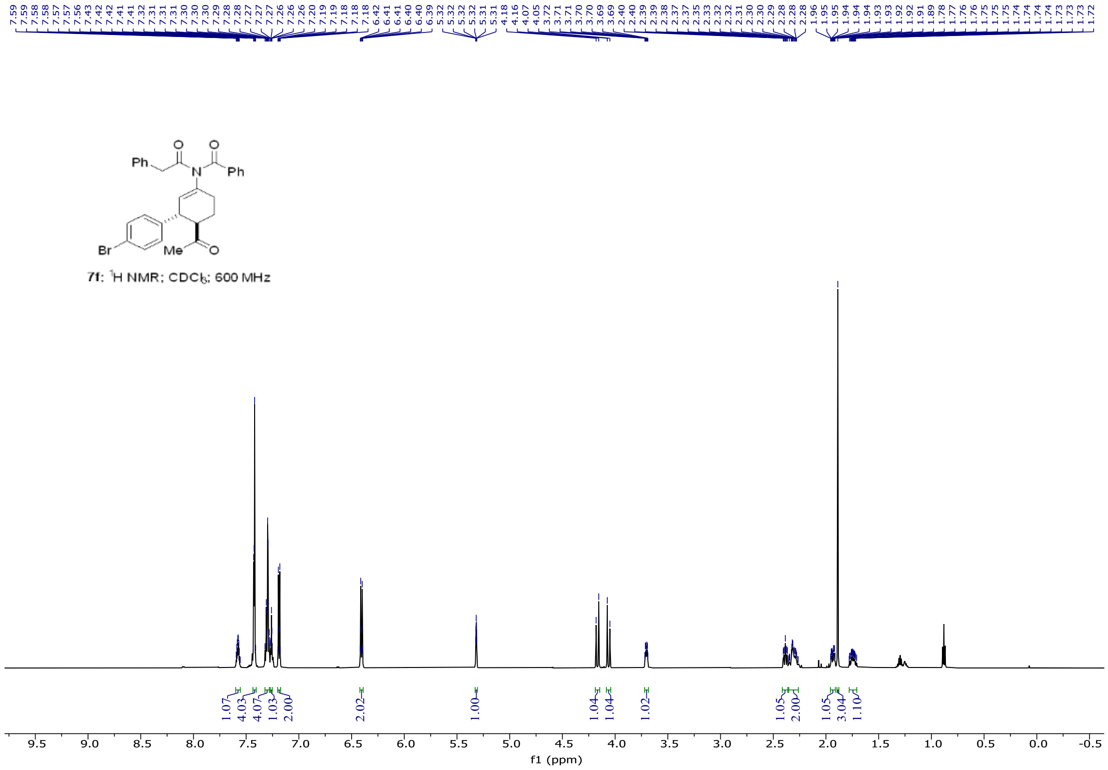
7f: ^{1}H NMR; CDCl$_3$; 600 MHz
f1 (ppm)

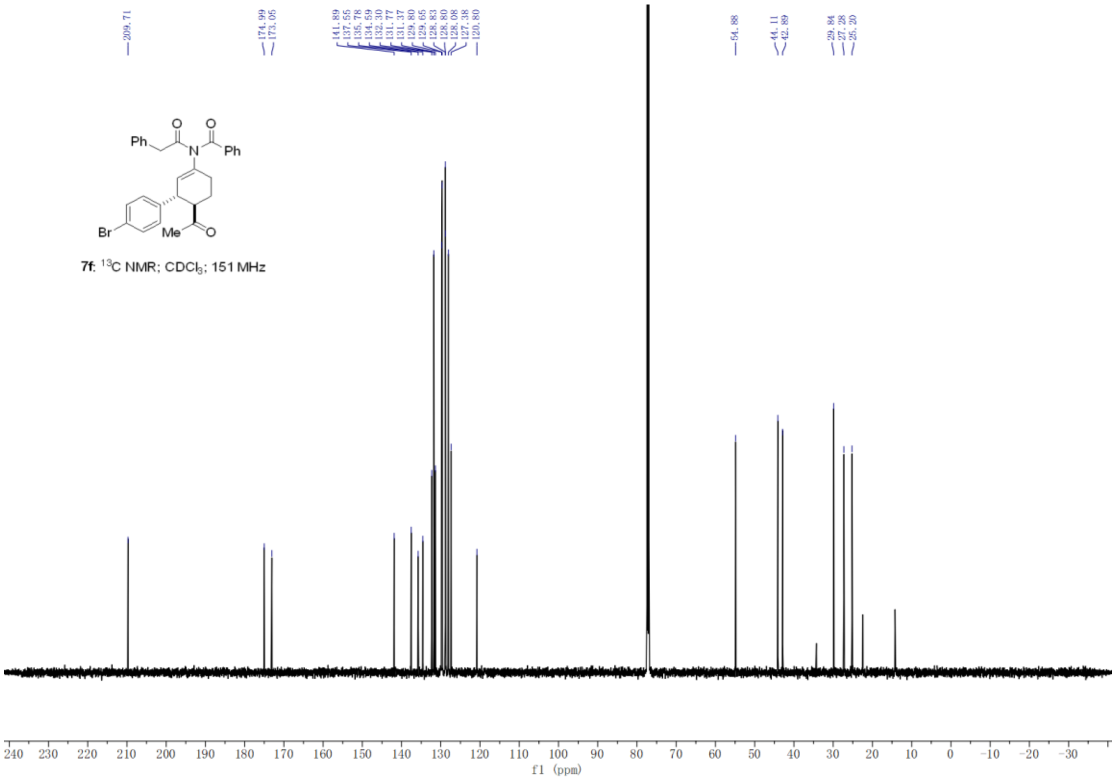
7f: ^{13}C NMR; CDCl$_3$; 151 MHz
209.71
174.99
173.05
141.89
137.55
135.78
134.59
132.30
131.77
131.37
129.80
129.65
128.83
128.80
128.08
127.38
120.80
54.88
44.11
42.89
29.84
27.28
25.20
f1 (ppm)

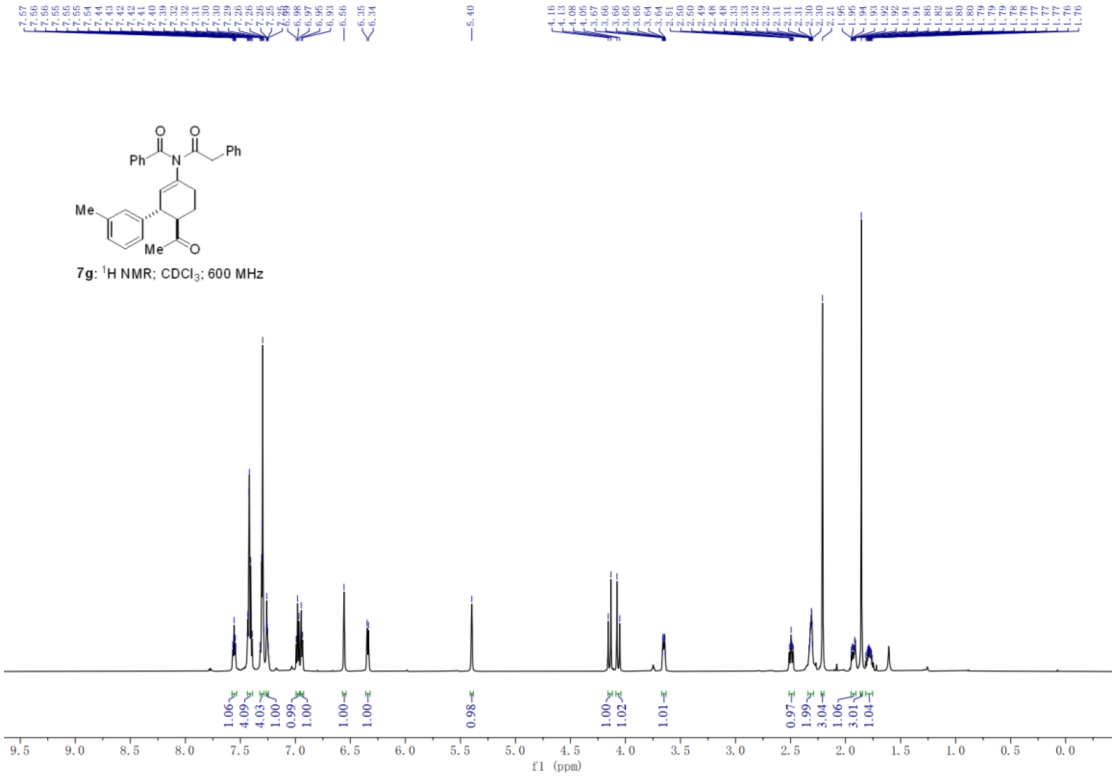
7g: ^{1}H NMR; CDCl$_3$; 600 MHz
f1 (ppm)

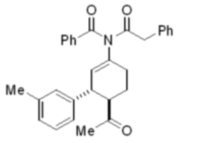

7g: ^{13}C NMR; $CDCl_3$; 151 MHz

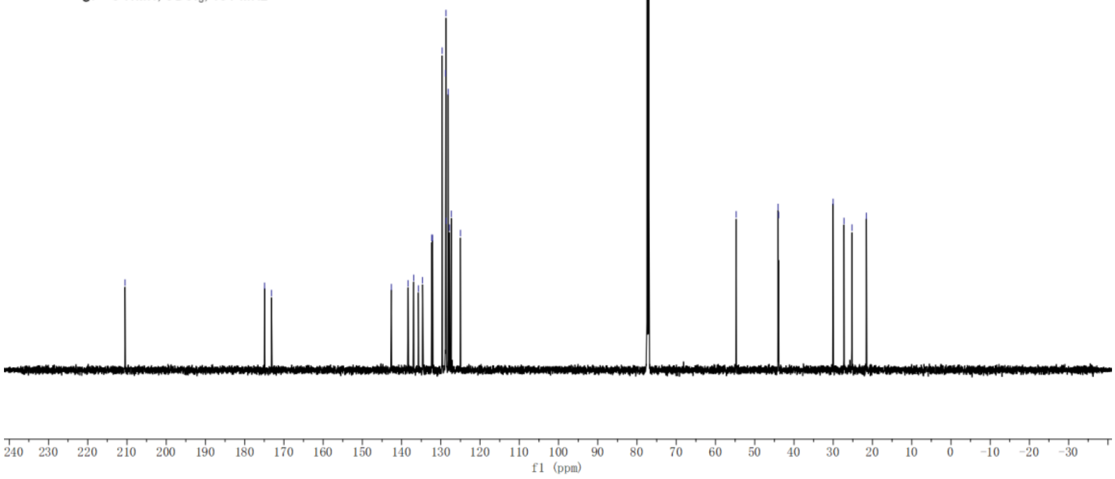

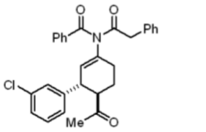

7h: ^{1}H NMR; $CDCl_3$; 400 MHz

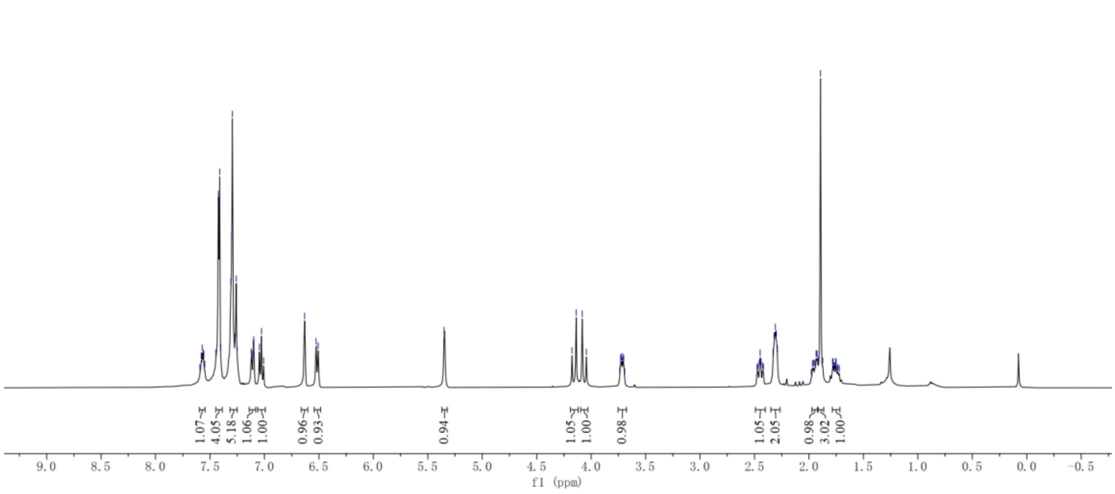

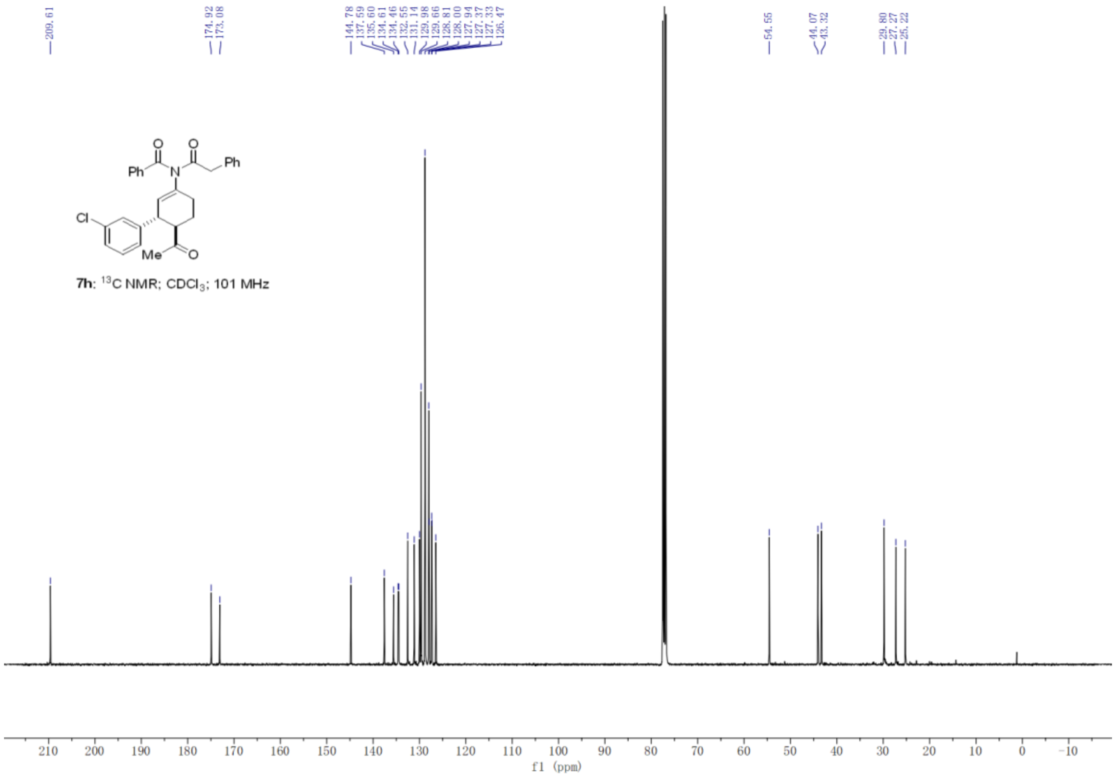

209.61
174.92
173.08
144.78
137.59
135.60
134.61
134.46
132.55
131.14
129.98
129.86
128.81
128.00
127.94
127.37
127.33
126.47
54.55
44.07
43.32
29.80
27.27
25.22
Ph
Cl
Me
N
O
7h: 13C NMR; CDCl3; 101 MHz
f1 (ppm)

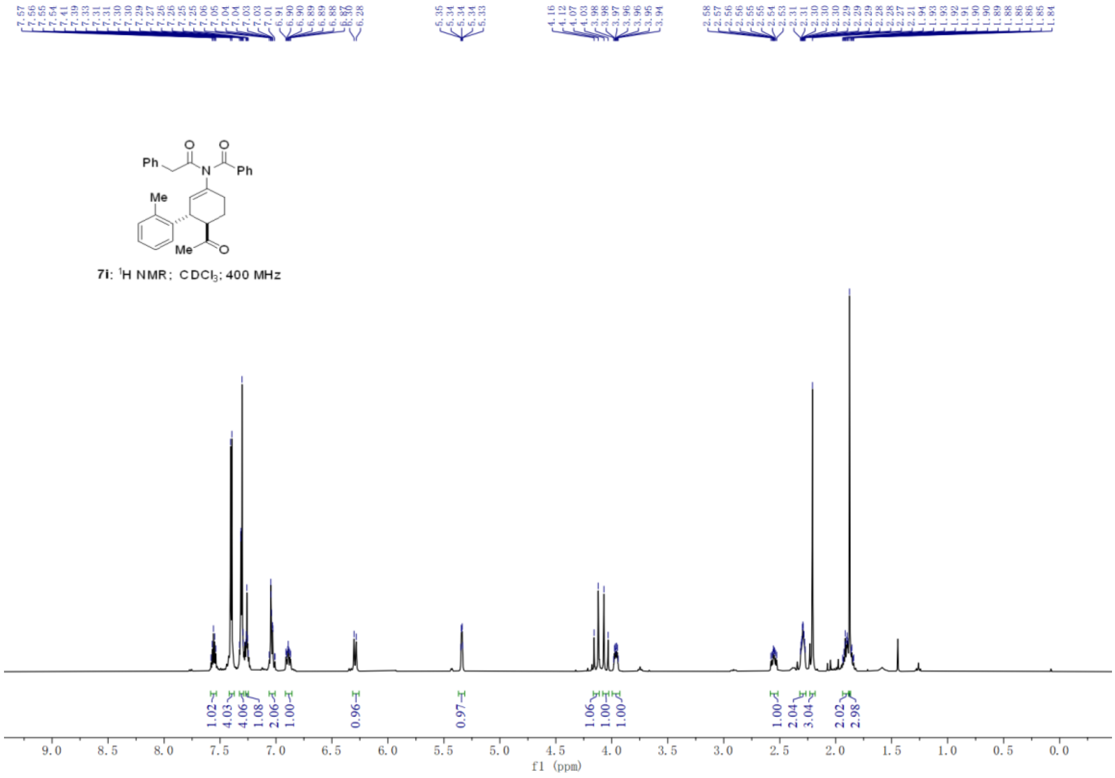

Ph
Me
N
O
7i: 1H NMR; CDCl3; 400 MHz
f1 (ppm)

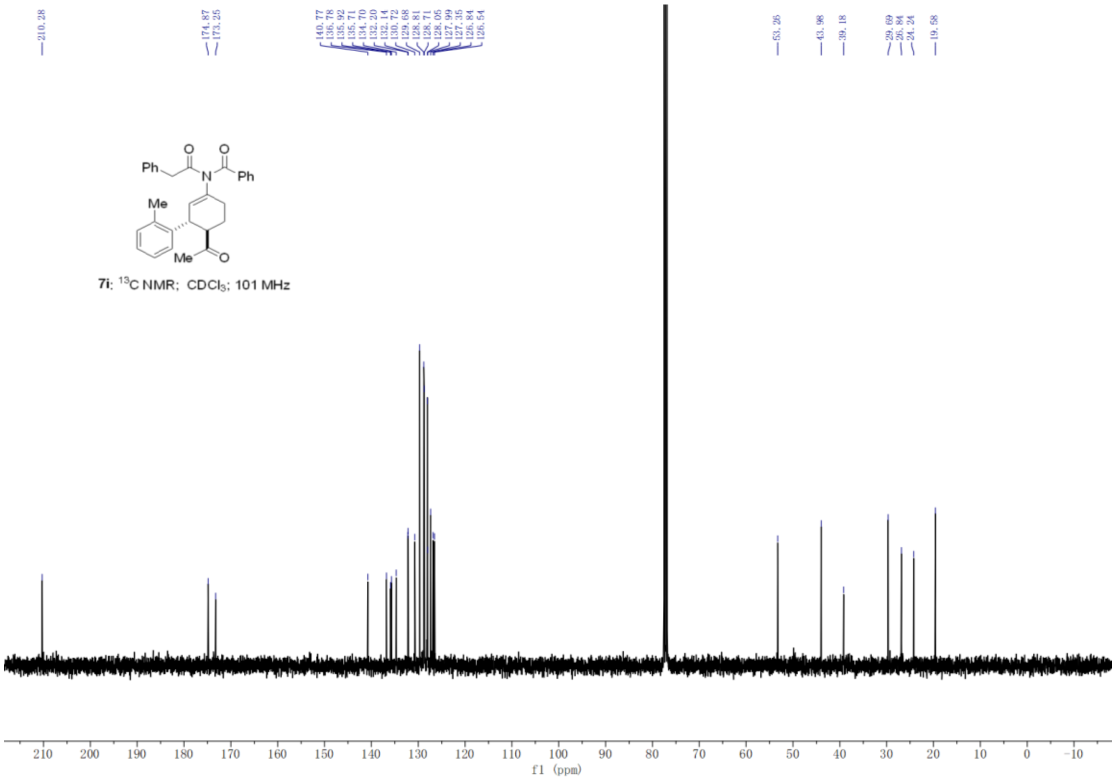
Ph
N
Ph
Me
Me
7i: 13C NMR; CDCl3; 101 MHz
f1 (ppm)

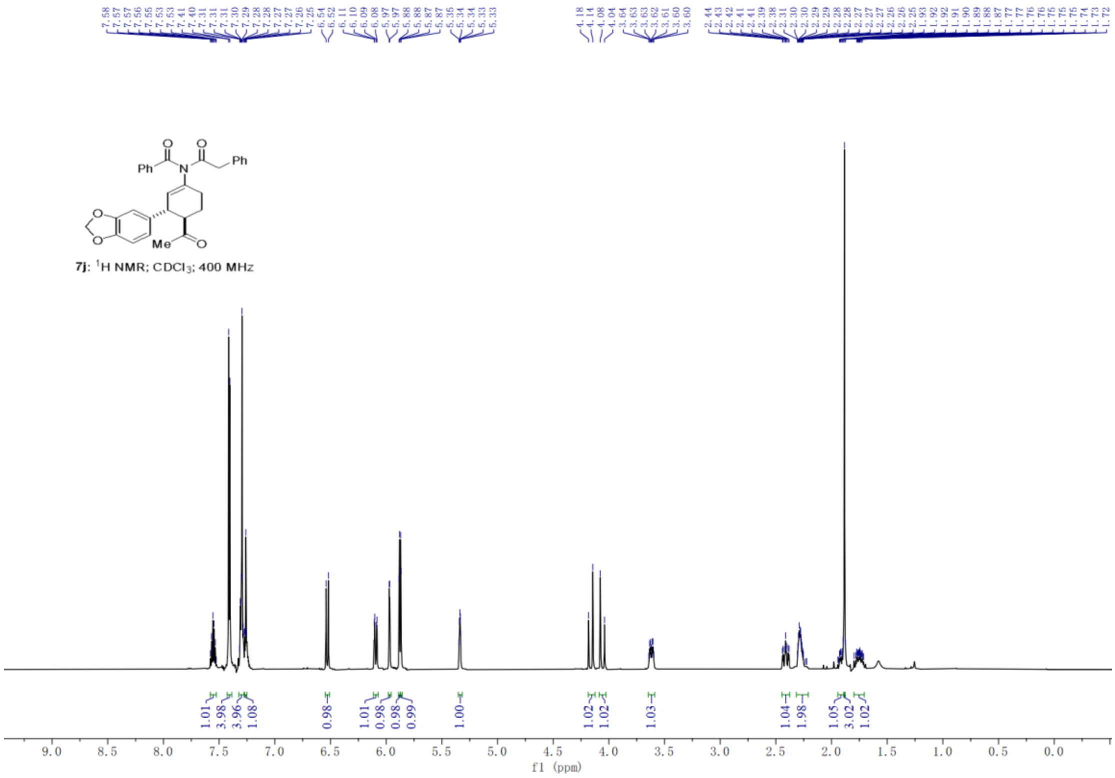
Ph
N
Ph
O
O
Me
7j: 1H NMR; CDCl3; 400 MHz
f1 (ppm)

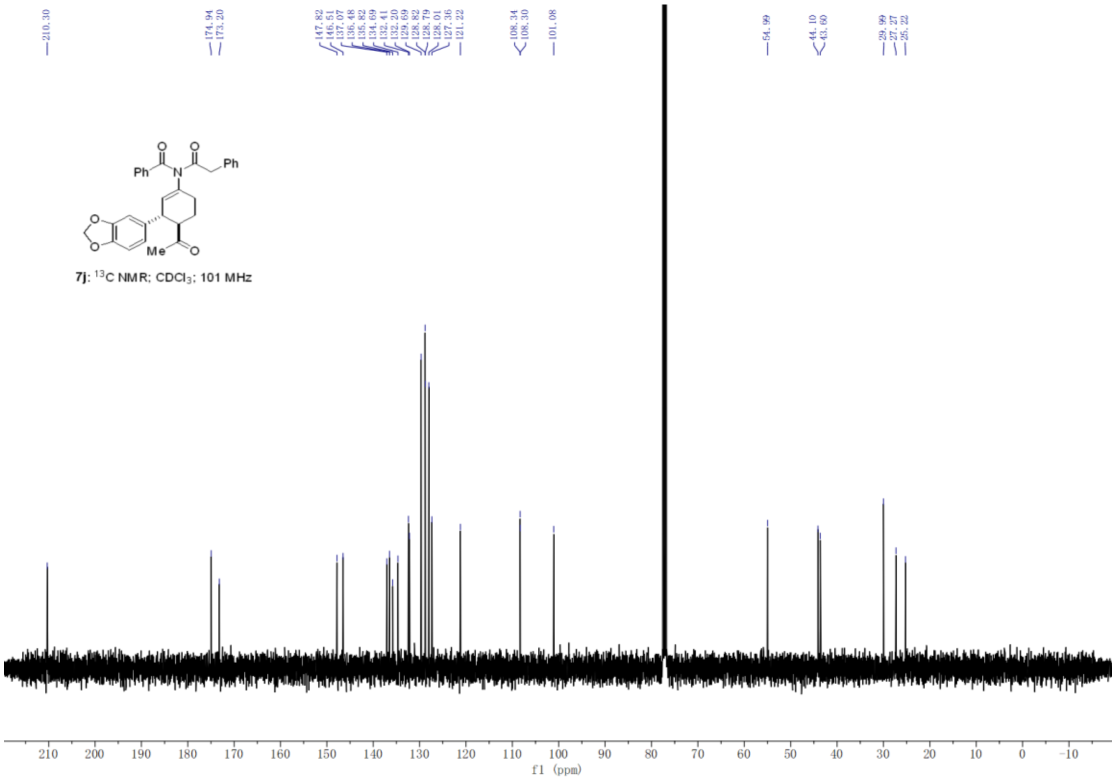
210.30
174.94
173.20
147.82
146.51
137.07
136.48
135.82
134.69
132.41
132.20
129.69
128.82
128.79
128.01
127.36
121.22
108.34
108.30
101.08
54.99
44.10
43.60
29.99
27.27
25.22
Ph
Ph
N
O
O
Me
7j: 13C NMR; CDCl3; 101 MHz
f1 (ppm)

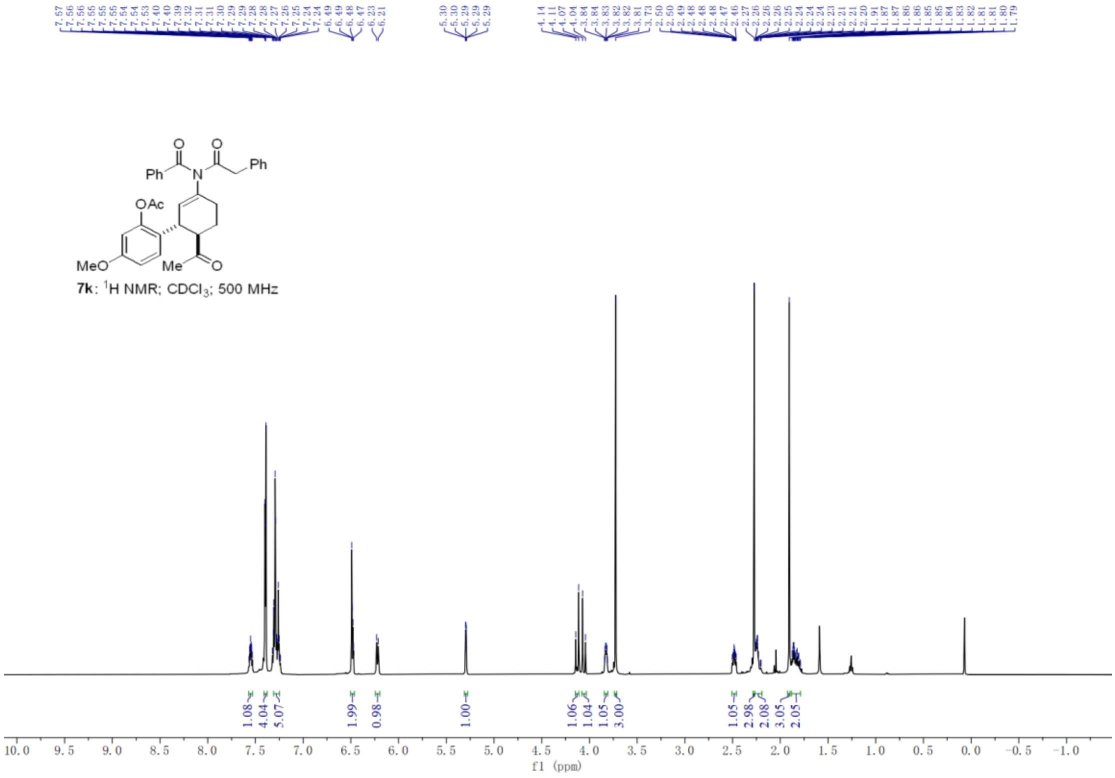
7.57
7.56
7.56
7.55
7.55
7.54
7.53
7.40
7.39
7.32
7.31
7.31
7.30
7.29
7.29
7.28
7.28
7.27
7.26
7.25
7.24
7.24
6.49
6.49
6.48
6.47
6.23
6.21
5.30
5.30
5.29
5.29
5.29
4.14
4.11
4.07
4.04
3.84
3.83
3.82
3.82
3.81
3.73
2.50
2.50
2.49
2.48
2.48
2.48
2.47
2.46
2.27
2.26
2.26
2.25
2.24
2.24
2.24
2.23
2.21
2.21
2.20
1.91
1.87
1.87
1.86
1.86
1.85
1.85
1.84
1.83
1.82
1.81
1.81
1.80
1.79
Ph
Ph
N
OAc
MeO
Me
O
7k: 1H NMR; CDCl3; 500 MHz
1.08
4.04
5.07
1.99
0.98
1.00
1.06
1.04
1.05
3.00
1.05
2.98
2.08
3.05
2.05
f1 (ppm)

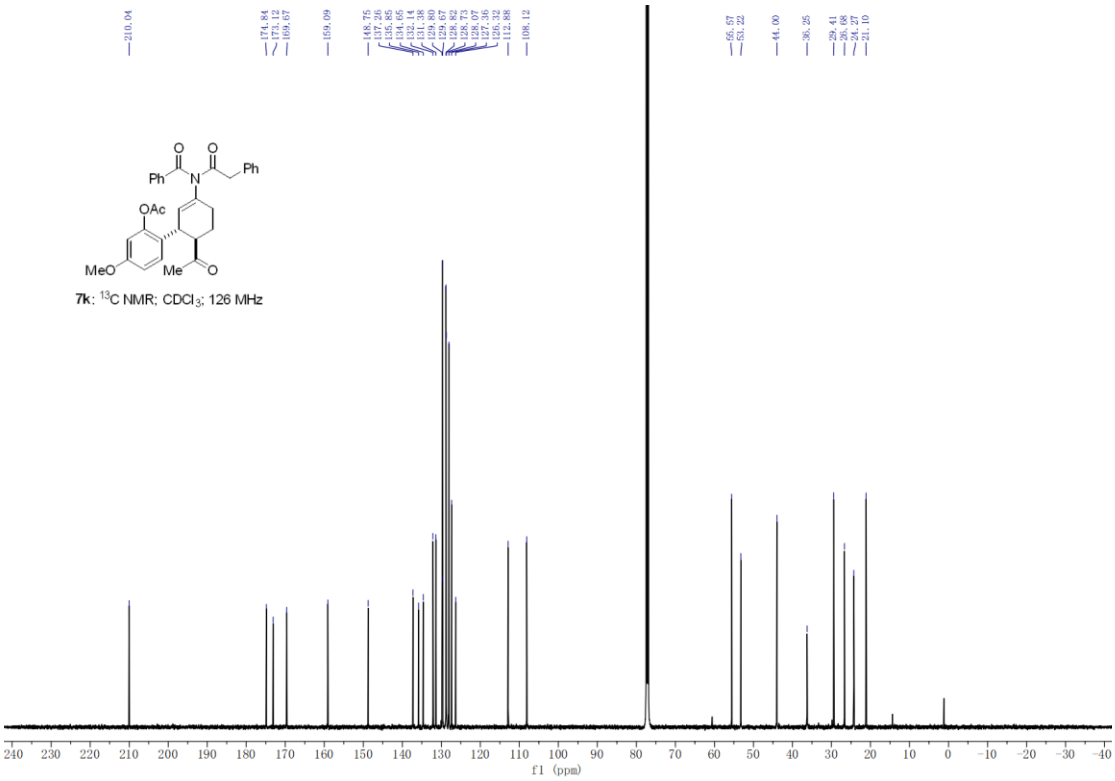
7k: 13C NMR; CDCl3; 126 MHz
f1 (ppm)

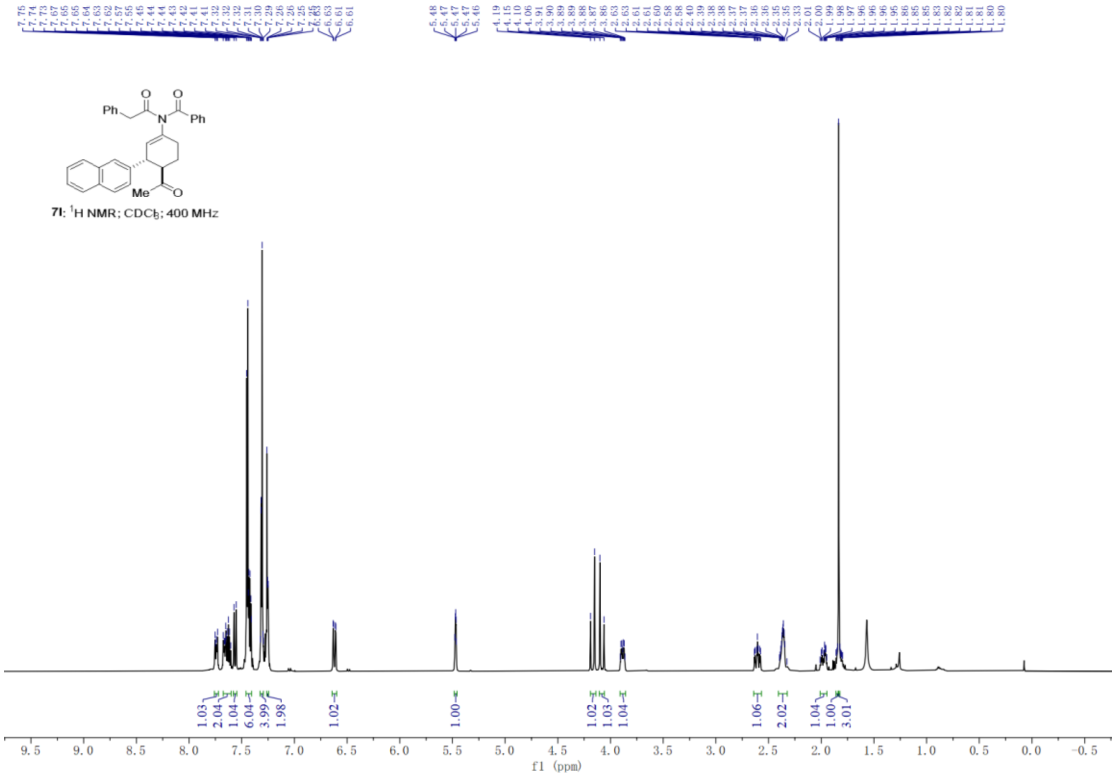
7l: 1H NMR; CDCl3; 400 MHz
f1 (ppm)

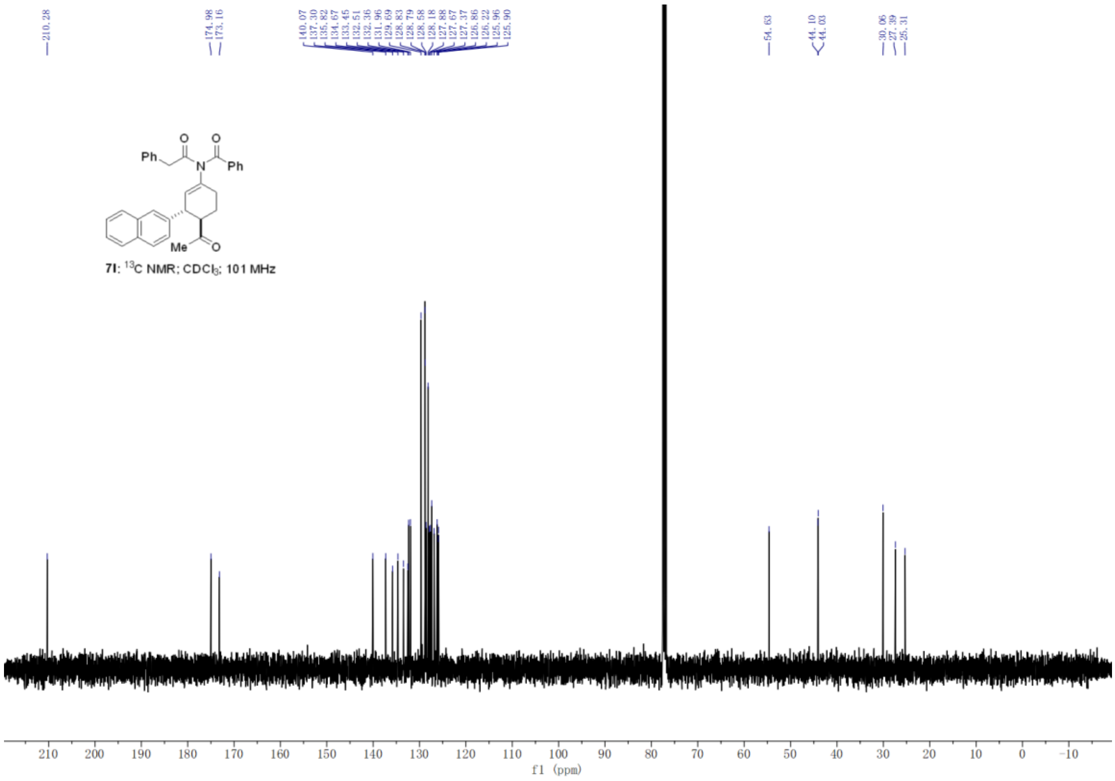

7l: ^{13}C NMR; $CDCl_3$; 101 MHz

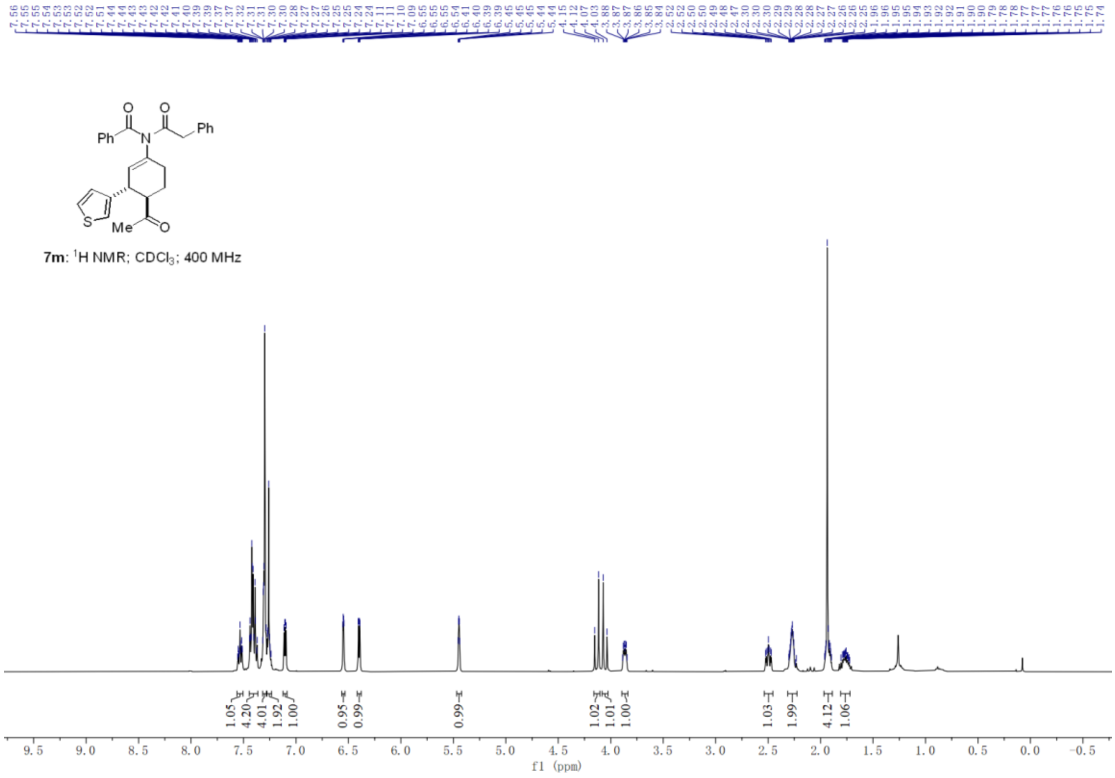

7m: ^1H NMR; $CDCl_3$; 400 MHz

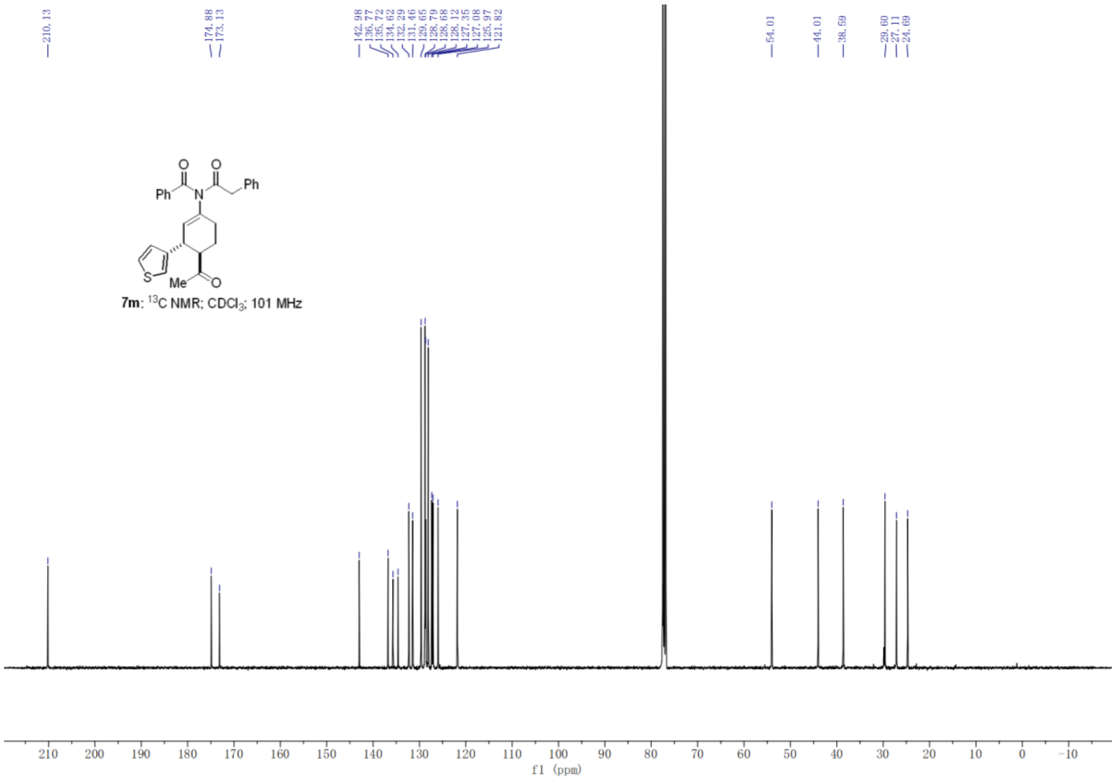

7m: ^{13}C NMR; $CDCl_3$; 101 MHz

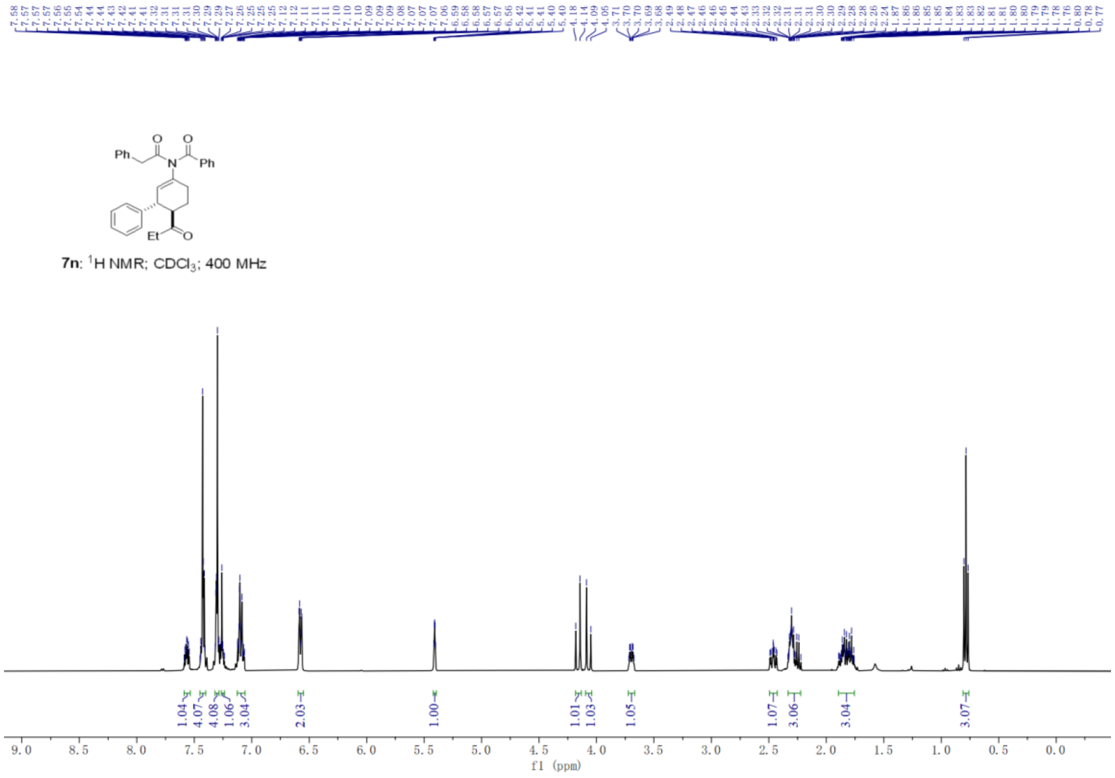

7n: ^{1}H NMR; $CDCl_3$; 400 MHz

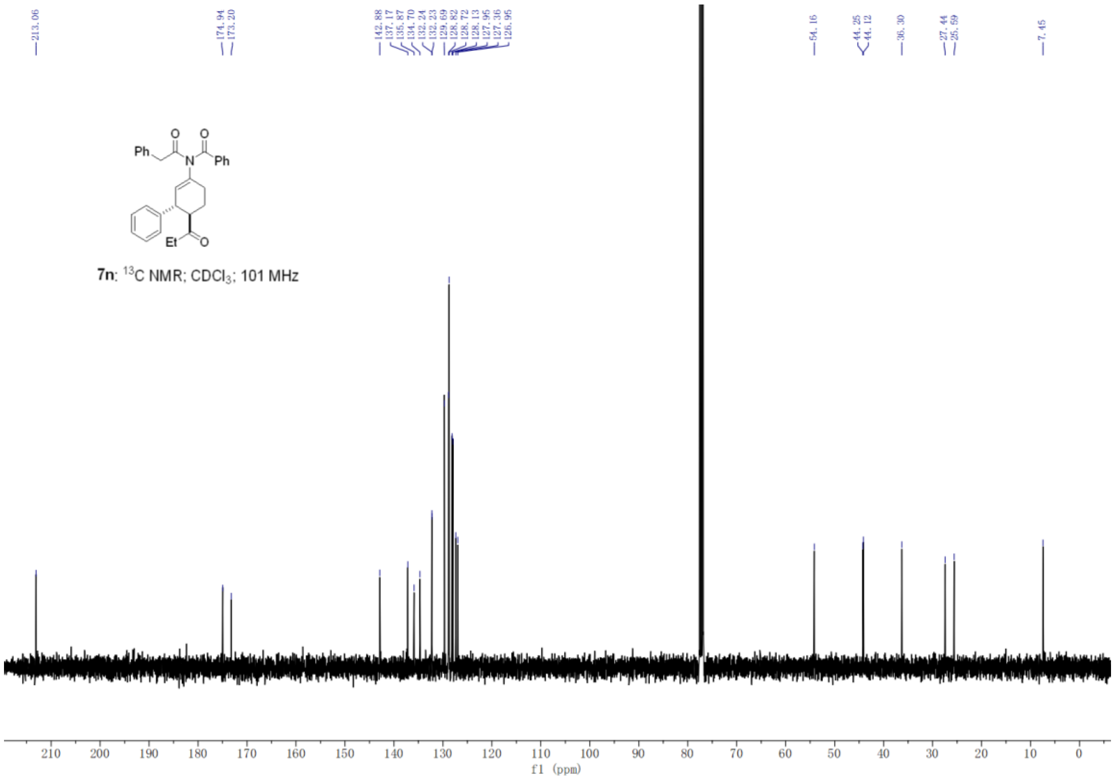

7n: ^{13}C NMR; $CDCl_3$; 101 MHz

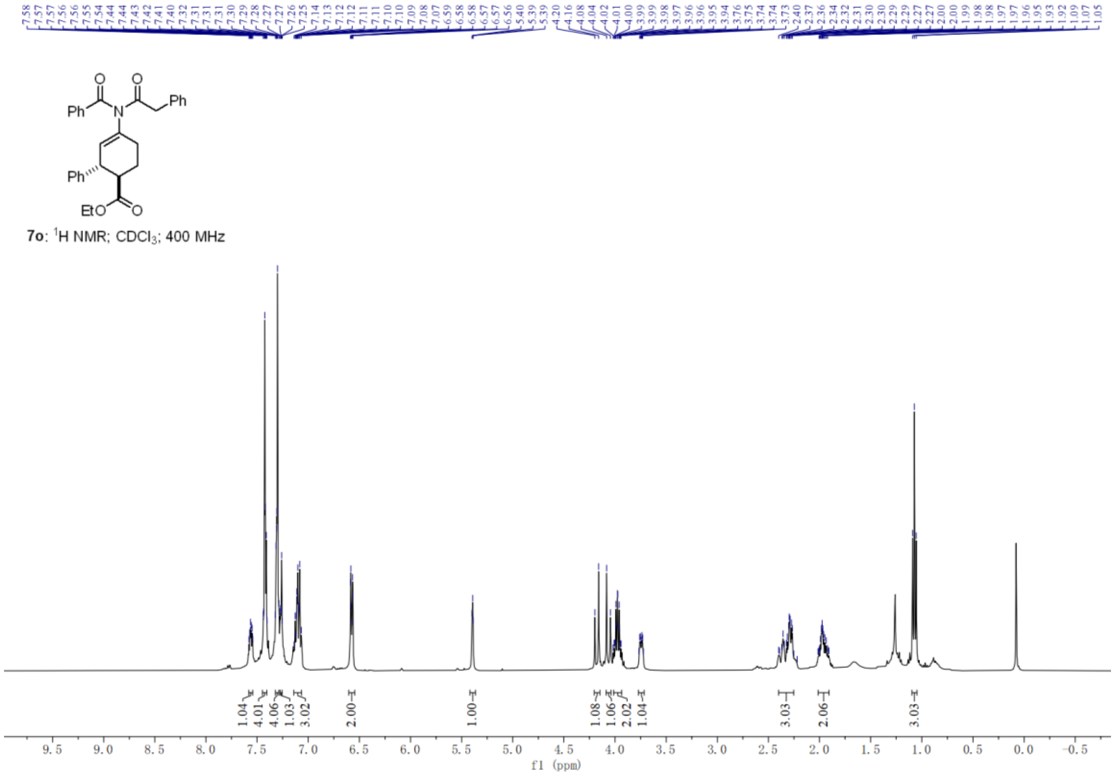

7o: ^{1}H NMR; $CDCl_3$; 400 MHz

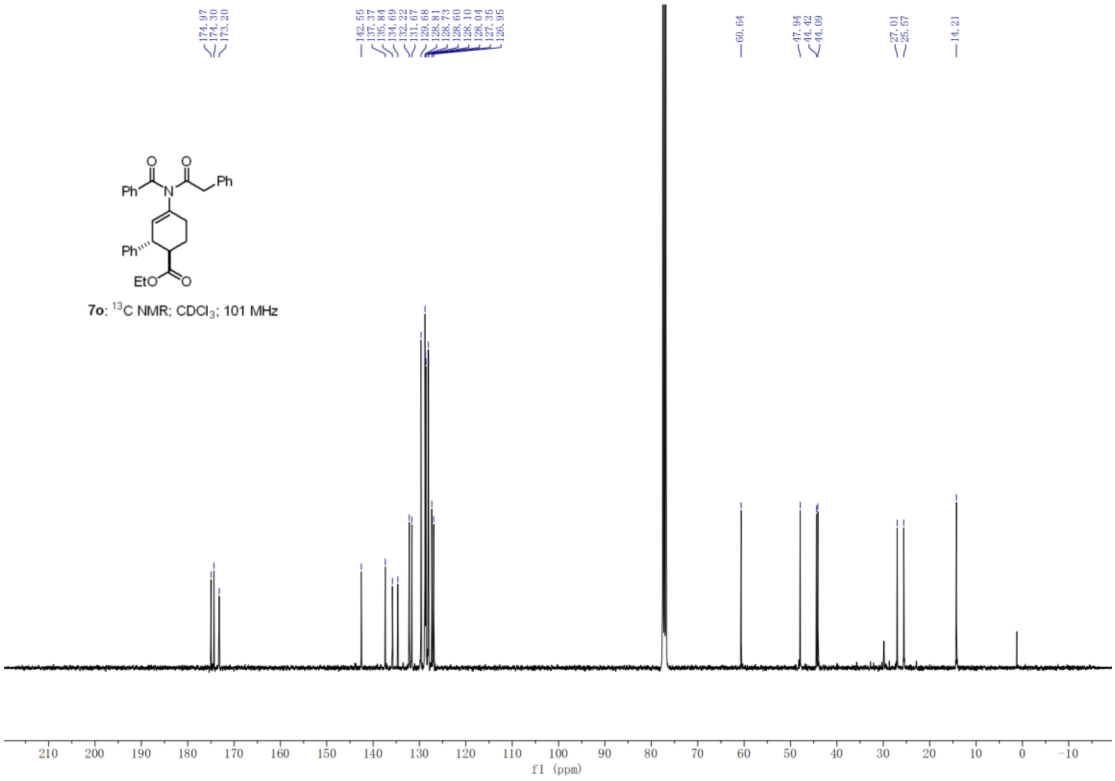

7o: ^{13}C NMR; $CDCl_3$; 101 MHz

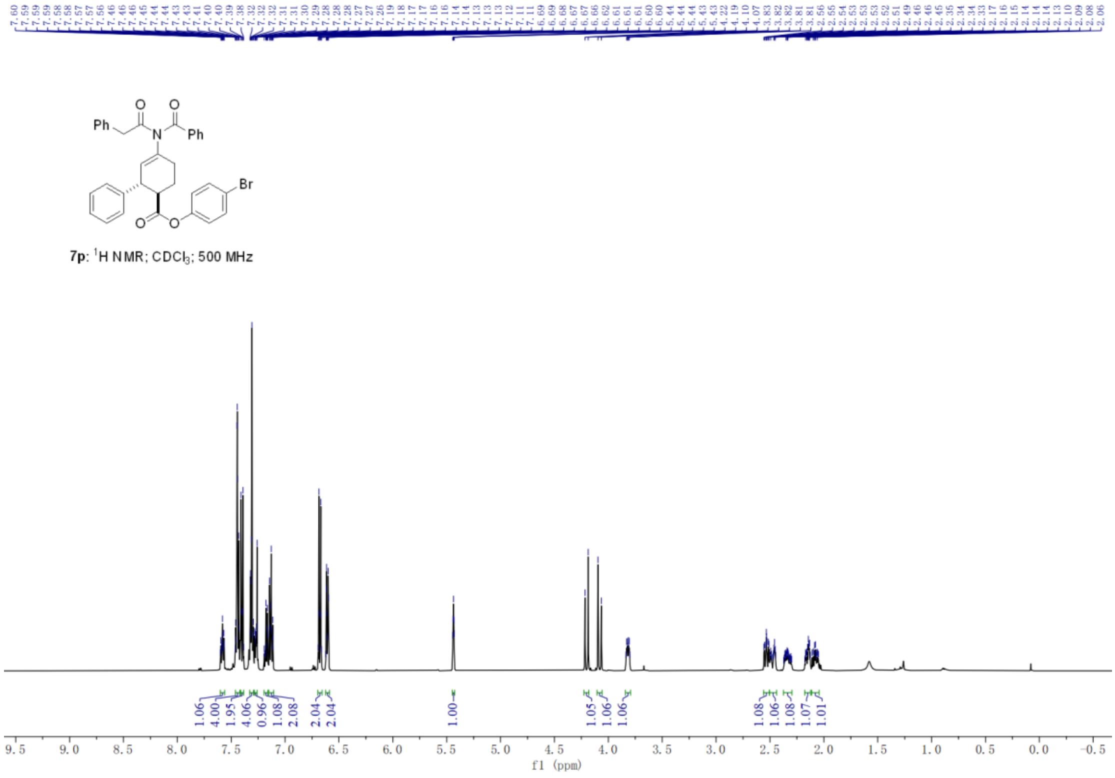

7p: ^{1}H NMR; $CDCl_3$; 500 MHz

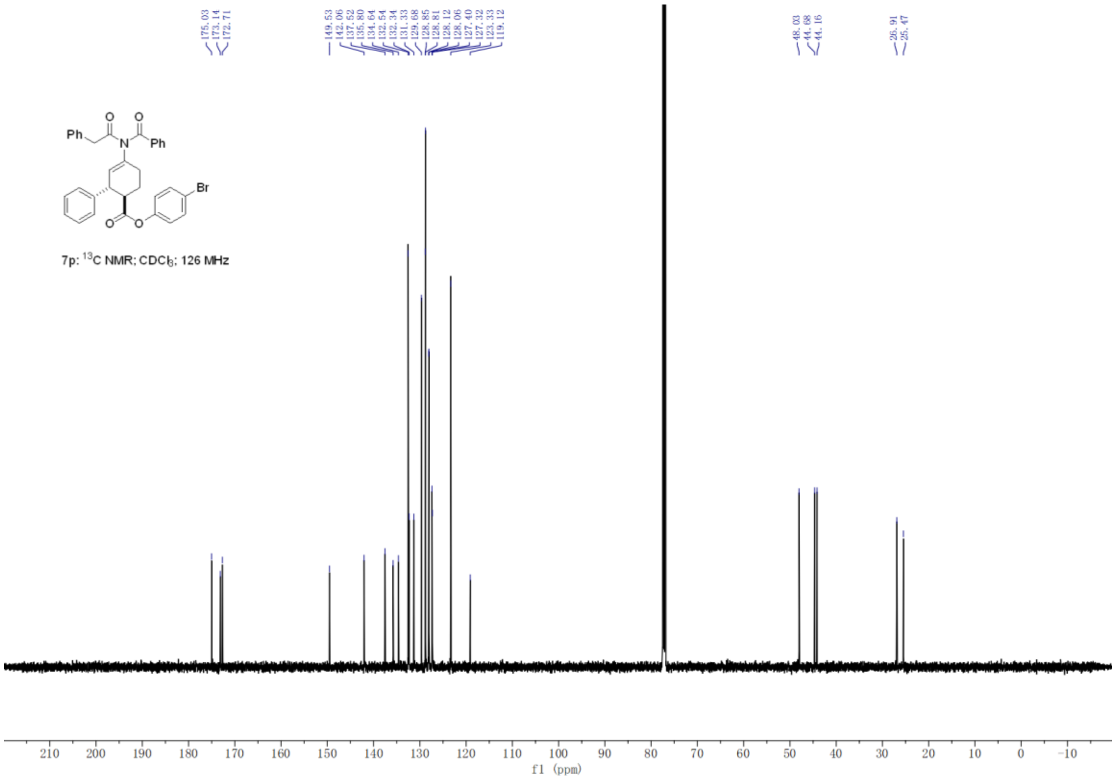

7p: ^{13}C NMR; $CDCl_3$; 126 MHz

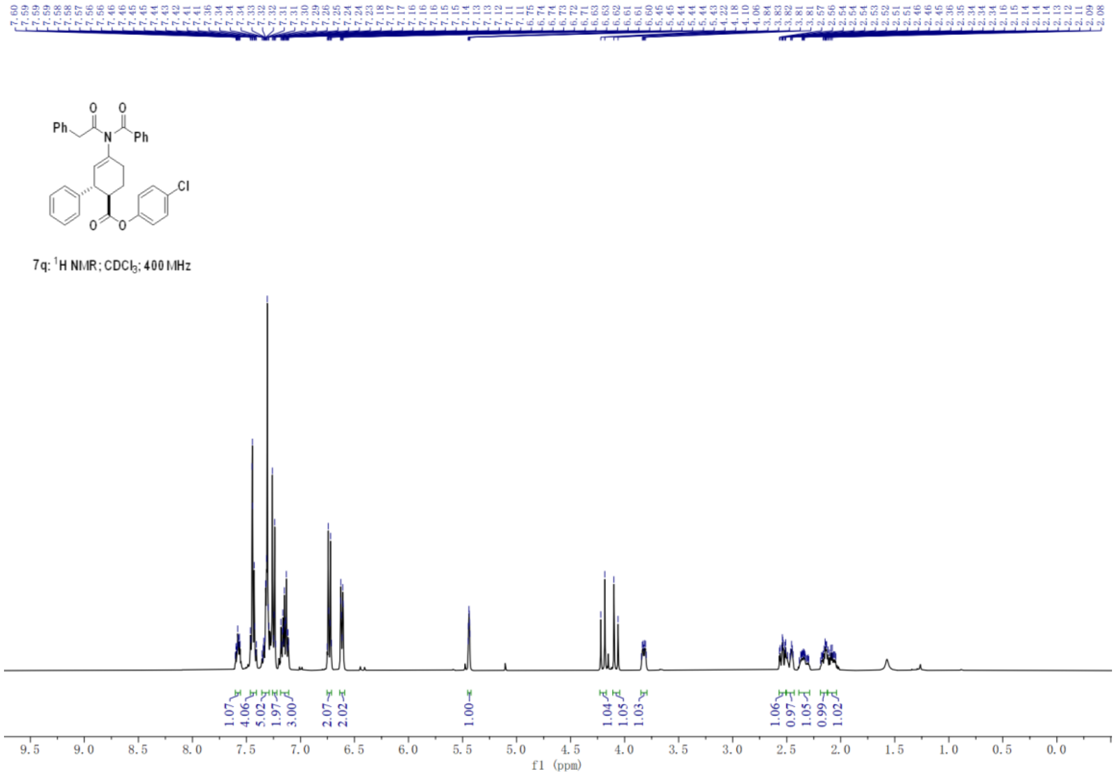

7q: ^{1}H NMR; $CDCl_3$; 400 MHz

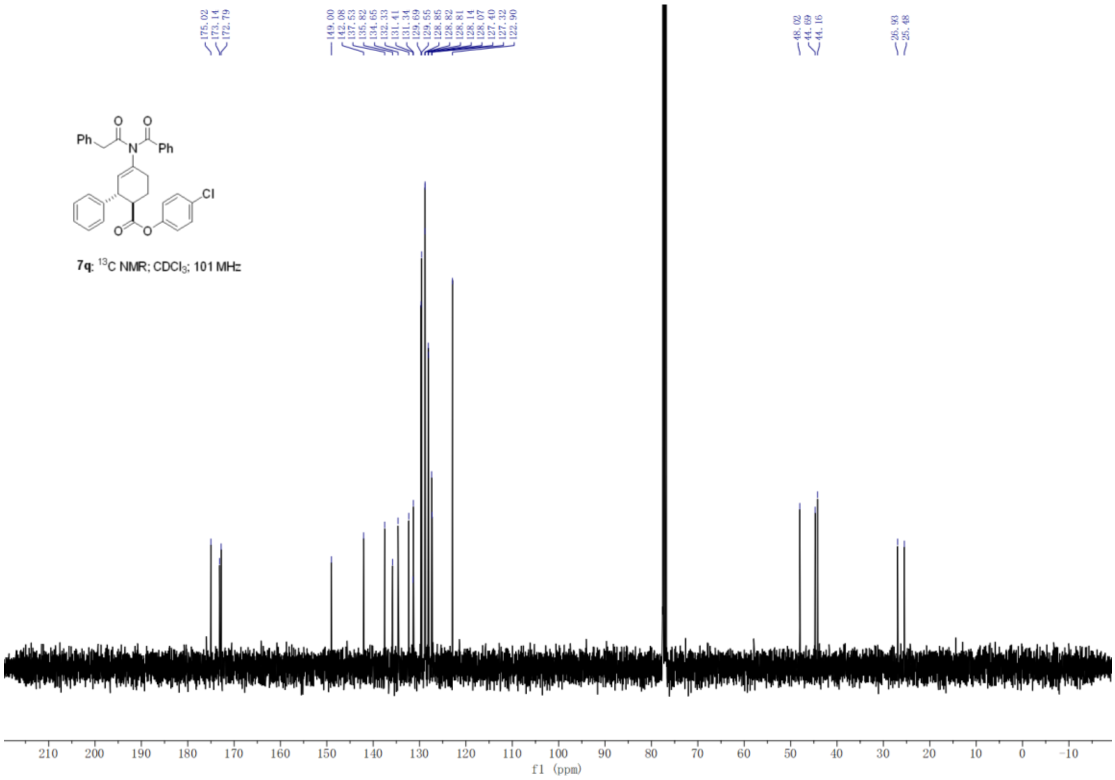
175.02
173.14
172.79
149.00
142.08
137.53
135.82
134.65
132.33
131.41
131.34
129.69
129.55
128.85
128.82
128.81
128.14
128.07
127.40
127.32
122.90
48.02
44.69
44.16
26.93
25.48
Ph
N
Ph
Cl
O
7q: 13C NMR; CDCl3; 101 MHz
f1 (ppm)

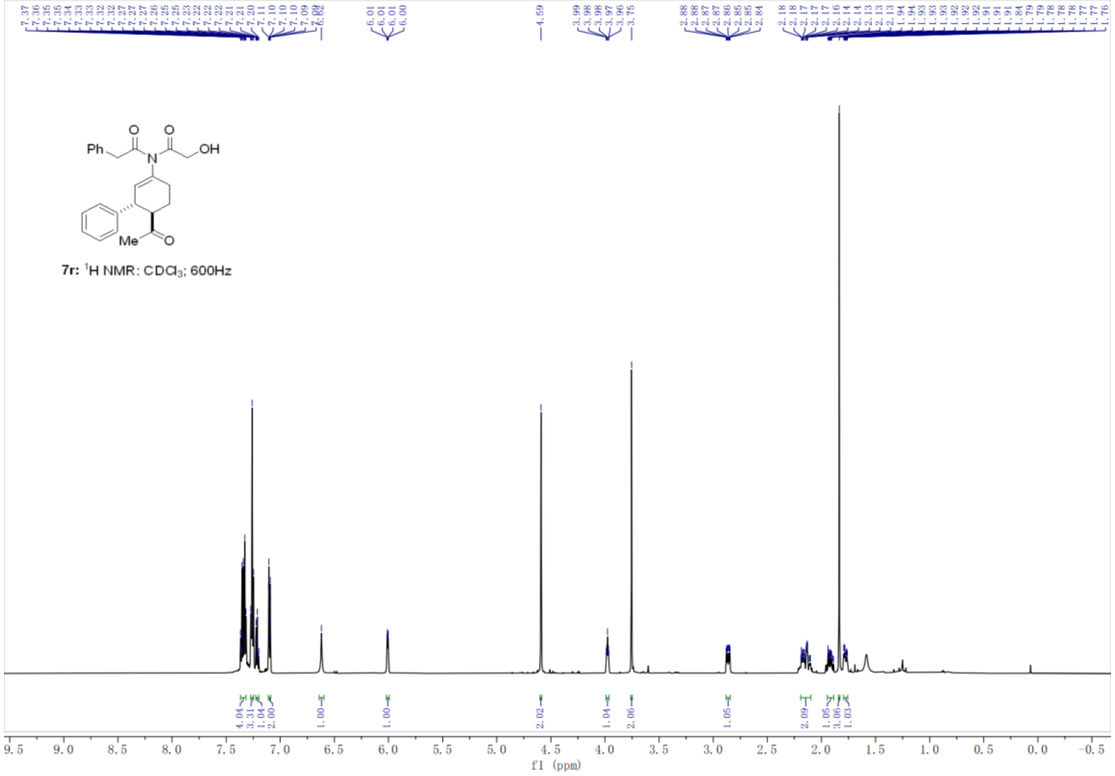
Ph
N
OH
Me
O
7r: 1H NMR: CDCl3; 600Hz
f1 (ppm)

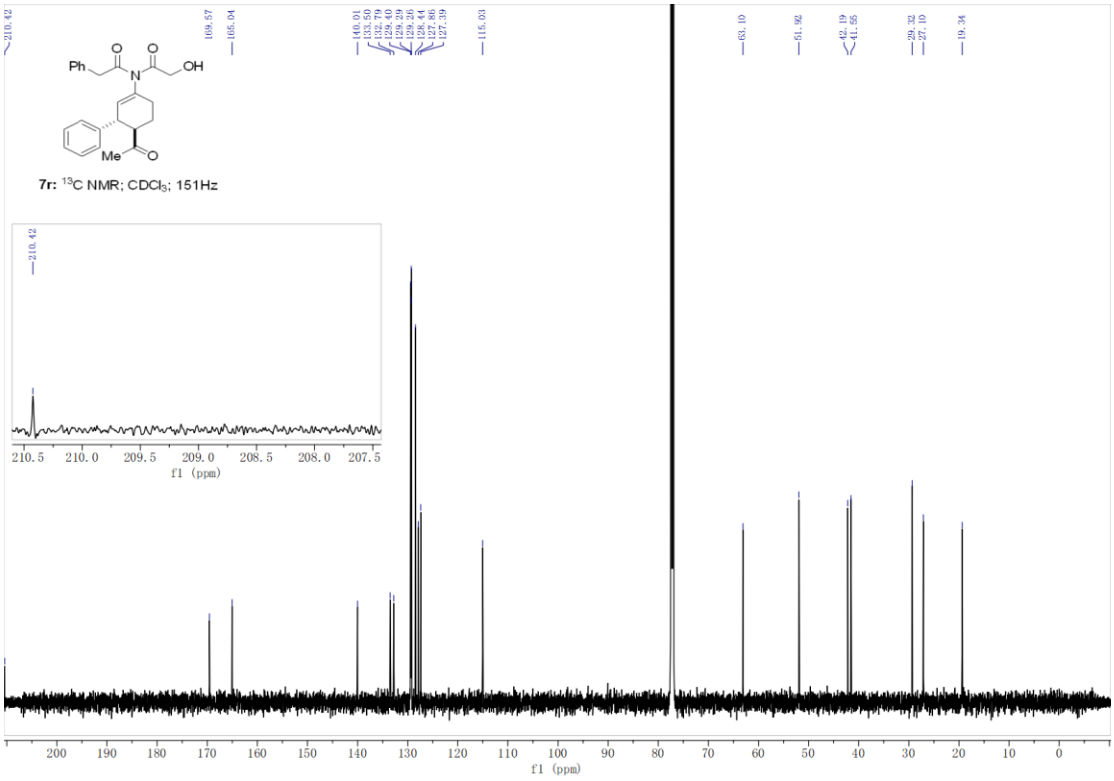
7r: ^{13}C NMR; CDCl$_3$; 151Hz
210.42
169.57
165.04
140.01
133.50
132.79
129.40
129.29
129.26
128.44
127.86
127.39
115.03
63.10
51.92
42.19
41.55
29.32
27.10
19.34
f1 (ppm)

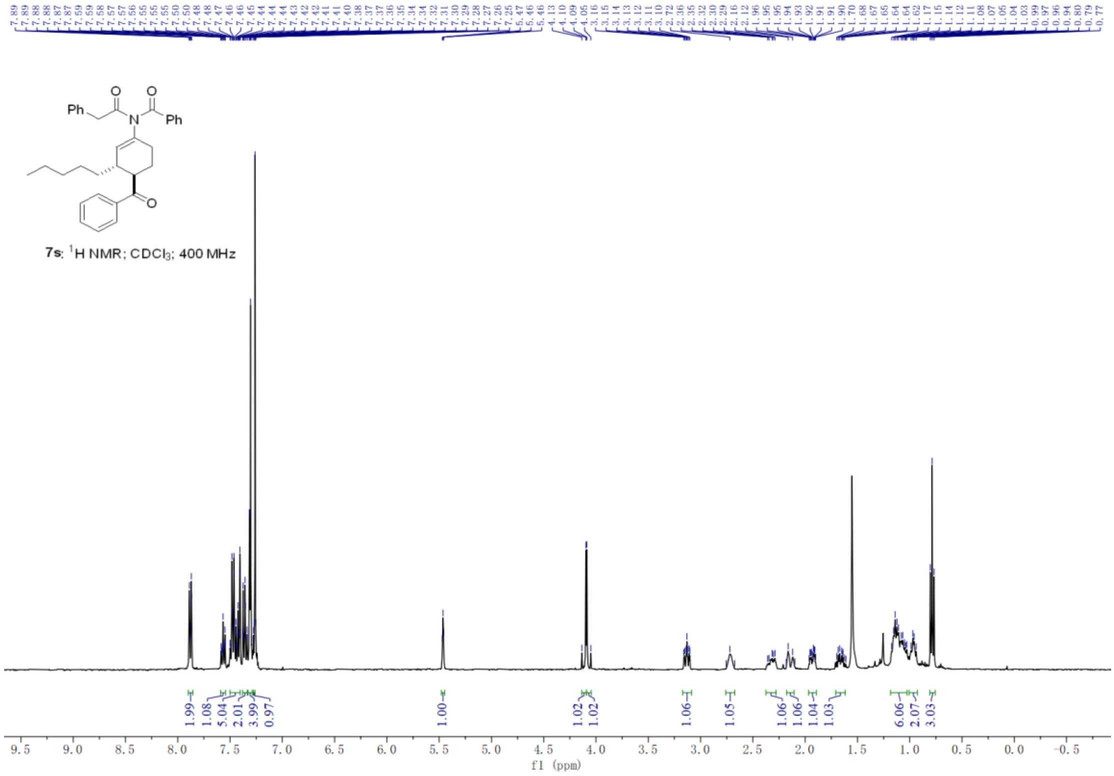
7s: ^{1}H NMR; CDCl$_3$; 400 MHz
1.99
1.08
5.04
2.01
3.99
0.97
1.00
1.02
1.02
1.06
1.05
1.06
1.06
1.04
1.03
6.06
2.07
3.03
f1 (ppm)

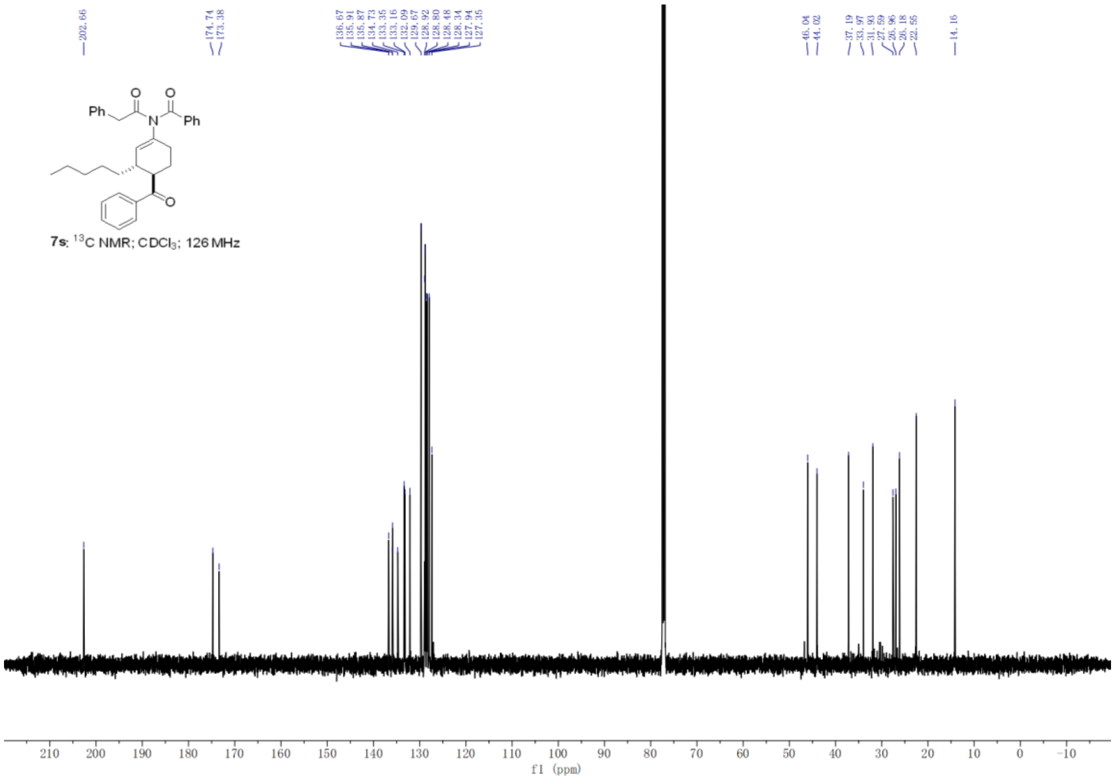

7s; ^{13}C NMR; $CDCl_3$; 126 MHz

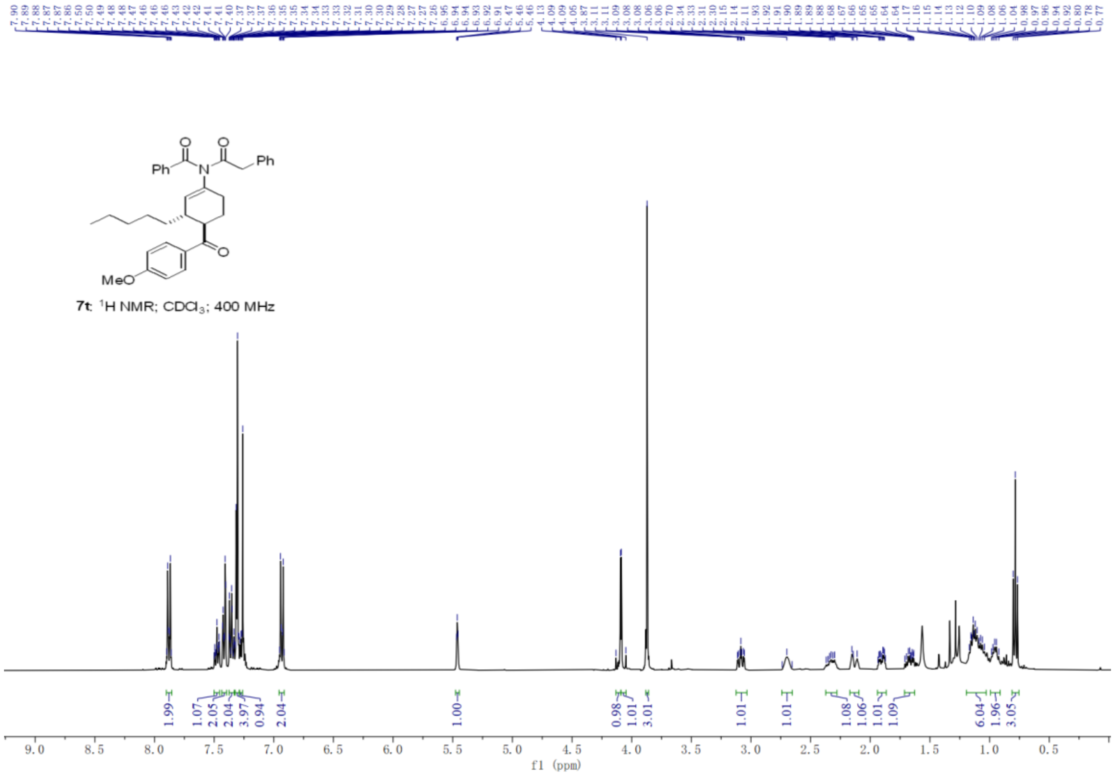

7t: ^{1}H NMR; $CDCl_3$; 400 MHz

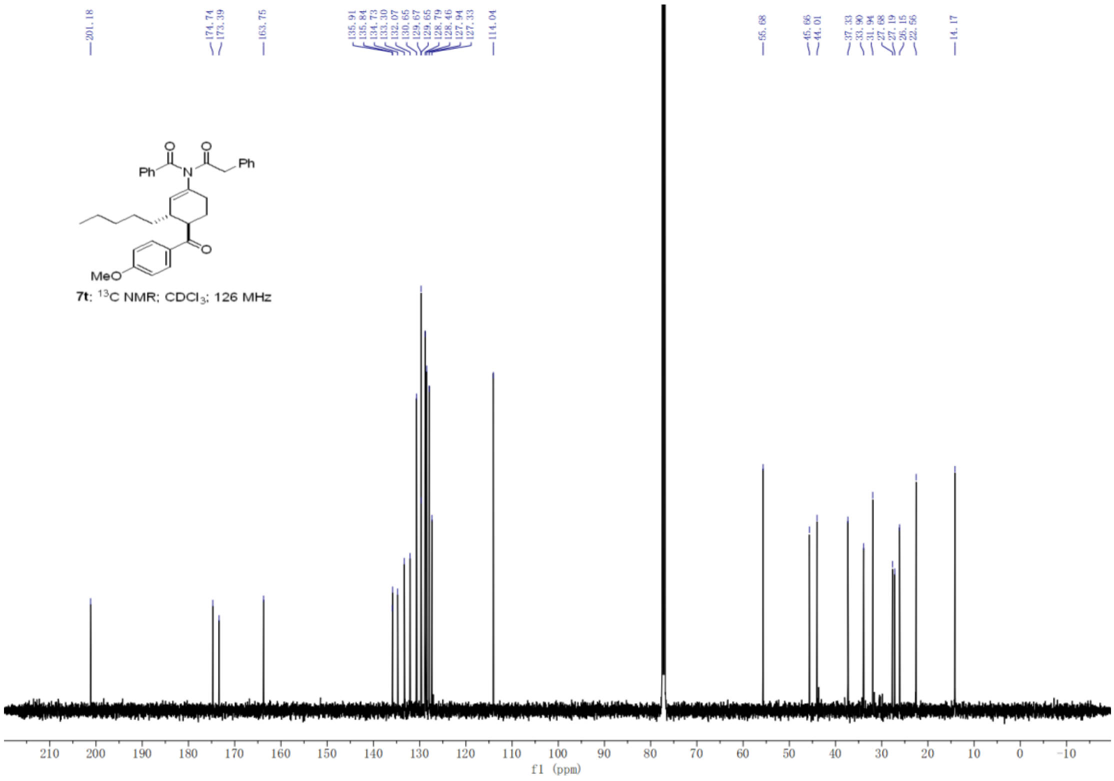
7t: 13C NMR; CDCl3; 126 MHz
f1 (ppm)

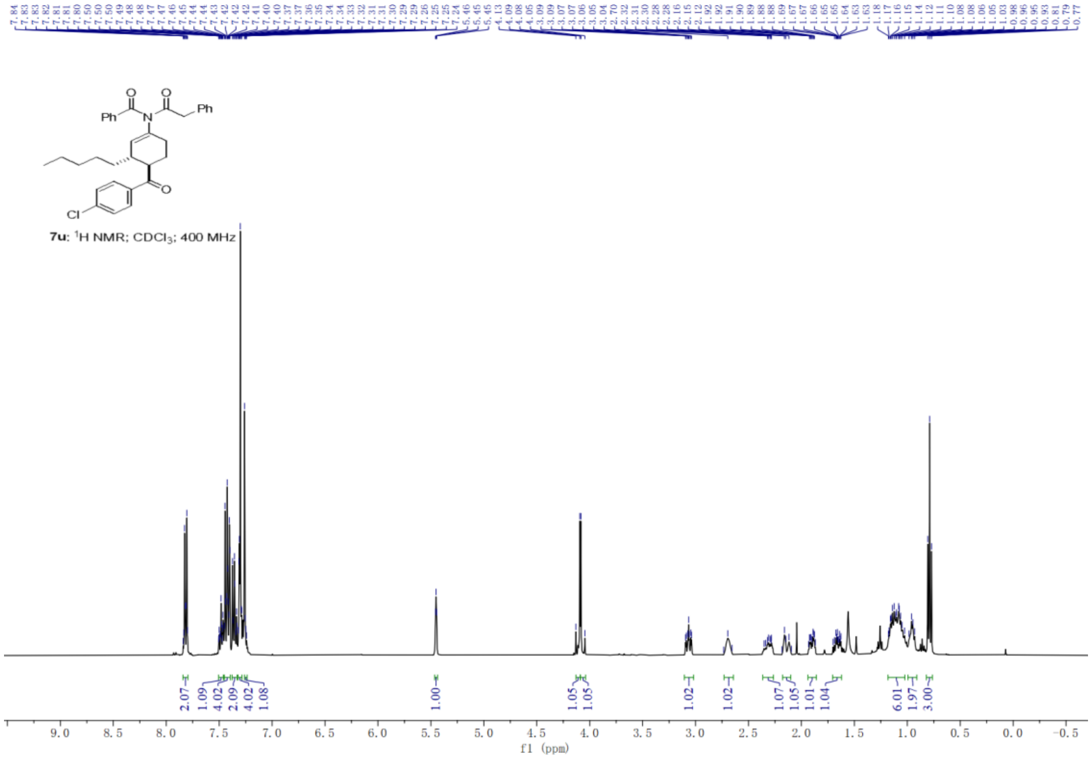
7u: 1H NMR; CDCl3; 400 MHz
f1 (ppm)

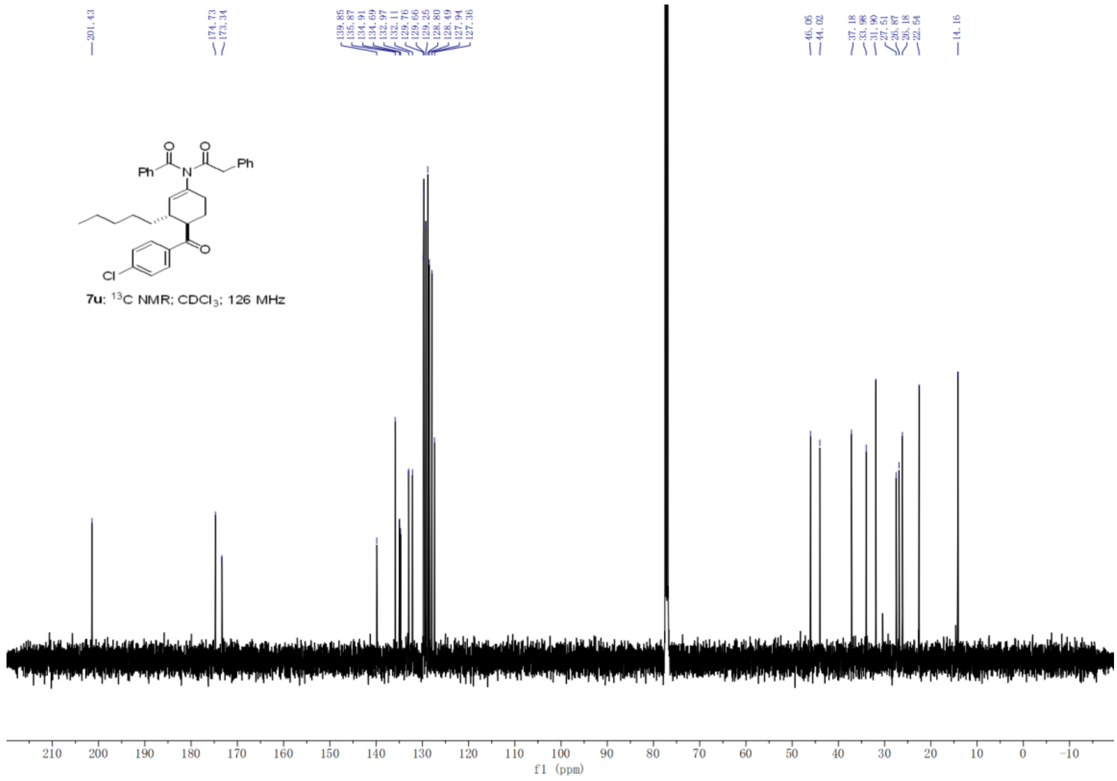

7u: ^{13}C NMR; $CDCl_3$; 126 MHz

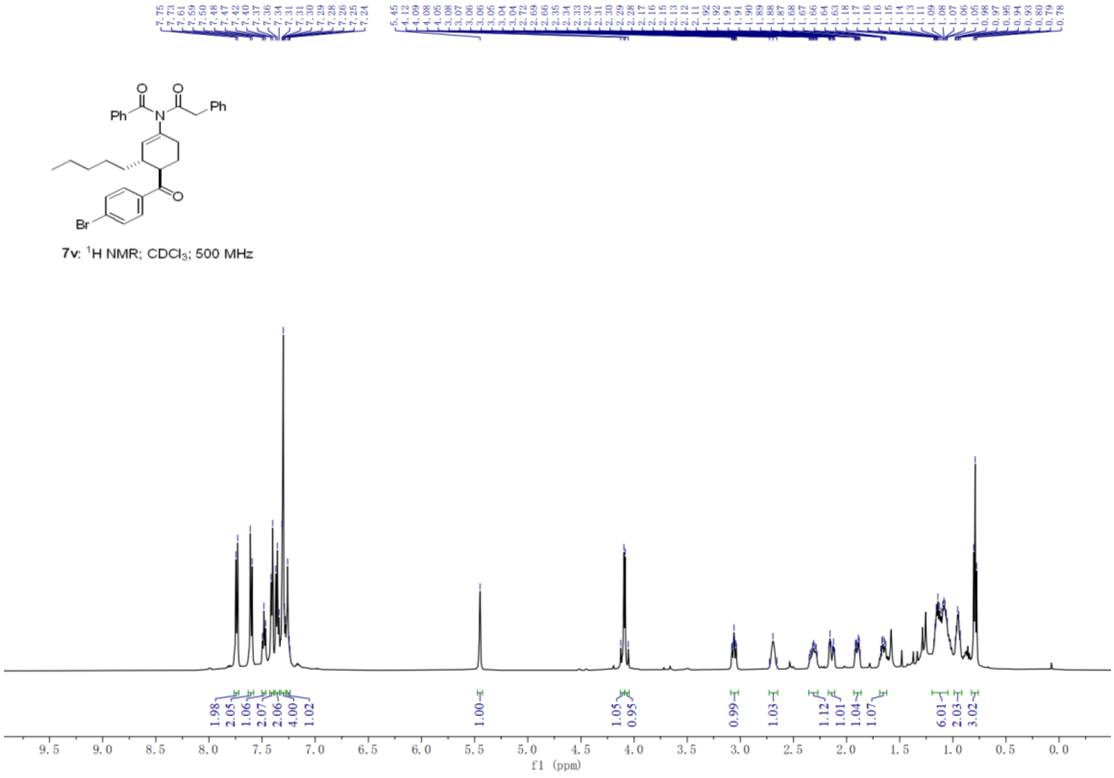

7v: ^{1}H NMR; $CDCl_3$; 500 MHz

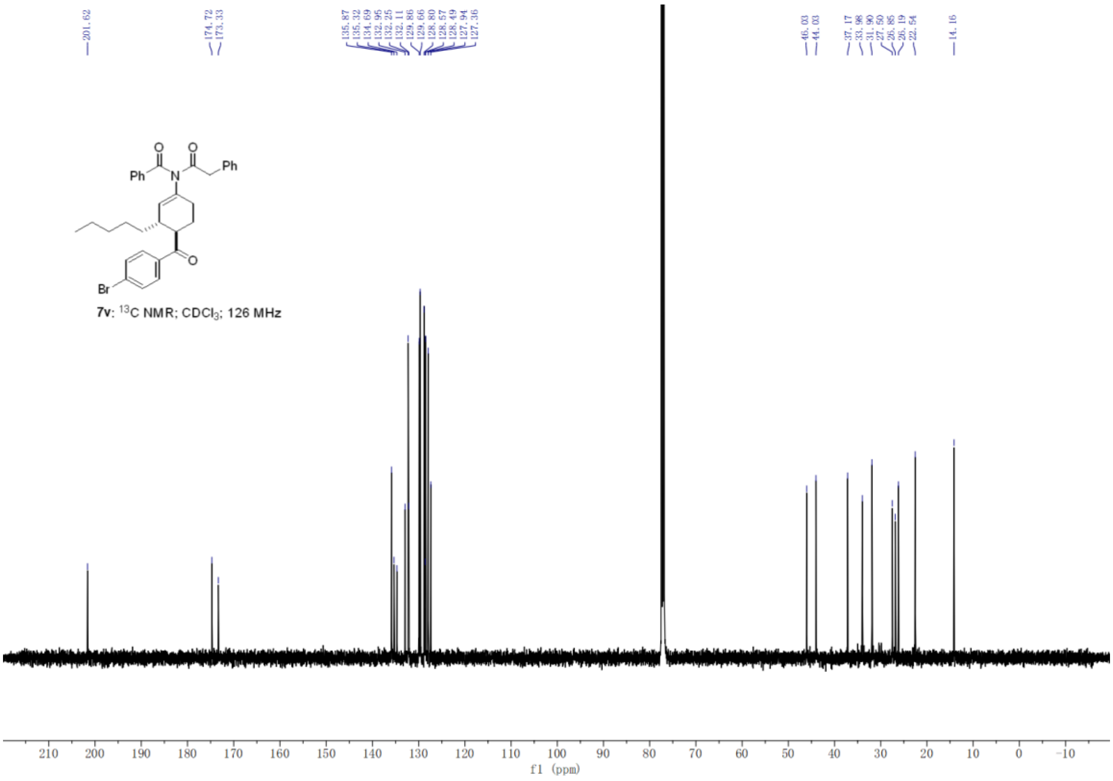
201.62
174.72
173.33
135.87
135.32
134.69
132.95
132.25
132.11
129.86
129.66
128.80
128.57
128.49
127.94
127.36
46.03
44.03
37.17
33.98
31.90
27.50
26.85
26.19
22.54
14.16
Ph
N
O
O
Ph
Br
O
7v: 13C NMR; CDCl3; 126 MHz
210 200 190 180 170 160 150 140 130 120 110 100 90 80 70 60 50 40 30 20 10 0 -10
f1 (ppm)

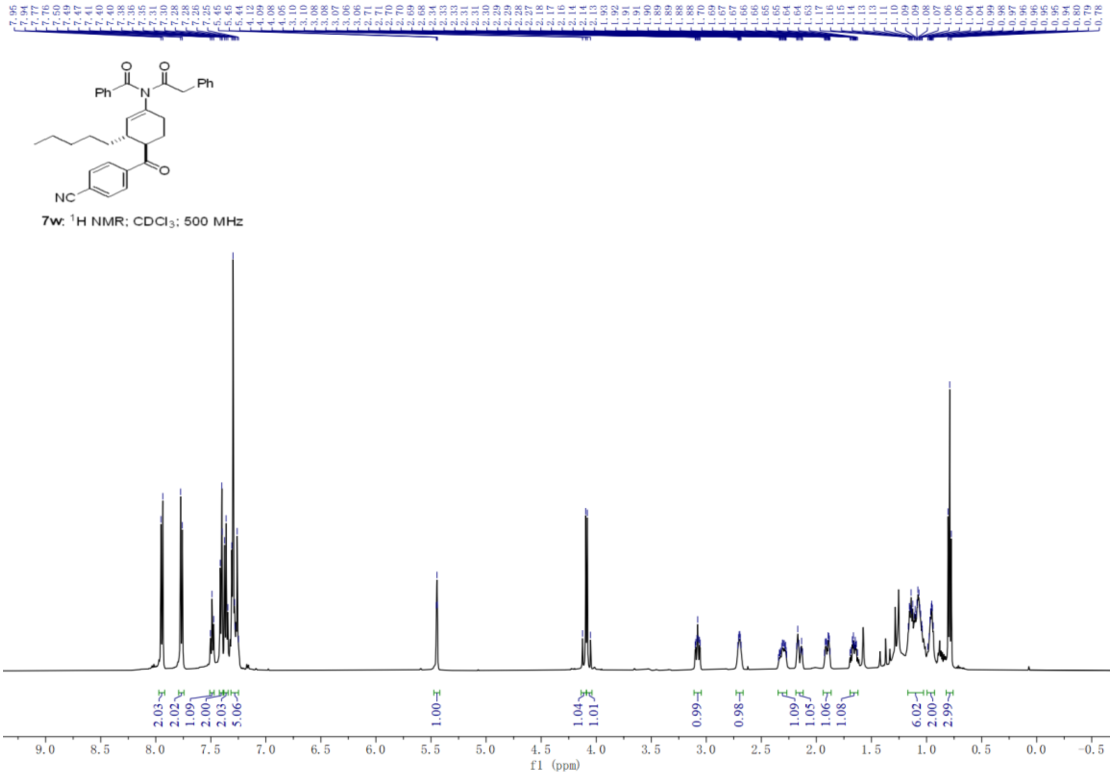
7.95
7.94
7.77
7.76
7.50
7.49
7.47
7.41
7.40
7.40
7.38
7.36
7.35
7.31
7.30
7.28
7.28
7.26
7.25
5.45
5.45
5.44
4.12
4.09
4.08
4.05
3.10
3.10
3.08
3.08
3.07
3.06
3.06
2.71
2.71
2.70
2.70
2.69
2.68
2.34
2.33
2.33
2.31
2.31
2.30
2.29
2.29
2.28
2.27
2.18
2.17
2.16
2.14
2.14
2.13
1.93
1.92
1.91
1.91
1.90
1.89
1.89
1.88
1.88
1.70
1.69
1.67
1.67
1.66
1.66
1.65
1.65
1.64
1.64
1.63
1.17
1.16
1.15
1.14
1.13
1.13
1.11
1.10
1.09
1.09
1.08
1.07
1.06
1.05
1.04
1.04
0.99
0.98
0.97
0.96
0.96
0.95
0.95
0.94
0.80
0.79
0.78
Ph
N
O
O
Ph
NC
O
7w: 1H NMR; CDCl3; 500 MHz
2.03
2.02
1.09
2.00
2.03
5.06
1.00
1.04
1.01
0.99
0.98
1.09
1.05
1.06
1.08
6.02
2.00
2.99
9.0 8.5 8.0 7.5 7.0 6.5 6.0 5.5 5.0 4.5 4.0 3.5 3.0 2.5 2.0 1.5 1.0 0.5 0.0 -0.5
f1 (ppm)

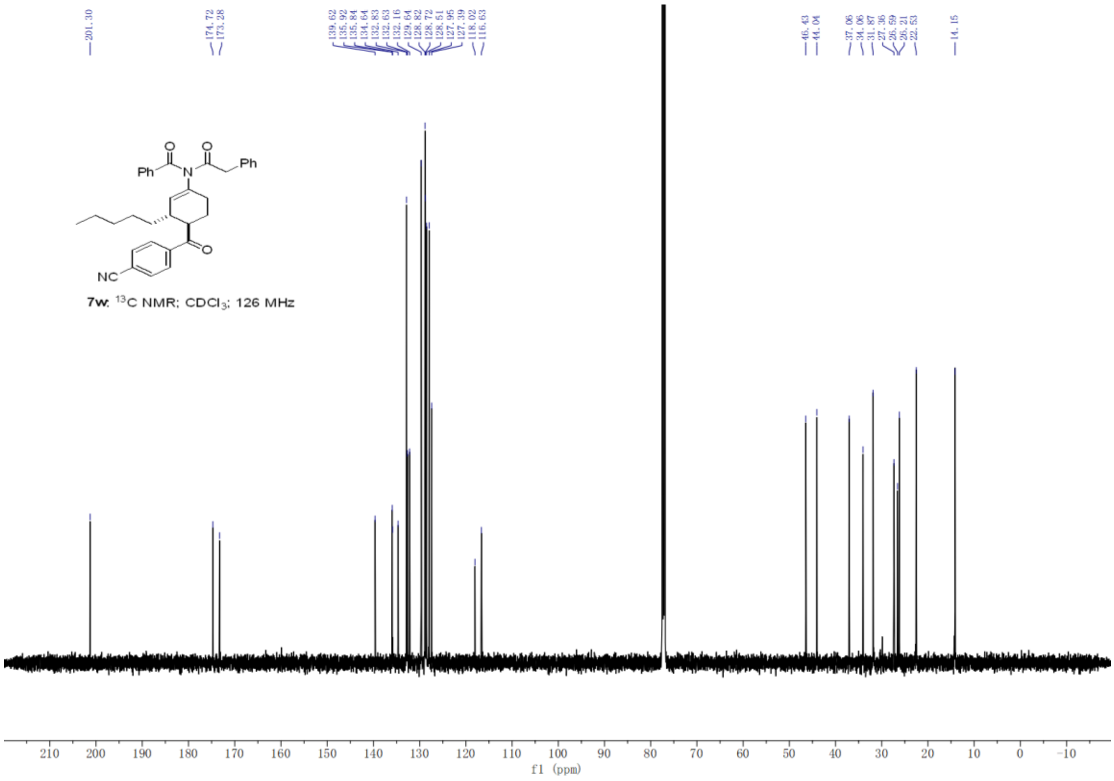

7w: ^{13}C NMR; $CDCl_3$; 126 MHz

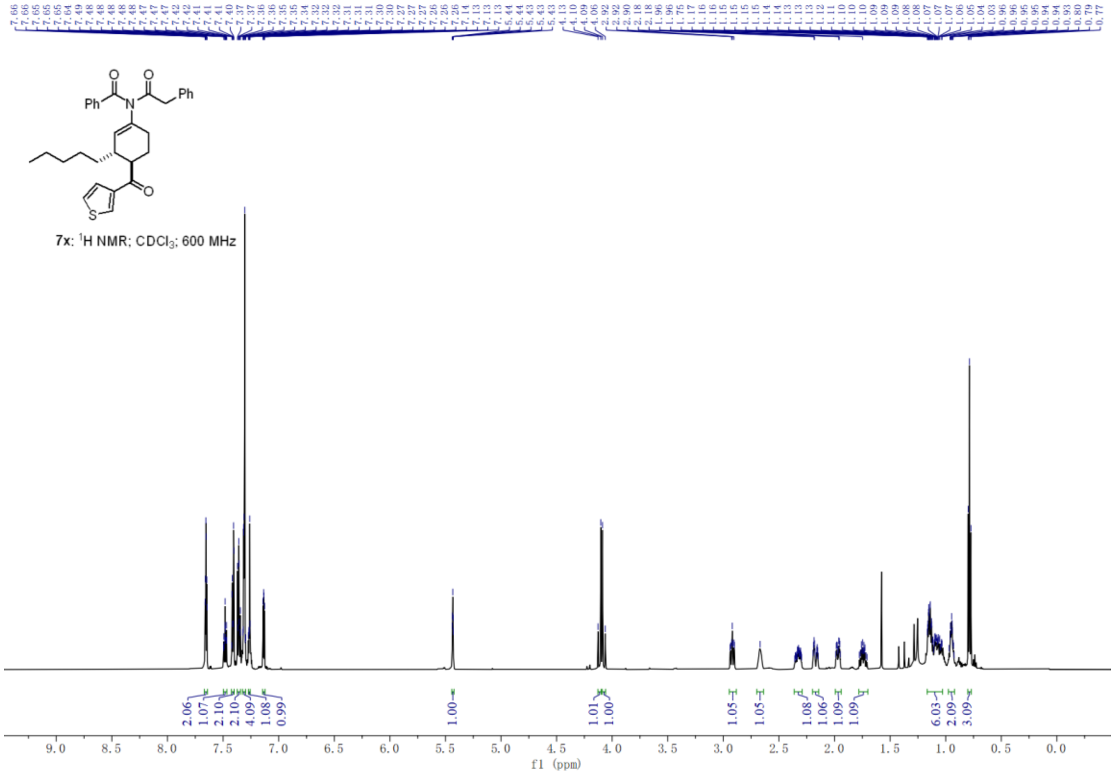

7x: ^{1}H NMR; $CDCl_3$; 600 MHz

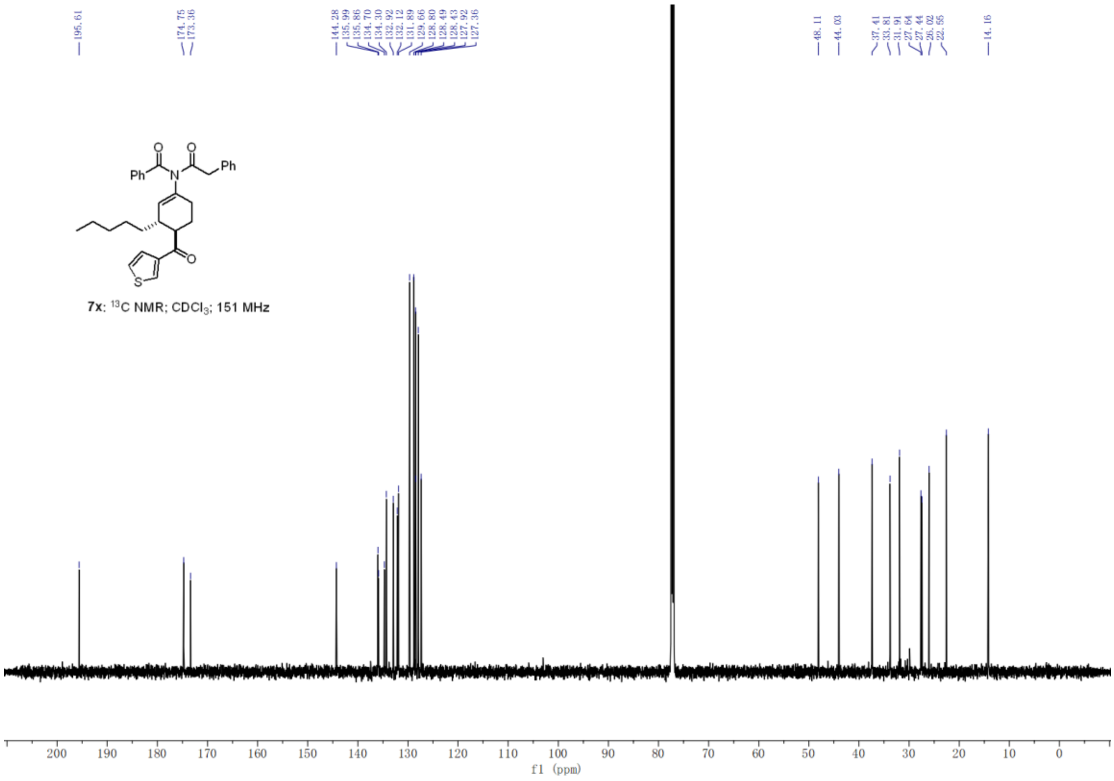
7x; ^{13}C NMR; CDCl$_3$; 151 MHz
f1 (ppm)

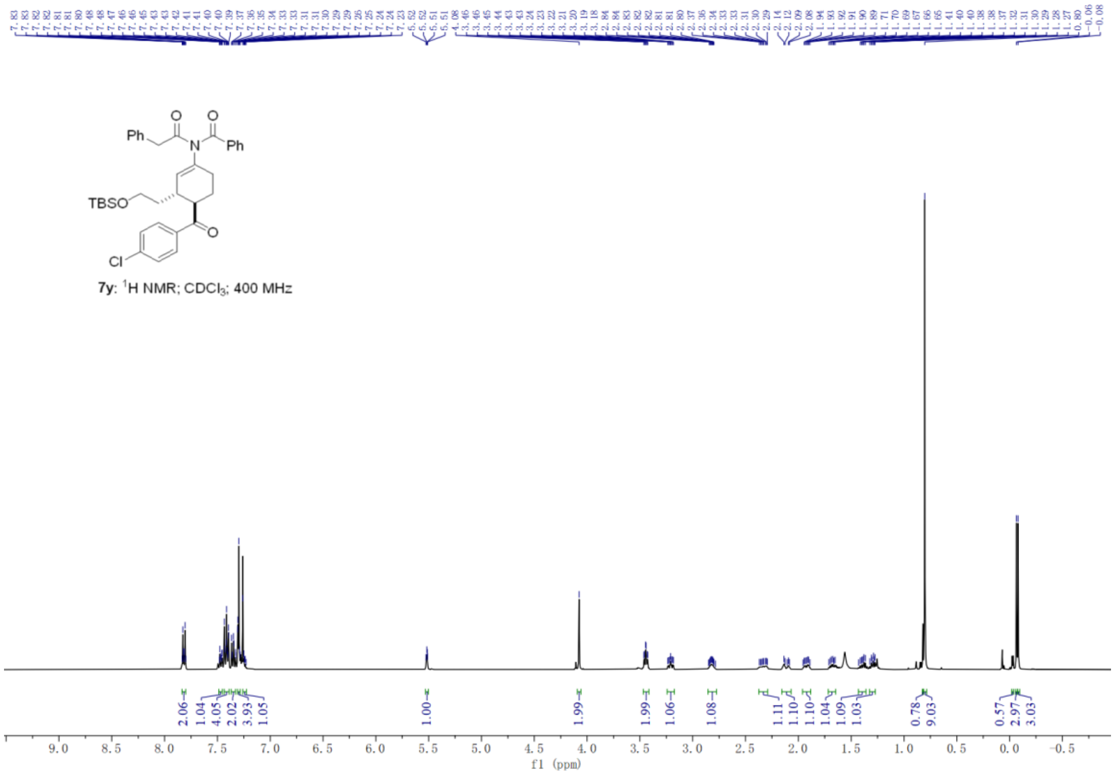
7y: ^{1}H NMR; CDCl$_3$; 400 MHz
f1 (ppm)

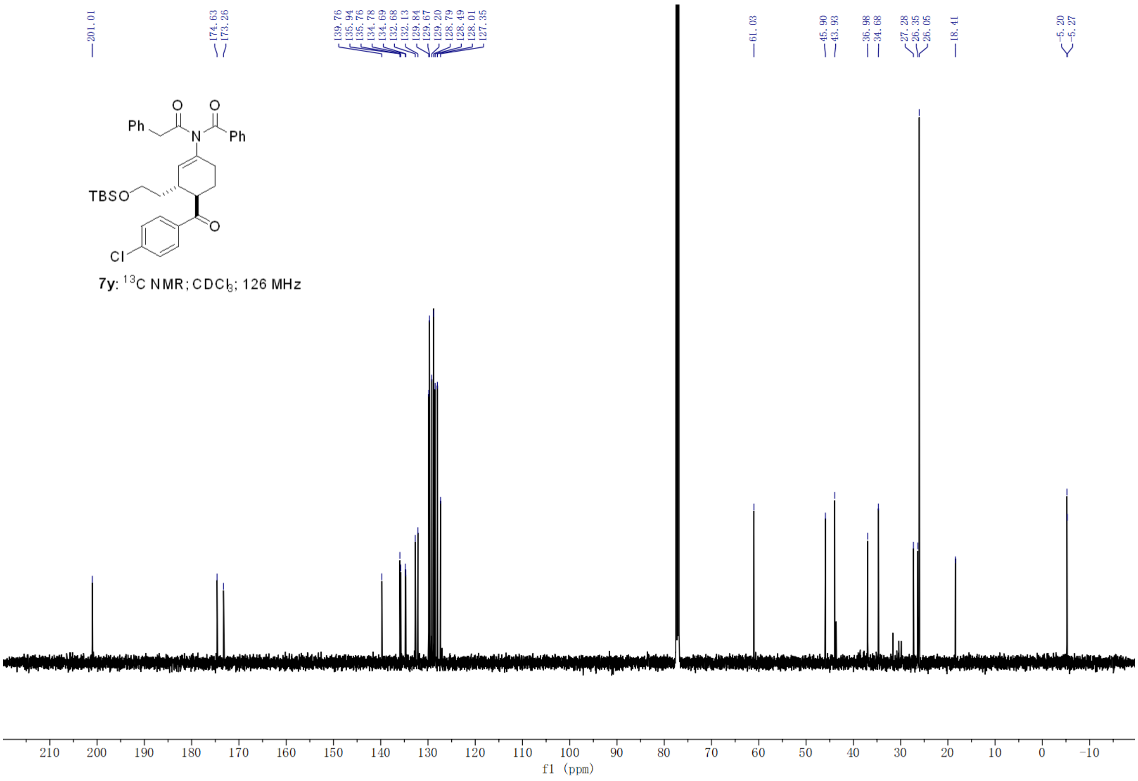

7y: ^{13}C NMR; $CDCl_3$; 126 MHz

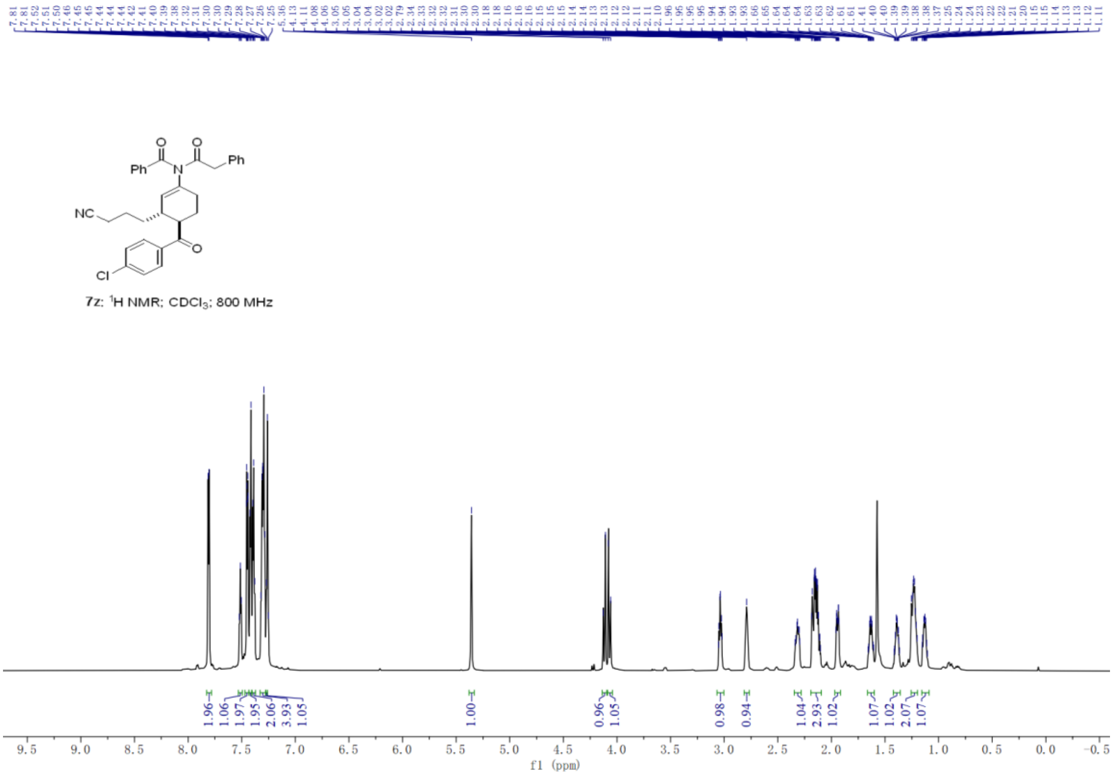

7z: ^{1}H NMR; $CDCl_3$; 800 MHz

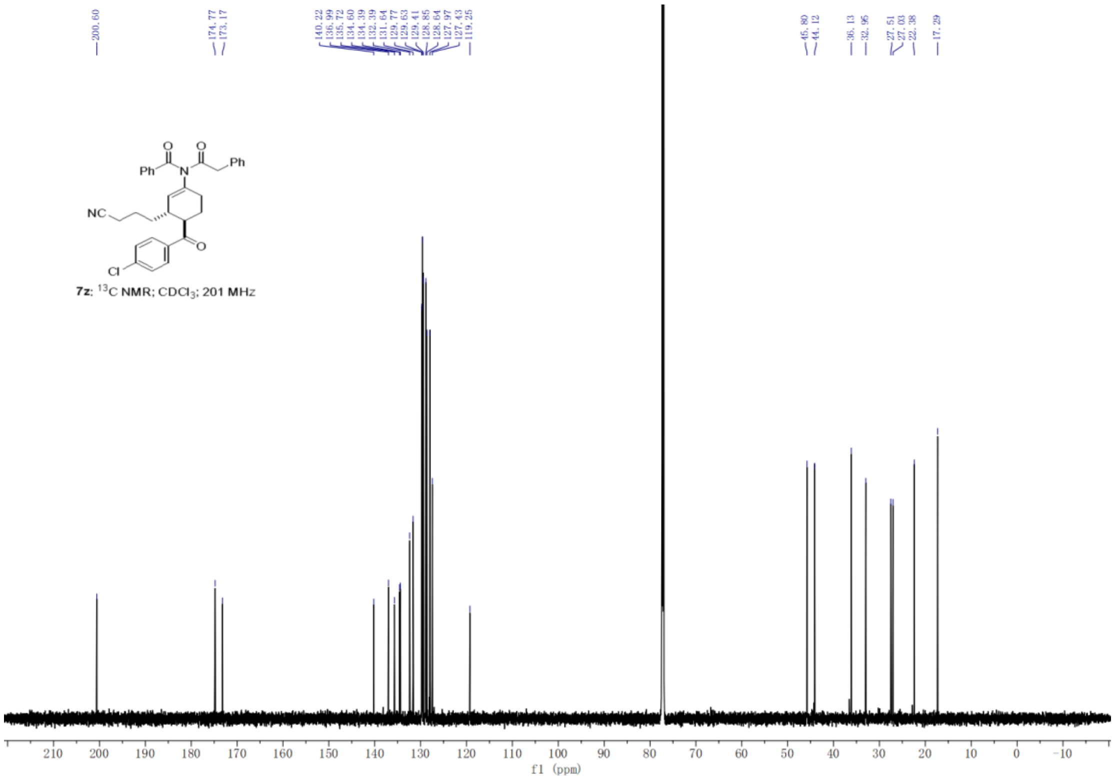
7z: ^{13}C NMR; $CDCl_3$; 201 MHz
f1 (ppm)

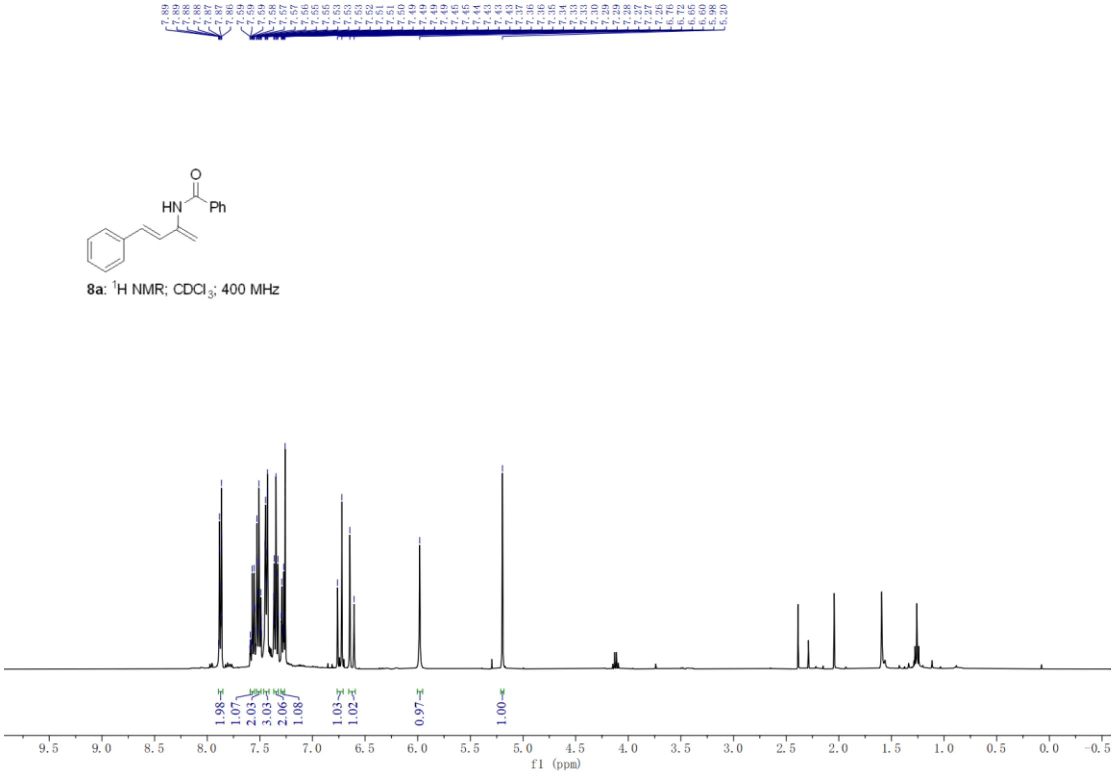
8a: ^{1}H NMR; $CDCl_3$; 400 MHz
f1 (ppm)

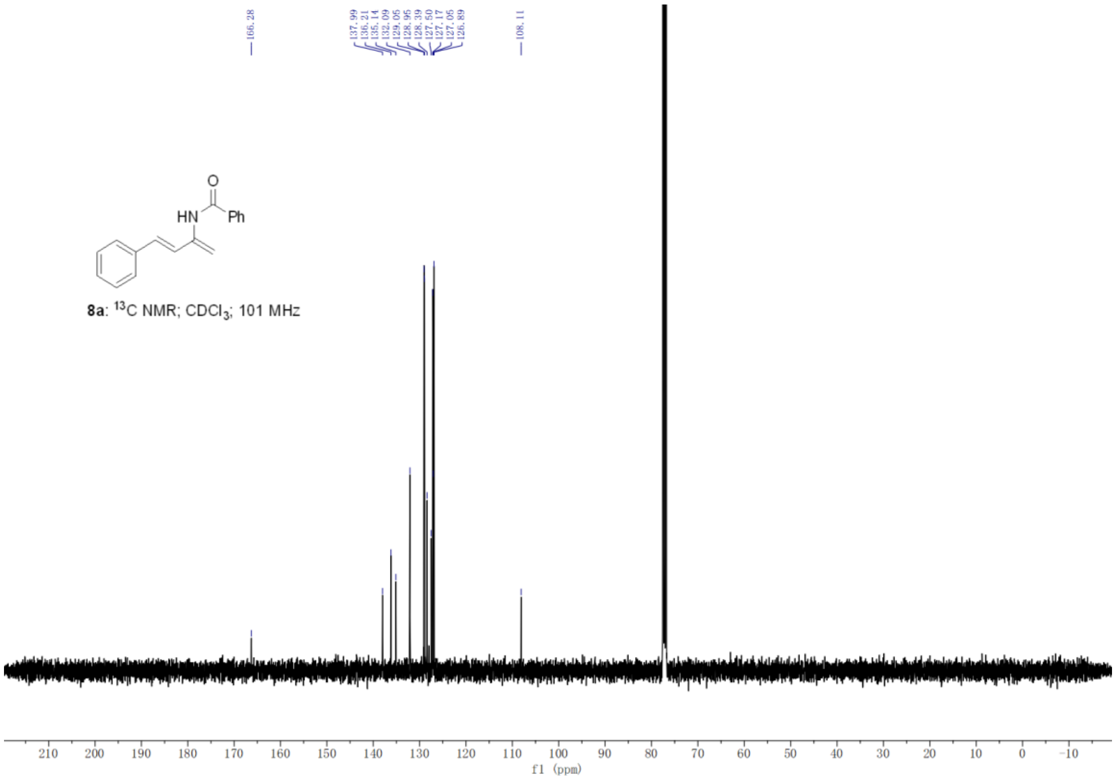
166.28
137.99
136.21
135.14
132.09
129.05
128.95
128.39
127.50
127.17
127.05
126.89
108.11
HN
O
Ph
8a: 13C NMR; CDCl3; 101 MHz
f1 (ppm)

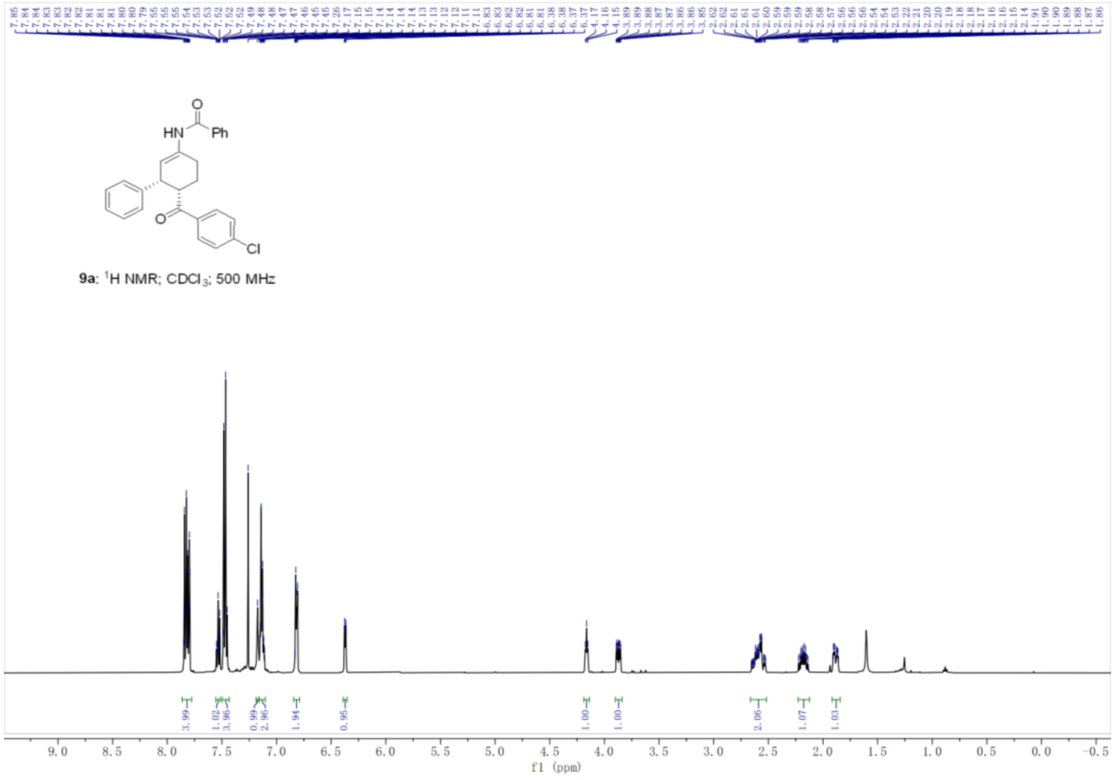
HN
O
Ph
Cl
9a: 1H NMR; CDCl3; 500 MHz
f1 (ppm)

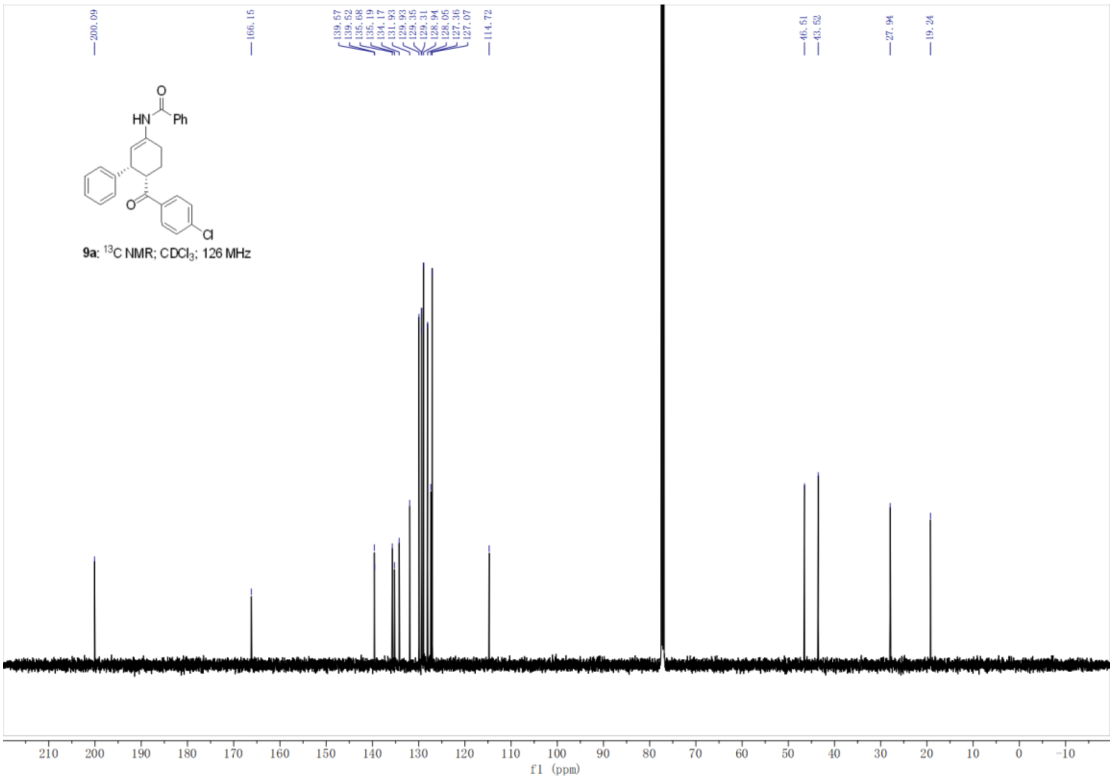
200.09
166.15
139.57
139.52
135.68
135.19
134.17
131.93
129.93
129.35
129.31
128.94
128.05
127.36
127.07
114.72
46.51
43.52
27.94
19.24
HN
O
Ph
O
Cl
9a; 13C NMR; CDCl3; 126 MHz
f1 (ppm)

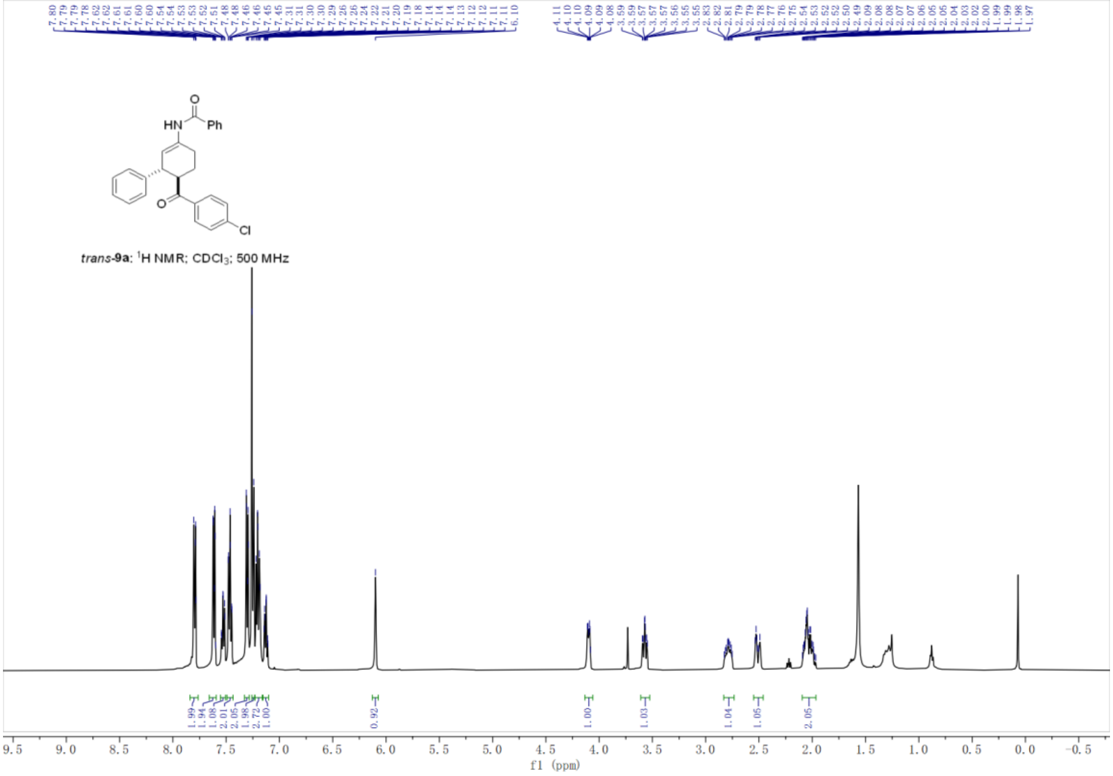
HN
O
Ph
O
Cl
trans-9a; 1H NMR; CDCl3; 500 MHz
1.99
1.94
1.08
2.01
2.05
1.98
2.72
1.00
0.92
1.00
1.03
1.04
1.05
2.05
f1 (ppm)

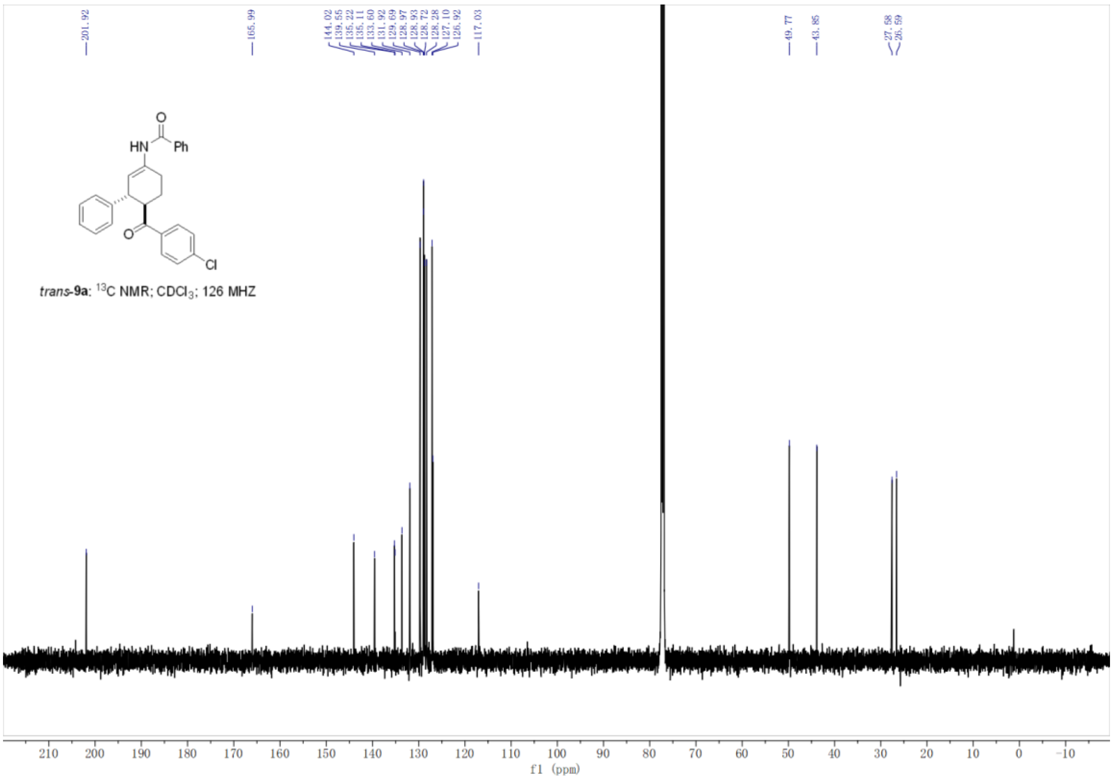
201.92
165.99
144.02
139.55
135.22
135.11
133.60
131.92
129.69
128.97
128.93
128.72
128.28
127.10
126.92
117.03
49.77
43.85
27.58
26.59
HN
Ph
O
Cl
trans-**9a**: ^{13}C NMR; $CDCl_3$; 126 MHZ
f1 (ppm)

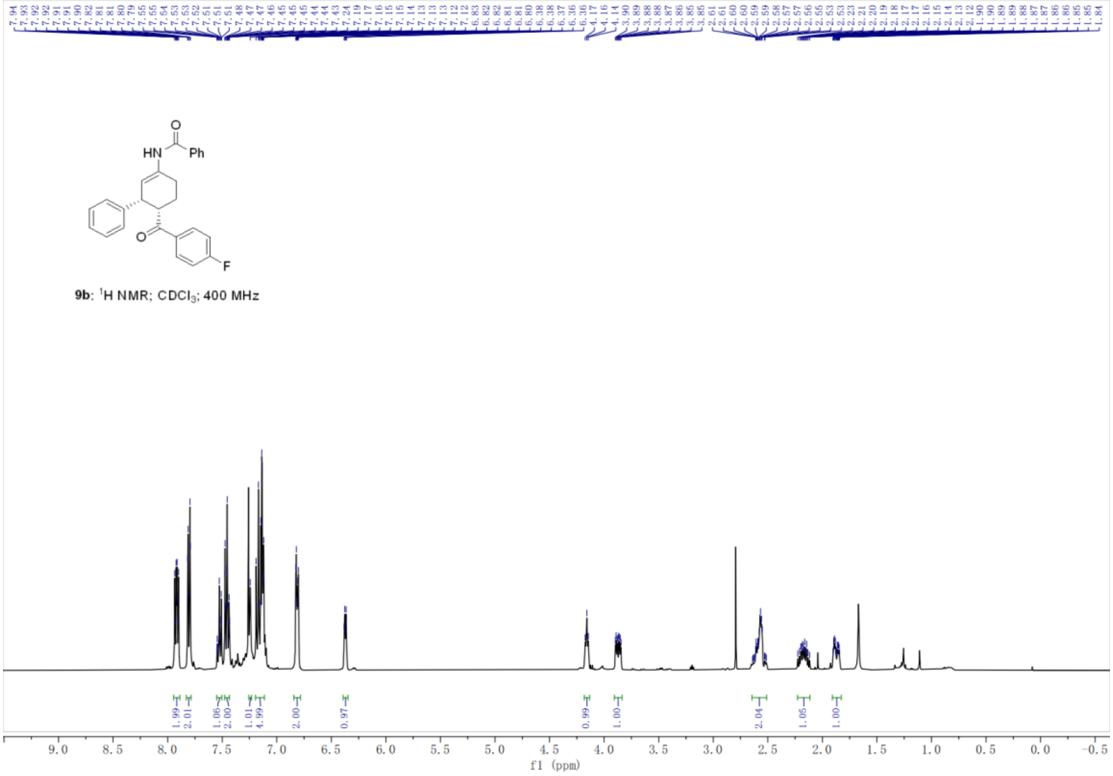
HN
Ph
O
F
9b: ^{1}H NMR; $CDCl_3$; 400 MHz
f1 (ppm)

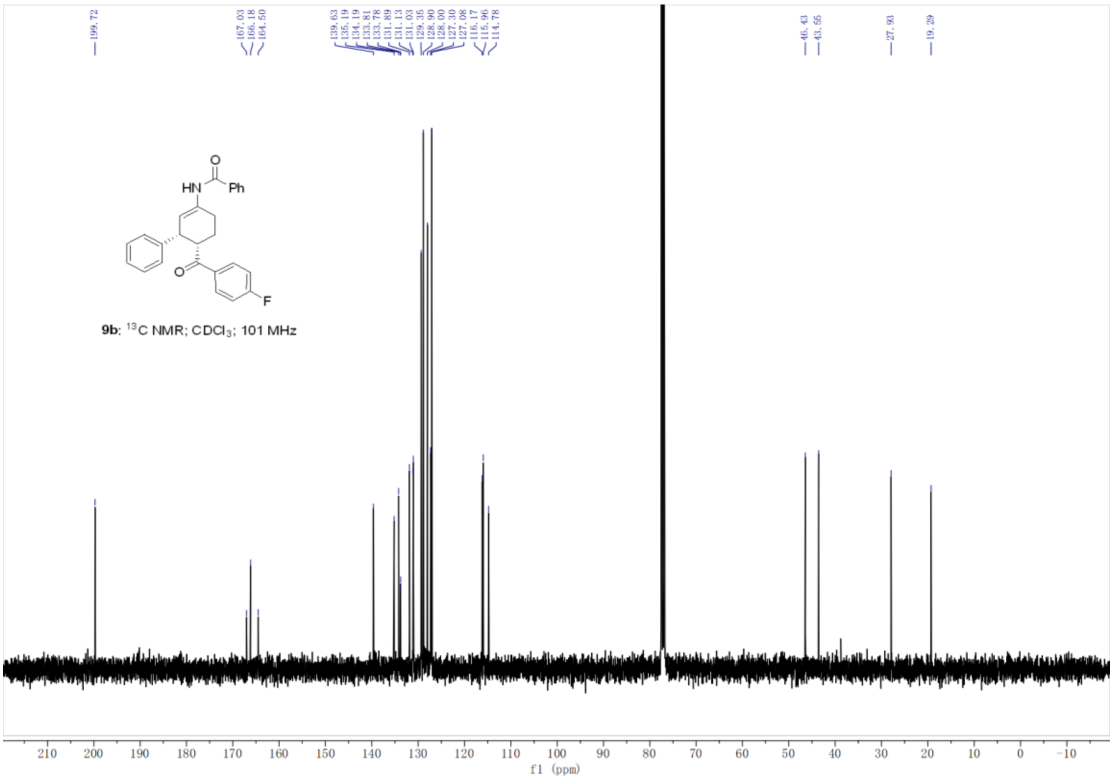
199.72
167.03
166.18
164.50
139.63
135.19
134.19
133.81
133.78
131.89
131.13
131.03
129.35
128.90
128.00
127.30
127.08
116.17
115.96
114.78
46.43
43.55
27.93
19.29
HN
Ph
O
O
F
9b: ^{13}C NMR; $CDCl_3$; 101 MHz
f1 (ppm)

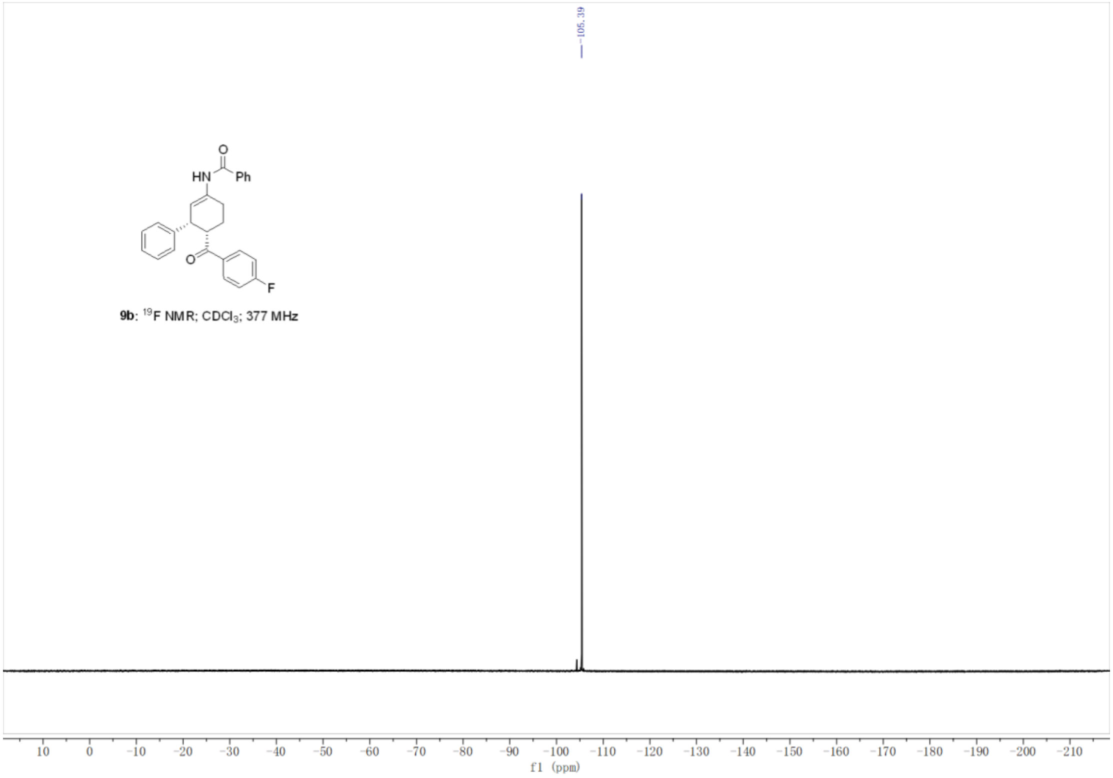
-105.39
HN
Ph
O
O
F
9b: ^{19}F NMR; $CDCl_3$; 377 MHz
f1 (ppm)

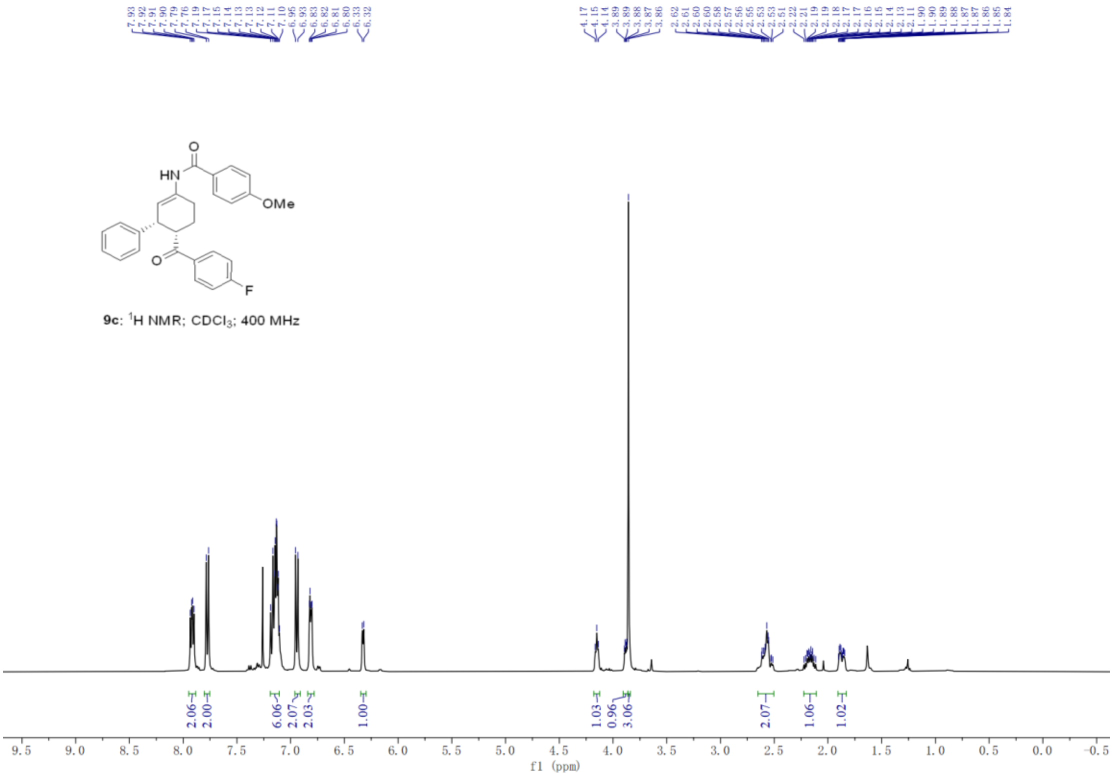
9c: 1H NMR; CDCl3; 400 MHz
f1 (ppm)

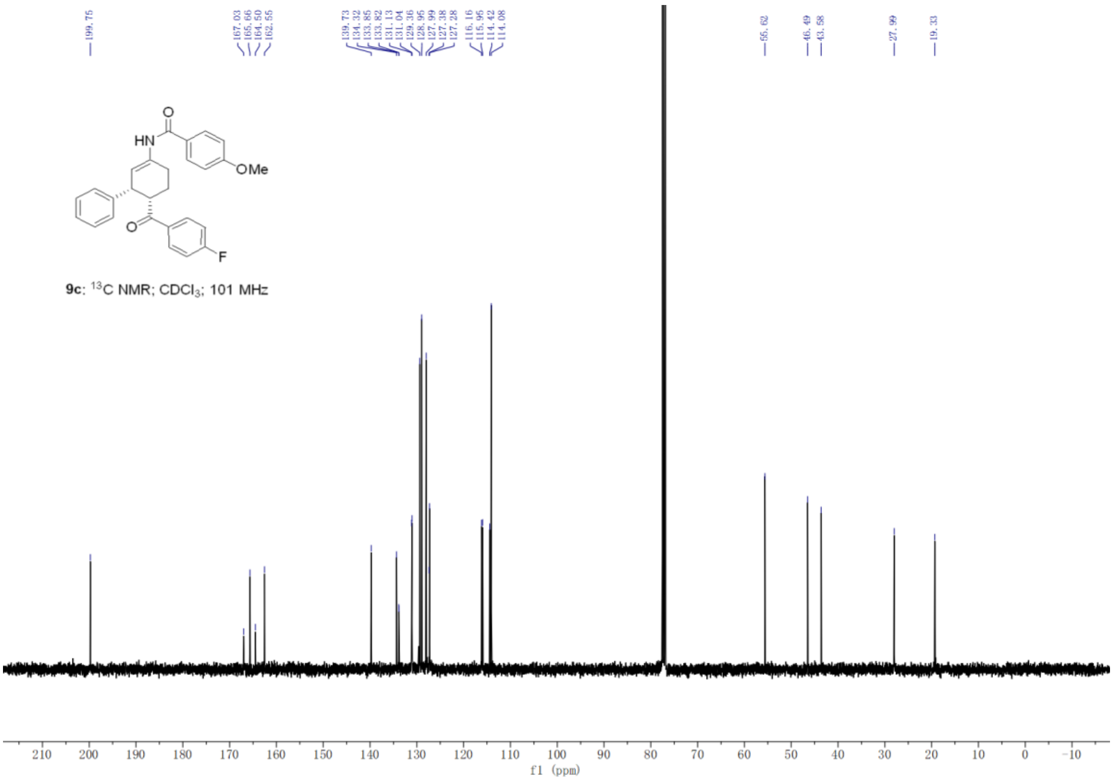
9c: 13C NMR; CDCl3; 101 MHz
f1 (ppm)

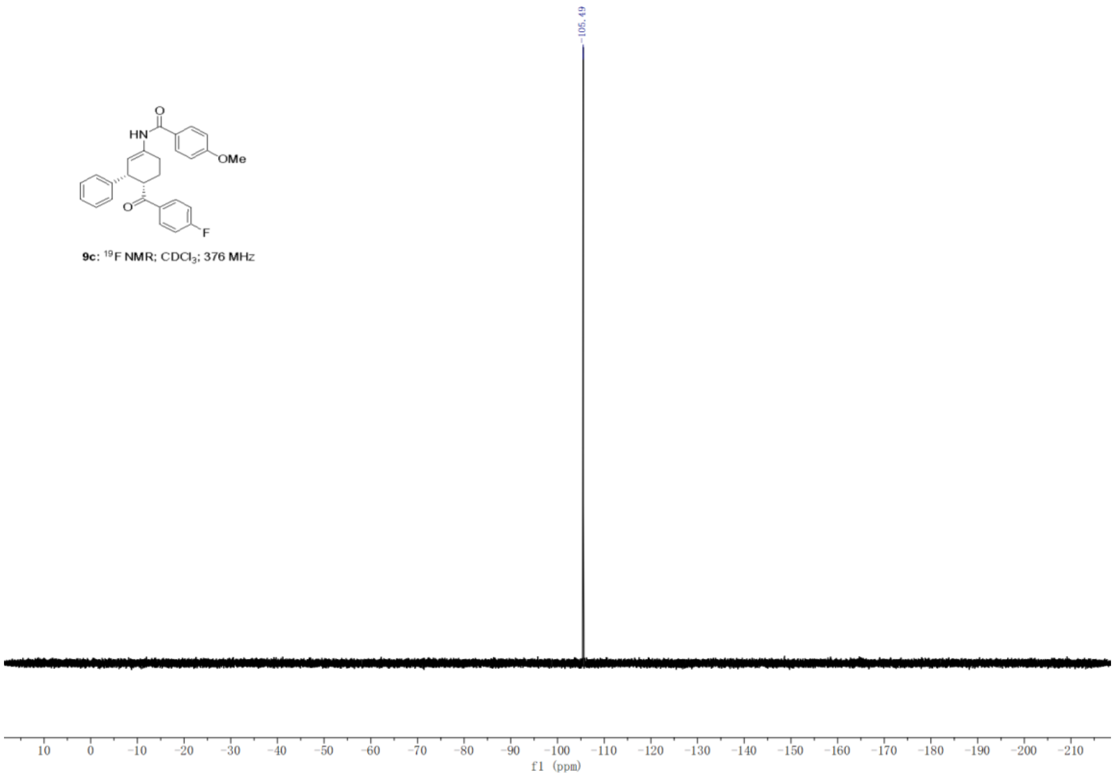

9c: ^{19}F NMR; $CDCl_3$; 376 MHz

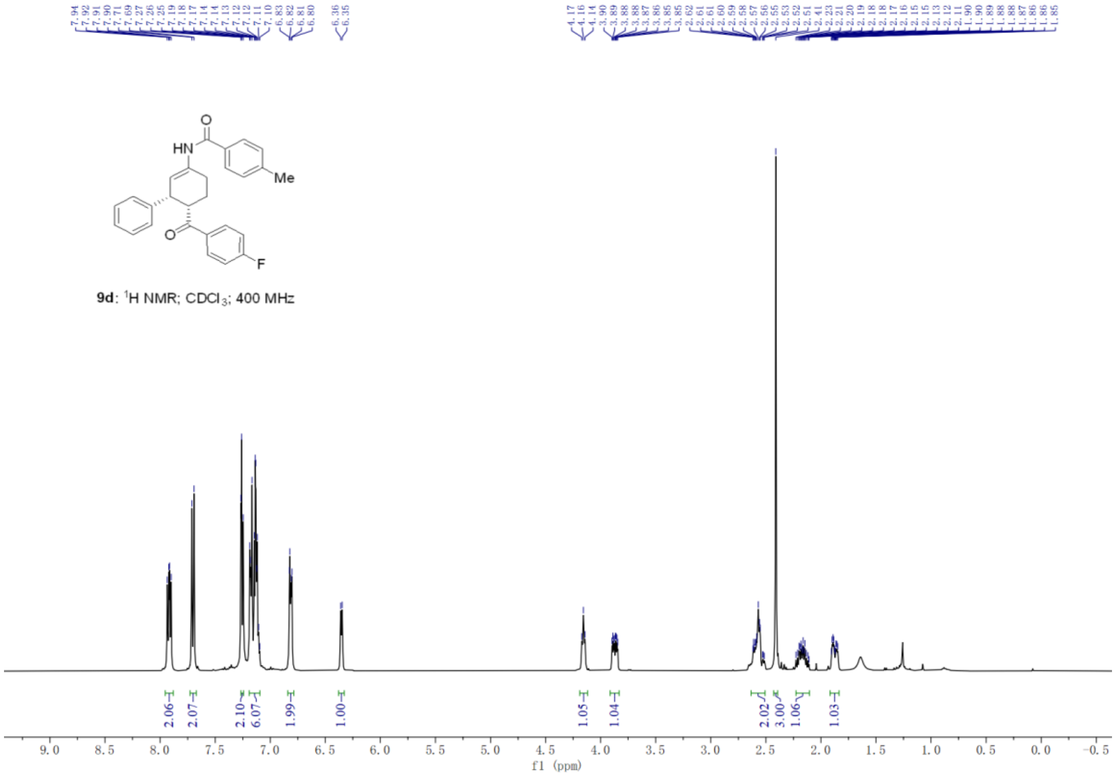

9d: ^{1}H NMR; $CDCl_3$; 400 MHz

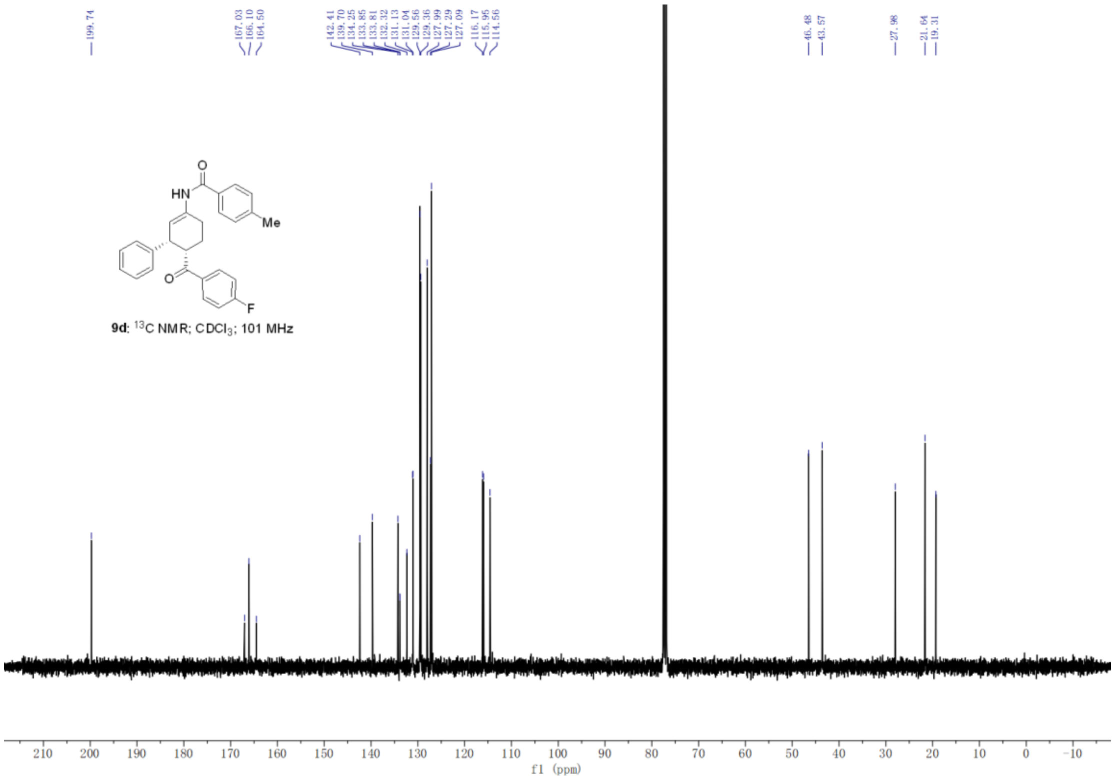

9d: ^{13}C NMR; $CDCl_3$; 101 MHz

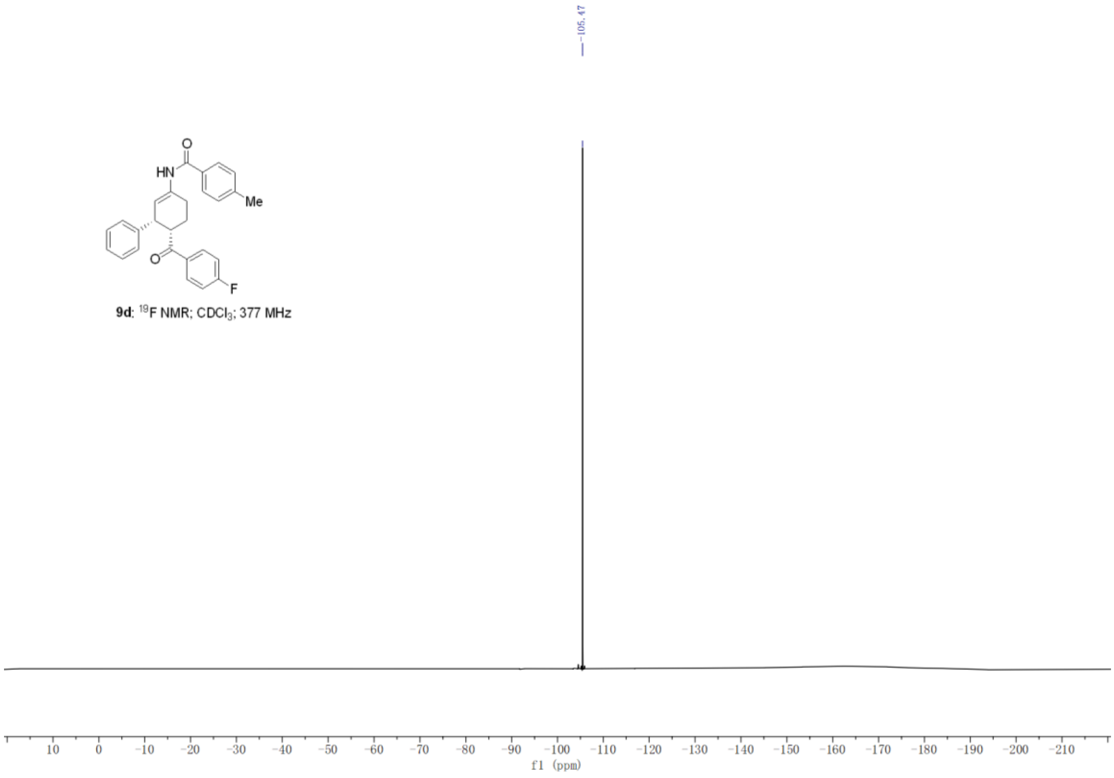

9d: ^{19}F NMR; $CDCl_3$; 377 MHz

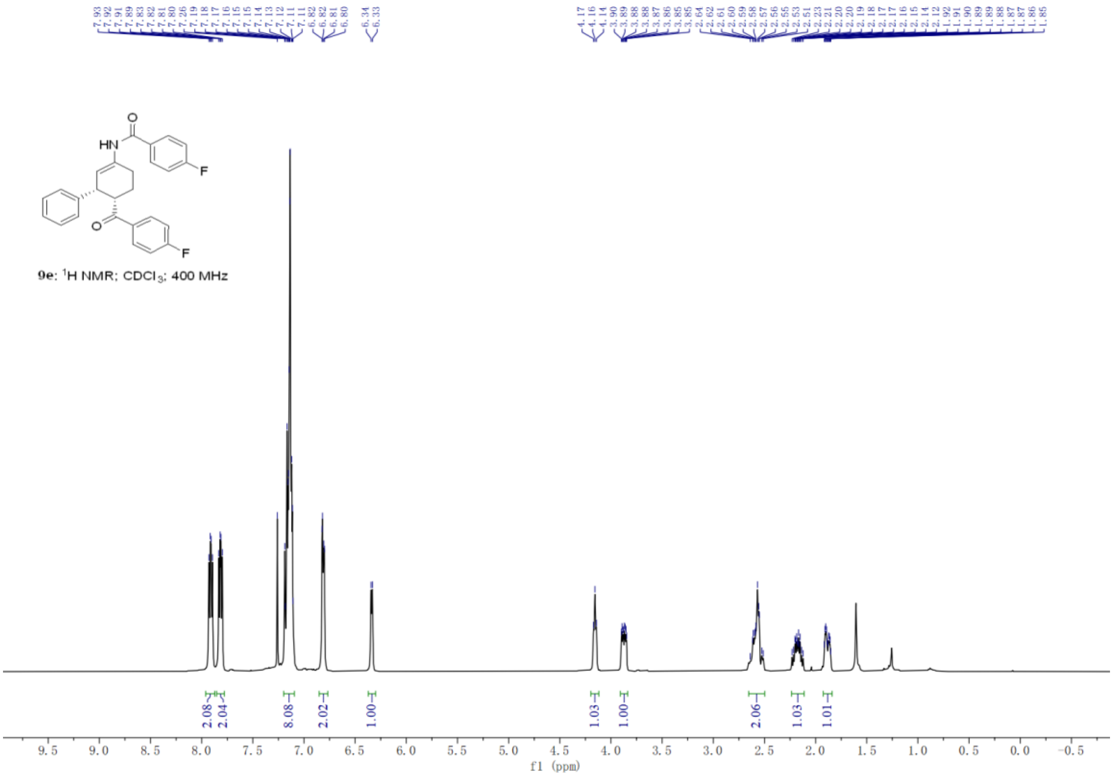

9e: ^{1}H NMR; $CDCl_3$; 400 MHz

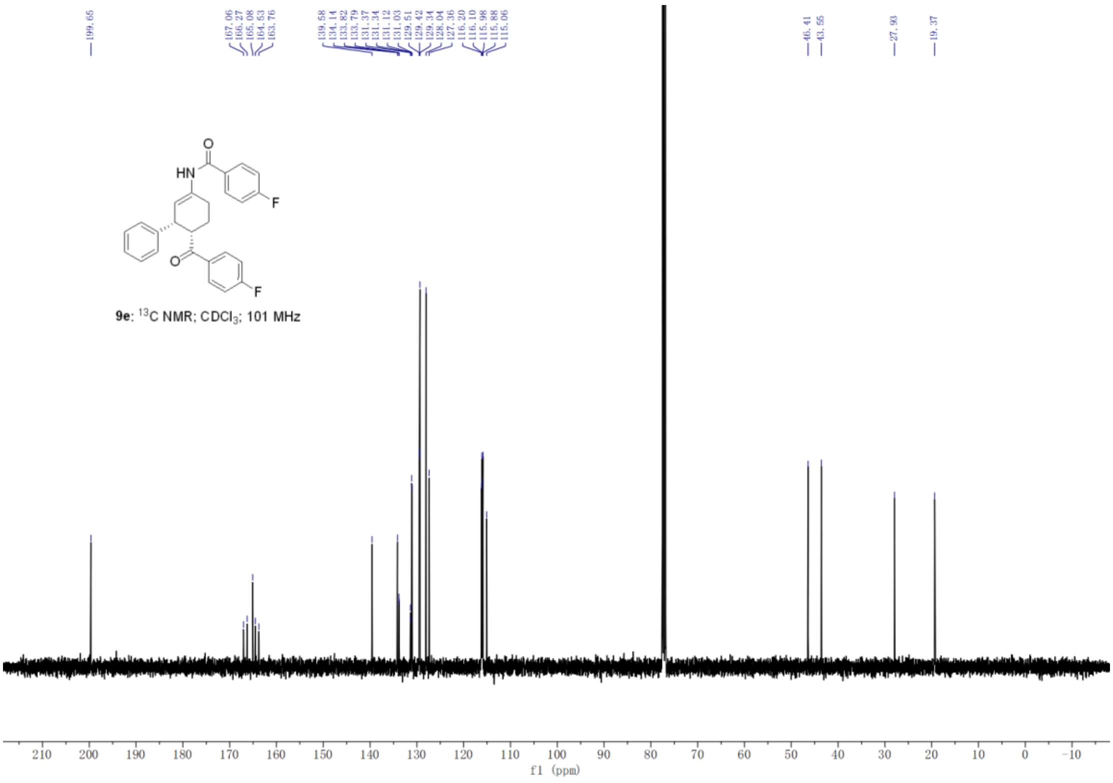

9e: ^{13}C NMR; $CDCl_3$; 101 MHz

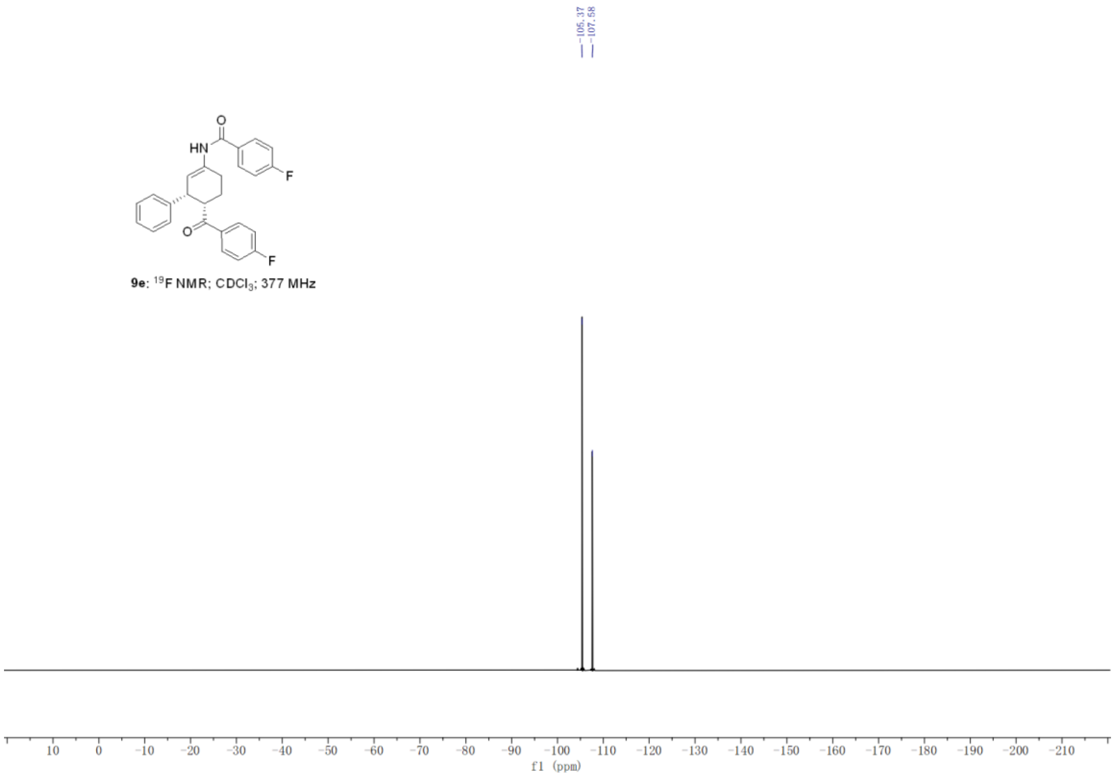
-105.37
-107.58
HN
O
F
O
F
9e: ^{19}F NMR; CDCl$_3$; 377 MHz
f1 (ppm)

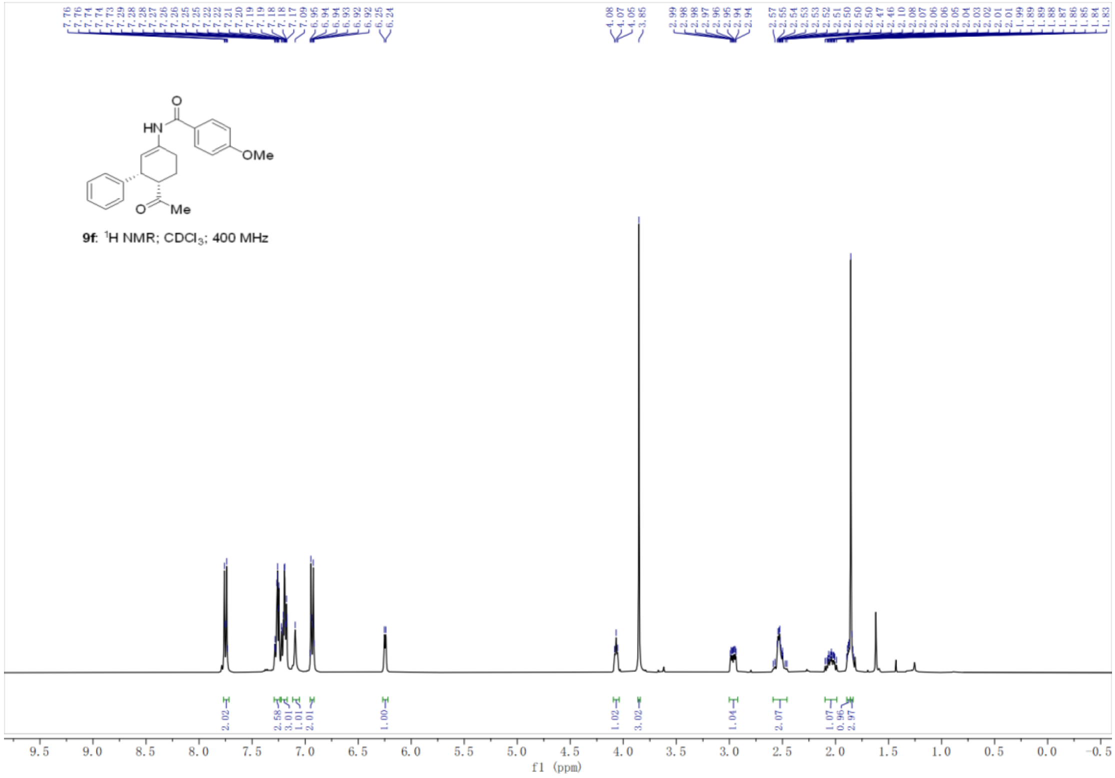
HN
O
OMe
O
Me
9f: ^{1}H NMR; CDCl$_3$; 400 MHz
f1 (ppm)

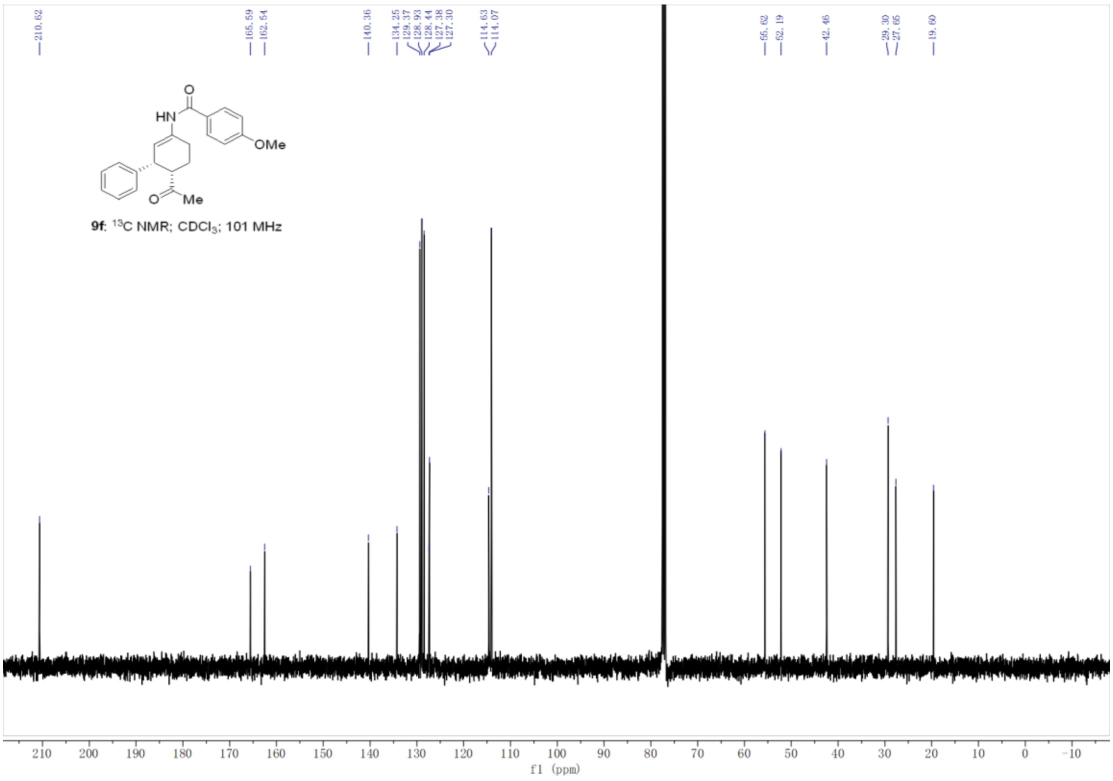
210.62
165.59
162.54
140.36
134.25
129.17
128.93
128.44
127.38
127.30
114.63
114.07
55.62
52.19
42.46
29.30
27.65
19.60
HN
O
OMe
O
Me
9f; ^{13}C NMR; $CDCl_3$; 101 MHz
210 200 190 180 170 160 150 140 130 120 110 100 90 80 70 60 50 40 30 20 10 0 −10
f1 (ppm)

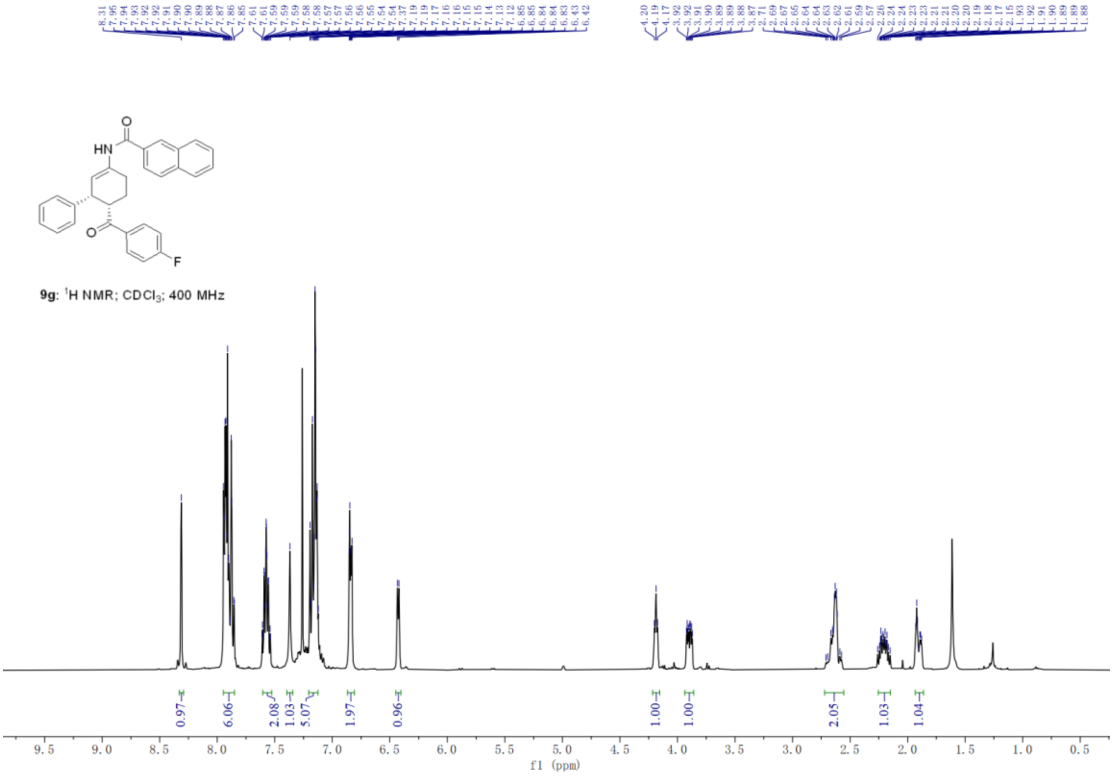
8.31 7.95 7.94 7.93 7.92 7.92 7.91 7.90 7.89 7.88 7.87 7.86 7.85 7.61 7.61 7.59 7.59 7.58 7.58 7.57 7.57 7.56 7.55 7.54 7.54 7.37 7.19 7.19 7.17 7.16 7.16 7.15 7.14 7.13 7.12 6.85 6.85 6.84 6.84 6.83 6.43 6.42
4.20 4.19 4.17 3.92 3.91 3.90 3.89 3.89 3.88 3.87 2.71 2.69 2.67 2.65 2.64 2.63 2.62 2.61 2.59 2.57 2.26 2.24 2.24 2.23 2.23 2.21 2.21 2.20 2.20 2.19 2.18 2.17 2.15 1.93 1.92 1.91 1.90 1.89 1.88
HN
O
O
F
9g; ^{1}H NMR; $CDCl_3$; 400 MHz
0.97 6.06 2.08 1.03 5.07 1.97 0.96 1.00 1.00 2.05 1.03 1.04
9.5 9.0 8.5 8.0 7.5 7.0 6.5 6.0 5.5 5.0 4.5 4.0 3.5 3.0 2.5 2.0 1.5 1.0 0.5
f1 (ppm)

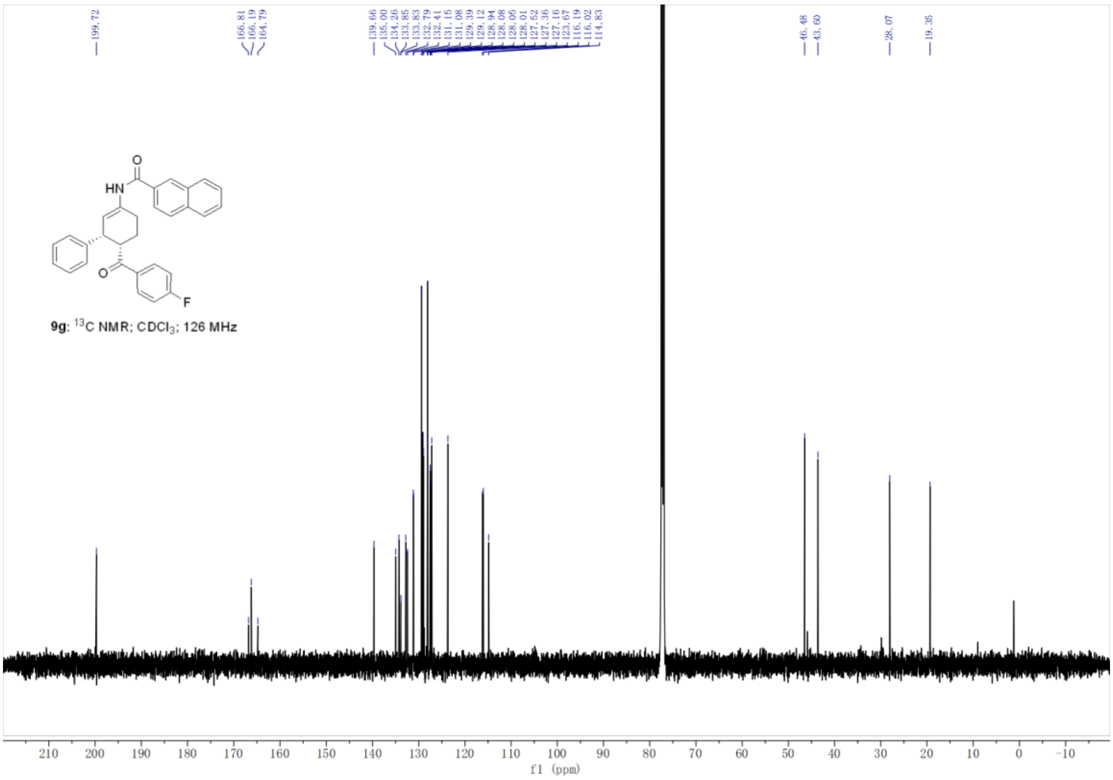

9g: ^{13}C NMR; $CDCl_3$; 126 MHz

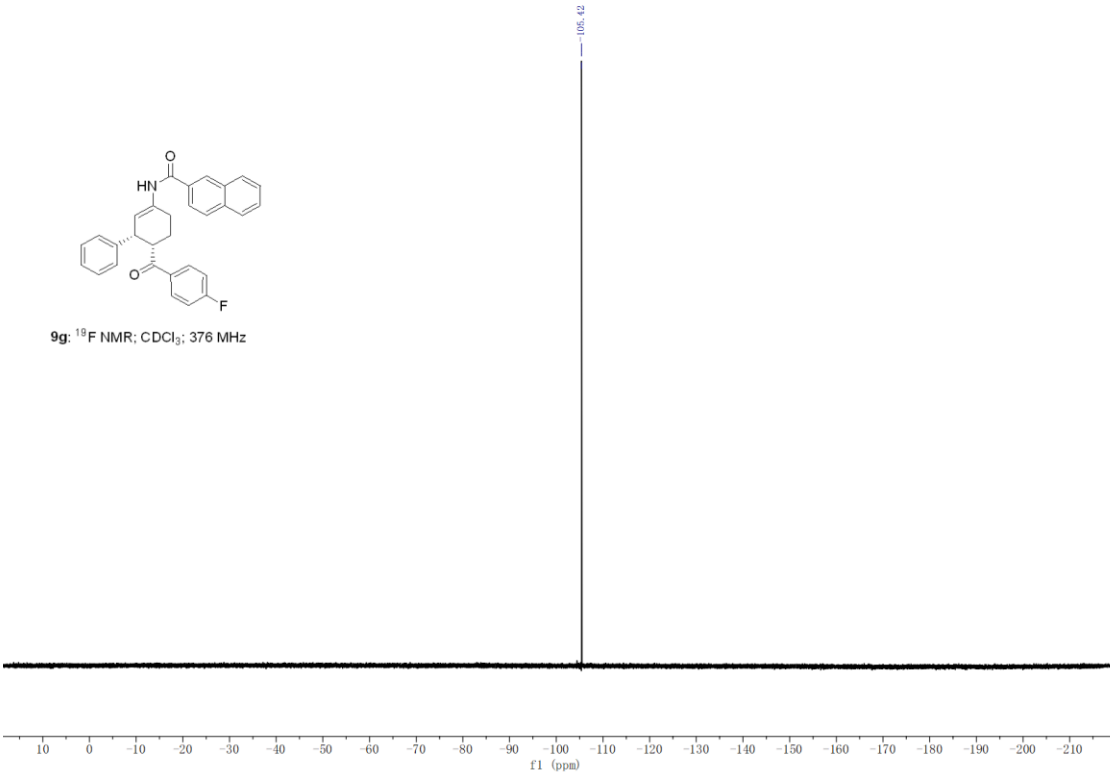

9g: ^{19}F NMR; $CDCl_3$; 376 MHz

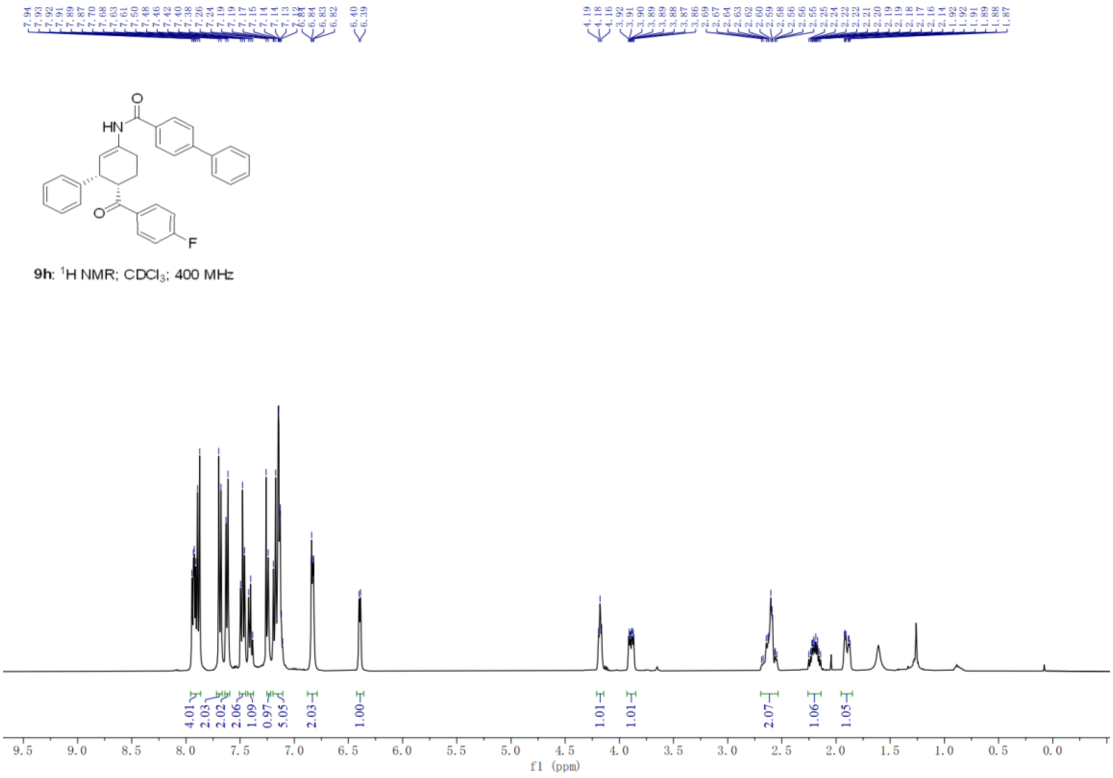

9h: ^{1}H NMR; $CDCl_3$; 400 MHz

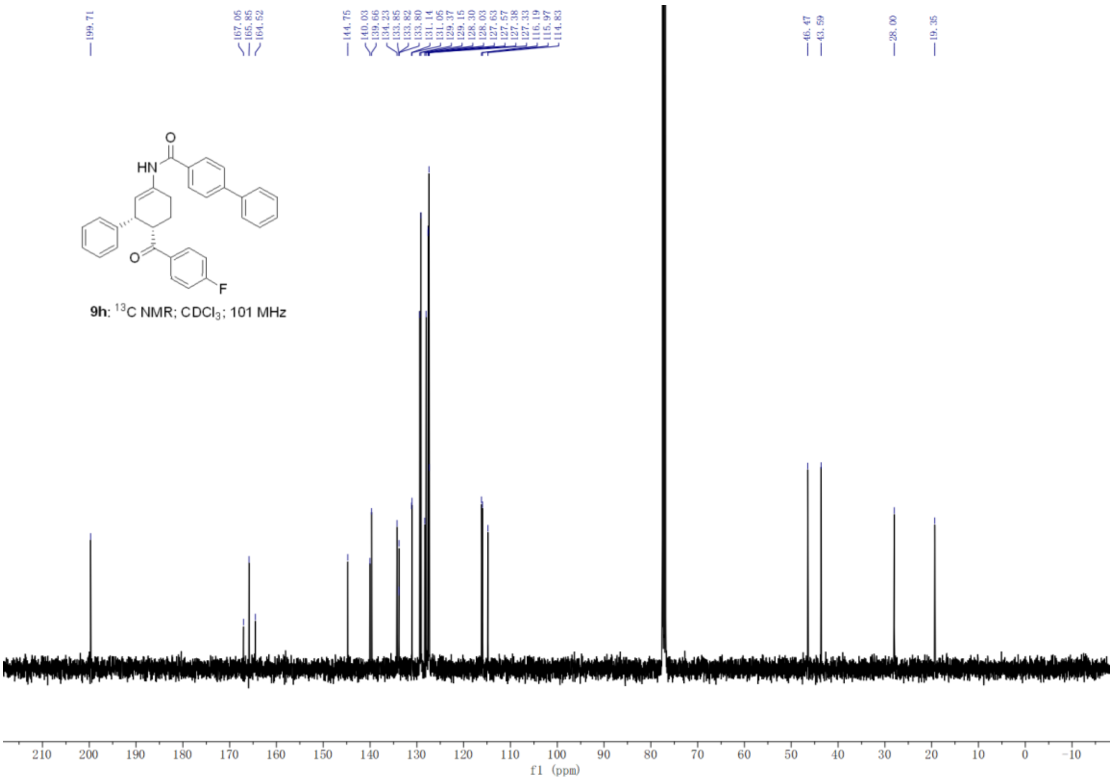

9h: ^{13}C NMR; $CDCl_3$; 101 MHz

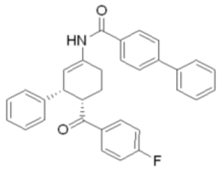

9h: ^{19}F NMR; $CDCl_3$; 377 MHz

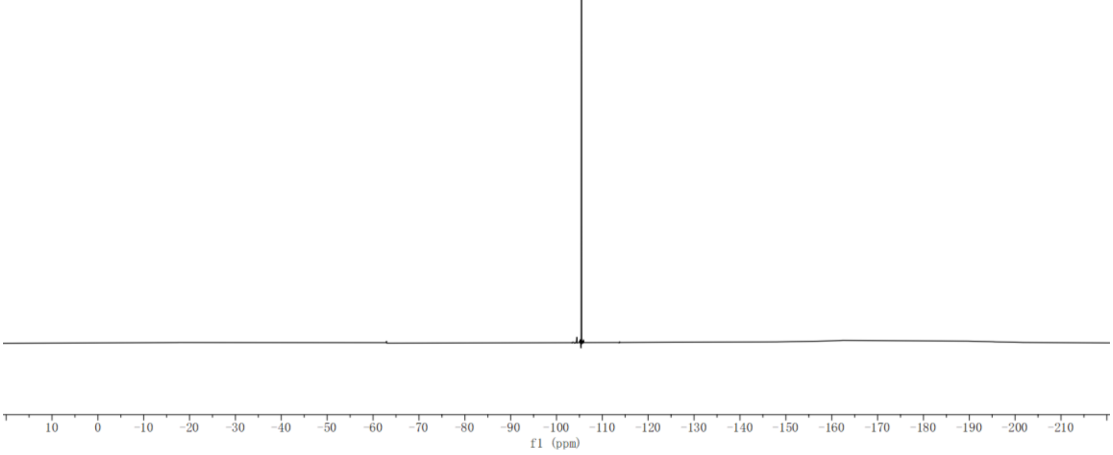

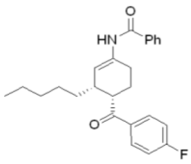

9i: ^{1}H NMR; $CDCl_3$; 400 MHz

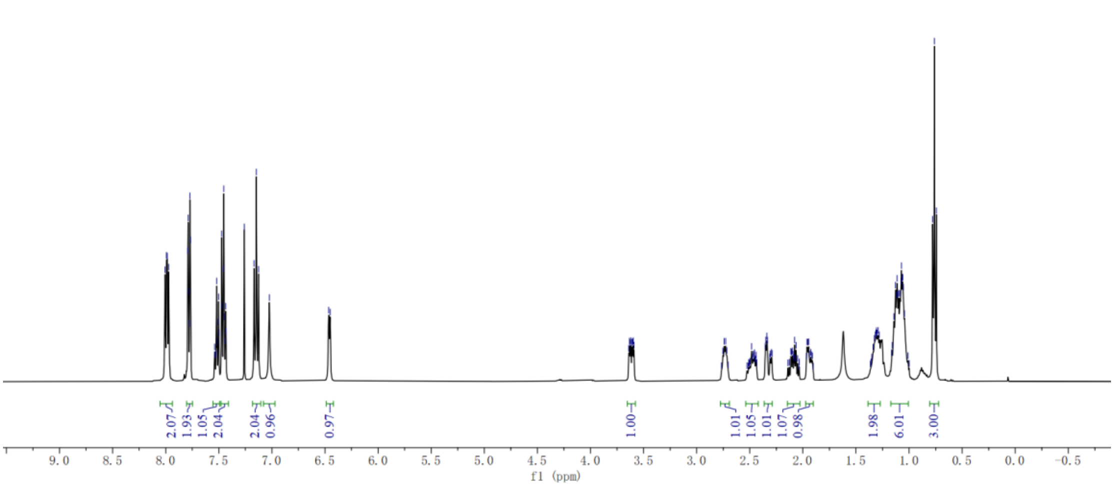

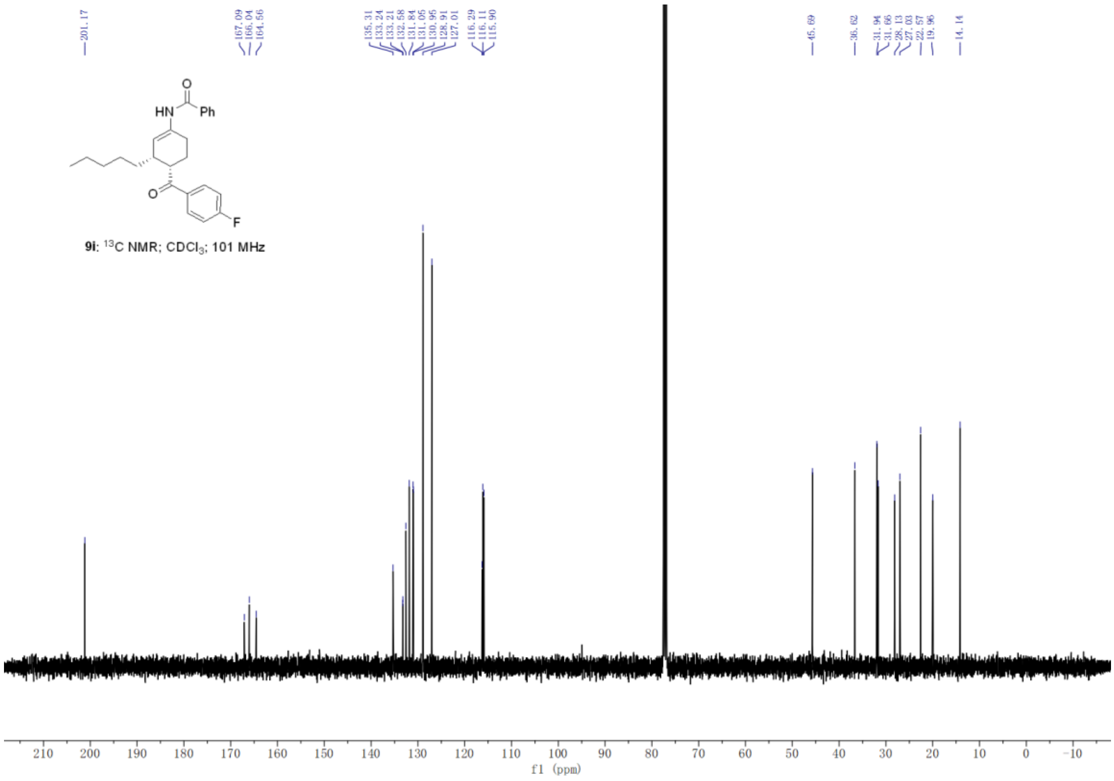

9i: ^{13}C NMR; $CDCl_3$; 101 MHz

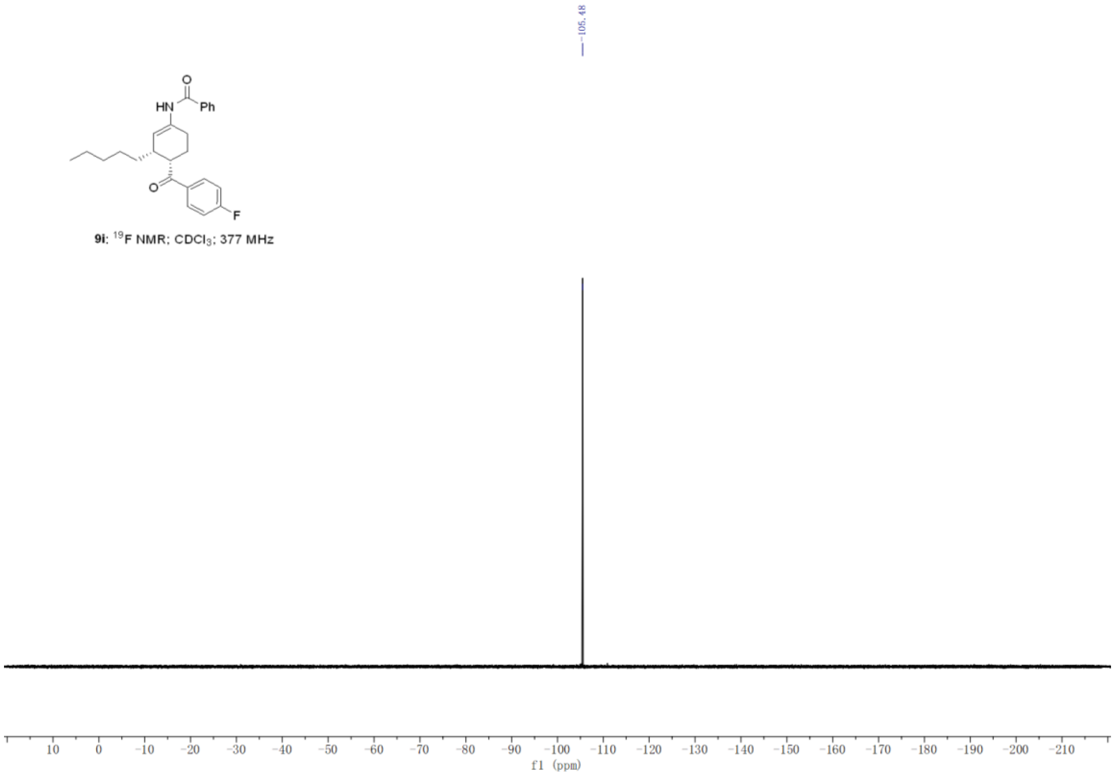

9i: ^{19}F NMR; $CDCl_3$; 377 MHz

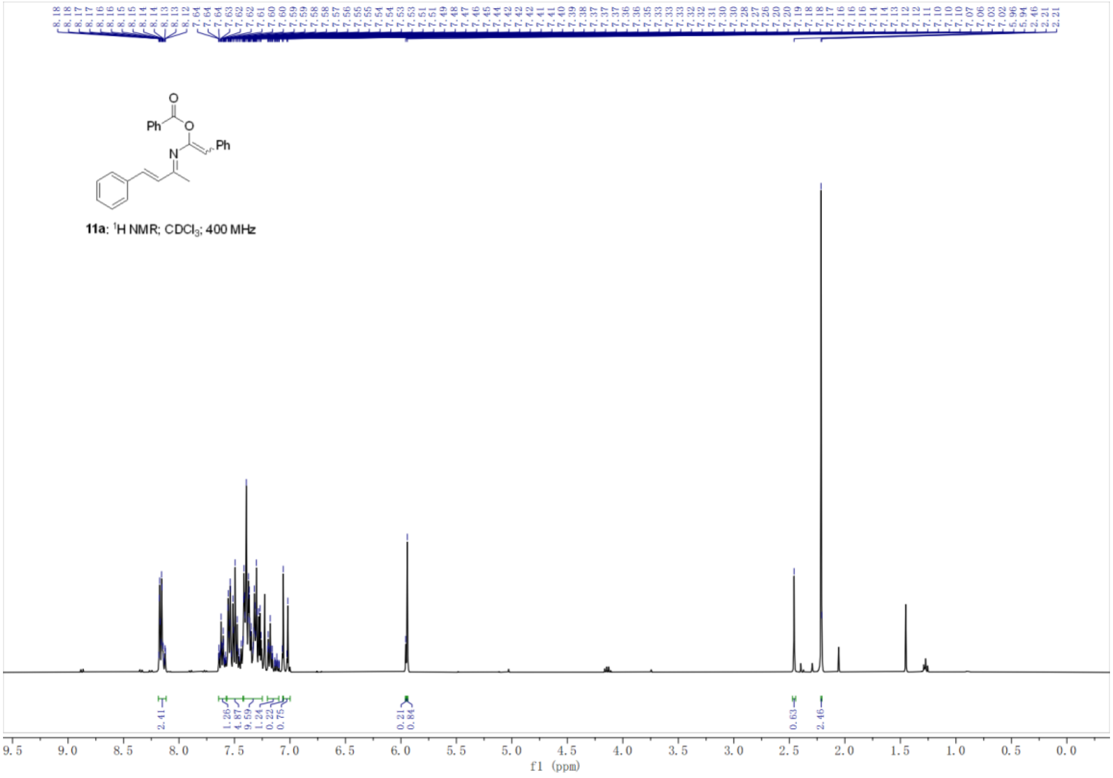

11a; 1H NMR; CDCl3; 400 MHz
f1 (ppm)

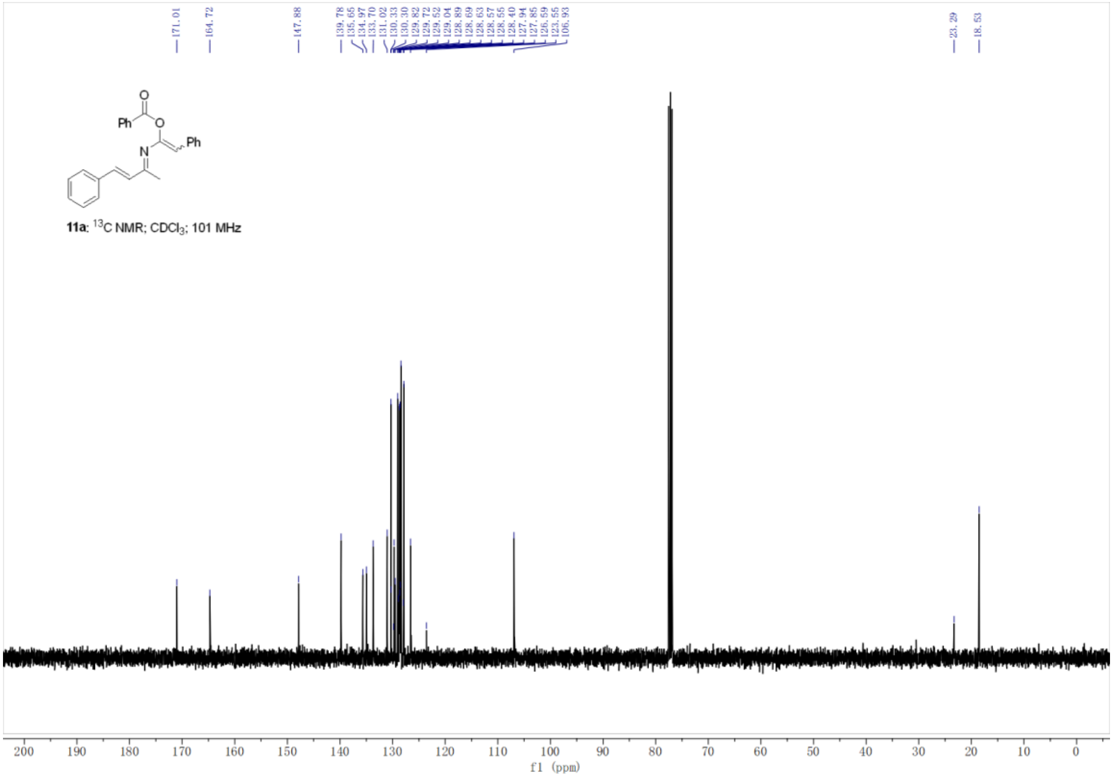

11a; 13C NMR; CDCl3; 101 MHz
f1 (ppm)

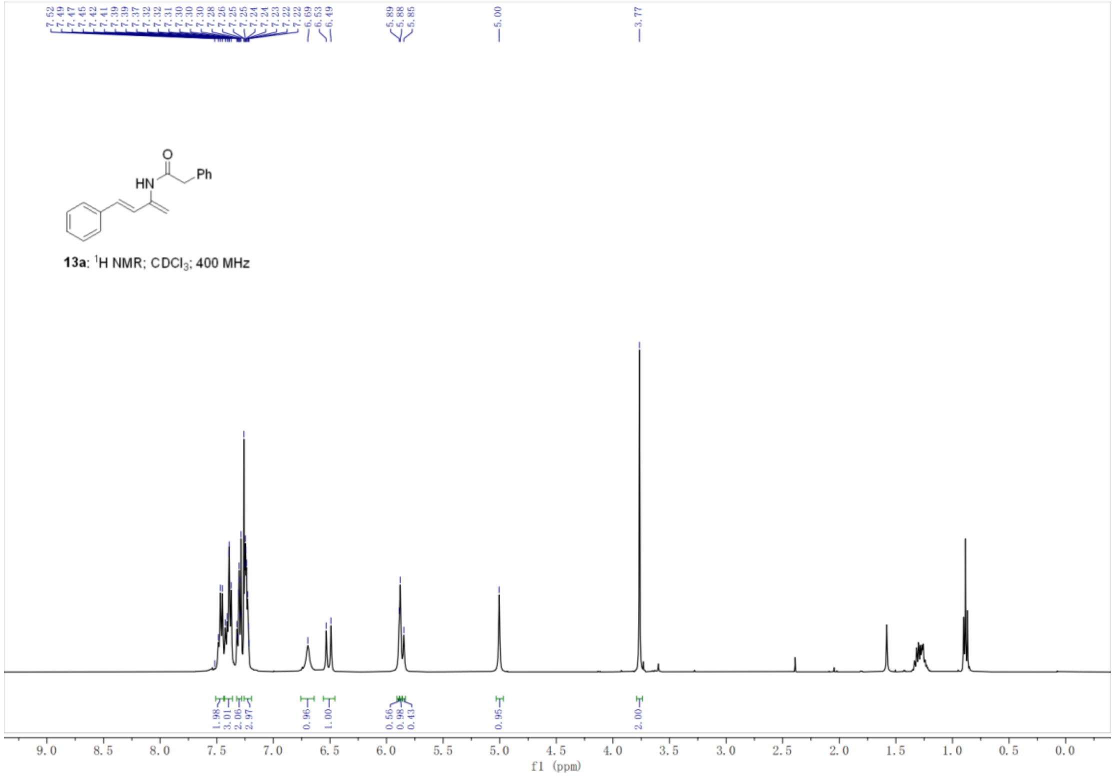

13a: ^{1}H NMR; CDCl$_3$; 400 MHz
f1 (ppm)

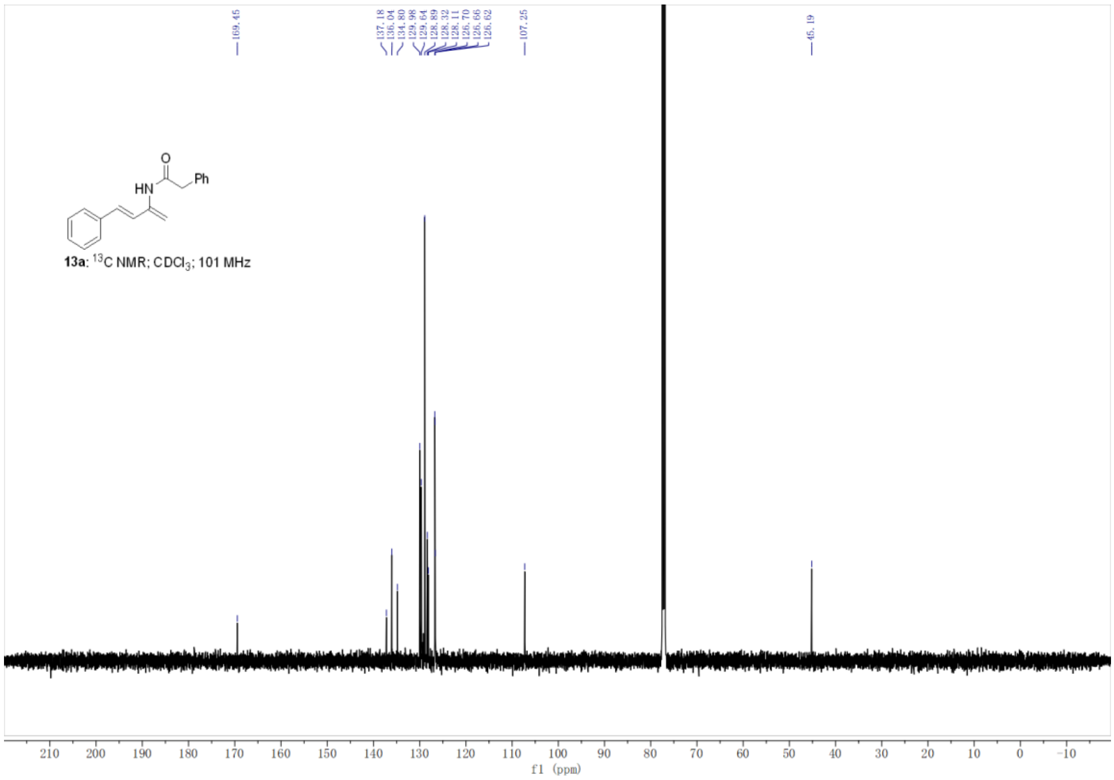

13a: ^{13}C NMR; CDCl$_3$; 101 MHz
f1 (ppm)

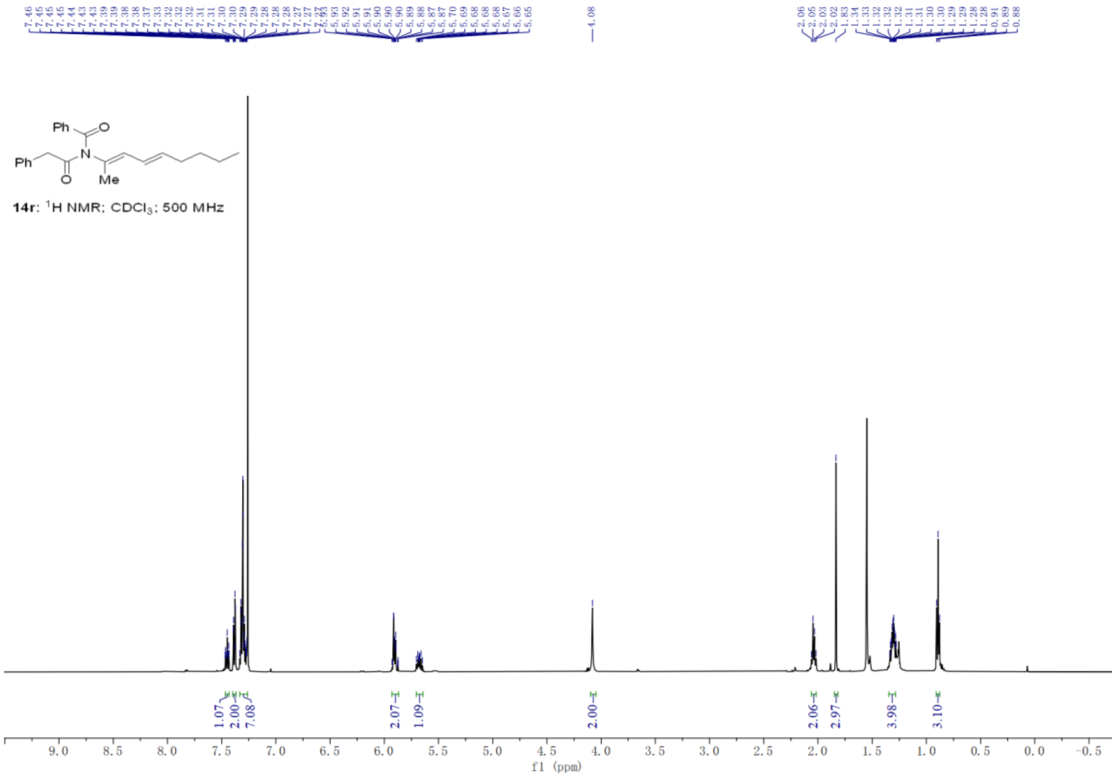

14r: ^{1}H NMR; $CDCl_3$; 500 MHz

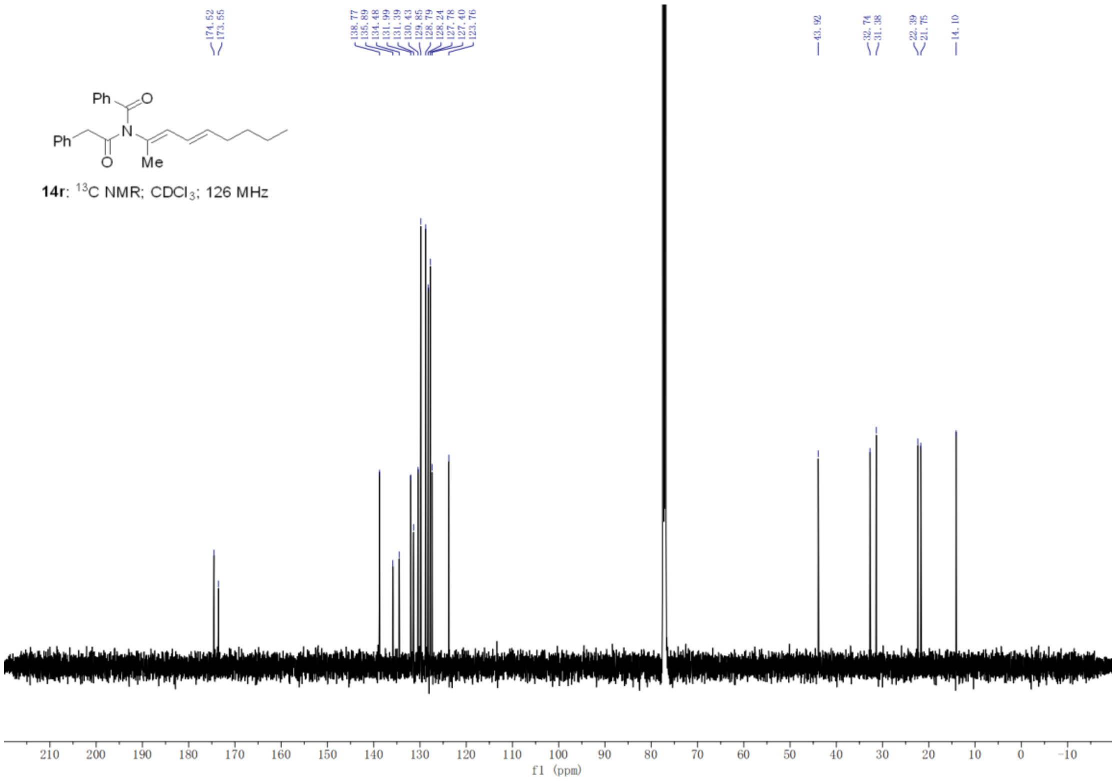

14r: ^{13}C NMR; $CDCl_3$; 126 MHz

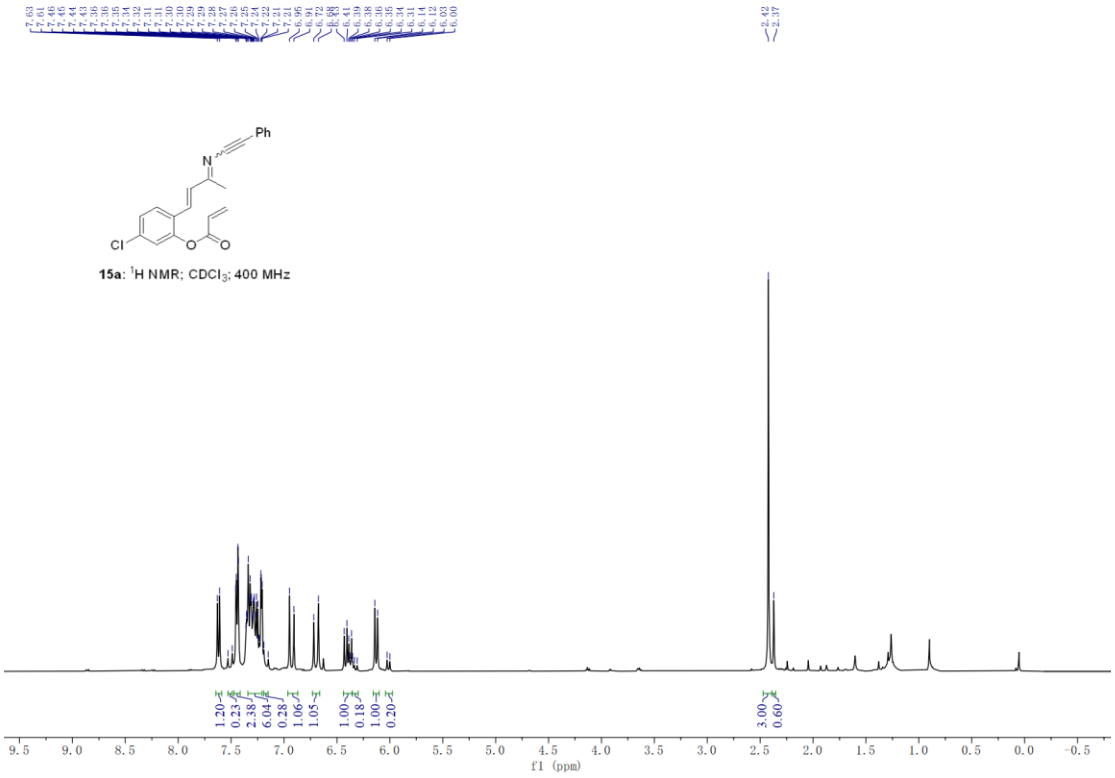

15a: ^1H NMR; $CDCl_3$; 400 MHz

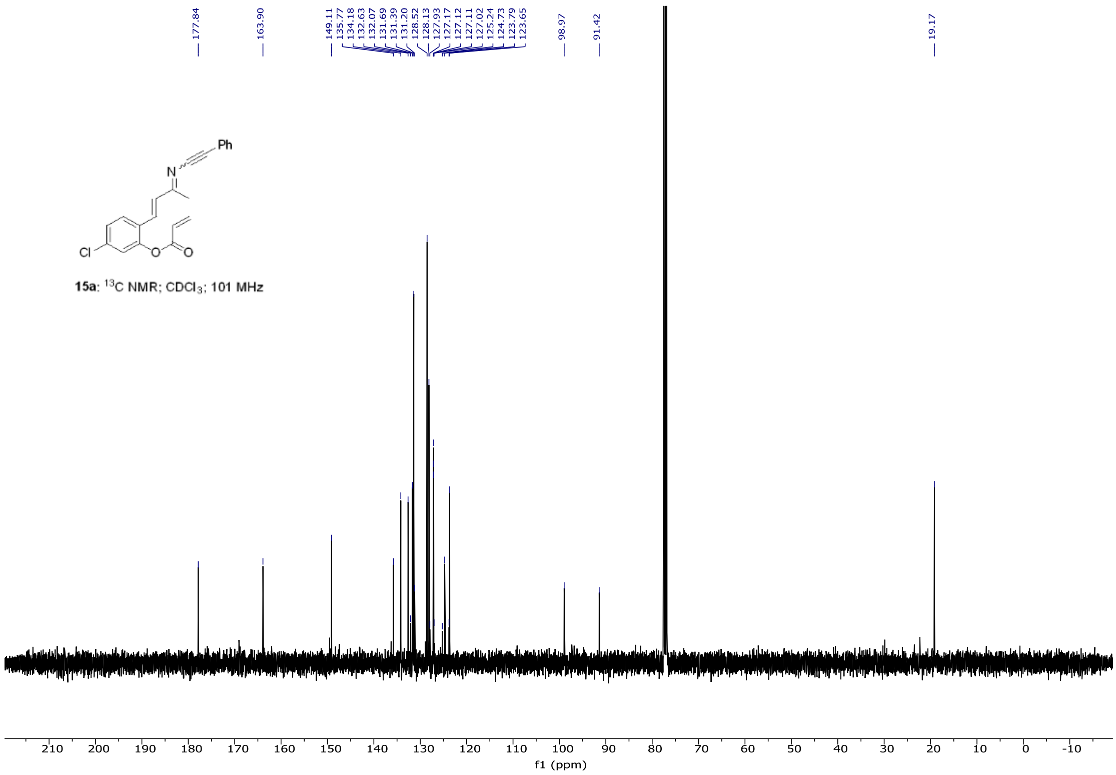

15a: ^{13}C NMR; $CDCl_3$; 101 MHz

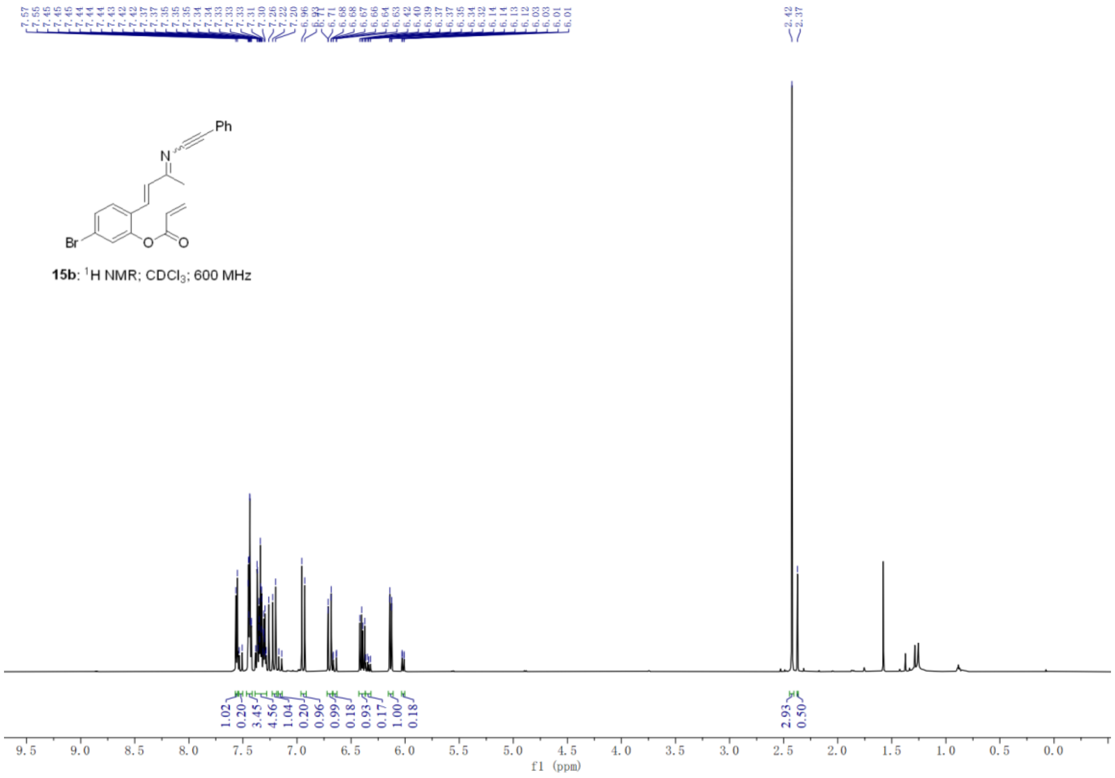
15b: 1H NMR; CDCl3; 600 MHz
f1 (ppm)

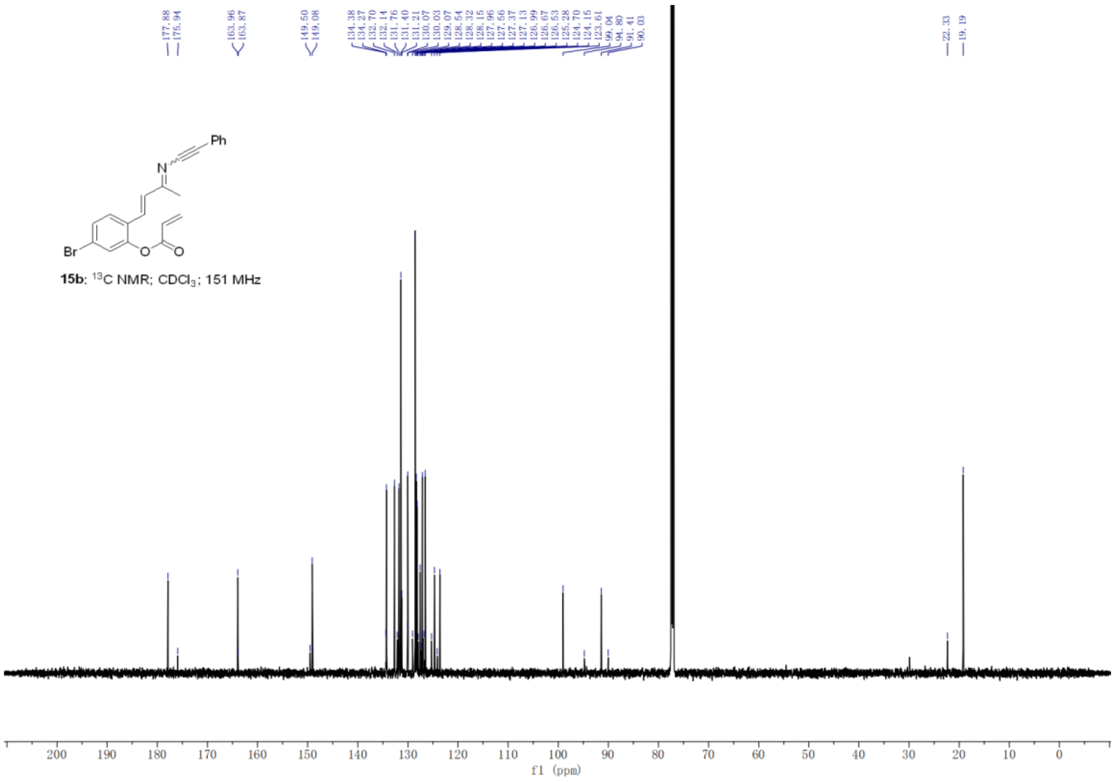
15b: 13C NMR; CDCl3; 151 MHz
f1 (ppm)

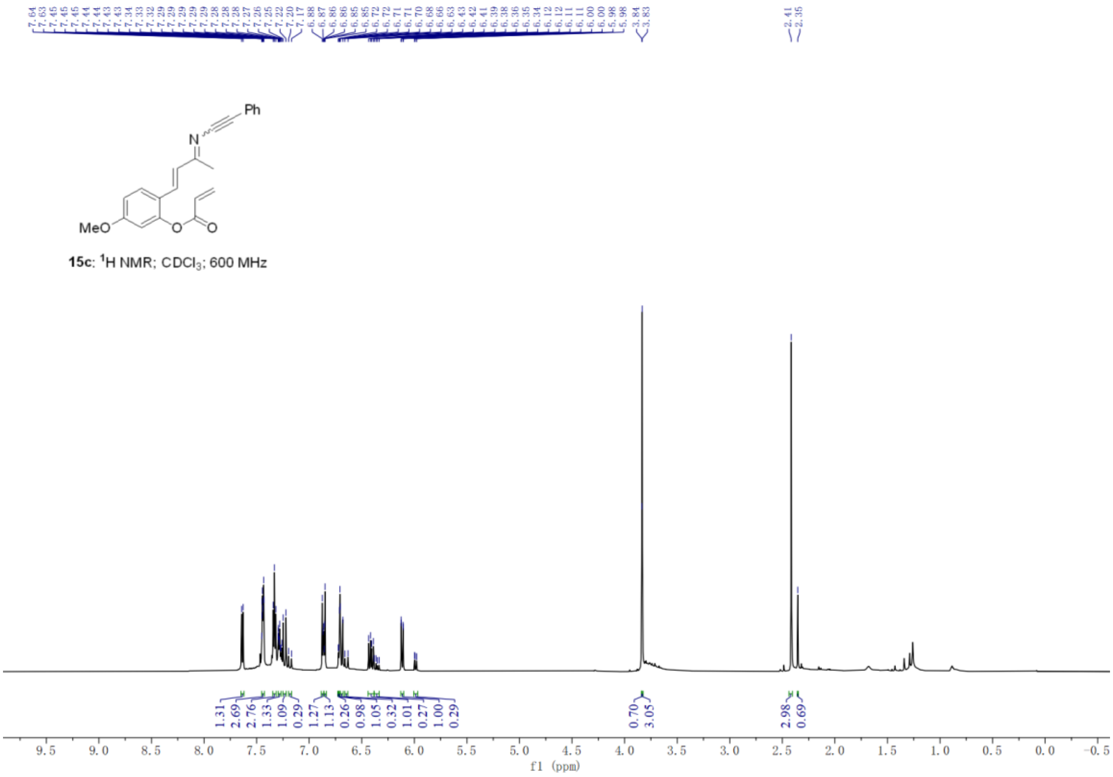

15c: ^{1}H NMR; $CDCl_3$; 600 MHz

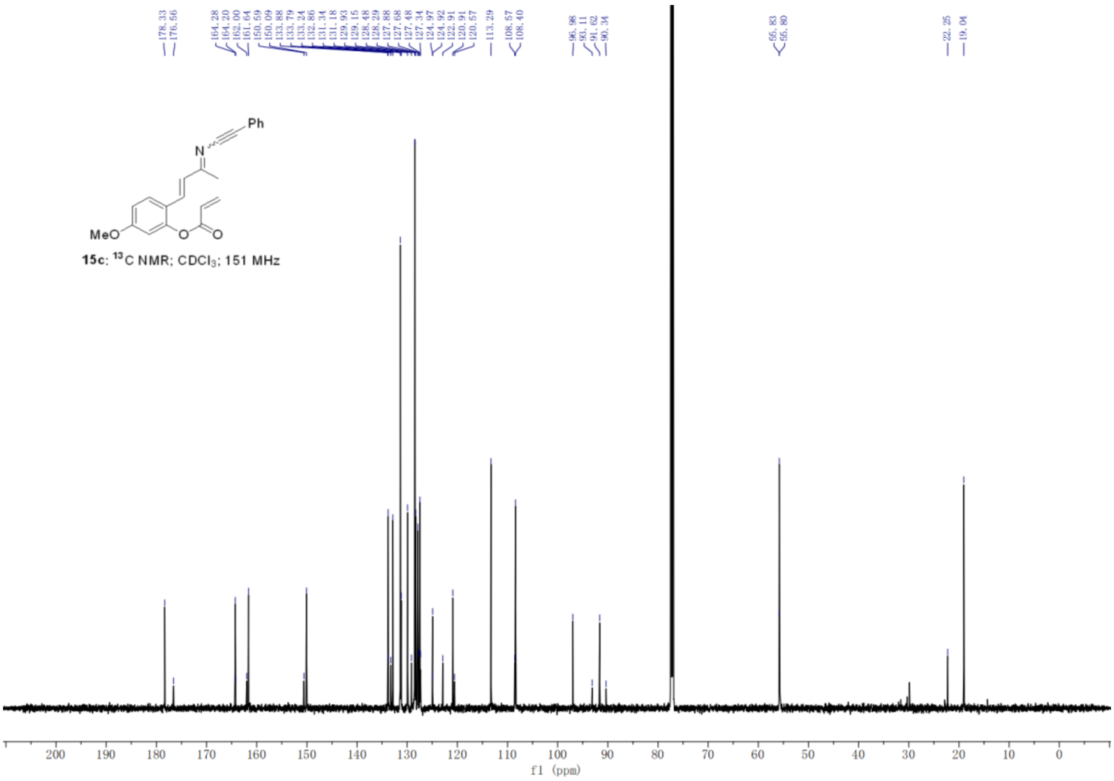

15c: ^{13}C NMR; $CDCl_3$; 151 MHz

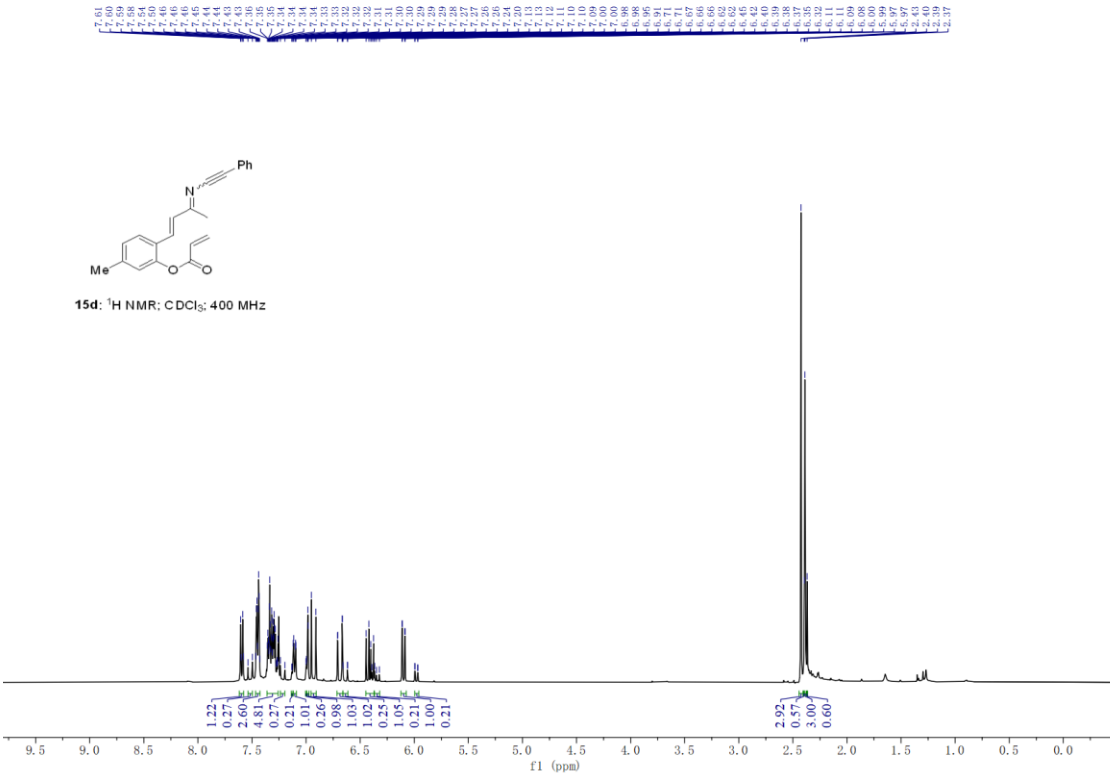

15d: ^{1}H NMR; $CDCl_3$; 400 MHz

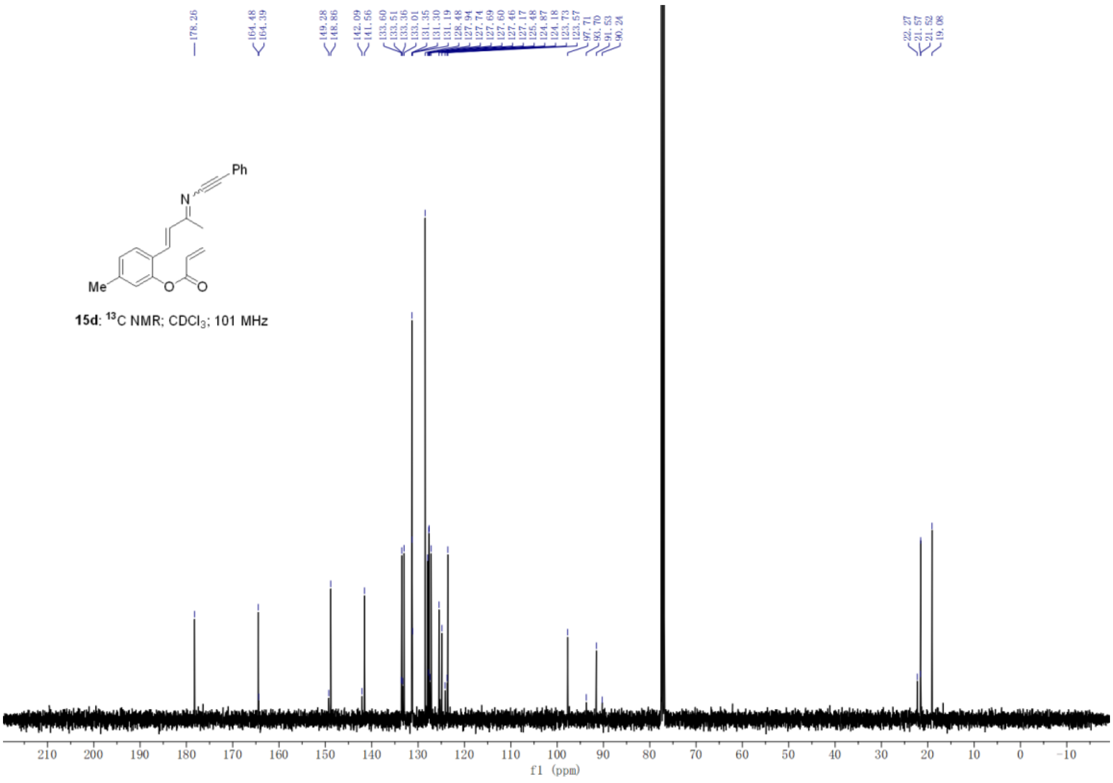

15d: ^{13}C NMR; $CDCl_3$; 101 MHz

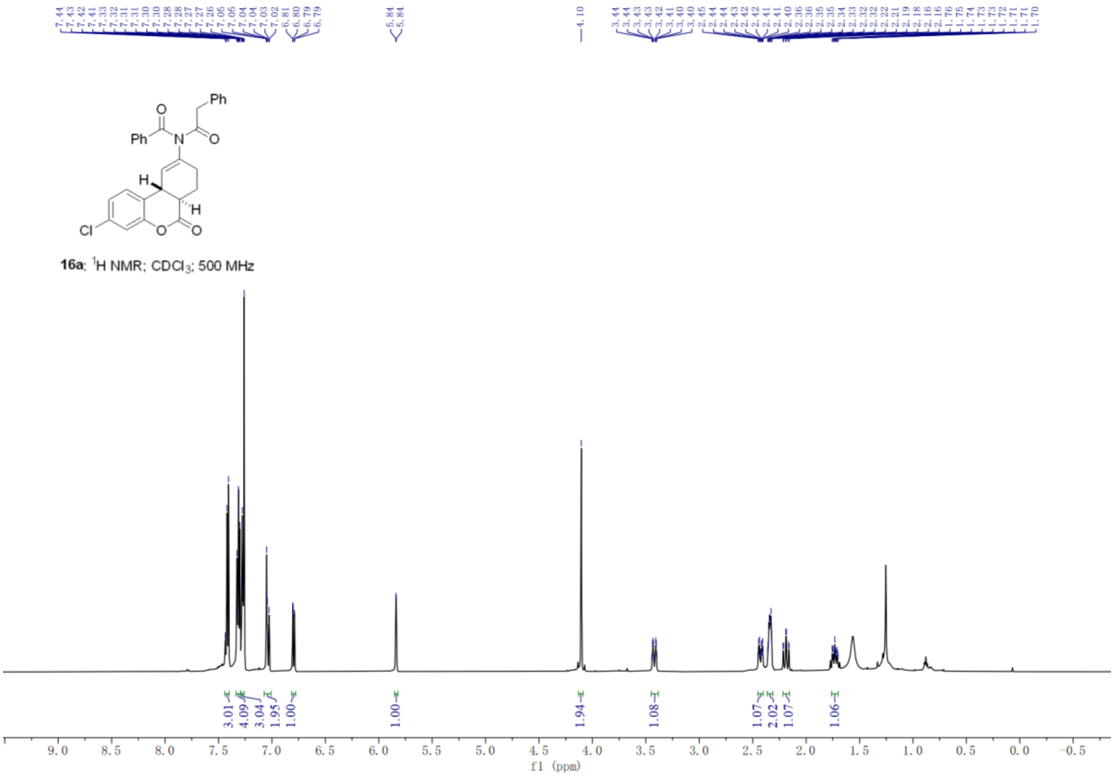

16a; ^{1}H NMR; $CDCl_3$; 500 MHz

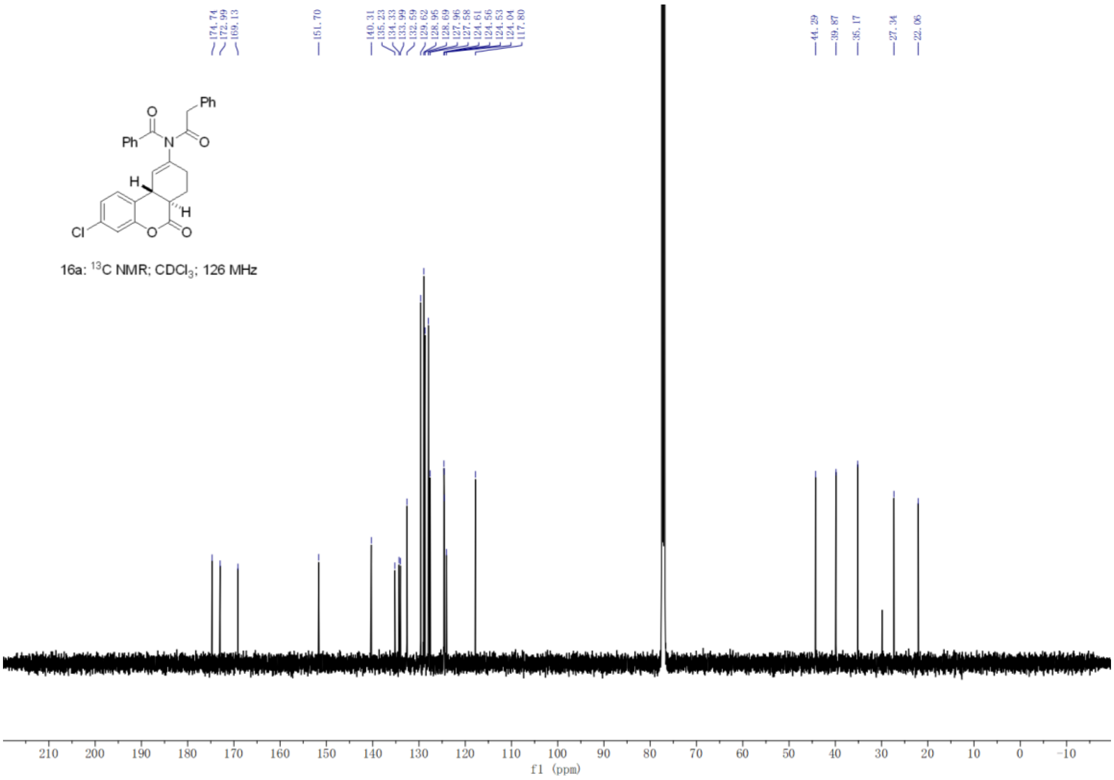

16a: ^{13}C NMR; $CDCl_3$; 126 MHz

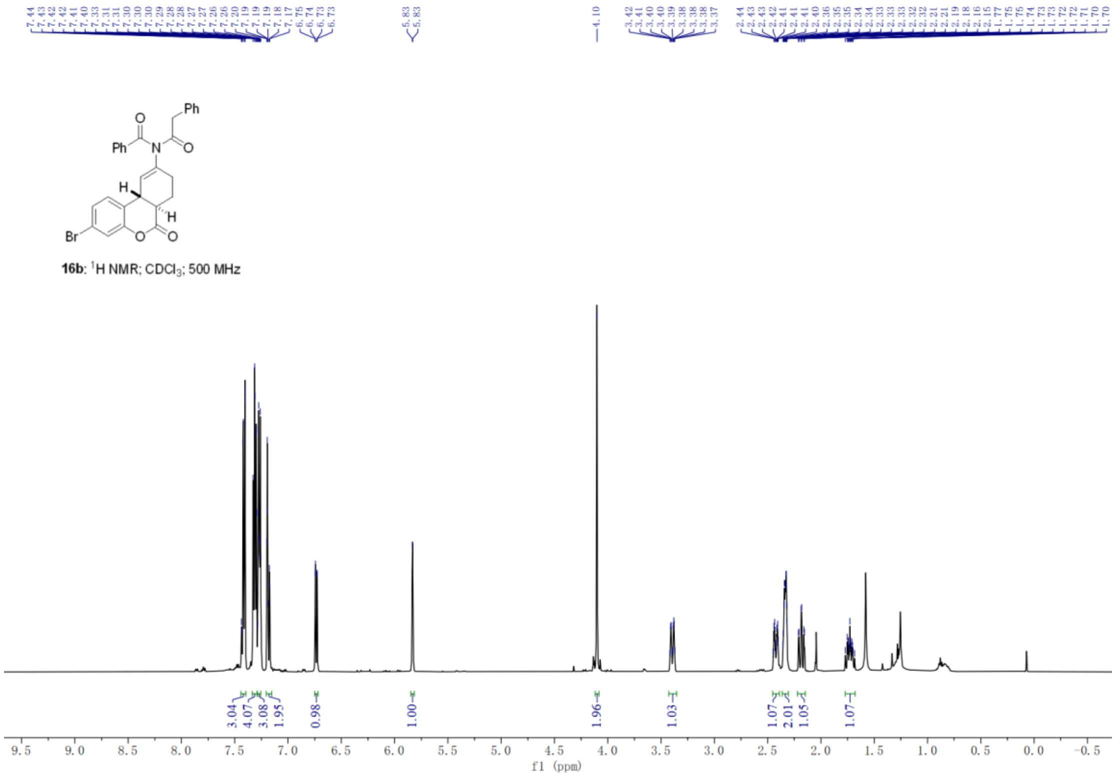
7.44 7.43 7.42 7.41 7.40 7.33 7.31 7.31 7.30 7.30 7.29 7.28 7.27 7.26 7.26 7.20 7.19 7.19 7.19 7.18 7.17 6.75 6.74 6.73 6.73
5.83 5.83
4.10
3.42 3.41 3.40 3.40 3.39 3.38 3.38 3.37
2.44 2.43 2.43 2.42 2.41 2.41 2.41 2.40 2.36 2.35 2.35 2.34 2.34 2.33 2.33 2.32 2.32 2.21 2.21 2.19 2.18 2.16 2.15 1.77 1.75 1.75 1.74 1.73 1.73 1.72 1.71 1.70 1.70
Ph
O
N
Ph
O
H
H
Br
O
O
16b: 1H NMR; CDCl3; 500 MHz
3.04 4.07 3.08 1.95 0.98 1.00 1.96 1.03 1.07 2.01 1.05 1.07
f1 (ppm)

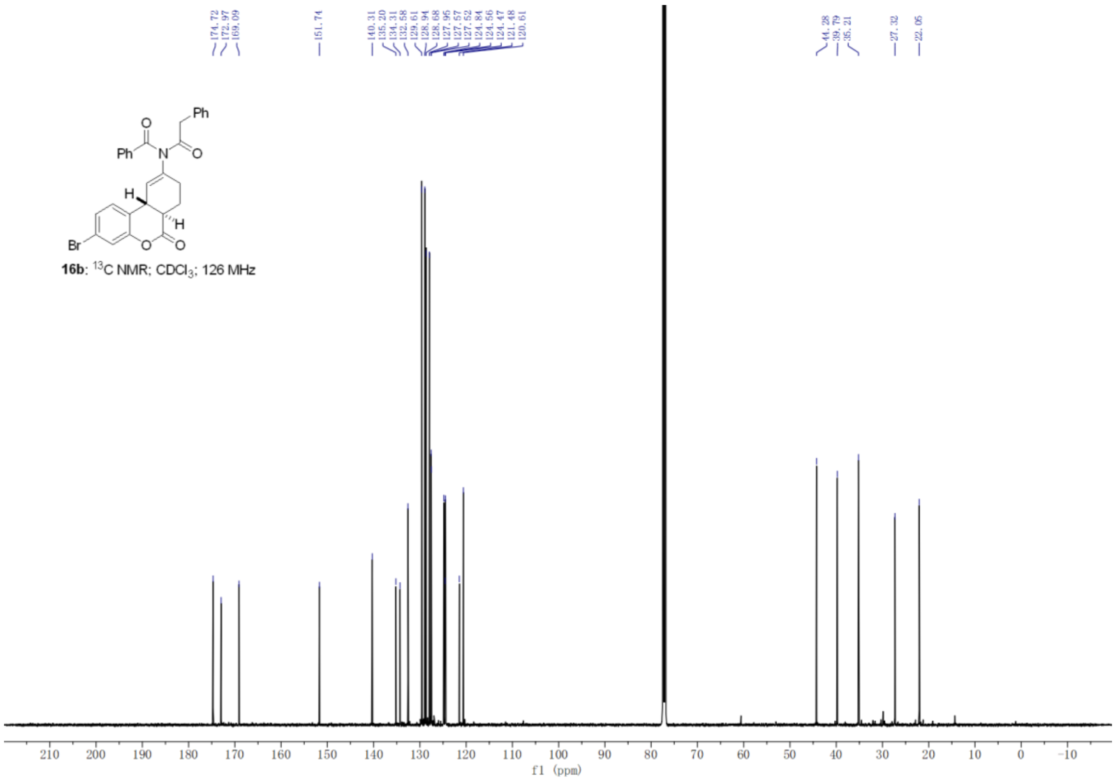
174.72 172.97 169.09
151.74
140.31 135.20 134.31 132.58 129.61 128.94 128.68 127.95 127.57 127.52 124.84 124.56 124.47 121.48 120.61
44.28 39.79 35.21
27.32
22.05
Ph
O
N
Ph
O
H
H
Br
O
O
16b: 13C NMR; CDCl3; 126 MHz
f1 (ppm)

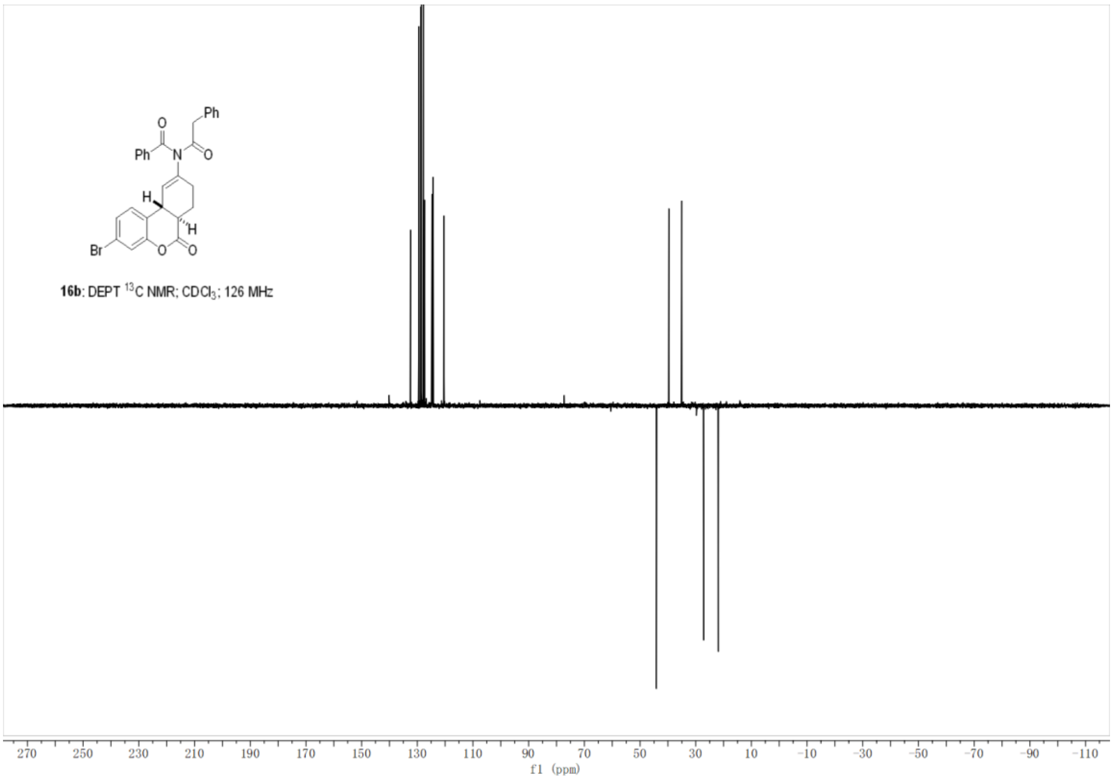
Ph
O
Ph
N
O
H
H
Br
O
O
16b: DEPT 13C NMR; CDCl3; 126 MHz
270 250 230 210 190 170 150 130 110 90 70 50 30 10 -10 -30 -50 -70 -90 -110
f1 (ppm)

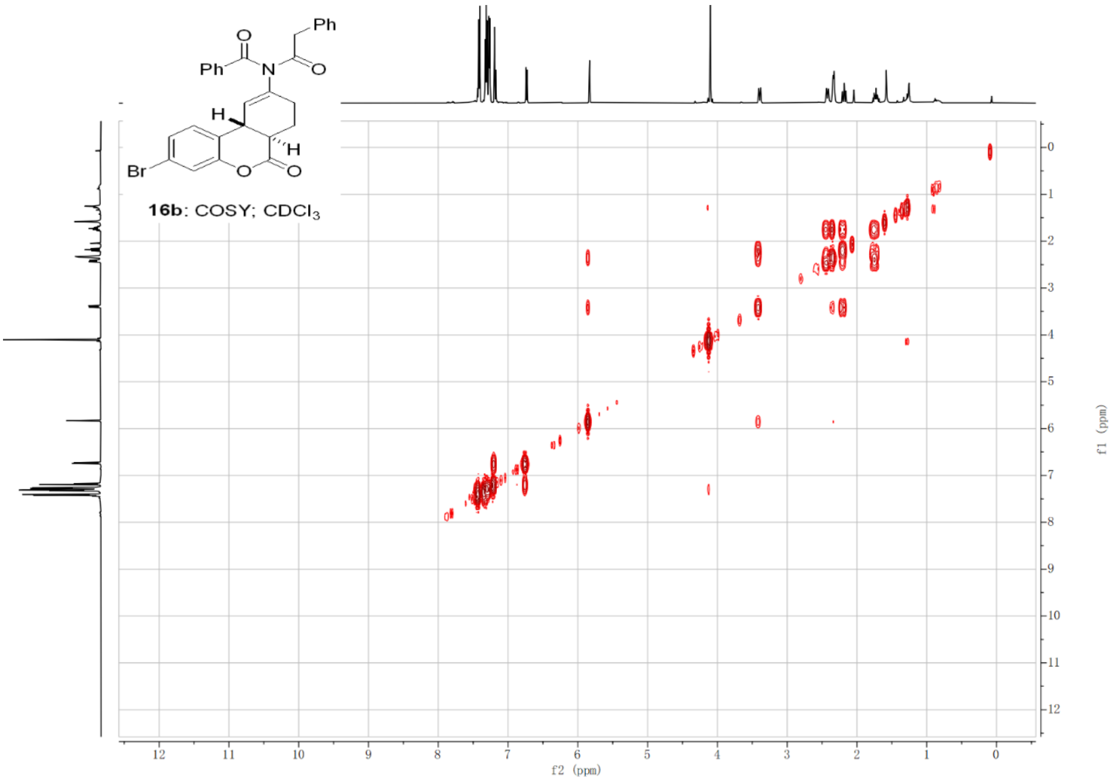
Ph
O
Ph
N
O
H
H
Br
O
O
16b: COSY; CDCl3
12 11 10 9 8 7 6 5 4 3 2 1 0
f2 (ppm)
f1 (ppm)

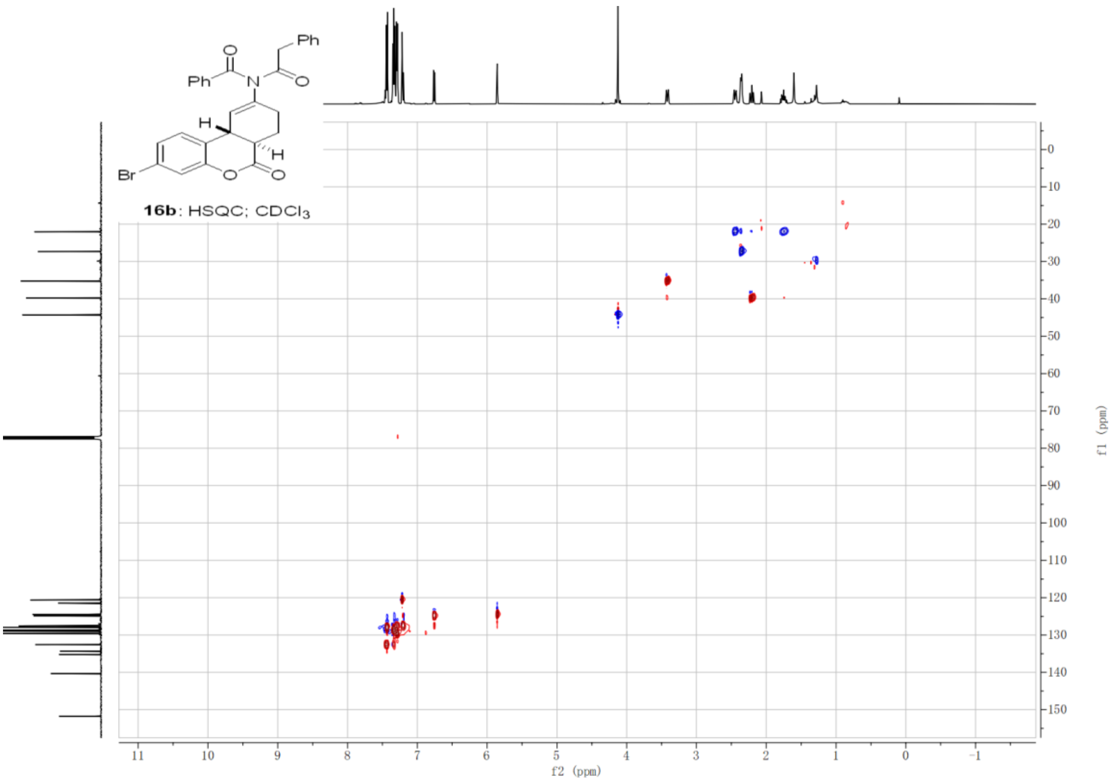
16b: HSQC; CDCl3
f1 (ppm)
f2 (ppm)

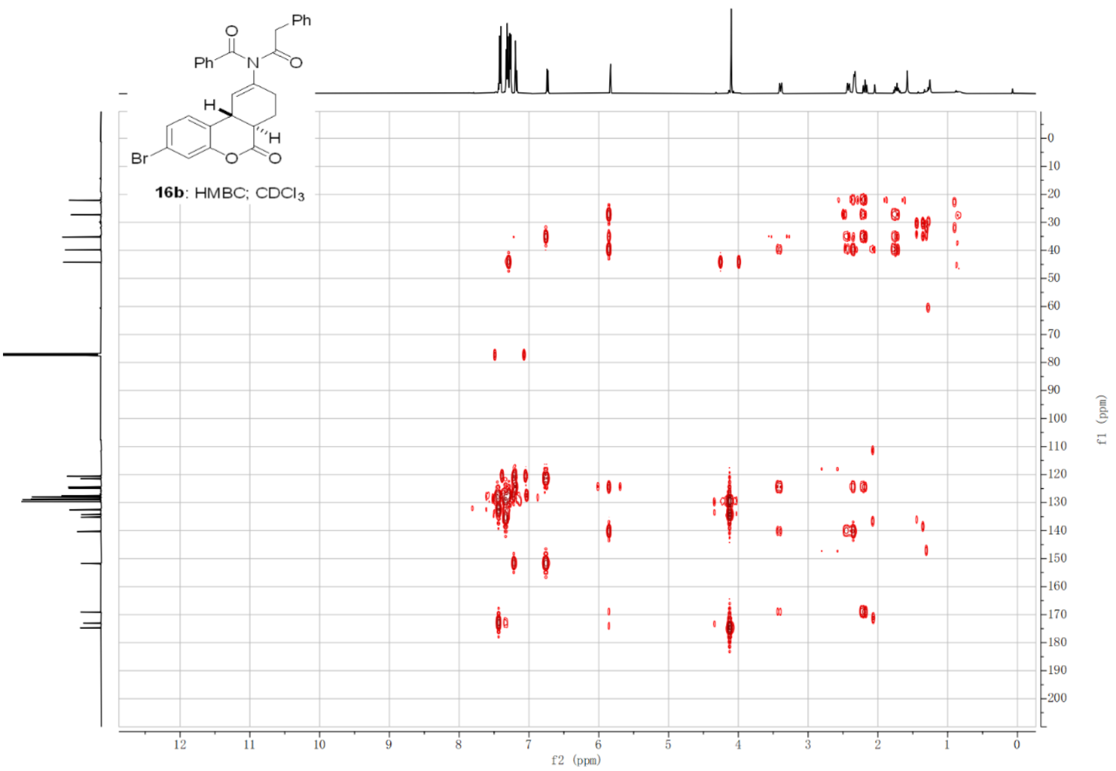
16b: HMBC; CDCl3
f1 (ppm)
f2 (ppm)

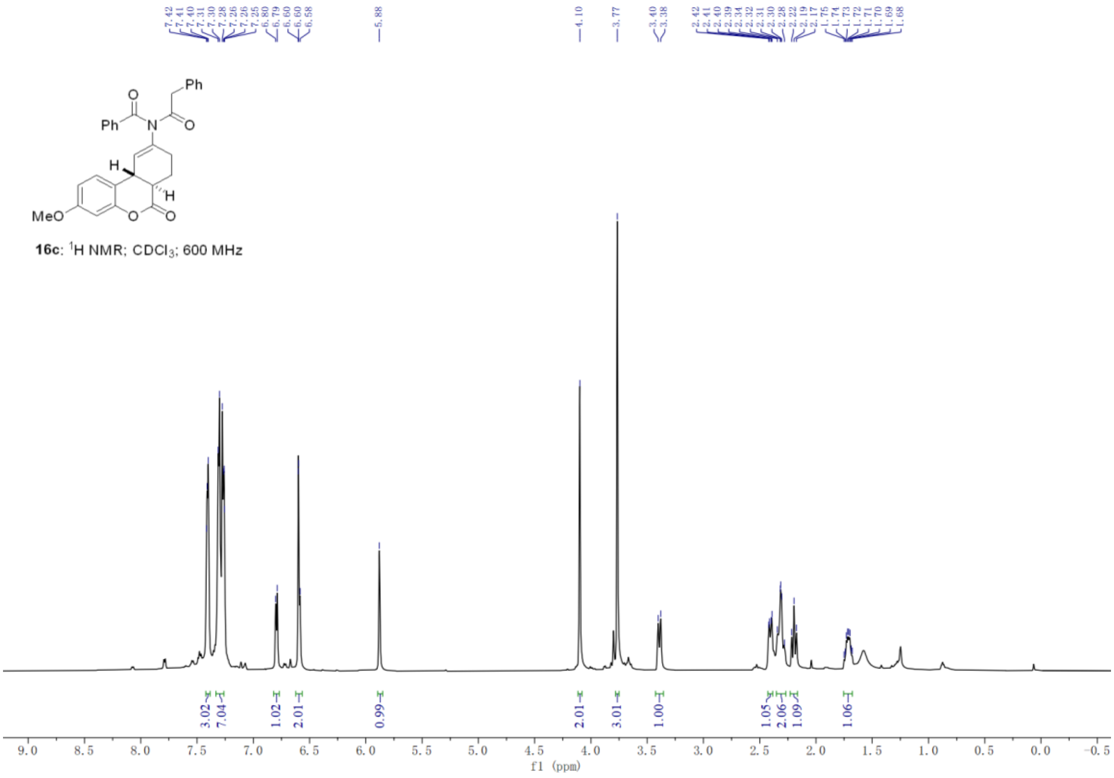
7.42 7.41 7.40 7.31 7.30 7.28 7.26 7.26 7.25 6.80 6.79 6.60 6.60 6.58
5.88
4.10
3.77
3.40 3.38
2.42 2.41 2.40 2.39 2.34 2.32 2.31 2.30 2.28 2.22 2.19 2.17 1.75 1.74 1.73 1.72 1.71 1.70 1.69 1.68
Ph
O
N
Ph
O
H
H
MeO
O
O
16c: 1H NMR; CDCl3; 600 MHz
3.02 7.04 1.02 2.01 0.99 2.01 3.01 1.00 1.05 2.06 1.09 1.06
f1 (ppm)

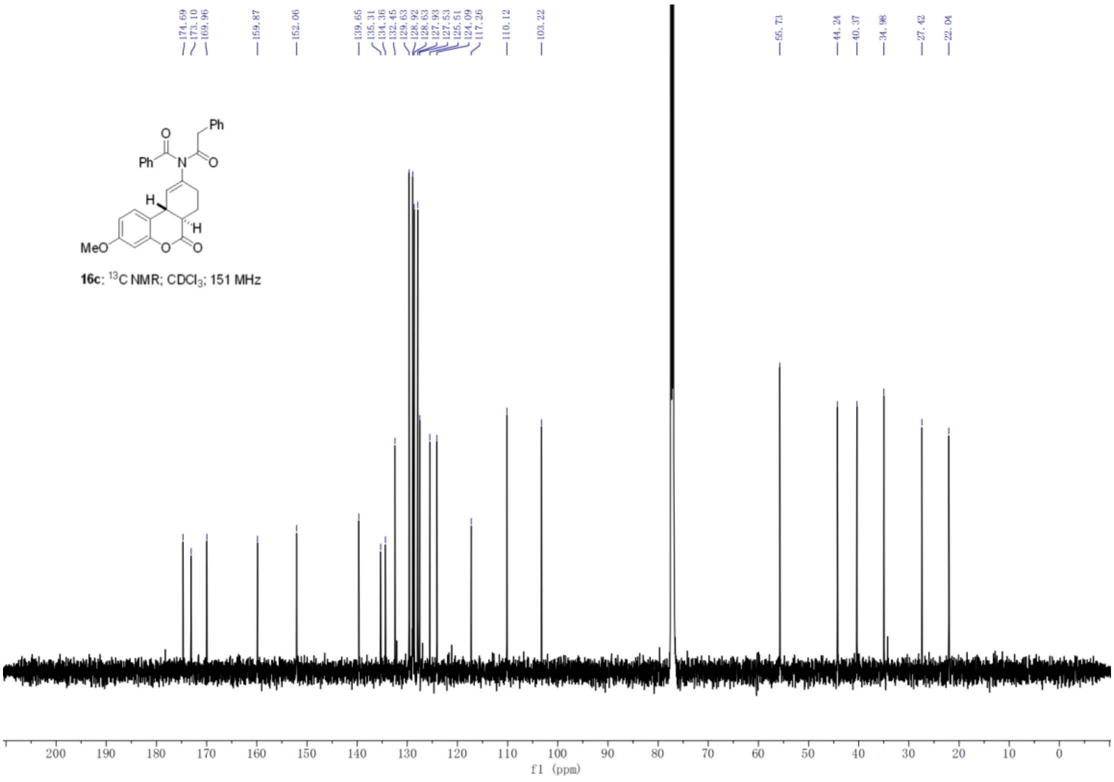
174.69 173.10 169.96
159.87
152.06
139.65 135.31 134.36 132.45 129.63 128.92 128.63 127.93 127.53 125.51 124.09 117.26
110.12
103.22
55.73
44.24 40.37 34.98 27.42 22.04
Ph
O
N
Ph
O
H
H
MeO
O
O
16c: 13C NMR; CDCl3; 151 MHz
f1 (ppm)

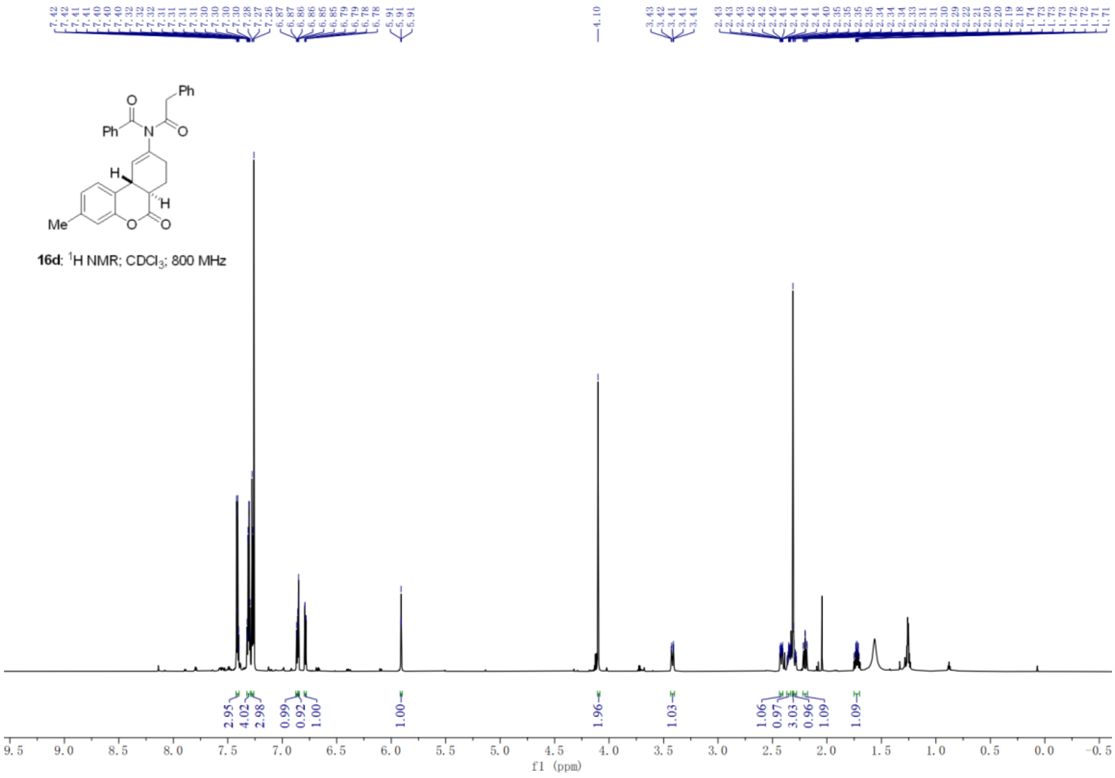
16d: 1H NMR; CDCl3; 800 MHz
f1 (ppm)

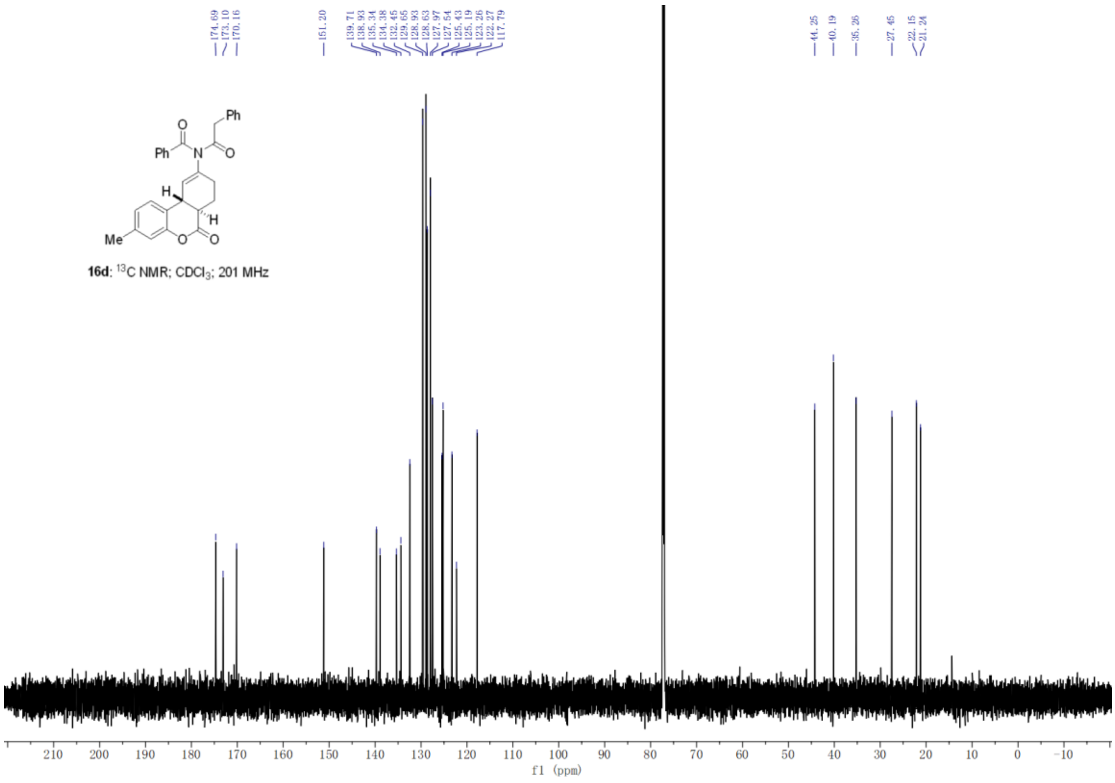
16d: 13C NMR; CDCl3; 201 MHz
f1 (ppm)

8. References:

1. R. Lavernhe, R. O. Torres-Ochoa, Q. Wang, J. Zhu, *Angew. Chem. Int. Ed.* **2021**, *60*, 24028-24033.

2. F. R. P. Crisóstomo, R. Carrillo, T. Martín, F. García-Tellado, V. S. Martín, *J. Org. Chem.* **2005**, *70*, 10099-10101.

3. M. K. Ghorai, A. Kumar, K. Das, *Org. Lett.* **2007**, *9*, 5441-5444.

4. K. Lang, C. Li, I. Kim, X. P. Zhang, *J. Am. Chem. Soc.* **2020**, *142*, 20902-20911.

5. O. S. Morozov, A. V. Lunchev, A. A. Bush, A. A. Tukov, A. F. Asachenko, V. N. Khrustalev, S. S. Zalesskiy, V. P. Ananikov, M. S. Nechaev, *Chem. Eur. J.* **2014**, *20*, 6162-6170.

6. J. Zhang, S. Zhang, H. Zou, *Org. Lett.* **2021**, *23*, 3466-3471.

7. G. S. Lee, S. H. Hong, *Chem. Sci.* **2018**, *9*, 5810-5815.

8. Y. Liu, P. Ji, J. Xu, Y. Hu, Q. Liu, W. Luo, C. Guo, *J. Org. Chem.* **2017**, *82*, 7159–7164.

9. G. M. Sheldrick, *Acta Cryst.* **2015**, *A71*, 3-8.

10. O. V. Dolomanov, L. J. Bourhis, R. J. Gildea, J. A. K. Howard, H. Puschmann, *J. Appl. Cryst.* **2009**, *42*, 339-341.

11. G. M. Sheldrick, *Acta Cryst.*, **2008**, *A64*, 339-341.
